# Transformation of a Metal Chelate into a “Catch and Anchor” Inhibitor of Botulinum A Protease

**DOI:** 10.3390/ijms24054303

**Published:** 2023-02-21

**Authors:** Lucy Lin, Ealin N. Patel, Alexander L. Nielsen, Lewis D. Turner, William H. Tepp, Kong Nguyen, Sabine Pellett, Kim Janda

**Affiliations:** 1 Department of Chemistry and Immunology, The Skaggs Institute for Chemical Biology, Worm Institute of Research and Medicine (WIRM), The Scripps Research Institute, La Jolla, CA 92037, USA; 2 Department of Bacteriology, University of Wisconsin, 1550 Linden Drive, Madison, WI 53706, USA; 3 Atomwise Inc., 717 Market Street, Suite 800, San Francisco, CA 94103, USA

**Keywords:** botulinum neurotoxin, catch and anchor inhibition, covalent inhibitors, structure-based drug design

## Abstract

Targeting the botulinum neurotoxin light chain (LC) metalloprotease using small-molecule metal chelate inhibitors is a promising approach to counter the effects of the lethal toxin. However, to overcome the pitfalls associated with simple reversible metal chelate inhibitors, it is crucial to investigate alternative scaffolds/strategies. In conjunction with Atomwise Inc., in silico and in vitro screenings were conducted, yielding a number of leads, including a novel 9-hydroxy-4*H*-pyrido [1,2-a]pyrimidin-4-one (PPO) scaffold. From this structure, an additional series of 43 derivatives were synthesized and tested, resulting in a lead candidate with a *K*_i_ of 150 nM in a BoNT/A LC enzyme assay and 17 µM in a motor neuron cell-based assay. These data combined with structure-activity relationship (SAR) analysis and docking led to a bifunctional design strategy, which we termed “catch and anchor” for the covalent inhibition of BoNT/A LC. Kinetic evaluation was conducted on structures prepared from this catch and anchor campaign, providing *k*_inact_/*K*_i_ values, and rationale for inhibition seen. Covalent modification was validated through additional assays, including an FRET endpoint assay, mass spectrometry, and exhaustive enzyme dialysis. The data presented support the PPO scaffold as a novel candidate for targeted covalent inhibition of BoNT/A LC.

## 1. Introduction

Botulinum neurotoxin A (BoNT/A) is the most toxic substance known, with an estimated i.v. lethal dose (LD_50_) of 1–2 ng/kg in humans [1]. It is a 50 kDa protease secreted by the bacterium *Clostridium botulinum* and related strains found in soil. BoNTs have found extensive use in cosmetics and therapeutics for an increasing number of indications, including pain management, urinary incontinence, and muscle spasms [2]. While the use of BoNTs is generally considered safe, off-target effects and adverse events can result in anaphylaxis, muscle weakness, dysphagia, and iatrogenic botulism [3]. BoNTs A, B, and, less commonly, E and F are capable of intoxicating humans, causing botulism. In humans, botulism causes flaccid paralysis, which can result in life-threatening conditions due to asphyxiation of the diaphragm. There are a few hundred annual cases of botulism in the United States, mainly stemming from improper food preparation or wound infections by *Clostridium botulinum* [4,5]. Despite this rarity, the Center for Disease Control (CDC) has designated BoNTs as a Category A bioterrorism threat owing to their extreme toxicity, simplicity of preparation, ease of potential dissemination, and historical evidence of their attempted use [1,6,7]. A single bioterrorism event could easily overwhelm lean healthcare infrastructure, as ventilators and long-term hospitalization are required for treatment. The current standard of care for botulism is treatment with an equine-derived antibody, most effective if administered within 24 h of intoxication [8,9]. Because antibodies are incapable of sequestering and inhibiting BoNTs after they enter neuronal compartments, they become significantly less effective after this initial window has passed. In addition, there is a risk of adverse reactions such as anaphylaxis occurring. Patients are required to remain hospitalized for weeks to months, often on mechanical ventilation and parenteral nutrition, during the course of treatment [6,7,10].

Many small-molecule drugs are capable of penetrating cells and are thus a promising strategy for meeting this unmet need. The BoNT holotoxin is composed of a heavy chain (HC) translocation domain that mediates the internalization of the light chain (LC), a zinc-dependent metalloprotease, into motor neurons. Thus, non-peptidic small-molecule inhibitors of BoNT usually target the active site of BoNT LC and typically contain a metal binding group (MBG) [11]. However, no such inhibitors have progressed to clinical trials. The difficulty of development can be partially attributed to the disparity between the half-life of BoNT/A LC in neurons and the drug in the body—the former persists for months, while the latter is cleared over a time frame of hours to days [12,13]. In our search for a clinically viable inhibitor of BoNT/A LC, we pivoted from the design of conventional reversible inhibitors to what we term a “catch and anchor” strategy. The general concept involves the tethering of a covalent warhead to an active-site inhibitor scaffold. Moreover, this approach leverages the high affinity afforded by an MBG, the specificity and complete inhibition of an active-site inhibitor, and the longevity of the covalent adduct.

Despite its putative low reactivity, Cys165 remains an attractive modality for covalent modification due to its position near the active site [14]. Despite not being involved directly in the catalytic cycle or structural stability, Cys165 is an important residue, as its mutation is associated with a 50-fold decrease in catalytic activity [15]. It is also understood that the active site of BoNT/A LC tolerates a significant amount of plasticity while maintaining the same conformation of zinc and the catalytic residues; however, the catalytic cleft microenvironment can be altered depending on the inhibitor that is bound [16,17]. Moreover, hydroxamic acids, the most commonly used MBG for BoNT/A LC inhibition, face significant stability issues in the physiological environment [18,19]. Thus, by investigating new inhibitor scaffolds, we aim to address the drawbacks of our previous efforts, namely stability, cell permeability, and potency. To this end, we describe the discovery and development of a new class of BoNT/A LC inhibitors and their subsequent advancement to a targeted covalent inhibitor strategy.

## 2. Results and Discussion

### 2.1. Screening Campaign

**Figure 1:**
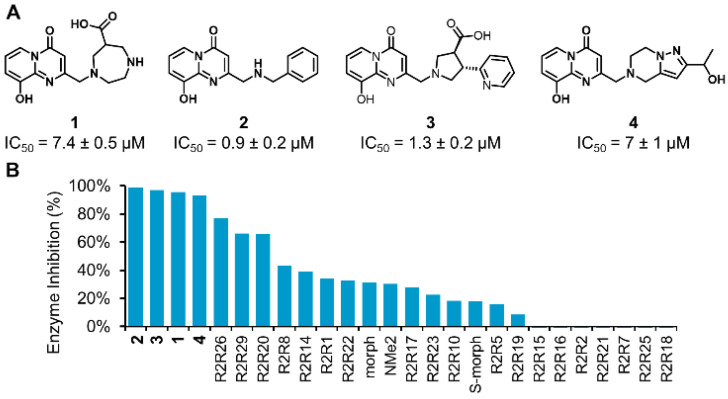
(**A**) Structures and IC_50_ of initial lead compounds (**1**–**4**). Values are reported as mean ± SD, *n* = 3. (**B**) Screening results of the PPO sub-library, tested at 40 µM in duplicate. Data were normalized to a DMSO control.

In collaboration with Atomwise Inc., the search began with an in silico screen of inhibitors using the company’s AtomNet^®^ model. The AtomNet^®^ model is the first deep neural network for structure-based drug discovery [20]. A single global AtomNet^®^ model was trained on a large number of protein structures and bioactivity data of small-molecule binders, as previously described [16]. The sglobal AtomNet^®^ model was then used to screen the eMolecules diverse library of 1 million molecules against the X-ray crystal structure of 2,4-dichlorocinnamyl hydroxamic acid bound to BoNT/A LC (PDB 2IMA) [16]. The 33 top-scoring hits were purchased from eMolecules and screened at a final concentration of 40 μM against BoNT/A LC in the SNAPtide FRET assay, a robust FRET assay that has led to the discovery of inhibitors with in vivo efficacy [21,22,23,24]. This screening effort yielded 9-hydroxy-4*H*-pyrido [1,2-a]pyrimidin-4-one (PPO)-based **1** (Figure 1A) as a top hit. Next, a focused sub-library of 26 derivatives was subsequently ranked in silico with AtomNet and screened within the SNAPtide FRET assay (Figure 1B). Four members (including **1**) of the PPO sub-library inhibited BoNT/A LC activity by more than 90%. Compounds **2** and **3,** both of which contained six-membered heterocycles, were found to be approximately seven-fold more potent than initial lead **1** (Figure 1A). Based upon these results, **2** was selected for further optimization due to its inhibitory potency, ease of synthesis, and high potential for derivatization due to the secondary amine embedded within its structure. In passing, we note that the chiral centers found within the pyrrolidine ring of the equipotent compound **3** would likely require undesirable synthetic complexity; hence, this compound was abandoned for fear of impeding forthcoming medicinal chemistry efforts.

### 2.2. BoNT/A LC Inhibition & SAR

**Scheme 1:**
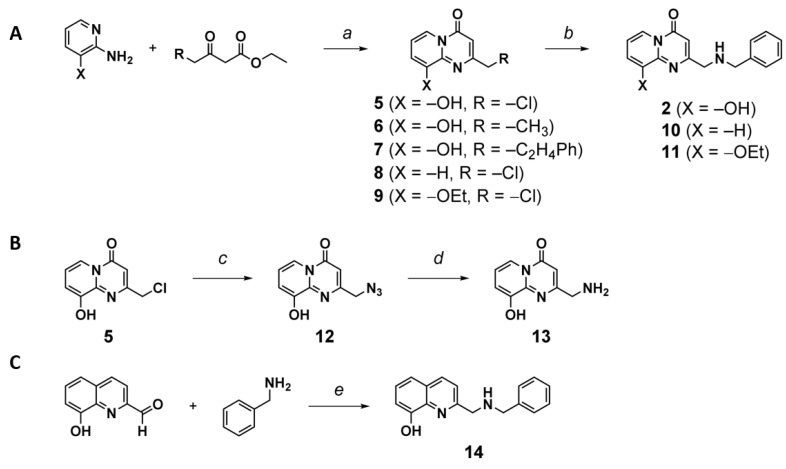
Synthesis of the PPO scaffold and compounds. (**A**) Synthesis of PPO scaffold and benzylamine derived compounds. Reagents and conditions: (a) polyphosphoric acid, 110 °C, 4 h, 25–74%; (b) benzylamine, DMF, rt, 18 h, 16–32%; (**B**) Synthesis of compound **13**. Reagents and conditions: (c) sodium azide, DMF, rt, 2 h, 83%; (d) triphenylphosphine, H_2_O/THF, 60 °C, 4 h, 27%; (**C**) Synthesis of compound **14**. Reagents and conditions: (e) sodium triacetoxyborohydride, DCE, 0 °C to rt, 18 h, 84%.

The PPO scaffold was further probed by syntheses of compounds **5**–**14** (Scheme 1) to determine the minimum pharmacophore. Analogous to the majority of non-peptidic small-molecule inhibitors of BoNT/A LC, PPO is a metal chelating scaffold that binds Zn^2+^ with the oxygen of its hydroxyl group and the nitrogen in the pyridopyrimidinone heterocycle [8]. The exclusion of the hydroxyl group (**10**) resulted in the complete ablation of inhibitory activity, while masking the hydroxyl functionality with an ethyl group (**11**) had a similar effect, which is consistent with its known metal binding mechanism [8].

To examine the impact of the benzylamine substituent, the terminal methyl analogue (**6**) and the primary amine (**13**) were synthesized. The loss of phenyl groups resulted in a decrease in inhibitory potency for both compounds. This, in combination with the complete lack of inhibitory activity of intermediates, suggests that the benzyl group interacts favorably with the active site and that the Zn-chelating group alone is not sufficient for binding. Additionally, based on the IC_50_ values of **1**–**4**, it is evident that a variety of heterocycles can be tolerated. We also posit that the nitrogen atom plays a role in potency, as replacing the secondary amine with a methylene group to granting **7** resulted in a drastic decrease in inhibitory activity. This reduction in inhibitory activity suggests that the secondary amine is not directly involved in metal chelation but likely engages in hydrogen bond interactions. Finally, quinolinol derivative **14** showed a 15-fold decrease in inhibition, implying that the PPO carbonyl may promote additional hydrogen bonding interactions.

As determined in past SAR campaigns, which also align with crystallographic analysis, aromatic groups tend to sit in the hydrophobic pocket, which contains aromatic residues amenable for π-π stacking [3,9,10,17]. Hence, the PPO-SAR study was focused on this region of the molecule. Compounds were synthesized from their corresponding benzylamines, benzyl alcohols, and N-containing heterocycles under the same conditions as those presented in Scheme 1A, and IC_50_ values were obtained and summarized in Table 1. Based on the trends observed for benzylamine derivatives **2**–**29**, some general conclusions could be drawn. Irrespective of their electronic properties, 3′-substituted molecules were around 3-fold more potent than their 4′-substituted counterparts, which suggests that activity depends less on electrostatic interactions but more on the positioning that the substituent occupies in the active site. This effect can be seen by comparing methyl ester structural isomers **24** and **25**; 3′-substituted methyl ester **24** is among the most potent of benzylamine derivatives, with an IC_50_ of 0.41 ± 0.08 µM. Conversely, the 4′-substituted analogue **25** is poorly positioned, so that inhibition is abolished (IC_50_ > 64 µM). Interestingly, the addition of a phenyl group to the benzyl ring at the 4′-position (**27**) significantly increased inhibitory potency compared to **2**, while substitution at the 3′-position proved slightly less potent (**26**). The *K*_i_ of compound **27** was calculated to be 150 nM based on the Cheng-Prusoff equation for a competitive inhibitor, where substrate *K*_m_ = [S] [25].

Past studies have reported that the BoNT/A LC active site can accommodate very large substituents and that inhibitor binding is then accompanied by a significant rearrangement of hydrophobic side chains in the active site, with retention of the geometry of the catalytic triad. This can be seen in the structural differences between X-ray crystal structures bound with the small 2,4-dichlorocinnamyl hydroxamic acid and a large adamantane hydroxamic acid inhibitor (PDB 2IMA and 4HEV, respectively) [3,9]. It is possible that the active site of BoNT/A LC accommodates the large PPO inhibitors in a similar manner.

Due to the protonation of the secondary amine at physiological pH, cell permeability could be compromised and therefore impact efficacy. Thus, a series of benzyl ether derivatives (**30**–**44**, Table 1) were synthesized to replace the amine, which afforded a series of 10-fold less potent analogues compared to the benzylamine series. The two main consequences of replacing the amine with an oxygen atom are the loss of an H-bond donor and the change in bond geometry. It is possible that both contribute to the lower inhibitory potency of this series. In addition to benzylamine and benzyl ethers, a limited series of isoindoline and indoline derivatives were prepared and examined. The isoindolines can be thought of as a constrained benzyl group and were synthesized to offer some insight into benzyl group positioning. Interestingly, we found that isoindoline **42** was equipotent to **2**. This lack of improvement in potency suggests that this is not likely the optimal orientation of the benzyl group, while any benefit stemming from the reduction in entropy afforded by the restrained benzyl group of **42** could be counteracted by slightly less favorable binding.

**Figure 2:**
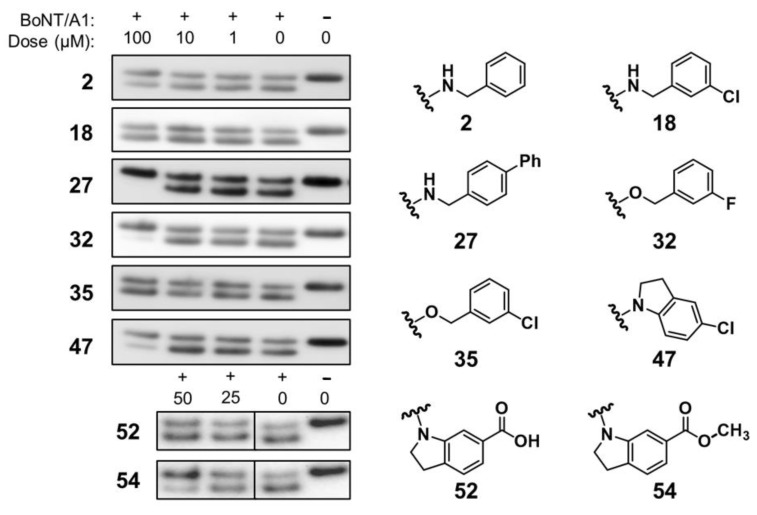
Cellular evaluation of PPO inhibitors. Western blots are representative images of each sample, full images can be found in Figure S1. The upper bands represent intact SNAP-25, and the lower bands represent cleaved SNAP-25. The positive control group was exposed to BoNT/A, and the negative control group was not. Both control groups were treated with cell media containing 1% DMSO.

To validate the inhibition of BoNT/A LC by the PPO compounds detailed, *vide supra*, a number of the most potent derivatives were evaluated in a cell assay that quantifies intact and cleaved SNAP-25 in human-induced pluripotent stem cell (hiPSC)-derived motor neurons. This cell assay has been used extensively to evaluate BoNT/A inhibitors, offering further insight into the external factors that affect inhibitor potency. The compounds presented in Figure 2 were selected based on potency and diversity. Surprisingly, neither **2** nor **18** inhibited the cleavage of SNAP-25 in the cellular assay despite their excellent inhibitory activity in the enzyme assay. We posit that the lack of inhibitory activity could be related to poor cell permeability stemming from the polarity of the secondary nitrogen, which would exist primarily in the protonated state at physiological pH. Notably, benzyl ether derivative **32**, which is nearly 10-fold less potent than **2** in the enzyme assay (Table 1), showed nearly complete protection of SNAP-25 at 100 µM. Similarly, isoindolines **47** and **54** both protect against SNAP-25 cleavage at 100 µM, although to a lesser degree. Interestingly, although **35** is also a benzyl ether derivative, it did not have any apparent effect on SNAP-25 cleavage. In sum, of the compounds tested, **27** was the most potent, with an IC_50_ of 13 µM (12–16 µM, 95% CI).

### 2.3. Docking-Based Covalent Linker Design

**Figure 3:**
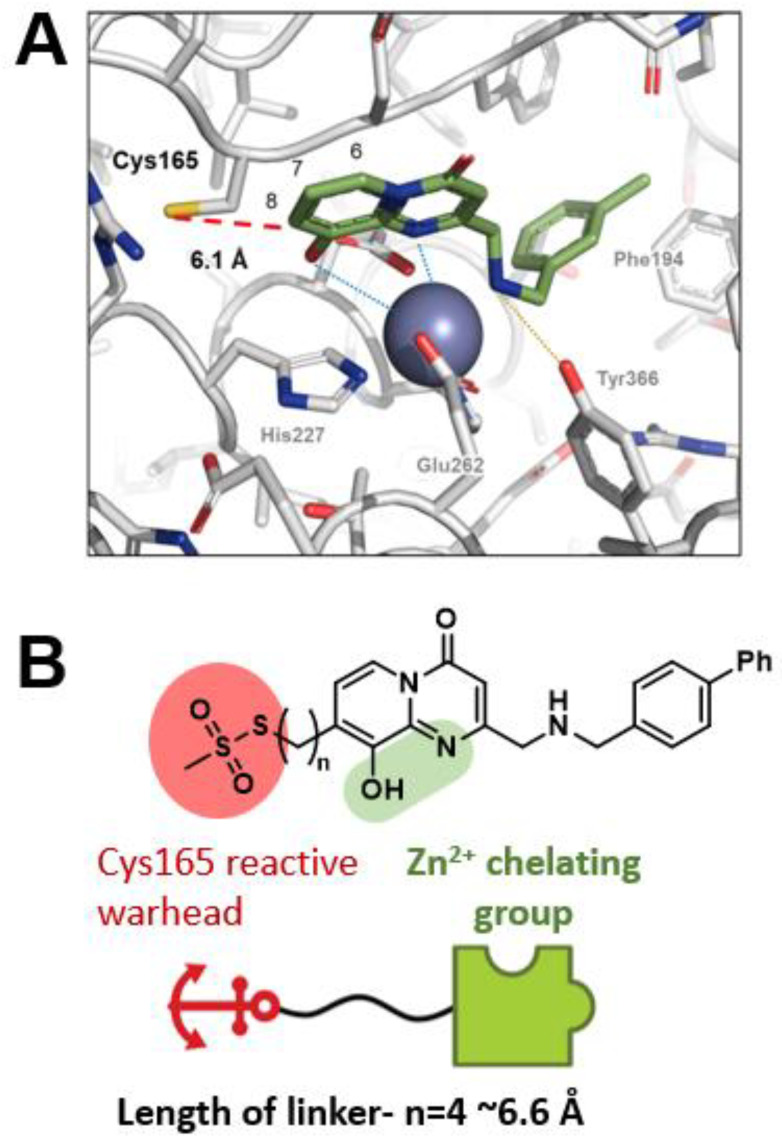
(**A**) Docking of compound **22** (green) at the active site of BoNT/A LC (PDB 4HEV). The dark gray sphere represents Zn^2+^. (**B**) Covalent linker design of “catch and anchor” inhibitor compounds **80**–**84** based on docking.

The promising inhibitory potency in both the in vitro enzyme and cell assays prompted the selection of **27** for lead development as a catch and anchor inhibitor. An X-ray crystal structure of BoNT/A LC co-crystallized with an adamantane inhibitor was used for docking (Figure 3A), as the larger hydrophobic pocket generated from this structure could accommodate the bulkier structural sizes currently under investigation [26]. Based on the lowest energy poses, it appeared that the heteroatom can form a hydrogen bond with Tyr366, echoing the experimental data obtained. The lowest-scoring compounds were molecules that did not interact with the hydrophobic pocket, and correlated with poor IC_50_ values.

**Scheme 2:**
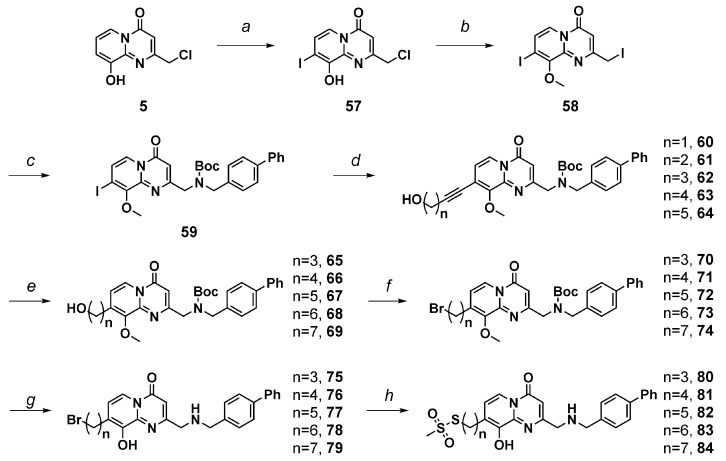
Synthesis of catch and anchor PPOs. Reaction conditions: (a) Iodine, hydrogen peroxide (30% aq.), EtOH, rt, 20 h, 91%; (b) methyl iodide, potassium carbonate, DMF, 0–4 °C, 4 h, 85%; (c) 4-phenyl benzylamine, DMF, rt, 1 h; di-tert-butyl carbonate, DMAP, DMF, rt, 18 h (50%, two steps); (d) Pd(PPh_3_)_2_Cl_2_, copper (I) iodide, Et_3_N, DCM, 18 h, rt, 53–84%; (**e**) H_2_, Pd/C (10% loading), EtOH, 3 h, rt, (72–100%); (f) carbon tetrabromide, triphenylphosphine, DCM, rt, 4 h, (18–53%); (g) BBr_3_, DCM, rt, 5 h, quantitative; (h) sodium methanethiosulfonate, EtOH, reflux, 16 h, (20–44%).

As detailed, vide supra, this strategy combines an active site binding scaffold with a pendent Cys-reactive warhead to achieve targeted covalent inhibition of BoNT/A LC [15,22]. Moreover, the success of the catch and anchor strategy is dependent on the proximity of the reactive warhead to the cysteine residue of interest, while simultaneously engaging the metal center. Based on the lowest energy position of the PPO derivatives shown in Figure 3A, it was determined that extension from position 8 would provide the optimal vector for a reactive warhead to engage with Cys165, with an approximate distance of 6–7 Å between C8 and Cys165. Using docking as our guide, a preliminary molecule was sought with a thiol-reactive methanethiosulfonate (MTS) warhead, and a linker length predicted to be *n* = 4, approximately 6.6 Å (Figure 3B). Ease of attachment and high thiol reactivity make MTS a useful tool for optimizing linker position and length, despite its low clinical viability. Catch and anchor compounds with linker lengths of three to seven carbons were designed (**80**–**84**, Scheme 2). Under this regime, **5** could be easily and selectively halogenated at position 8 using iodine and hydrogen peroxide in ethanol to obtain **57** [23,24]. Initially, S_N_2 reactions and cross-coupling reactions were attempted directly on **57**; however, yields were significantly poorer than expected. Double alkylation of 4-phenyl benzylamine with **57** occurred, and some cross-coupling reactions were hypothetically impeded by the metal binding of the PPO group. To overcome these barriers, the phenol was initially protected by TBDMS and TMS O-silyl protecting groups, which, unfortunately, proved to be too labile. Instead, intermediate **57** was O-methylated. Here, a felicitous trans-halogenation occurred during the methylation reaction, forming **58** in the presence of excess alkylating reagent. This caused a dramatic acceleration of the subsequent S_N_2 reaction compared to the corresponding chloride intermediate, while Boc protection yielded the fully protected 8-iodofunctionalized **59**. As an added advantage, the presence of the Boc and methoxy protecting groups drastically eased purification.

Scaffolds with various linker lengths (**60**–**64**) were then accessed by Pd-catalyzed Sonogashira coupling between **59** and the corresponding alkynyl alcohols. Hydrogenation (**65**–**69**) and subsequent bromination yielded protected alkyl bromide compounds (**70**–**74**). Although Sonogashira coupling led to a high yield, the resulting compounds could not be hydrogenated. Double deprotection of **70**–**74** was achieved by treatment with BBr_3_ in anhydrous DCM in quantitative yield to furnish the penultimate alkyl bromide intermediates (**75**–**79**), which were functionalized by substitution with sodium methanethiosulfonate to yield catch and anchor compounds **80**–**84**.

### 2.4. Catch and Anchor Inhibition

**Figure 4:**
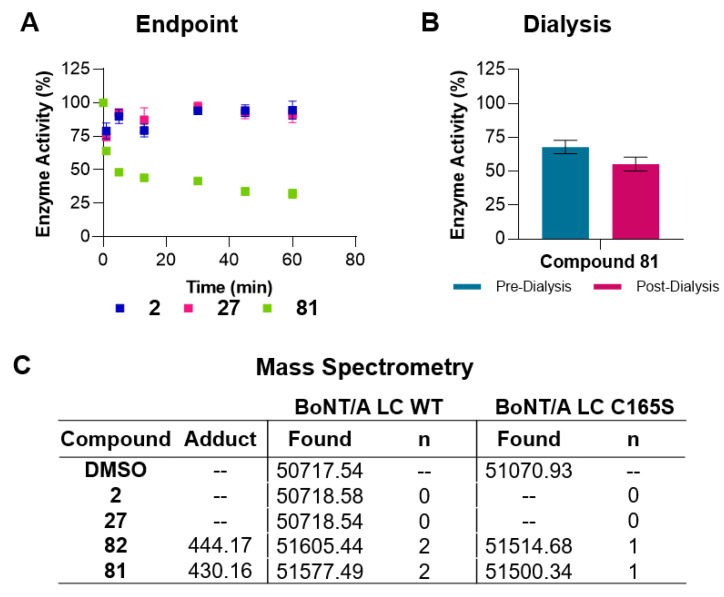
(**A**) Endpoint assay for compounds **2**, **27**, and **81** incubated at 5 µM against BoNT/A LC (10 nM). Data points normalized to a DMSO control and represent the mean activity ± SD, *n* = 3. (**B**) Effect of dialysis on enzyme activity of BoNT/A LC (500 nM) incubated with compound **81** (5 µM). Data points normalized to a DMSO control and represent the mean ± SD, *n* = 3. (**C**) Covalent adduct formation by compounds **2**, **27**, **81**, and **82** (100 µM) incubated with BoNT/A LC WT or C165S variants (40 µM) at 37 °C for 30 min.

This new series of catch and anchor compounds was first screened in the continuous SNAPtide enzyme assay, presenting time-dependent inhibition (Figure S2). MTS molecules have been shown to covalently inhibit BoNT/A LC [14,22], but it was nevertheless necessary to validate irreversible inhibition. PPO **2** and parent **27** were included as reversible controls in all experiments. To assess long-term time-dependent inhibition, an endpoint assay was performed with **81**, which was found to inhibit BoNT/A LC in a time-dependent manner (Figure 4A).

In order to rule out slow-binding kinetics as a cause of time-dependent inhibition, a dialysis experiment was conducted. BoNT/A LC was incubated with 5 µM of **81** for 30 min, and its enzyme activity was measured before and after exhaustive dialysis (Figure 4B). Enzyme incubated with **81** only showed ~60% of enzyme activity compared to the DMSO control, which was not recovered following dialysis. Since the enzyme samples were exhaustively dialyzed over 18 h, it can be concluded that the observed time-dependent inhibition is not a result of slow binding, but rather of covalent modification. Finally, covalent adduct formation was probed using HRMS. As in previous reports, the BoNT/A LC C165S variant was included as a method to differentiate the two cysteines present on BoNT/A LC [14,22]. As expected, only BoNT/A LC WT that had been incubated with either **81** or **82** showed an increase in mass matching two covalent adducts, which corresponded to adduct formation at both the active-site proximal Cys165 and distal Cys134 of the WT. Neither of the reversible compounds **2** and **27** showed any change in mass. When the C165S variant was incubated with either **81** or **82**, a mass increase corresponding to the addition of one adduct was observed. Because Ser165 of the C165S variant is unreactive to methanethiosulfonate, it can be concluded that the compounds indeed label BoNT/A LC WT at Cys165.

Following the validation of covalent irreversible inhibition, the *k*_inact_/*K*_i_ values of these compounds were obtained using the previously described continuous SNAPtide assay and are reported in (Table 2) [22]. A clear trend could be seen between linker length and *k*_inact_/*K*_i_, which indicated that a linker of four carbons (**81**) would be ideal. This compound had a *k*_inact_/*K*_i_ value that is four-fold higher than the analogous proof-of-concept compound that utilizes the 2,4-dichlorocinammic hydroxamate (DCHA) scaffold (*k*_inact_/*K*_i_ = 514 ± 17 M^−1^s^−1^) [14]. The effect of linker length and warhead proximity to Cys165 is extremely informative within this series of compounds; there is more than a two-fold difference between compounds **81** and **82**, and seven-fold between **81** and **80**, with only one carbon difference in linker length. It is likely that the difference in potency between **81** and **80** versus **81** and **82** is due to the linker length being too constrained; thus, the needed bridge between zinc ion and Cys165 is ultimately compromised. Conversely, the channel that the linker resides in may accommodate additional length. To compare linker lengths between the PPO series and the previously published DCHA series, the energy-minimized conformation of compound **81** was obtained in Chem3D. The length from the chelating group to the reactive moiety was measured to be approximately 6.9 Å, which is slightly shorter than the 7.8 Å measured in the X-ray crystal structure of the optimal chain length obtained in the proof-of-concept study. Furthermore, the distance between the reactive cysteine and C8 was measured to be 6.3 Å, close to the predicted distance of 6.1 Å obtained from docking (Figure 3A). Although Chem3D provides the minimum energy in the absence of the enzyme structure, the similarity between actual, predicted, and optimal linker lengths underlines the importance of obtaining structural information. An overall comparison of the inhibitory constants shows that *k*_inact_ remained in the same range and that the difference in potency stemmed from differences in *K*_i_. As we have noted, *vide supra,* this series of inhibitors are functionalized with an extremely reactive MTS group, which, unlike less reactive warheads, does not necessarily require active site targeting to be effective. In fact, as exemplified by the mass spectrometry experiments (Figure 4C), the MTS group react with any available cysteine. Thus, we posit that the potency of these compounds is governed by their affinity for the enzyme (*K*_i_) active site rather than their rate of enzyme inactivation (*k*_inact_). This improvement and emphasis on *K*_i_ will allow the incorporation of less reactive warheads without compromising on potency; such efforts are in progress to test this hypothesis.

## 3. Experimental

### 3.1. Assays

#### 3.1.1. General

All biochemical analyses and cell assays were performed as previously reported by Lin et al. [22] and Turner, Nielsen et al. [14] with the following modifications.

#### 3.1.2. SNAPtide Enzyme Activity Assay

The assay was conducted as previously described [22]. The final assay concentrations are as follows: SNAPtide substrate #523 = 4 µM; BoNT/A LC = 10 nM; assay buffer = 40 mM HEPES pH 7.4, 0.01% Triton X-100, 1% DMSO.

#### 3.1.3. Screening

Initial compound screening was performed at room temperature with SNAPtide substrate #521 (List Labs, Campbell, CA, USA) at a compound concentration of 40 µM.

#### 3.1.4. Endpoint Assay

In total, 500 nM BoNT/A LC was incubated with 5 µM of each compound for 30 min at 37 °C. At the indicated timepoints, 2 µL aliquots were quenched in 48 µL of SNAPtide #523 substrate (25-fold dilution) to give a SNAPtide assay final concentration of 20 nM BoNT/A LC and 0.2 µM compound.

#### 3.1.5. Dialysis Assay

Inhibitors were incubated at a concentration of 5 μM 500 nM BoNT/A LC for 30 min at 37 °C in assay buffer (40 mM HEPES, 0.01% Triton X-100, pH 7.4), 1% DMSO. Control samples containing no inhibitors were also prepared. Enzyme activity was evaluated before and after dialysis in the SNAPtide assay. Data analysis proceeded as previously described.

#### 3.1.6. Hi-resolution Mass Spectrometry of Covalent Adducts

Inhibitors were incubated with BoNT/A LC WT for 30 min at 37 °C at a concentration of 100 µM inhibitor, 40 µM enzyme in assay buffer (40 mM HEPES, 0.01% Triton X-100, pH 7.4), and 1% DMSO. Control samples containing only enzymes in assay buffer were also analyzed. Putative masses of the covalent adducts were determined using ChemDraw 19. HRMS analysis of proteins was carried out as described previously. Expected protein masses were determined using the published construct sequences and ProtParam https://web.expasy.org/protparam/ (accessed on 18 June 2020), and subtracting the first five fragmented residues (MGSSH).

#### 3.1.7. Interference Compound Filter

All final compounds were examined using publicly accessible PAINS filters: ZINC patterns search http://zinc15.docking.org/patterns/home/ (accessed on 13 March 2020); Shoichet’s aggregation advisor https://advisor.bkslab.org/ (accessed on 13 March 2020) [27]. No PAINS compounds were identified. 

#### 3.1.8. hiPSC Motor Neuron Cell Assay

BoNT/A cell activity assays were performed as previously reported. Human-induced pluripotent stem cell (hiPSC)-derived GABA neurons and culture medium (Fujifilm Cellular Dynamics International (Madison, WI, USA) were cultured in 0.01% poly-L-ornithine (Sigma Aldrich, Burlington, MA, USA) and 8.3 μg/cm^2^ matrigel, growth factor reduced, (BD Biosciences) coated 96-well TPP plates (Midsci) for 12 days prior to the assay. 200 LD50 Units of BoNT/A1 (150 kDa purified as described previously,15 specific activity (1.7 × 108 U/mg) was added to the cells in 50 μL stimulation medium (modified neurobasal containing 2.2 mM CaCl_2_ and 56 mM KCl and supplemented with 1 × B27 and 1 × GlutaMAX (100×-stocks, Life Technologies, Carlsbad, CA, USA), and the cells were incubated at 37 °C in a humidified 5% CO_2_ atmosphere for 7.5 min. Toxin was removed, cells were washed twice in 300 μL of culture medium, and further incubated in fresh culture medium at 37 °C in a humidified 5% CO_2_ atmosphere. At 30 min post first toxin addition, the inhibitors were added at the indicated concentrations and with a final DMSO concentration of 1%. Positive control (+C) was toxin without inhibitor in culture media, and negative control (-C) was culture media, both with 1% DMSO added. Cells were incubated for 7.5 h post toxin addition at 37 °C, 5% CO_2_ to allow for SNAP-25 cleavage. Inhibitor mixtures were then aspirated, and cells lysed in 50 μL of 1 × LDS lysis buffer (Invitrogen, Waltham, MA, USA). Cell lysates were analyzed by Western blot using a monoclonal anti-SNAP-25 antibody (Synaptic Systems, Göttingen, Germany; cat. #111011) as described previously, and bands were visualized using Phosphaglo chemiluminescent reagent (KPL) on an Azure C600 imaging system equipped with a CCD camera (Azure Biosystems). Assays were performed in triplicate.

#### 3.1.9. Molecular Modeling

Molecular modeling was performed with modules from the Schrödinger Small Molecule Drug Discovery Suite (Maestro), release 2018–3, using the OPLS3 force field for parameterization. The X-ray co-crystal structure of BoNT/LC (PDB 4HEV) was imported from the protein data bank and prepared using the Protein Preparation Wizard using default settings. Compounds were prepared in LigPrep, and docked using the XP Glide docking module (using metal-binding constraint) [28]. The lowest energy binding mode of compound **22** is visualized in Figure 3, and the image was generated using the PyMol Molecular Graphics System (version 2.3.4., Schrödinger, LLC., New York, NY, USA).

### 3.2. Synthesis

#### 3.2.1. General Procedures and Instrumentation

Reactions were carried out under atmospheric conditions, unless otherwise stated. All reagents were obtained from Sigma, Combi-blocks, or Fisher and used without further purification. Anhydrous solvents were distilled and stored over 4 Å molecular sieves. The reactions were monitored using thin-layer chromatography (TLC) or high-performance liquid chromatography–MS (HPLC–MS). TLC was performed using Merck precoated analytical plates (0.25 mM thick silica gel 60 F254). HPLC–MS analysis was performed on an Agilent 1260 Infinity II instrument coupled to a single quadrupole InfinityLab LC/MSD instrument running a gradient of eluent I (0.1% formic acid in H_2_O) and eluent II (0.1% formic acid in MeCN) rising linearly from 0 to 95% of II during t = 0.00–6.00 min and then with eluent II from t = 6.00–10.0 min, at a flow rate of 0.5 mL/min on a Zorbax 300SB-C8 column at 35 °C. Flash automated column chromatography (ACC) was performed using a CombiFlash Rf+ Lumen (Teledyne Isco) purification system with flash silica RediSep Rf columns for normal phase (NP), amine-functionalized silica RediSep Rf columns for normal phase, or RediSep Rf Gold C18 HP columns for reversed phase (RP). The purity of all tested compounds is >95%, as determined by HPLC–MS.

Nuclear magnetic resonance (NMR) spectra were recorded on either a Bruker AVIII HD 600 NMR equipped with a 5 mM CPQCl CryoProbe/5 mM CPDCH CryoProbe or a Bruker AV NEO at 400, 500, or 600 MHz for ^1^H NMR and 101, 126, or 151 MHz for ^13^C NMR. Chemical shifts are reported in ppm and are reported with reference to the residual solvent peak (δH CDCl_3_ 7.26 ppm; δC CDCl_3_ 77.16 ppm; δH DMSO-d_6_ 2.50 ppm; δC DMSO-d_6_ 39.52 ppm). Multiplicities are reported with coupling constants and are given to the nearest 0.1 Hz. Spectra of final compounds are provided in the Supplementary Materials. HRMS was carried out using an Agilent 1260 Infinity II instrument coupled to an Agilent 6230 TOF-MS spectrometer using electrospray ionization (ES+), giving masses correct to four decimal places.

#### 3.2.2. Synthesis and Characterization of PPO Hits

##### 2-(Chloromethyl)-9-hydroxy-4*H*-pyrido [1,2-a]pyrimidin-4-one (**5**)

2-aminopyridin-3-ol (5.5 g, 50 mmol, 1 equiv.) and ethyl 4-chloroacetoacetate (52.5 g, 52.5 mmol, 1.05 equiv.) were mixed with polyphosphoric acid (approx. 30 g) and heated to 110 °C for 4 h. After heating, the reaction was diluted with 100 mL ice water and adjusted to pH 2 using 2 M NaOH (aq.). The resulting brown precipitate was filtered and washed lightly with 1 M HCl. The crude precipitate was purified by ACC (0–5% MeOH in DCM) to give the title compound a light yellow solid (7.030 g, 33.473 mmol, 66.7%). ^1^H NMR (600 MHz, DMSO-d_6_) δ 10.49 (s, 1H), 8.50 (dd, *J* = 7.0, 1.4 Hz, 1H), 7.30 (dd, *J* = 7.6, 1.5 Hz, 1H), 7.25 (t, *J* = 7.3 Hz, 1H), 6.55 (s, 1H), 4.69 (s, 2H). ^13^C NMR (151 MHz, DMSO-d_6_) δ 160.5, 157.4, 150.2, 144.8, 117.5, 116.6, 116.2, 101.8, 45.9. HRMS (ES+) *m*/*z* calc. for C_9_H_7_ClN_2_O_2_ [M + H]^+^: 211.0269; found: 211.0271.

##### 2-((Benzylamino)methyl)-9-hydroxy-4*H*-pyrido [1,2-a]pyrimidin-4-one (**2**)

Compound **5** (210 mg, 1.0 mmol, 1.0 equiv.) was dissolved in 3 mL DMF. Benzylamine (327 µL, 3.0 mmol, 3.0 equiv.) was added, and the reaction was stirred overnight at room temperature. Upon completion, the reaction was filtered to obtain the product. An additional product was obtained as the HCl salt by addition of 1 mL conc. HCl and filtration of the resulting precipitate. The title compound was collected as a beige solid (49 mg, 0.174 mmol 16.0%). ^1^H NMR (600 MHz, DMSO-d_6_) δ 8.48 (dd, *J* = 7.0, 1.3 Hz, 1H), 7.62–7.55 (m, 2H), 7.43–7.36 (m, 4H), 7.31 (t, *J* = 7.6, 7.1 Hz, 1H), 6.44 (s, 1H), 4.23 (s, 2H), 4.22 (s, 2H). ^13^C NMR (151 MHz, DMSO-d_6_) δ 157.3, 156.1, 150.3, 144.6, 132.0, 130.9, 129.5, 129.1, 118.0, 117.6, 116.6, 101.8, 50.2, 49.0. HRMS (ES+) *m*/*z* calc. for C_16_H_15_N_3_O_2_ [M + H]⁺: 282.1238; found: 282.125.

##### 2-Ethyl-9-hydroxy-4*H*-pyrido [1,2-a]pyrimidin-4-one (**6**)

Synthesized from 2-aminopyridin-3-ol (220 mg, 2 mmol, 1.0 equiv.) and ethyl propionyl acetate (314 µL, 2.2 mmol, 1.1 equiv.) in polyphosphoric acid (approx. 2 g) in the same manner as **5**, with no purification necessary. The resulting precipitate was dried under hi-vac to yield the title compound as a beige solid (280 mg, 1.473 mmol, 74%). ^1^H NMR (600 MHz, DMSO-d_6_) δ 8.43 (dd, *J* = 7.0, 1.5 Hz, 1H), 7.21 (dd, *J* = 7.5, 1.5 Hz, 1H), 7.15 (t, *J* = 7.3 Hz, 1H), 6.28 (s, 1H), 2.66 (q, *J* = 7.6 Hz, 2H), 1.25 (t, *J* = 7.6 Hz, 3H). ^13^C NMR (151 MHz, DMSO-d_6_) δ 167.6, 157.3, 149.6, 144.5, 117.2, 115.7, 115.0, 100.9, 30.4, 12.7. HRMS (ES+) *m*/*z* calc. for C_10_H_10_N_2_O_2_ [M + H]⁺: 191.0816; found: 191.0819.

##### 9-Hydroxy-2-(3-phenylpropyl)-4H-pyrido [1,2-a]pyrimidin-4-one (**7**)

Synthesized from 2-aminopyridin-3-ol (182 mg g, 1.65 mmol, 1.1 equiv.) and ethyl 3-oxo-6-phenylhexanoate (351 mg, 1.5 mmol, 1.0 equiv.) in polyphosphoric acid (approx. 2 g) in the same manner as 5, and purified using normal-phase ACC (0–5% MeOH in DCM). The title compound was obtained as a clear residue (105 mg, 0.374 mmol, 25%). ^1^H NMR (600 MHz, DMSO-d_6_) δ 8.46–8.43 (m, 1H), 7.48 (td, *J* = 7.9, 2.3 Hz, 1H), 7.30–7.24 (m, 3H), 7.18–7.14 (m, 3H), 6.29 (s, 1H), 2.64 (dtd, *J* = 17.8, 10.0, 9.2, 4.9 Hz, 4H), 2.09–2.01 (m, 2H). ^13^C NMR (151 MHz, DMSO-d_6_) δ 157.2, 149.6, 144.5, 140.3, 133.0, 131.9, 128.5, 128.1, 117.3, 115.7, 115.0, 101.8, 36.9, 34.8, 29.5. HRMS (ES+) *m*/*z* calc. for C_17_H_16_N_2_O_2_ [M + H]⁺: 281.1285; found: 281.1292.

##### 2-(Chloromethyl)-4*H*-pyrido [1,2-a]pyrimidin-4-one (**8**)

2-aminopyridine (471 mg, 5.0 mmol, 1.0 equiv.) and ethyl 4-chloroacetoacetate (1.0 mL, 7.5 mmol, 1.5 equiv.) were mixed with polyphosphoric acid (approx. 7.5 g) and heated to 110 °C. After 3.5 h, the reaction mixture was cooled, diluted with 30 mL ice water, and adjusted to pH 2 using 2 M NaOH (aq.). This aqueous solution was extracted with DCM (6 × 20 mL), and the combined organic extracts dried over Na_2_SO_4_ to yield 450 mg (2.31 mmol, 46.2%) of the title compound as a beige solid. ^1^H NMR (600 MHz, DMSO-d^6^) δ 8.96 (ddd, *J* = 7.1, 1.5, 0.8 Hz, 1H), 8.00 (ddd, *J* = 8.6, 6.7, 1.6 Hz, 1H), 7.71 (dt, *J* = 8.9, 1.1 Hz, 1H), 7.38 (td, *J* = 6.9, 1.4 Hz, 1H), 6.56 (s, 1H), 4.67 (s, 2H). ^13^C NMR (151 MHz, DMSO-d_6_) δ 162.1, 150.8, 138.1, 127.1, 125.8, 116.6, 101.8, 45.8.

##### 2-(Chloromethyl)-9-ethoxy-4*H*-pyrido [1,2-a]pyrimidin-4-one (**9**)

Synthesized from ethyl chloroacetoacetate and 3-ethoxypyridin-2-amine in the same manner as 5. The title compound was obtained as a light brown solid (288 mg, 1.21 mmol, 60.4%). ^1^H NMR (600 MHz, DMSO-d_6_) δ 8.55 (dd, *J* = 7.1, 1.3 Hz, 1H), 7.39 (dd, *J* = 7.8, 1.3 Hz, 1H), 7.27 (t, *J* = 7.4 Hz, 1H), 6.57 (s, 1H), 4.69 (s, 2H), 4.23 (q, *J* = 7.0 Hz, 2H), 1.43 (t, *J* = 7.0 Hz, 3H). ^13^C NMR (151 MHz, DMSO-d_6_) δ 160.9, 157.4, 150.9, 145.0, 118.2, 115.8, 113.9, 102.4, 102.4, 65.0, 45.9, 14.3.

##### 2-(((4-Chlorobenzyl)amino)methyl)-4*H*-pyrido [1,2-a]pyrimidin-4-one (**10**)

4-chlorobenzylamine (243 µL, 2.0 mmol, 1.0 equiv.), K_2_CO_3_ (414 mg, 3.0 mmol, 3.0 equiv.), and 8 (194 mg, 1.0 mmol,1 equiv.) were suspended in 10 mL DMF and heated to 70 °C. After 3.5 h the reaction was removed from heat and diluted with 20 mL H_2_O and 20 mL Et_2_O. The aqueous layer was separated and extracted with Et_2_O (3 × 20 mL). The combined organic layers were dried over Na_2_SO_4_ and concentrated. The crude product was purified using normal-phase ACC (0–100% EtOAc in hexanes) to afford 45 mg (0.3 mmol, 30.0%) of white solid as the title compound.

^1^H NMR (600 MHz, DMSO-d_6_) δ 8.93 (dt, *J* = 7.0, 1.2 Hz, 1H), 7.93 (ddd, *J* = 8.5, 6.7, 1.6 Hz, 1H), 7.62 (dt, *J* = 8.9, 1.1 Hz, 1H), 7.41–7.27 (m, 6H), 6.52 (s, 1H), 3.74 (s, 2H), 3.68 (s, 2H), 2.89 (s, 1H). ^13^C NMR (151 MHz, DMSO-d_6_) δ 166.9, 157.3, 150.5, 139.7, 137.3, 131.1, 129.8, 128.0, 126.9, 125.5, 115.9, 100.2, 53.1, 51.4. HRMS (ES+) *m*/*z* calc. for C_16_H_14_ClN_3_O_2_ [M + H]⁺: 300.0899; found: 300.0907

##### 2-((Benzylamino)methyl)-9-ethoxy-4*H*-pyrido [1,2-a]pyrimidin-4-one (**11**)

Synthesized from **9** (119 mg, 0.5 mmol, 1.0 equiv.) and benzylamine (109 µL, 1.0 mmol, 1.0 equiv.) in the same manner as 2. The title compound was obtained as an off-white solid (50 mg, 0.162 mmol, 32.2%). ^1^H NMR (600 MHz, DMSO-d_6_) δ 8.53 (d, *J* = 7.1 Hz, 1H), 7.39 (d, *J* = 7.5 Hz, 2H), 7.33 (t, *J* = 7.5 Hz, 3H), 7.22 (dt, *J* = 22.2, 7.3 Hz, 2H), 6.57 (s, 1H), 4.20 (q, *J* = 6.9 Hz, 2H), 3.79 (s, 2H), 3.72 (s, 2H), 1.42 (t, *J* = 6.8 Hz, 3H). ^13^C NMR (151 MHz, DMSO-d_6_) δ 165.4, 157.4, 150.7, 144.7, 140.2, 128.1, 128.1, 126.7, 118.1, 115.0, 113.2, 100.9, 64.9, 53.0, 52.2, 14.3. NMR contains formic acid. HRMS (ES+) *m*/*z* calc. for C_18_H_19_N_3_O_2_ [M + H]⁺: 310.1550; found: 310.1558.

##### 2-(Azidomethyl)-9-hydroxy-4*H*-pyrido [1,2-a]pyrimidin-4-one (**12**)

**5** (210 mg, 1.0 mmol, 1.0 equiv.) and sodium azide (325 mg, 5.0 mmol, 5 equiv.) were stirred in 2 mL DMF at room temperature. After 2 h, the rection mixture was diluted with 25 mL EtOAc and washed with water (2 × 25 mL) then brine (25 mL). The organic extract was dried over Na_2_SO_4_ and the title compound was obtained as a light tan solid (180 mg, 0.829 mmol, 82.9%).

^1^H NMR (600 MHz, CDCl_3_) δ 8.57 (dd, *J* = 7.2, 1.3 Hz, 1H), 7.26 (dd, *J* = 7.6, 1.4 Hz, 1H), 7.12 (t, *J* = 7.3 Hz, 1H), 6.49 (s, 1H), 4.38 (s, 2H). ^13^C NMR (151 MHz, CDCl_3_) δ 160.0, 157.9, 148.7, 144.6, 118.4, 116.2, 114.7, 102.3, 54.3. The NMR spectra contain trace DMF.

##### 2-(Aminomethyl)-9-hydroxy-4*H*-pyrido [1,2-a]pyrimidin-4-one (**13**)

**12** (180 mg, 0.829 mmol, 1.0 equiv.) and triphenyl phosphine (261 mg, 0.995 mmol, 1.2 equiv.) were dissolved in 2 mL H_2_O and 2 mL THF. The reaction mixture was heated to 60 °C for 4 h. After completion, the reaction mixture was cooled and concentrated. The residue was triturated in MeOH, filtered, and washed with MeOH to yield 43 mg of brown solid as the title compound (0.225 mmol, 27.2%). ^1^H NMR (600 MHz, DMSO-d_6_) δ 8.51 (dd, *J* = 7.1, 1.3 Hz, 1H), 7.46 (d, *J* = 7.3 Hz, 1H), 7.32 (t, *J* = 7.4 Hz, 2H), 6.34 (s, 1H), 4.36 (s, 2H). ^13^C NMR (151 MHz, DMSO-d_6_) δ 157.3, 156.8, 149.8, 144.0, 117.7, 117.1, 116.3, 100.3, 41.7. HRMS (ES+) *m*/*z* calc. for C_9_H_9_N_3_O_2_ [M + H]⁺: 192.0768; found: 192.077.

##### 2-((Benzylamino)methyl)quinolin-8-ol (**14**)

In an oven-dried flask under argon, 8-hydroxylquinoline-2-carboxaldehyde (173 mg, 1.0 mmol, 1.0 equiv.) and benzylamine (120 µL, 1.2 mmol, 1.2 equiv.) were dissolved in 5 mL anhydrous dichloroethane and cooled to 0 °C in an ice bath. Sodium triacetoxyborohydride (317 mg, 1.5 mmol, 1.0 equiv.) was added in three portions, flushing the vessel with argon after each addition. The reaction was stirred for 5 min in the ice bath before the bath was removed and the reaction allowed to warm to room temperature. After 24 h, 10 mL H_2_O was added slowly to quench the reaction. The reaction mixture was extracted with 20 mL DCM, and the organic extract was dried over Na_2_SO4 and concentrated. The crude product was purified using normal-phase ACC on amine-functionalized silica (0–50% EtOAc in hexanes) to yield 222 mg (0.84 mmol, 84.1%) yellow solid as the title compound. ^1^H NMR (600 MHz, CDCl_3_) δ 8.10 (d, *J* = 8.4 Hz, 1H), 7.46 (d, *J* = 8.4 Hz, 1H), 7.42 (t, *J* = 7.9 Hz, 1H), 7.39–7.33 (m, 4H), 7.31 (dd, *J* = 8.2, 1.2 Hz, 1H), 7.29–7.26 (m, 1H), 7.18 (dd, *J* = 7.6, 1.2 Hz, 1H), 4.12 (s, 2H), 3.90 (s, 2H). ^13^C NMR (151 MHz, CDCl_3_) δ 157.6, 151.5, 139.4, 137.1, 136.1, 128.0, 127.8, 127.0, 126.7, 126.7, 120.9, 117.3, 109.8, 54.0, 53.0. HRMS (ES+) *m*/*z* calc. for C_17_H_16_N_2_O [M + H]⁺: 265.1335; found: 265.1344.

##### 2-((Dimethylamino)methyl)-9-hydroxy-4*H*-pyrido [1,2-a]pyrimidin-4-one (**15**)

**5** (105 mg, 0.5 mmol, 1.0 equiv.) was dissolved in 1 mL DMF and a solution of 2 M dimethylbenzyl amine in THF (750 µL, 1.5 mmol, 3.0 equiv.) was added. The reaction mixture was stirred at room temperature overnight, then filtered. The filtrate was concentrated and purified using normal-phase ACC on an amine-functionalized silica column (0–10% MeOH in DCM). The title compound was obtained as a light yellow oil (89 mg, 0.406 mmol, 81.7%). ^1^H NMR (600 MHz, DMSO-d_6_) δ 8.36 (d, *J* = 6.3 Hz, 1H), 7.15 (t, *J* = 7.3 Hz, 1H), 7.10 (d, *J* = 7.2 Hz, 1H), 6.36 (s, 1H), 3.46 (s, 2H), 2.25 (s, 6H). ^13^C NMR (151 MHz, DMSO-d_6_) δ 172.3, 163.1, 152.4, 145.5, 116.5, 115.4, 115.1, 100.6, 64.3, 34.2. HRMS (ES+) *m*/*z* calc. for C_11_H_13_N_3_O_2_ [M + H]⁺: 220.1081; found: 220.1086.

#### 3.2.3. General Synthesis of Benzylamine PPO (**16**–**29**)

Compound **5** (210 mg, 0.5 mmol, 1.0 equiv.) was dissolved in 1 mL DMF. The corresponding benzylamine (3.0 mmol, 3.0 equiv.) was added, and the reaction was stirred overnight at room temperature. Upon completion, the reaction was diluted with water and EtOAc. The organic phase was separated, dried over Na_2_SO_4_ and concentrated. The crude product was purified by RP ACC (0–100% ACN in 0.1% formic acid) and lyophilized to yield final compounds.

##### 2-(((3-Fluorobenzyl)amino)methyl)-9-hydroxy-4*H*-pyrido [1,2-a]pyrimidin-4-one (**16**)

The title compound was obtained as a fluffy white solid (88 mg, 0.294 mmol, 58.7%). ^1^H NMR (600 MHz, DMSO-d_6_) δ 8.44 (dd, *J* = 6.9, 1.4 Hz, 1H), 8.22 (s, 1H), 7.36 (td, *J* = 7.9, 6.1 Hz, 1H), 7.27–7.21 (m, 3H), 7.21–7.18 (m, 1H), 7.09–7.05 (m, 1H), 6.43 (s, 1H), 3.85 (s, 2H), 3.80 (s, 2H). ^13^C NMR (151 MHz, DMSO-d_6_) δ 163.8, 163.0, 163.0, 161.4, 157.3, 150.0, 144.5, 142.2, 142.2, 130.1, 130.0, 124.4, 124.4, 117.1, 116.2, 115.2, 115.0, 114.9, 113.8, 113.7, 100.7, 100.7, 52.1, 51.2, 51.2, 51.2, 51.2. HRMS (ES+) *m*/*z* calc. for C_16_H_14_FN_3_O_2_ [M + H]⁺: 300.1143; found: 300.1149.

##### 2-(((4-Fluorobenzyl)amino)methyl)-9-hydroxy-4*H*-pyrido [1,2-a]pyrimidin-4-one (**17**)

The title compound was obtained as an off-white solid (62 mg, 0.207 mmol, 41.3%). ^1^H NMR (600 MHz, DMSO-d_6_) δ 8.43 (dd, *J* = 6.9, 1.5 Hz, 1H), 8.25 (s, 1H), 7.45–7.39 (m, 2H), 7.24 (dd, *J* = 7.5, 1.5 Hz, 1H), 7.21–7.18 (m, 1H), 7.18–7.11 (m, 2H), 6.42 (s, 1H), 3.83 (s, 2H), 3.80 (s, 2H). ^13^C NMR (151 MHz, DMSO-d_6_) δ 164.1, 162.2, 160.6, 157.3, 144.6, 134.9, 130.46 (d, *J* = 8.2 Hz), 116.3, 115.2, 115.0, 114.8, 100.6, 51.9, 50.9. HRMS (ES+) *m*/*z* calc. for C_16_H_14_FN_3_O_2_ [M + H]⁺: 300.1143; found: 300.1135.

##### 2-(((3-Chlorobenzyl)amino)methyl)-9-hydroxy-4*H*-pyrido [1,2-a]pyrimidin-4-one (**18**)

The title compound was obtained as a yellow solid (93 mg, 0.295 mmol, 59.2%). ^1^H NMR (400 MHz, DMSO-d_6_) δ 8.38 (dd, *J* = 6.7, 1.5 Hz, 1H), 7.47–7.41 (m, 1H), 7.38–7.24 (m, 3H), 7.20 (dd, *J* = 7.7, 6.8 Hz, 1H), 7.15 (dd, *J* = 7.7, 1.5 Hz, 1H), 6.49 (s, 1H), 4.67 (s, 2H), 3.z (s, 2H). ^13^C NMR (151 MHz, DMSO-d_6_) δ 164.2, 160.1, 157.7, 153.0, 145.8, 139.5, 133.0, 130.2, 128.3, 127.6, 127.1, 117.2, 116.0, 101.1, 46.0, 42.5. HRMS (ES+) *m*/*z* calc. for C_16_H_14_ClN_3_O_2_ [M + H]⁺: 316.0848; found: 316.0852.

##### 2-(((4-Chlorobenzyl)amino)methyl)-9-hydroxy-4*H*-pyrido [1,2-a]pyrimidin-4-one (**19**)

The product was obtained as a yellow solid (55 mg, 0.175 mg, 17.5%). ^1^H NMR (600 MHz, DMSO-d_6_) δ 8.49 (dd, *J* = 7.0, 1.3 Hz, 1H), 7.62–7.58 (m, 2H), 7.51–7.47 (m, 2H), 7.38 (dd, *J* = 7.6, 1.3 Hz, 1H), 7.32 (dd, *J* = 7.7, 7.1 Hz, 1H), 6.45 (s, 1H), 4.24 (apparent s, 4H). ^13^C NMR (151 MHz, DMSO-d_6_) δ 156.9, 155.6, 149.8, 144.2, 133.9, 132.4, 130.6, 128.6, 117.6, 117.1, 116.2, 101.4, 48.9, 48.5. HRMS (ES+) *m*/*z* calc. for C_16_H_14_N_3_O_2_ [M + H]⁺: 282.1238; found: 282.1250.

##### 9-Hydroxy-2-(((3-methoxybenzyl)amino)methyl)-4*H*-pyrido [1,2-a]pyrimidin-4-one (**20**)

Title compound was obtained as a light yellow solid (120 mg, 0.386 mmol, 77.4%). ^1^H NMR (600 MHz, DMSO-d_6_) δ 8.43 (dd, *J* = 6.8, 1.5 Hz, 1H), 8.25 (s, 1H), 7.26–7.19 (m, 3H), 7.02–6.99 (m, 1H), 6.96 (dt, *J* = 7.6, 1.2 Hz, 1H), 6.82 (ddd, *J* = 8.2, 2.7, 0.9 Hz, 1H), 6.41 (s, 1H), 3.87 (s, 2H), 3.85 (s, 2H), 3.73 (s, 3H). ^13^C NMR (151 MHz, DMSO-d_6_) δ 164.2, 161.9, 159.3, 157.3, 150.4, 144.6, 139.4, 129.3, 120.9, 117.0, 116.4, 115.3, 114.1, 112.9, 100.7, 55.0, 51.5. HRMS (ES+) *m*/*z* calc. for C_17_H_17_N_3_O_3_ [M + H]⁺: 312.1343; found: 312.1352.

##### 9-Hydroxy-2-(((4-methoxybenzyl)amino)methyl)-4*H*-pyrido [1,2-a]pyrimidin-4-one (**21**)

Title compound was obtained as a beige solid (10 mg, 0.032 mmol, 3.2%). ^1^H NMR (600 MHz, DMSO-d_6_) δ 8.47 (dd, *J* = 7.0, 1.4 Hz, 1H), 7.49 (dd, *J* = 7.2, 0.3 Hz, 2H), 7.36 (dd, *J* = 7.6, 1.3 Hz, 1H), 7.32–7.27 (m, 1H), 6.95–6.91 (m, 2H), 6.42 (s, 1H), 4.13 (d, *J* = 8.0 Hz, 4H), 3.74 (s, 3H). ^13^C NMR (151 MHz, DMSO-d_6_) δ 159.6, 156.9, 149.9, 144.2, 131.9, 130.5, 117.5, 117.0, 116.0, 113.9, 113.9, 101.3, 55.2, 49.3, 48.4. HRMS (ES+) *m*/*z* calc. for C_17_H_17_N_3_O_3_ [M + H]⁺: 312.1343; found: 312.1339.

##### 9-Hydroxy-2-(((3-methylbenzyl)amino)methyl)-4*H*-pyrido [1,2-a]pyrimidin-4-one (**22**)

Title compound was obtained as a fluffy cream solid (74 mg, 0.251 mmol, 50.3%). ^1^H NMR (600 MHz, DMSO-d_6_) δ 8.42 (dd, *J* = 6.9, 1.6 Hz, 1H), 8.26 (s, 1H), 7.26–7.16 (m, 5H), 7.06 (dt, *J* = 7.2, 2.0 Hz, 1H), 6.39 (s, 1H), 3.84 (s, 2H), 3.84 (s, 2H), 2.26 (s, 3H). ^13^C NMR (151 MHz, DMSO-d_6_) δ 164.3, 161.8, 157.3, 150.6, 144.7, 137.7, 137.4, 129.5, 128.2, 128.0, 125.9, 116.8, 116.5, 115.4, 100.7, 51.6, 51.5, 21.0. HRMS (ES+) *m*/*z* calc. for C_17_H_17_N_3_O_2_ [M + H]⁺: 296.1394; found: 296.1399.

##### 9-Hydroxy-2-(((4-methylbenzyl)amino)methyl)-4*H*-pyrido [1,2-a]pyrimidin-4-one (**23**)

Title compound was obtained as a light yellow solid (26 mg, 0.088 mmol, 13.1%). ^1^H NMR (600 MHz, DMSO-d_6_) δ 8.42 (d, *J* = 6.2 Hz, 1H), 8.21 (s, 1H), 7.26 (d, *J* = 7.7 Hz, 2H), 7.24–7.17 (m, 2H), 7.13 (d, *J* = 7.7 Hz, 2H), 6.41 (s, 1H), 3.76 (s, 2H), 3.74 (s, 2H), 2.27 (s, 3H). ^13^C NMR (151 MHz, DMSO-d_6_) δ 163.7, 163.4, 157.3, 136.3, 135.9, 129.1, 128.7, 128.3, 117.1, 116.2, 115.1, 100.6, 52.2, 51.7, 20.7. HRMS (ES+) *m*/*z* calc. for C_17_H_17_N_3_O_2_ [M + H]⁺: 296.1394; found: 296.14.

##### Methyl 3-((((9-hydroxy-4-oxo-4*H*-pyrido [1,2-a]pyrimidin-2-yl)methyl)amino)methyl) benzoate (**24**)

The title compound was obtained as a fluffy white solid (93 mg, 0.274 mmol, 54.7%). ^1^H NMR (600 MHz, DMSO-d_6_) δ 8.43 (dd, *J* = 7.0, 1.4 Hz, 1H), 8.20 (s, 1H), 8.01 (t, *J* = 1.8 Hz, 1H), 7.83 (dt, *J* = 7.7, 1.5 Hz, 1H), 7.65 (dt, *J* = 7.6, 1.5 Hz, 1H), 7.47 (t, *J* = 7.7 Hz, 1H), 7.24 (dd, *J* = 7.7, 1.5 Hz, 1H), 7.19 (t, *J* = 7.3 Hz, 1H), 6.41 (s, 1H), 3.88 (s, 2H), 3.84 (s, 3H), 3.78 (s, 2H). ^13^C NMR (151 MHz, DMSO-d_6_) δ 166.3, 163.7, 163.3, 157.3, 150.0, 144.5, 140.2, 133.3, 129.5, 129.0, 128.6, 127.8, 117.1, 116.2, 115.1, 100.6, 52.3, 52.1, 51.5. HRMS (ES+) *m*/*z* calc. for C_18_H_17_N_3_O_4_ [M + H]⁺: 340.1292; found: 340.1293.

##### Methyl 4-((((9-hydroxy-4-oxo-4*H*-pyrido [1,2-a]pyrimidin-2-yl)methyl)amino)methyl) benzoate (**25**)

The title compound was obtained as a white solid (44 mg, 0.13 mmol, 26%).

^1^H NMR (600 MHz, DMSO-d_6_) δ 8.41 (dd, *J* = 6.7, 1.7 Hz, 1H), 7.94–7.88 (m, 2H), 7.54–7.49 (m, 2H), 7.23–7.15 (m, 2H), 6.50 (s, 1H), 4.68 (s, 2H), 3.89 (s, 2H), 3.84 (s, 3H). ^13^C NMR (151 MHz, DMSO-d_6_) δ 185.1, 166.2, 160.1, 157.6, 147.3, 145.6, 129.1, 128.0, 127.7, 117.1, 116.0, 115.7, 101.2, 52.0, 46.0, 44.4.

HRMS (ES+) *m*/*z* calc. for C_18_H_17_N_3_O_4_ [M + H]⁺: 340.1292; found: 340.1288.

##### 2-((([1,1′-biphenyl]-3-ylmethyl)amino)methyl)-9-hydroxy-4*H*-pyrido [1,2-a]pyrimidin-4-one (**26**)

The title compound was obtained as a fluffy white solid (110 mg, 0.308 mmol, 61.8%). ^1^H NMR (600 MHz, DMSO-d_6_) δ 8.43 (dd, *J* = 6.9, 1.5 Hz, 1H), 8.24 (s, 1H), 7.72–7.69 (m, 1H), 7.65–7.62 (m, 2H), 7.55 (dt, *J* = 7.4, 1.7 Hz, 1H), 7.47–7.37 (m, 4H), 7.37–7.34 (m, 1H), 7.24 (dd, *J* = 7.6, 1.5 Hz, 1H), 7.21–7.18 (m, 1H), 6.43 (s, 1H), 3.95 (s, 2H), 3.88 (s, 2H). ^13^C NMR (151 MHz, DMSO-d_6_) δ 164.0, 162.3, 157.2, 150.4, 144.6, 140.2, 140.1, 138.9, 128.9, 128.8, 127.8, 127.5, 127.1, 126.7, 125.6, 116.9, 116.3, 115.3, 100.7, 51.8, 51.7. HRMS (ES + ) *m*/*z* calc. for C_22_H_19_N_3_O_2_ [M + H]⁺: 358.1551; found: 358.1566.

##### 2-((([1,1′-biphenyl]-4-ylmethyl)amino)methyl)-9-hydroxy-4*H*-pyrido [1,2-a]pyrimidin-4-one (**27**)

The title compound was obtained as a white solid (50 mg, 0.14 mmol, 28.1%).

^1^H NMR (600 MHz, DMSO-d_6_) δ 8.45 (dd, *J* = 7.0, 1.3 Hz, 1H), 7.67–7.62 (m, 4H), 7.53 (d, *J* = 8.1 Hz, 2H), 7.46 (t, *J* = 7.7 Hz, 2H), 7.39–7.34 (m, 1H), 7.29 (dd, *J* = 7.6, 1.4 Hz, 1H), 7.23 (t, *J* = 7.3 Hz, 1H), 6.46 (s, 1H), 3.98 (s, 3H), 3.94 (s, 2H). ^13^C NMR (151 MHz, DMSO-d_6_) δ 163.4, 157.2, 149.9, 144.4, 139.8, 139.4, 129.6, 128.9, 127.4, 126.6, 126.6, 117.3, 116.4, 115.4, 100.9, 51.3, 51.0. NMR spectra contain formic acid. HRMS (ES+) *m*/*z* calc. for C_22_H_19_N_3_O_2_ [M + H]⁺: 358.1550; found: 358.1556.

##### 2-((([1,1′-biphenyl]-4-ylmethyl)(methyl)amino)methyl)-9-hydroxy-4*H*-pyrido [1,2-a]pyrimidin-4-one (**28**)

1-([1,1′-biphenyl]-4-yl)-N-methylmethanamine (197 mg, 1.000 mmol, 2.0 equiv.) and compound 5 (105 mg, 0.500 mmol, 1.0 equiv.) were dissolved in 5 mL DMF and heated to 60 °C. The reaction was stirred at 60 °C for two hours, then allowed to cool to room temperature before filtering. The filtrate was collected and purified by RP ACC (0–100% ACN in 0.1% formic acid) followed by normal-phase ACC (0–10% MeOH in DCM) followed to yield the title compound as a colorless solid (112 mg, 0.302 mmol, 60.2%). ^1^H NMR (600 MHz, DMSO-d_6_) δ 8.47 (dd, *J* = 7.0, 1.4 Hz, 1H), 7.67–7.60 (m, 4H), 7.49–7.42 (m, 4H), 7.37–7.31 (m, 1H), 7.23 (dd, *J* = 7.5, 1.4 Hz, 1H), 7.18 (t, *J* = 7.7 Hz, 1H), 6.58 (s, 1H), 3.66 (s, 2H), 3.62 (s, 2H), 2.25 (s, 3H). ^13^C NMR (151 MHz, DMSO-d_6_) δ 164.1, 157.5, 149.9, 144.5, 140.0, 138.9, 138.0, 129.2, 128.9, 127.3, 126.6, 117.4, 116.0, 115.4, 100.7, 61.9, 60.9, 42.2. HRMS (ES+) *m*/*z* calc. for C_23_H_21_N_3_O_2_ [M + H]⁺: 372.1707; found: 372.1700.

##### 9-Hydroxy-2-((phenethylamino)methyl)-4*H*-pyrido [1,2-a]pyrimidin-4-one (**29**)

The title compound was obtained as a beige solid (68 mg, 0.23 mmol, 56.8%). ^1^H NMR (600 MHz, DMSO-d_6_) δ 8.49 (dd, *J* = 7.1, 1.3 Hz, 1H), 7.36 (dd, *J* = 7.7, 1.3 Hz, 1H), 7.34–7.28 (m, 3H), 7.28–7.25 (m, 2H), 7.25–7.22 (m, 1H), 6.45 (s, 1H), 4.25 (s, 2H), 3.18 (t, *J* = 9.0 Hz, 2H), 3.05–3.01 (m, 2H). ^13^C NMR (151 MHz, DMSO-d_6_) δ 156.9, 149.8, 144.3, 137.6, 137.3, 128.6, 128.5, 126.6, 117.6, 116.9, 116.1, 101.0, 49.8, 48.1, 32.1. HRMS (ES+) *m*/*z* calc. for C_17_H_17_N_3_O_2_ [M + H]⁺: 296.1394; found: 296.1400.

#### 3.2.4. General Synthesis of Benzyl Ether PPO (**30**–**41**)

53 mg (0.25 mmol) of **5** was dissolved in 1 mL DMF. 3.0 equivalents of the corresponding benzyl alcohol and 1 mL of 1.25 M NaOH were added. The mixture was heated to 70 °C and stirred overnight. After cooling to room temperature, the reaction mixture was concentrated in vacuo. The crude residue was purified on ACC (0–5% MeOH in DCM), followed by RP ACC (0–100% ACN in 0.1% formic acid) to yield pure benzyl alcohol derivatives.

##### 2-((Benzyloxy)methyl)-9-hydroxy-4*H*-pyrido [1,2-a]pyrimidin-4-one (**30**)

The title compound was obtained as a light yellow solid (85 mg, 0.301 mmol, 30.1%). ^1^H NMR (600 MHz, DMSO-d_6_) δ 10.27 (s, 1H), 8.48 (dd, *J* = 7.0, 1.5 Hz, 1H), 7.44–7.37 (m, 4H), 7.34–7.30 (m, 1H), 7.24 (dd, *J* = 7.5, 1.5 Hz, 1H), 7.19 (t, *J* = 7.3 Hz, 1H), 4.67 (s, 2H), 4.56 (d, *J* = 1.0 Hz, 2H). ^13^C NMR (151 MHz, DMSO-d_6_) δ 163.1, 157.4, 149.9, 144.7, 138.0, 128.4, 127.7, 127.6, 117.5, 116.0, 115.6, 99.4, 72.1, 71.2. HRMS (ES+) *m*/*z* calc. for C_16_H_14_N_2_O_3_ [M + H]⁺: 283.1078; found: 283.1076.

##### 2-(((2-Fluorobenzyl)oxy)methyl)-9-hydroxy-4*H*-pyrido [1,2-a]pyrimidin-4-one (**31**)

The title compound was obtained as a white solid (21 mg, 0.070 mmol, 28%). ^1^H NMR (600 MHz, CDCl_3_) δ 8.55 (dd, *J* = 7.2, 1.3 Hz, 1H), 7.50 (td, *J* = 7.5, 1.8 Hz, 1H), 7.31 (tdd, *J* = 7.5, 5.3, 1.8 Hz, 1H), 7.17 (td, *J* = 7.8, 1.2 Hz, 2H), 7.08 (ddd, *J* = 9.7, 8.2, 1.1 Hz, 1H), 7.05 (t, *J* = 7.3 Hz, 1H), 6.69 (s, 1H), 4.76 (s, 2H), 4.61 (s, 2H). ^13^C NMR (151 MHz, CDCl3) δ 162.88, 161.72, 160.08, 158.30, 148.63, 144.60, 130.20 (d, *J* = 4.3 Hz), 129.90 (d, *J* = 8.2 Hz), 124.73 (d, *J* = 14.7 Hz), 124.39 (d, *J* = 3.7 Hz), 118.23, 115.54, 113.48, 101.57, 71.86, 66.87 (d, *J* = 3.8 Hz). HRMS (ES+) *m*/*z* calc. for C_16_H_13_FN_2_O_3_ [M + H]⁺: 301.0983; found: 301.0994.

##### 2-(((3-Fluorobenzyl)oxy)methyl)-9-hydroxy-4*H*-pyrido [1,2-a]pyrimidin-4-one (**32**)

The title compound was obtained as a white solid (15.6 mg, 0.052 mmol, 20.8%). ^1^H NMR (600 MHz, CDCl_3_) δ 8.58 (dd, *J* = 7.1, 1.3 Hz, 1H), 7.37 (td, *J* = 7.9, 5.8 Hz, 1H), 7.21–7.17 (m, 2H), 7.15 (dt, *J* = 9.6, 2.1 Hz, 1H), 7.09 (t, *J* = 7.3 Hz, 1H), 7.06–7.01 (m, 1H), 6.71 (s, 1H), 4.70 (s, 2H), 4.60 (d, *J* = 1.0 Hz, 2H). ^13^C NMR (151 MHz, CDCl_3_) δ 163.34, 162.12, 161.71, 157.65, 148.00, 143.98, 139.63 (d, *J* = 7.1 Hz), 129.62 (d, *J* = 7.9 Hz), 122.52 (d, *J* = 3.0 Hz), 117.66, 114.99, 114.14 (dd, *J* = 62.2, 21.4 Hz), 113.01, 100.92, 71.89 (d, *J* = 2.7 Hz), 71.10. HRMS (ES+) *m*/*z* calc’d for C_16_H_13_FN_2_O_3_ [M + H]⁺: 301.0983; found: 301.0991.

##### 2-(((4-Fluorobenzyl)oxy)methyl)-9-hydroxy-4*H*-pyrido [1,2-a]pyrimidin-4-one (**33**)

The title compound was obtained as a white solid (9.3 mg, 0.031 mmol, 12.4%). ^1^H NMR (600 MHz, CDCl3) δ 8.55 (dd, *J* = 7.2, 1.4 Hz, 1H), 7.39–7.36 (m, 2H), 7.17 (dd, *J* = 7.5, 1.3 Hz, 1H), 7.06 (ddd, *J* = 12.0, 6.7, 2.7 Hz, 3H), 6.67 (s, 1H), 4.64 (s, 2H), 4.56 (s, 2H). ^13^C NMR (151 MHz, CDCl3) δ 163.49, 162.91, 161.86, 158.31, 148.64, 144.61, 133.35 (d, *J* = 3.3 Hz), 129.70 (d, *J* = 8.2 Hz), 118.25, 115.75–115.46 (m), 113.57, 101.55, 72.59, 71.55.

HRMS (ES+) *m*/*z* calc. for C_16_H_13_FN_2_O_3_ [M + H]⁺: 301.0983; found: 301.0983.

##### 2-(((2-Chlorobenzyl)oxy)methyl)-9-hydroxy-4*H*-pyrido [1,2-a]pyrimidin-4-one (**34**)

The title compound was obtained as a yellow residue (7.4 mg, 0.023 mmol, 9.4%). ^1^H NMR (600 MHz, CDCl_3_) δ 8.56 (dd, *J* = 7.1, 1.3 Hz, 1H), 7.58 (dd, *J* = 7.6, 1.7 Hz, 1H), 7.38 (dd, *J* = 7.9, 1.4 Hz, 1H), 7.31 (td, *J* = 7.5, 1.4 Hz, 2H), 7.29–7.23 (m, 8H), 7.18 (dd, *J* = 7.5, 1.3 Hz, 1H), 7.06 (t, *J* = 7.3 Hz, 1H), 6.72 (d, *J* = 0.9 Hz, 1H), 4.79 (s, 2H), 4.65 (d, *J* = 0.9 Hz, 2H). ^13^C NMR (151 MHz, CDCl_3_) δ 162.2, 157.7, 148.0, 144.0, 134.8, 132.4, 128.9, 128.5, 126.5, 117.7, 115.0, 113.0, 100.9, 71.5, 69.8. Quaternary C missing. HRMS (ES+) *m*/*z* calc. for C_16_H_13_ClN_2_O_3_ [M + H]⁺: 317.0688; found: 317.0701.

##### 2-(((3-Chlorobenzyl)oxy)methyl)-9-hydroxy-4*H*-pyrido [1,2-a]pyrimidin-4-one (**35**)

The title compound was obtained as a yellow residue (11.6 mg, 0.037 mmol, 14.6%). ^1^H NMR (600 MHz, CDCl_3_) δ 8.56 (dd, *J* = 7.2, 1.3 Hz, 1H), 7.41–7.39 (m, 1H), 7.33–7.26 (m, 3H), 7.18 (dd, *J* = 7.5, 1.3 Hz, 1H), 7.06 (t, *J* = 7.3 Hz, 1H), 6.68 (s, 1H), 4.66 (s, 2H), 4.57 (s, 2H). ^13^C NMR (151 MHz, CDCl_3_) δ 162.1, 157.7, 148.0, 144.0, 139.1, 134.0, 129.4, 127.6, 127.2, 125.2, 117.7, 115.0, 113.0, 100.9, 71.9, 71.1. HRMS (ES+) *m*/*z* calc. for C_16_H_13_ClN_2_O_3_ [M + H]⁺: 317.0688; found: 317.0703.

##### 2-(((4-Chlorobenzyl)oxy)methyl)-9-hydroxy-4*H*-pyrido [1,2-a]pyrimidin-4-one (**36**)

The title compound was obtained as a white solid (8 mg, 0.025 mmol, 10.7%). 1H NMR (600 MHz, DMSO-d_6_) δ 8.46 (dd, *J* = 6.9, 1.4 Hz, 1H), 7.45 (s, 4H), 7.25–7.15 (m, 2H), 6.43 (s, 1H), 4.66 (s, 2H), 4.56 (s, 2H). 13C NMR (151 MHz, DMSO-d_6_) δ 162.8, 157.4, 144.9, 137.1, 132.1, 129.4, 128.4, 117.1, 116.2, 115.6, 99.3, 71.4, 71.2. Quaternary carbon missing. HRMS (ES+) *m*/*z* calc. for C_16_H_13_ClN_2_O_3_ [M + H]⁺: 317.0688; found: 317.0698.

##### 9-Hydroxy-2-(((3-hydroxybenzyl)oxy)methyl)-4*H*-pyrido [1,2-a]pyrimidin-4-one (**37**)

The title compound was obtained as a gray solid (18 mg, 0.06 mmol, 24%). ^1^H NMR (600 MHz, DMSO-d_6_) δ 10.84–10.00 (m, 1H), 8.48 (d, *J* = 6.8 Hz, 1H), 7.26 (q, *J* = 7.5 Hz, 2H), 7.22 (t, *J* = 7.3 Hz, 1H), 7.01 (t, *J* = 1.9 Hz, 1H), 6.92 (d, *J* = 7.5 Hz, 1H), 6.90 (dd, *J* = 8.2, 2.6 Hz, 1H), 6.41 (s, 1H), 5.14 (s, 2H), 4.48 (s, 2H). ^13^C NMR (151 MHz, DMSO-d_6_) δ 161.7, 157.8, 157.3, 150.0, 144.9, 144.5, 129.3, 119.2, 117.5, 116.3, 115.9, 112.9, 112.6, 99.7, 68.9, 62.7. HRMS (ES+) *m*/*z* calc. for C_16_H_14_N_2_O_4_ [M + H]⁺: 299.1027; found: 299.1033.

##### 9-Hydroxy-2-(((3-methylbenzyl)oxy)methyl)-4*H*-pyrido [1,2-a]pyrimidin-4-one (**38**)

The title compound was obtained as a yellow residue (6 mg, 0.02 mmol, 8.1%). ^1^H NMR (600 MHz, CDCl_3_) δ 8.55 (dd, *J* = 7.1, 1.3 Hz, 1H), 7.27 (t, *J* = 7.6 Hz, 1H), 7.23–7.21 (m, 1H), 7.21–7.18 (m, 1H), 7.16 (dd, *J* = 7.5, 1.3 Hz, 1H), 7.15–7.12 (m, 1H), 7.05 (t, *J* = 7.3 Hz, 1H), 6.70 (s, 1H), 4.65 (s, 2H), 4.56 (d, *J* = 0.9 Hz, 2H), 2.37 (s, 3H). ^13^C NMR (151 MHz, CDCl_3_) δ 163.1, 158.3, 148.6, 144.6, 138.4, 137.5, 128.9, 128.7, 128.6, 125.0, 118.2, 115.5, 113.4, 101.6, 73.4, 71.6, 21.6. HRMS (ES+) *m*/*z* calc. for C_17_H_16_N_2_O_3_ [M + H]⁺: 297.1234; found: 297.1243.

##### 9-Hydroxy-2-(((4-methylbenzyl)oxy)methyl)-4*H*-pyrido [1,2-a]pyrimidin-4-one (**39**)

The title compound was obtained as a yellow residue (6 mg, 0.02 mmol, 8.1%). ^1^H NMR (600 MHz, CDCl_3_) δ 8.55 (dd, *J* = 7.2, 1.3 Hz, 1H), 7.31–7.27 (m, 2H), 7.20–7.17 (m, 2H), 7.16 (dd, *J* = 7.5, 1.3 Hz, 1H), 7.05 (t, *J* = 7.3 Hz, 1H), 6.71–6.67 (m, 1H), 4.64 (s, 2H), 4.54 (d, *J* = 0.9 Hz, 2H), 2.36 (s, 3H). ^13^C NMR (151 MHz, CDCl_3_) δ 163.2, 158.3, 148.6, 144.6, 137.9, 134.5, 129.4, 128.1, 118.2, 115.5, 113.4, 101.6, 73.2, 71.4, 21.3. HRMS (ES+) *m*/*z* calc. for C_17_H_16_N_2_O_3_+ H]⁺: 297.1234; found: 297.1241.

##### 9-Hydroxy-2-(((3-nitrobenzyl)oxy)methyl)-4*H*-pyrido [1,2-a]pyrimidin-4-one (**40**)

The title compound was obtained as a yellow solid (33 mg, 0.101 mmol, 40.2%). ^1^H NMR (600 MHz, CDCl_3_) δ 8.56 (dd, *J* = 7.2, 1.3 Hz, 1H), 8.27 (t, *J* = 2.0 Hz, 1H), 8.19 (ddd, *J* = 8.2, 2.4, 1.0 Hz, 1H), 7.75 (ddd, *J* = 7.5, 1.8, 1.0 Hz, 1H), 7.57 (t, *J* = 7.9 Hz, 1H), 7.19 (dd, *J* = 7.5, 1.3 Hz, 1H), 7.07 (t, *J* = 7.3 Hz, 1H), 6.67 (d, *J* = 0.8 Hz, 1H), 4.78 (s, 2H), 4.62 (d, *J* = 0.9 Hz, 2H). ^13^C NMR (151 MHz, CDCl_3_) δ 162.3, 158.2, 148.6, 144.6, 139.9, 133.4, 129.7, 123.0, 122.4, 118.3, 115.7, 113.7, 101.5, 72.1, 72.0. HRMS (ES + ) *m*/*z* calc. for C_16_H_13_N_3_O_5_ [M + H]⁺: 328.0928; found: 328.0942.

##### 2-(((3,5-Dimethoxybenzyl)oxy)methyl)-9-hydroxy-4*H*-pyrido [1,2-a]pyrimidin-4-one (**41**)

The title compound was obtained as a yellow solid (77 mg, 0.225 mmol, 89.5%). ^1^H NMR (600 MHz, CDCl_3_) δ 8.55 (dd, *J* = 7.2, 1.3 Hz, 1H), 7.16 (dd, *J* = 7.5, 1.3 Hz, 1H), 7.07–7.02 (m, 2H), 6.82 (s, 2H), 6.73 (s, 1H), 4.70 (s, 2H), 4.62 (s, 2H), 3.81 (s, 3H), 3.79 (s, 3H). ^13^C NMR (151 MHz, CDCl_3_) δ 163.2, 158.3, 148.6, 144.6, 137.9, 134.5, 129.4, 128.1, 118.2, 115.5, 113.4, 101.6, 73.2, 71.4, 21.3. HRMS (ES + ) *m*/*z* calc. for C_18_H_18_N_2_O5 [M + H]⁺: 343.1289; found: 343.1278.

#### 3.2.5. General Synthesis of Indoline and Isoindoline PPO

Compound **5** (210 mg, 0.5 mmol, 1.0 equiv.) was dissolved in 1 mL DMF. The corresponding isoindoline or indoline (3.0 mmol, 3.0 equiv.) was added, and the reaction was stirred overnight at room temperature. Upon completion, the reaction was filtered, and the filtrate concentrated. The crude product was crystallized from DCM/MeOH and optionally further purified by RP ACC (0–100% ACN in 0.1% formic acid) and lyophilized to yield final compounds.

##### 9-Hydroxy-2-(isoindolin-2-ylmethyl)-*4H*-pyrido [1,2-a]pyrimidin-4-one (**42**)

The title compound was obtained as a yellow solid (40 mg, 0.136 mmol, 27.4%). ^1^H NMR (600 MHz, DMSO-d_6_) δ 8.47 (d, *J* = 6.9 Hz, 1H), 7.27–7.22 (m, 3H), 7.22–7.16 (m, 3H), 6.48 (s, 1H), 4.02 (s, 4H), 3.97 (s, 2H). ^13^C NMR (151 MHz, DMSO-d_6_) δ 163.5, 157.5, 139.9, 126.7, 122.2, 116.1, 115.4, 101.1, 60.3, 58.6.

HRMS (ES+) *m*/*z* calc. for C_17_H_15_N_3_O_2_ [M + H]⁺: 294.1238; found: 294.1243.

##### 2-((5-Chloroisoindolin-2-yl)methyl)-9-hydroxy-4*H*-pyrido [1,2-a]pyrimidin-4-one (**43**)

The title compound was obtained as a light tan solid (23 mg, 0.07 mmol, 14.1%). ^1^H NMR (600 MHz, DMSO-d_6_) δ 8.48 (dd, *J* = 7.0, 1.4 Hz, 1H), 7.34 (s, 1H), 7.29–7.24 (m, 3H), 7.20 (t, *J* = 7.3 Hz, 1H), 6.47 (s, 1H), 4.03 (d, *J* = 11.0 Hz, 4H), 3.98 (s, 2H). ^13^C NMR (151 MHz, DMSO-d_6_) δ 163.1, 157.4, 149.9, 144.6, 142.3, 138.8, 131.3, 126.7, 124.0, 122.5, 117.4, 116.1, 115.5, 101.2, 60.0, 58.2, 58.0. HRMS (ES+) *m*/*z* calc. for C_17_H_14_ClN_3_O_2_ [M + H]⁺: 328.0847; found: 328.0862.

##### 2-((5-Bromoisoindolin-2-yl)methyl)-9-hydroxy-4*H*-pyrido [1,2-a]pyrimidin-4-one (**44**)

Tile compound was obtained as a brown solid (182 mg, 0.491 mmol, 49%). ^1^H NMR (600 MHz, DMSO-d_6_) δ 8.48 (dd, *J* = 7.0, 1.4 Hz, 1H), 7.46 (d, *J* = 1.9 Hz, 1H), 7.38 (dd, *J* = 8.0, 1.9 Hz, 1H), 7.24 (dd, *J* = 7.6, 1.4 Hz, 1H), 7.23–7.18 (m, 2H), 6.47 (s, 1H), 4.05–3.92 (m, 6H). ^13^C NMR (151 MHz, DMSO-d_6_) δ 163.5, 157.4, 149.9, 144.6, 142.9, 139.5, 129.4, 125.3, 124.3, 119.5, 117.4, 116.0, 115.5, 101.2, 60.1, 58.2, 58.0. HRMS (ES+) *m*/*z* calc. for C_17_H_14_BrN_3_O_2_ [M + H]⁺: 372.0343; found: 372.0328.

##### 9-Hydroxy-2-((5-phenylisoindolin-2-yl)methyl)-4*H*-pyrido [1,2-a]pyrimidin-4-one (**45**)

**44** (112 mg, 0.300 mmol, 1 equiv.) and phenyl boronic acid (37 mg, 0.36 mmol, 1.2 equiv.) were dissolved in a mixture of 2 M aqueous K_2_CO_3_ (1 mL) and ACN (1 mL). Tetrakis(triphenylphosphine) palladium (17 mg, 0.015 mmol, 0.05 equiv.) was added, and the mixture was heated to 80 °C. After 18 h, the reaction mixture was removed from the heat, diluted with EtOAc (5 mL), and filtered through celite. The filtrate was then washed with 5 mL sat. NaHCO3 and the organic phase extracted with 1 M HCl (5 × 3 mL). The combined aqueous extracts were then basified with 1 mL 15% NaOH and extracted with EtOAc (5 × 3 mL). The combined organic extracts were dried over Na_2_SO_4_ and concentrated in vacuo. The crude product was further purified by RP ACC (0–100% ACN in 0.1% formic acid) to yield the title compound as a white solid (7 mg, 0.019 mmol, 6.3%). ^1^H NMR (600 MHz, DMSO-d_6_) δ 8.49 (d, *J* = 6.9 Hz, 1H), 7.66–7.61 (m, 2H), 7.54 (s, 1H), 7.50 (dd, *J* = 7.8, 1.7 Hz, 1H), 7.45 (t, *J* = 7.6 Hz, 2H), 7.37–7.32 (m, 2H), 7.28–7.23 (m, 1H), 7.21 (t, *J* = 7.2 Hz, 1H), 6.51 (s, 1H), 4.07 (d, *J* = 11.8 Hz, 4H), 3.99 (s, 2H). ^13^C NMR (151 MHz, DMSO-d_6_) δ 163.3, 157.5, 149.9, 140.9, 140.4, 139.3, 139.0, 134.1, 130.0, 128.9, 127.3, 127.2, 126.7, 125.4, 122.7, 120.7, 117.4, 116.1, 115.4, 101.1, 60.3, 58.6, 58.4. HRMS (ES+) *m*/*z* calc. for C_23_H_19_N_3_O_2_ [M + H]⁺: 370.1551; found: 370.1568.

##### 9-Hydroxy-2-(indolin-1-ylmethyl)-4*H*-pyrido [1,2-a]pyrimidin-4-one (**46**)

The title compound was obtained as a light yellow powder (30 mg, 0.102 mmol, 20.5%). ^1^H NMR (600 MHz, DMSO-d_6_) δ 8.46 (dd, *J* = 7.0, 1.4 Hz, 1H), 7.25 (dd, *J* = 7.6, 1.4 Hz, 1H), 7.19 (t, *J* = 7.3 Hz, 1H), 7.06 (dd, *J* = 7.3, 1.3 Hz, 1H), 6.96 (td, *J* = 7.7, 1.3 Hz, 1H), 6.59 (td, *J* = 7.3, 0.9 Hz, 1H), 6.50 (d, *J* = 7.8 Hz, 1H), 6.33 (s, 1H), 4.31 (s, 2H), 3.49 (t, *J* = 8.4 Hz, 2H), 2.98 (t, *J* = 8.4 Hz, 2H). ^13^C NMR (151 MHz, DMSO-d_6_) δ 163.1, 157.4, 151.8, 150.0, 144.8, 129.4, 127.1, 124.3, 117.4, 117.3, 116.1, 115.6, 106.7, 100.2, 54.0, 53.4, 28.1. HRMS (ES+) *m*/*z* calc. for C_17_H_15_N_3_O_2_ [M + H]⁺: 294.1238; found: 294.1230.

##### 2-((5-Chloroindolin-1-yl)methyl)-9-hydroxy-4*H*-pyrido [1,2-a]pyrimidin-4-one (**47**)

The title compound was obtained as a beige solid (50 mg, 0.153 mmol, 45.9%). ^1^H NMR (600 MHz, CDCl_3_) δ 8.54 (dd, *J* = 7.2, 1.3 Hz, 1H), 7.20 (dd, *J* = 7.5, 1.3 Hz, 1H), 7.11–7.05 (m, 2H), 6.99 (dd, *J* = 8.3, 2.1 Hz, 1H), 6.53 (s, 1H), 6.34 (d, *J* = 8.3 Hz, 1H), 4.28 (s, 2H), 3.56 (t, *J* = 8.4 Hz, 2H), 3.05 (t, *J* = 8.4 Hz, 2H). ^13^C NMR (151 MHz, CDCl_3_) δ 162.7, 158.1, 150.4, 148.6, 144.6, 131.6, 127.2, 125.0, 123.0, 118.3, 115.8, 113.9, 107.6, 102.0, 54.4, 54.3, 28.6. HRMS (ES+) *m*/*z* calc. for C_17_H_14_ClN_3_O_2_ [M + H]⁺: 328.0848; found: 328.0855.

##### 2-((5-Bromoindolin-1-yl)methyl)-9-hydroxy-4*H*-pyrido [1,2-a]pyrimidin-4-one (**48**)

**5** (211 mg, 1.0 mmol, 1.0 equiv.) and 5-bromoindoline (238 mg, 1.2 mmol 1.2 equiv.) were dissolved in DMF. DIPEA (349 µL, 2.0 mmol, 2.0 equiv.) was added and the reaction was stirred overnight at 37 °C. The reaction mixture was then concentrated to remove DMF, and purified using normal-phase ACC (0–5% MeOH in DCM). The title compound was obtained as a yellow solid (105 mg, 0.283 mmol, 28%). ^1^H NMR (600 MHz, DMSO-d_6_) δ 10.27 (s, 1H), 8.46 (dd, *J* = 7.0, 1.4 Hz, 1H), 7.28–7.23 (m, 1H), 7.22–7.18 (m, 2H), 7.09 (dd, *J* = 8.3, 2.1 Hz, 1H), 6.46 (d, *J* = 8.3 Hz, 1H), 6.30 (s, 1H), 4.31 (s, 2H), 3.55 (t, *J* = 8.5 Hz, 2H), 3.01 (t, *J* = 8.5 Hz, 2H). ^13^C NMR (151 MHz, DMSO-d_6_) δ 162.6, 157.3, 151.0, 149.8, 144.8, 132.4, 129.5, 127.0, 117.5, 116.1, 115.7, 108.1, 107.9, 100.2, 53.5, 53.3, 27.8. HRMS (ES+) *m*/*z* calc. for C_17_H_14_BrN_3_O_2_ [M + H]⁺: 372.0343; found: 372.0351.

##### 2-((6-Bromoindolin-1-yl)methyl)-9-hydroxy-4*H*-pyrido [1,2-a]pyrimidin-4-one (**49**)

The title compound was synthesized from **5** (211 mg, 1.0, 1.0 equiv.), 6-bromoindoline (238 mg, 1.2 mmol, 1.2 equiv.), and DIPEA (349 µL, 2.0 mmol, 2.0 equiv.). Following normal-phase ACC, the compound was further purified by preparative HPLC to yield the title compound as a brown solid (110 mg, 0.296 mmol, 30%). ^1^H NMR (600 MHz, DMSO-d_6_) δ 8.48 (dd, *J* = 7.0, 1.4 Hz, 1H), 7.29 (dd, *J* = 7.6, 1.4 Hz, 1H), 7.25–7.21 (m, 1H), 6.98 (dt, *J* = 7.7, 1.2 Hz, 1H), 6.70 (dd, *J* = 7.7, 1.8 Hz, 1H), 6.69 (d, *J* = 1.8 Hz, 1H), 6.31 (s, 1H), 4.36 (s, 2H), 3.57 (t, *J* = 8.5 Hz, 2H), 2.96 (td, *J* = 8.6, 1.2 Hz, 2H). ^13^C NMR (151 MHz, DMSO-d_6_) δ 162.0, 157.2, 153.3, 149.6, 144.5, 128.9, 125.8, 120.2, 119.4, 117.6, 116.4, 116.2, 108.9, 100.2, 53.2, 52.8, 27.5. HRMS (ES+) *m*/*z* calc. for C_17_H_14_BrN_3_O_2_ [M + H]⁺: 372.0343; found: 372.0351.

##### 9-Hydroxy-2-((7-methylindolin-1-yl)methyl)-4*H*-pyrido [1,2-a]pyrimidin-4-one (**50**)

The title compound was obtained as a light brown solid (78 mg, 0.254 mmol, 76.5%). ^1^H NMR (600 MHz, CDCl_3_) δ 8.54 (dd, *J* = 7.1, 1.3 Hz, 1H), 7.15 (dd, *J* = 7.6, 1.3 Hz, 1H), 7.05 (t, *J* = 7.3 Hz, 1H), 7.01 (d, *J* = 7.4 Hz, 1H), 6.87 (d, *J* = 7.5 Hz, 1H), 6.72 (t, *J* = 7.5 Hz, 1H), 6.66 (s, 1H), 4.47 (s, 2H), 3.51 (t, *J* = 8.7 Hz, 2H), 3.06 (t, *J* = 8.6 Hz, 2H), 2.30 (s, 3H). ^13^C NMR (151 MHz, CDCl_3_) δ 162.8, 158.2, 150.5, 148.6, 144.6, 131.6, 127.2, 125.0, 122.9, 118.2, 115.7, 113.7, 107.5, 102.1, 54.4, 54.3, 28.7. HRMS (ES+) *m*/*z* calc. for C_18_H_17_N_3_O_2_ [M + H]⁺: 308.1394; found: 308.1401.

##### 9-Hydroxy-2-((6-methoxyindolin-1-yl)methyl)-4*H*-pyrido [1,2-a]pyrimidin-4-one (**51**)

The title compound was obtained as a yellow solid (48 mg, 0.148 mmol, 44.4%). ^1^H NMR (600 MHz, DMSO-d_6_) δ 7.80 (d, *J* = 6.7 Hz, 1H), 7.09 (dd, *J* = 7.8, 6.9 Hz, 1H), 6.80 (dd, *J* = 7.8, 1.2 Hz, 1H), 6.36 (d, *J* = 8.1 Hz, 1H), 6.29 (s, 1H), 6.23 (d, *J* = 2.2 Hz, 1H), 5.82 (dd, *J* = 8.1, 2.2 Hz, 1H), 4.34 (s, 2H), 3.56 (s, 3H), 3.03 (t, *J* = 8.2 Hz, 2H), 2.77 (t, *J* = 8.0 Hz, 2H). ^13^C NMR (151 MHz, DMSO-d_6_) δ 158.4, 158.1, 157.7, 156.9, 152.3, 146.8, 123.8, 121.8, 119.0, 115.1, 110.5, 103.4, 100.0, 95.7, 54.9, 54.8, 54.5, 54.0, 26.9. HRMS (ES+) *m*/*z* calc. for C_18_H_17_N_3_O_3_ [M + H]⁺: 324.1343; found: 324.1328.

##### Methyl1-((9-hydroxy-4-oxo-4*H*-pyrido [1,2-a]pyrimidin-2-yl)methyl)indoline-5-carboxylate (**52**)

The title compound was obtained as a light beige solid (35 mg, 0.1 mmol, 20%). ^1^H NMR (600 MHz, DMSO-d_6_) δ 10.28 (s, 1H), 8.46 (dd, *J* = 7.0, 1.4 Hz, 1H), 7.65 (dd, *J* = 8.3, 1.8 Hz, 1H), 7.60 (q, *J* = 1.4 Hz, 1H), 7.26 (dd, *J* = 7.6, 1.4 Hz, 1H), 7.20 (t, *J* = 7.3 Hz, 1H), 6.53 (d, *J* = 8.3 Hz, 1H), 6.25 (s, 1H), 4.45 (s, 2H), 3.74 (s, 3H), 3.71 (t, *J* = 8.7 Hz, 2H), 3.08 (t, *J* = 8.6 Hz, 2H). ^13^C NMR (151 MHz, DMSO-d_6_) δ 166.3, 162.1, 157.3, 155.5, 149.8, 144.8, 130.5, 129.4, 125.2, 117.5, 117.4, 116.2, 115.7, 104.9, 100.0, 52.7, 52.1, 51.3, 27.1. HRMS (ES+) *m*/*z* calc. for C_19_H_17_N_3_O_4_ [M + H]⁺: 352.1292; found: 352.1305.

##### Methyl-1-((9-hydroxy-4-oxo-4*H*-pyrido [1,2-a]pyrimidin-2-yl)methyl)indoline-6-carboxylate (**53**)

The title compound was obtained as a fluffy white solid (127 mg, 0.362 mmol, 72.2%). ^1^H NMR (600 MHz, DMSO-d_6_) δ 8.46 (dd, *J* = 7.1, 1.4 Hz, 1H), 7.26 (dd, *J* = 7.6, 1.6 Hz, 2H), 7.23–7.16 (m, 2H), 6.95 (s, 1H), 6.29 (s, 1H), 4.39 (s, 2H), 3.76 (s, 3H), 3.61 (t, *J* = 8.5 Hz, 2H), 3.08 (t, *J* = 8.5 Hz, 2H). ^13^C NMR (151 MHz, DMSO-d_6_) δ 166.6, 162.7, 157.3, 152.0, 150.0, 144.9, 135.6, 128.9, 124.3, 119.2, 117.3, 116.2, 115.7, 105.7, 100.0, 53.3, 53.2, 51.8, 28.1. Aromatic C missing. HRMS (ES+) *m*/*z* calc. for C_19_H_17_N_3_O_4_ [M + H]⁺: 352.1292; found: 352.1298.

##### 1-((9-Hydroxy-4-oxo-4*H*-pyrido [1,2-a]pyrimidin-2-yl)methyl)indoline-6-carboxylic acid (**54**)

Compound 53 (65 mg, 0.185 mmol, 1.0 equiv.) was suspended in 2 mL MeOH. 246 µL of 3 M aqueous NaOH (0.74 mmol, 4 equiv.) was added. The mixture was heated to reflux. After 1.5 h, the mixture was removed from heat and acidified with 1 M HCl. The resulting precipitate was filtered and washed with cold 1 M HCl to give 40 mg of light yellow solid (0.119 mmol, 64.5%). ^1^H NMR (600 MHz, DMSO-d_6_) δ 8.58 (dd, *J* = 7.0, 1.2 Hz, 1H), 7.65 (d, *J* = 7.6 Hz, 1H), 7.44 (t, *J* = 7.4 Hz, 1H), 7.30 (dd, *J* = 7.5, 1.4 Hz, 1H), 7.19 (d, *J* = 7.6 Hz, 1H), 7.03 (d, *J* = 1.4 Hz, 1H), 6.42 (s, 1H), 4.47 (s, 2H), 3.58 (t, *J* = 8.5 Hz, 2H), 3.08 (t, *J* = 8.4 Hz, 2H). ^13^C NMR (151 MHz, DMSO-d_6_) δ 167.7, 156.2, 151.6, 148.1, 142.7, 135.2, 130.1, 124.2, 119.9, 118.3, 118.0, 106.6, 100.1, 53.4, 51.5, 28.1. HRMS (ES+) *m*/*z* calc. for C_18_H_15_N_3_O_4_ [M + H]⁺: 338.1135; found: 338.1139.

##### 9-Hydroxy-2-((5-phenylindolin-1-yl)methyl)-4*H*-pyrido [1,2-a]pyrimidin-4-one (**55**)

**5** (96 mg, 0.473 mmol, 1.0 equiv.) and 5-phenylindoline (100 mg, 0.512, 1.1 equiv.) were dissolved in 1 mL DMF. Triethylamine (71 µL, 0.512 mmol, 1.1 equiv.) was added and the reaction was stirred for 48 h at room temperature. The reaction mixture was then concentrated, and the residue diluted with 10 mL sat. NaHCO3 and 10 mL EtOAc. The organic layer was separated, and the aqueous layer was further extracted with EtOAc (2 × 5 mL). The combined organic extracts were dried over Na_2_SO_4_ and concentrated in vacuo. The crude product was purified using normal-phase ACC (0–10% MeOH in DCM) followed by purification by RP ACC (0–100% ACN in 0.1% formic acid) to yield product as a light yellow solid (42 mg, 0.114 mmol, 40.8%). ^1^H NMR (600 MHz, DMSO-d_6_) δ 10.29 (s, 1H), 8.47 (dd, *J* = 6.9, 1.4 Hz, 1H), 7.57–7.52 (m, 2H), 7.42–7.34 (m, 3H), 7.29 (dd, *J* = 8.1, 2.0 Hz, 1H), 7.27 (dd, *J* = 7.6, 1.4 Hz, 1H), 7.25–7.17 (m, 2H), 6.59 (d, *J* = 8.1 Hz, 1H), 6.35 (s, 1H), 4.38 (s, 2H), 3.58 (t, *J* = 8.4 Hz, 2H), 3.07 (t, *J* = 8.4 Hz, 2H). ^13^C NMR (151 MHz, DMSO-d_6_) δ 163.0, 157.3, 151.4, 149.9, 144.8, 140.9, 130.3, 129.6, 128.7, 125.9, 125.8, 125.7, 122.8, 117.5, 116.1, 115.6, 106.8, 100.2, 53.7, 53.4, 28.1. HRMS (ES+) *m*/*z* calc. for C_23_H_19_N_3_O_2_ [M + H]⁺: 370.1550; found: 370.1563.

##### 9-Hydroxy-2-((6-phenylindolin-1-yl)methyl)-4*H*-pyrido [1,2-a]pyrimidin-4-one (**56**)

The title compound was synthesized from **5** (96 mg, 0.473 mmol, 1.0 equiv.) and 6-phenylindoline (100 mg, 0.512, 1.1 equiv.) in the same manner as **55** to yield the title compound as a yellow solid (58 mg, 0.157 mmol, 33.2%). ^1^H NMR (600 MHz, DMSO-d_6_) δ 8.46 (dd, *J* = 7.1, 1.6 Hz, 1H), 7.60–7.53 (m, 2H), 7.40–7.35 (m, 2H), 7.31–7.22 (m, 2H), 7.19 (dd, *J* = 8.3, 6.3 Hz, 1H), 7.14 (d, *J* = 7.7 Hz, 1H), 6.88 (dd, *J* = 7.5, 1.8 Hz, 1H), 6.82 (d, *J* = 1.7 Hz, 1H), 6.36 (s, 1H), 4.43 (s, 2H), 3.57 (t, *J* = 8.5 Hz, 2H), 3.03 (t, *J* = 8.7 Hz, 2H). ^13^C NMR (151 MHz, DMSO-d_6_) δ 163.1, 157.4, 152.4, 149.8, 144.8, 141.1, 139.7, 128.9, 128.7, 126.9, 126.6, 124.6, 117.5, 116.1, 116.0, 115.6, 104.9, 100.3, 53.6, 53.4, 27.8. HRMS (ES+) *m*/*z* calc. for C_23_H_19_N_3_O_2_ [M + H]⁺: 370.1550; found: 370.1565.

#### 3.2.6. Synthesis of Catch and Anchor Compounds

##### 2-(Chloromethyl)-9-hydroxy-8-iodo-4*H*-pyrido [1,2-a]pyrimidin-4-one (**57**)

**5** (1.05 g, 5.0 mmol, 1.0 equiv.) was suspended in 30 mL of anhydrous EtOH. Iodine (1.40 g, 5.5 mmol, 1.1 equiv.) was added, followed by 600 µL of 30% w/w hydrogen peroxide. The reaction mixture was stirred for 24 h at room temperature. The smooth suspension was then filtered and washed lightly with EtOH to yield the title compound as an off-white fine solid (1.42 g, 4.22 mmol, 84.1%). ^1^H NMR (600 MHz, DMSO-d_6_) δ 8.21 (d, *J* = 7.4 Hz, 1H), 7.59 (d, *J* = 7.4 Hz, 1H), 6.58 (s, 1H), 4.69 (s, 2H). ^13^C NMR (151 MHz, DMSO-d_6_) δ 160.7, 157.3, 150.9, 142.6, 124.7, 117.7, 102.4, 87.4, 45.7.

##### 8-iodo-2-(iodomethyl)-9-methoxy-4*H*-pyrido [1,2-a]pyrimidin-4-one (**58**)

A round-bottom flask was charged with **57** (2.20 g, 6.54 mmol, 3.0 equiv.) and anhydrous potassium carbonate (3.61 g, 26.15 mmol, 4 equiv.), then flushed with argon. Ice-cold anhydrous DMF (40 mL) was added, and the suspension was cooled to –10 °C. Methyl iodide (3.02 mL, 26.15 mmol, 4 equiv.) was added dropwise, and the reaction stirred at –10 °C. After 30 min the temperature was raised to 4 °C and the reaction was stirred for 16 h. Upon reaction completion, 150 mL of H_2_O was added to dilute the reaction mixture. The resulting beige precipitate was filtered and dried before purification using normal-phase ACC (0–100% EtOAc in hexanes). The title compound was obtained as a beige solid (2.31 g, 5.26 mmol, 79.7%). ^1^H NMR (600 MHz, DMSO-d_6_) δ 8.43 (d, *J* = 7.4 Hz, 1H), 7.64 (d, *J* = 7.5 Hz, 1H), 6.59 (s, 1H), 4.70 (s, 2H), 4.02 (s, 3H). ^13^C NMR (151 MHz, DMSO-d_6_) δ 161.2, 157.4, 152.9, 145.6, 123.9, 122.6, 102.6, 100.8, 60.9, 45.9.

##### Tert-butyl ([1,1′-biphenyl]-4-ylmethyl)((8-iodo-9-methoxy-4-oxo-4*H*-pyrido [1,2-a] pyrimidin-2-yl)methyl)carbamate (**59**)

4-Phenyl benzylamine (2.42 g, 13.2 mmol, 3.0 equiv.) and compound **58** (1.94 g, 4.4 mmol, 1.0 equiv.) were dissolved in 30 mL DMF. The reaction mixture was stirred at room temperature for 1 h. Upon completion, the reaction was diluted with 100 mL H_2_O and extracted with EtOAc. The combined organic extracted were dried over Na_2_SO_4_ and concentrated to yield a crude brown solid. The crude solid was then dissolved in anhydrous DCM under argon atmosphere. Di-tert-butyl dicarbonate (2.88g, 13.2 mmol, 3.0 equiv.) and DMAP (161 mg, 1.32 mmol, 0.3 eq) dissolved in anhydrous DCM were added. The reaction mixture was stirred at room temperature for 24 h, then concentrated. The crude product was purified using normal-phase ACC (0–100% EtOAc in hexanes) to yield the title compound as a glassy yellow amorphous solid (1.33 g, 2.226 mmol, 50.6%). ^1^H NMR (600 MHz, DMSO-d_6_) δ 8.42 (t, *J* = 6.5 Hz, 1H), 7.66–7.57 (m, 5H), 7.47–7.43 (m, 2H), 7.39–7.33 (m, 3H), 6.24 (s, 1H), 4.56 (d, *J* = 28.0 Hz, 2H), 4.39 (d, *J* = 38.1 Hz, 2H), 4.01 (s, 3H), 1.46–1.52 (s, 9H). ^13^C NMR (151 MHz, DMSO-d_6_) δ 163.54 (d, *J* = 43.6 Hz), 157.20, 155.10 (d, *J* = 22.8 Hz), 152.74, 145.45, 139.84 (d, *J* = 8.0 Hz), 139.09, 137.32 (d, *J* = 19.6 Hz), 128.90, 128.17 (d, *J* = 26.7 Hz), 127.37, 126.74 (d, *J* = 11.4 Hz), 126.58, 123.53, 122.59, 100.70 (d, *J* = 2.7 Hz), 100.31 (d, *J* = 20.5 Hz), 79.50 (d, *J* = 25.1 Hz), 60.92 (d, *J* = 5.3 Hz), 51.18 (d, *J* = 59.5 Hz), 50.24 (d, *J* = 77.4 Hz), 27.97 (d, *J* = 10.8 Hz) *, (* Peaks in ^1^H and ^13^C NMR are broad and split due to their bulky structure and Boc rotamers. Compounds were sensitive to high temperature, preventing high temperature NMR).

#### 3.2.7. General Procedure for Sonogashira Couplings (**60**–**64**)

A round-bottom flask was charged with compound 59 (300 mg, 0.500 mmol, 1.0 equiv.), Pd(PPh_3_)Cl_2_ (18 mg, 0.025 mmol, 0.05 equiv.) and copper (I) iodide (5 mg, 0.025 mmol, 0.05 equiv.) and back-filled with argon. Anhydrous DCM (2 mL) and anhydrous Et_3_N (2 mL) were added to suspend the reagents. The corresponding alkynol (0.750 mmol, 1.5 equiv.) was added to 1 mL anhydrous DCM. The reaction mixture was stirred at room temperature for 16 h and then diluted with DCM (10 mL). The reaction mixture was then washed with 5% citric acid (2 × 10 mL), then sat. NaHCO3 (10 mL). The organic extract was dried over Na_2_SO_4_ and concentrated in vacuo. The resulting crude product was purified using normal-phase ACC (0–100% EtOAc in hexanes) to yield compounds 60–64 * (* Peaks in ^1^H and 13C NMR are broad and split due to their bulky structure and Boc rotamers. Compounds were sensitive to high temperature, preventing high temperature NMR).

##### Tert-butyl([1,1′-biphenyl]-4-ylmethyl)((8-(3-hydroxyprop-1-yn-1-yl)-9-methoxy-4-oxo-4*H*-pyrido [1,2-a]pyrimidin-2-yl)methyl)carbamate (**60**)

The title compound was obtained as a light yellow oil (190 mg, 0.361 mmol, 72.4%). ^1^H NMR (600 MHz, CDCl_3_) δ 8.67 (dd, *J* = 15.1, 7.4 Hz, 1H), 7.56 (dd, *J* = 11.3, 6.8 Hz, 4H), 7.47–7.38 (m, 2H), 7.36–7.27 (m, 3H), 6.94 (dd, *J* = 18.0, 7.5 Hz, 1H), 6.38 (d, *J* = 17.6 Hz, 1H), 4.60 (d, *J* = 47.4 Hz, 2H), 4.57 (s, 2H), 4.46 (d, *J* = 70.0 Hz, 2H), 4.17 (d, *J* = 10.1 Hz, 3H), 1.49 (d, *J* = 34.1 Hz, 9H). ^13^C NMR (151 MHz, CDCl_3_) δ 164.2, 163.9, 158.3, 156.0, 153.8, 153.7, 147.3, 140.9, 140.7, 140.5, 136.7, 136.6, 132.2, 132.1, 128.9, 128.6, 128.0, 127.4, 127.1, 121.7, 120.1, 117.1, 101.8, 101.8, 99.7, 81.0, 80.8, 79.2, 62.3, 51.6, 51.4, 51.3, 51.1, 50.3, 28.5, 28.5.

##### Tert-butyl([1,1′-biphenyl]-4-ylmethyl)((8-(4-hydroxybut-1-yn-1-yl)-9-methoxy-4-oxo-4*H*-pyrido [1,2-a]pyrimidin-2-yl)methyl)carbamate (**61**)

The title compound was obtained as a light yellow oil (225 mg, 0.417 mmol, 83.6%). ^1^H NMR (600 MHz, CDCl_3_) δ 8.66 (dd, *J* = 11.1, 7.3 Hz, 1H), 7.58–7.51 (m, 4H), 7.42 (q, *J* = 6.7, 6.3 Hz, 2H), 7.36–7.27 (m, 3H), 6.95 (dd, *J* = 14.5, 7.4 Hz, 1H), 6.37 (d, *J* = 19.8 Hz, 1H), 4.61 (d, *J* = 40.4 Hz, 2H), 4.45 (d, *J* = 69.3 Hz, 2H), 4.16 (d, *J* = 6.0 Hz, 3H), 3.86 (t, *J* = 6.4 Hz, 2H), 2.78 (t, *J* = 6.4 Hz, 2H), 1.48 (d, *J* = 33.6 Hz, 9H). ^13^C NMR (151 MHz, CDCl_3_) δ 164.3, 164.0, 158.4, 158.3, 155.9, 153.7, 147.4, 140.8, 140.7, 140.5, 136.8, 136.6, 132.2, 132.1, 132.1, 128.8, 128.6, 128.0, 127.4, 127.1, 121.6, 121.2, 117.4, 117.3, 101.4, 100.1, 100.1, 80.9, 80.7, 76.4, 62.0, 60.7, 51.4, 51.3, 51.0, 50.3, 28.5, 28.5, 24.4.

##### Tert-butyl([1,1′-biphenyl]-4-ylmethyl)((8-(5-hydroxypent-1-yn-1-yl)-9-methoxy-4-oxo-4*H*-pyrido [1,2-a]pyrimidin-2-yl)methyl)carbamate (**62**)

The title compound was obtained as a light yellow oil (203 mg, 0.367 mmol, 73.3%). ^1^H NMR (600 MHz, CDCl_3_) δ 8.67 (t, *J* = 7.7 Hz, 1H), 7.60–7.51 (m, 4H), 7.45–7.39 (m, 2H), 7.37–7.28 (m, 3H), 6.95 (dd, *J* = 10.3, 7.3 Hz, 1H), 6.36 (d, *J* = 20.6 Hz, 1H), 4.61 (d, *J* = 40.7 Hz, 2H), 4.46 (d, *J* = 70.4 Hz, 2H), 4.15 (d, *J* = 5.8 Hz, 3H), 3.83 (t, *J* = 6.1 Hz, 2H), 2.66 (t, *J* = 7.0 Hz, 2H), 1.90 (q, *J* = 6.5 Hz, 2H), 1.48 (d, *J* = 33.7 Hz, 9H). ^13^C NMR (151 MHz, CDCl_3_) δ 164.2, 164.0, 158.4, 158.3, 155.9, 153.5, 147.4, 140.9, 140.8, 140.5, 136.8, 136.7, 132.2, 128.9, 128.6, 128.1, 127.4, 127.2, 121.7, 121.6, 117.6, 117.5, 103.0, 102.9, 1f01.4, 80.9, 80.7, 75.4, 62.0, 60.5, 51.4, 51.3, 51.1, 50.3, 31.1, 28.5, 28.5, 16.7.

##### Tert-butyl([1,1′-biphenyl]-4-ylmethyl)((8-(6-hydroxyhex-1-yn-1-yl)-9-methoxy-4-oxo-4*H*-pyrido [1,2-a]pyrimidin-2-yl)methyl)carbamate (**63**)

The title compound was obtained as a light yellow oil (150 mg, 0.264 mmol, 53.0%). ^1^H NMR (600 MHz, CDCl_3_) δ 8.70–8.64 (m, 1H), 7.59–7.51 (m, 4H), 7.43 (dq, *J* = 10.2, 3.0 Hz, 2H), 7.37–7.28 (m, 3H), 6.96 (d, *J* = 7.6 Hz, 1H), 6.36 (d, *J* = 21.1 Hz, 1H), 4.61 (d, *J* = 40.0 Hz, 2H), 4.48 (d, *J* = 70.4 Hz, 2H), 4.16 (d, *J* = 7.0 Hz, 3H), 3.66 (t, *J* = 6.3 Hz, 2H), 2.30 (t, *J* = 6.8 Hz, 2H), 1.80–1.74 (m, 4H), 1.48 (d, *J* = 34.2 Hz, 9H). ^13^C NMR (151 MHz, CDCl_3_) δ 164.2, 163.9, 158.4, 158.3, 156.0, 153.4, 147.4, 141.0, 140.8, 140.5, 136.9, 136.8, 132.2, 128.9, 128.7, 128.1, 127.4, 127.2, 122.0, 121.7, 117.7, 103.6, 101.3, 80.9, 80.7, 75.4, 65.7, 62.1, 51.4, 51.3, 51.1, 50.4, 31.9, 28.6, 28.5, 24.7, 24.7, 19.9.

##### Tert-butyl([1,1′-biphenyl]-4-ylmethyl)((8-(7-hydroxyhept-1-yn-1-yl)-9-methoxy-4-oxo-4*H*-pyrido [1,2-a]pyrimidin-2-yl)methyl)carbamate (**64**)

The title compound was obtained as a light yellow oil (193 mg, 0.331 mmol, 66.3%). ^1^H NMR (600 MHz, CDCl3) δ 8.66 (t, *J* = 7.2 Hz, 1H), 7.58–7.49 (m, 4H), 7.45–7.38 (m, 2H), 7.32 (dt, *J* = 19.6, 7.9 Hz, 3H), 6.97–6.91 (m, 1H), 6.35 (d, *J* = 21.7 Hz, 1H), 4.60 (d, *J* = 41.3 Hz, 2H), 4.46 (d, *J* = 71.0 Hz, 2H), 4.14 (d, *J* = 6.4 Hz, 3H), 3.65 (t, *J* = 6.4 Hz, 2H), 2.53 (t, *J* = 7.1 Hz, 2H), 1.68 (p, *J* = 7.2 Hz, 2H), 1.65–1.58 (m, 2H), 1.57–1.54 (m, 2H), 1.48 (d, *J* = 33.5 Hz, 10H). ^13^C NMR (151 MHz, CDCl3) δ 164.2, 164.0, 158.4, 158.3, 155.9, 153.4, 147.5, 140.8, 140.7, 140.4, 136.8, 136.7, 128.8, 128.8, 128.6, 128.0, 127.4, 127.3, 127.1, 121.9, 121.5, 117.7, 117.6, 103.7, 103.6, 101.2, 80.8, 80.6, 75.1, 65.5, 62.6, 62.6, 61.9, 51.4, 51.3, 51.0, 50.3, 32.2, 32.2, 28.1, 25.2, 20.0.

#### 3.2.8. General Procedure for Catalytic Hydrogenation (**65**–**69**)

Compounds **60**–**64** were dissolved in absolute EtOH (3 mL) and the solution was bubbled with argon for 5 min. Pd/C (10% loading, 50 mg) was added to the solution. The flask was sealed and flushed with argon thrice, then hydrogen. The suspension was stirred for 2–4 h under a hydrogen balloon and then filtered through celite to yield pure 65–69 with no further purification necessary.*Peaks in ^1^H and ^13^C NMR are broad and split due to their bulky structure and Boc rotamers. Compounds were sensitive to high temperature, preventing high temperature NMR.

##### Tert-butyl ([1,1′-biphenyl]-4-ylmethyl)((8-(3-hydroxypropyl)-9-methoxy-4-oxo-4*H*-pyrido [1,2-a]pyrimidin-2-yl)methyl)carbamate (**65**)

The reaction was performed in a Parr bomb under 200 PSI hydrogen. The title compound was obtained as a white amorphous solid (130 mg, 0.245 mmol, 82.3%). ^1^H NMR (600 MHz, CDCl_3_) δ 8.69 (dd, *J* = 11.5, 7.1 Hz, 1H), 7.52–7.43 (m, 5H), 7.38–7.32 (m, 2H), 7.30–7.21 (m, 3H), 6.93 (dd, *J* = 12.9, 7.3 Hz, 1H), 6.27 (d, *J* = 19.8 Hz, 1H), 4.55 (d, *J* = 44.5 Hz, 2H), 4.38 (d, *J* = 70.7 Hz, 2H), 4.00 (d, *J* = 4.4 Hz, 3H), 3.59 (q, *J* = 6.4 Hz, 2H), 2.80 (q, *J* = 7.1, 6.6 Hz, 2H), 1.86–1.77 (m, 2H), 1.41 (d, *J* = 34.3 Hz, 9H). ^13^C NMR (151 MHz, CDCl_3_) δ 158.1, 158.0, 155.4, 148.8, 146.7, 140.9, 140.7, 140.3, 140.2, 139.9, 136.3, 136.2, 131.6, 131.6, 128.3, 128.0, 127.5, 126.8, 126.8, 126.6, 121.7, 116.6, 116.5, 99.7, 80.2, 80.0, 61.4, 60.9, 50.9, 50.8, 50.5, 49.8, 45.4, 31.6, 28.0, 27.9, 25.7, 8.2.

##### Tert-butyl([1,1′-biphenyl]-4-ylmethyl)((8-(4-hydroxybutyl)-9-methoxy-4-oxo-4*H*-pyrido [1,2-a]pyrimidin-2-yl)methyl)carbamate (**66**)

The title compound was obtained as a light yellow oil (167 mg, 0.307 mmol, 92.3%). ^1^H NMR (600 MHz, CDCl_3_) δ 8.76 (dd, *J* = 10.5, 7.2 Hz, 1H), 7.59–7.51 (m, 4H), 7.45–7.39 (m, 2H), 7.37–7.27 (m, 3H), 6.97 (dd, *J* = 11.9, 7.3 Hz, 1H), 6.34 (d, *J* = 20.6 Hz, 1H), 4.63 (d, *J* = 45.6 Hz, 2H), 4.46 (d, *J* = 73.3 Hz, 2H), 4.06 (d, *J* = 4.6 Hz, 3H), 3.68 (t, *J* = 6.3 Hz, 2H), 2.81–2.75 (m, 2H), 1.77–1.69 (m, 2H), 1.67–1.59 (m, 2H), 1.48 (d, *J* = 34.1 Hz, 9H). ^13^C NMR (151 MHz, CDCl_3_) δ 164.2, 163.9, 158.7, 158.6, 156.0, 149.3, 147.3, 141.8, 141.6, 140.9, 140.8, 140.5, 136.9, 136.8, 132.2, 132.1, 128.9, 128.6, 128.0, 127.4, 127.4, 127.1, 122.2, 117.0, 116.9, 100.2, 80.8, 80.6, 62.4, 61.9, 51.5, 51.3, 51.0, 50.3, 36.9, 32.3, 29.5, 28.5, 28.5, 25.8.

##### Tert-butyl([1,1′-biphenyl]-4-ylmethyl)((8-(5-hydroxypentyl)-9-methoxy-4-oxo-4*H*-pyrido [1,2-a]pyrimidin-2-yl)methyl)carbamate (**67**)

The title compound was obtained as a light yellow oil (140 mg, 0.250 mmol, quant.). ^1^H NMR (600 MHz, CDCl_3_) δ 8.75 (dd, *J* = 9.6, 7.2 Hz, 1H), 7.59–7.50 (m, 4H), 7.45–7.38 (m, 2H), 7.37–7.27 (m, 3H), 6.96 (dd, *J* = 10.5, 7.2 Hz, 1H), 6.34 (d, *J* = 20.6 Hz, 1H), 4.62 (d, *J* = 45.7 Hz, 2H), 4.46 (d, *J* = 73.5 Hz, 2H), 4.05 (d, *J* = 4.2 Hz, 3H), 3.63 (t, *J* = 6.5 Hz, 2H), 2.76 (td, *J* = 7.8, 4.1 Hz, 2H), 1.70–1.63 (m, 2H), 1.60 (q, *J* = 7.1 Hz, 2H), 1.48 (d, *J* = 34.0 Hz, 9H), 1.46 (m, 2H). ^13^C NMR (151 MHz, CDCl_3_) δ 164.1, 163.9, 158.7, 158.6, 156.0, 149.2, 147.3, 142.0, 141.9, 140.9, 140.8, 140.4, 136.9, 136.8, 132.2, 132.1, 128.9, 128.6, 128.6, 128.0, 127.4, 127.4, 127.1, 122.2, 117.1, 117.0, 100.1, 80.8, 80.6, 62.6, 61.9, 51.5, 51.3, 51.0, 50.3, 32.5, 29.8, 29.3, 28.5, 28.5, 25.7, 18.5.

##### Tert-butyl([1,1′-biphenyl]-4-ylmethyl)((8-(6-hydroxyhexyl)-9-methoxy-4-oxo-4*H*-pyrido [1,2-a]pyrimidin-2-yl)methyl)carbamate (**68**)

The title compound was obtained as a light yellow oil (82 mg, 0.143 mmol, 71.9%). ^1^H NMR (600 MHz, CDCl_3_) δ 8.81–8.75 (m, 1H), 7.62–7.53 (m, 4H), 7.45 (td, *J* = 7.9, 3.1 Hz, 2H), 7.41–7.31 (m, 3H), 6.99 (t, *J* = 9.5, 7.4 Hz, 1H), 6.36 (d, *J* = 20.2 Hz, 1H), 4.65 (d, *J* = 44.9 Hz, 2H), 4.50 (d, *J* = 74.5 Hz, 2H), 4.07 (d, *J* = 3.9 Hz, 3H), 3.64 (t, *J* = 6.6 Hz, 2H), 2.82–2.73 (m, 2H), 1.72–1.63 (m, 2H), 1.62–1.55 (m, 4H), 1.51 (d, *J* = 34.1 Hz, 9H), 1.45–1.43 (m, 2H). ^13^C NMR (151 MHz, CDCl_3_) δ 164.1, 163.8, 158.6, 156.0, 149.1, 147.3, 140.9, 140.8, 140.5, 136.9, 136.8, 136.6, 128.9, 128.6, 128.1, 127.4, 127.2, 122.2, 117.1, 109.0, 100.1, 80.8, 80.6, 62.9, 61.9, 51.5, 51.3, 51.1, 50.4, 32.6, 29.8, 29.5, 29.3, 28.5, 28.5, 25.6.

##### Tert-butyl([1,1′-biphenyl]-4-ylmethyl)((8-(7-hydroxyheptyl)-9-methoxy-4-oxo-4*H*-pyrido [1,2-a]pyrimidin-2-yl)methyl)carbamate (**69**)

The title compound was obtained as a light yellow oil (146 mg, 0.250 mmol, quant.). ^1^H NMR (600 MHz, CDCl_3_) δ 8.79 (t, *J* = 8.8, 7.5 Hz, 1H), 7.62–7.52 (m, 4H), 7.45 (dt, *J* = 7.8, 4.1 Hz, 2H), 7.41–7.29 (m, 3H), 7.00 (t, *J* = 7.7 Hz, 1H), 6.36 (d, *J* = 21.1 Hz, 1H), 4.65 (d, *J* = 42.5 Hz, 2H), 4.53 (d, *J* = 75.9 Hz, 2H), 4.08 (s, 3H), 3.65 (t, *J* = 6.6 Hz, 3H), 2.81–2.74 (m, 2H), 1.70–1.61 (m, 2H), 1.61–1.55 (m, 2H), 1.51 (d, *J* = 33.6 Hz, 9H), 1.40 (t, *J* = 6.2 Hz, 9H). ^13^C NMR (151 MHz, CDCl_3_) δ 163.9, 163.6, 158.4, 155.9, 149.0, 147.1, 142.7, 140.9, 140.8, 140.5, 136.9, 136.8, 128.9, 128.6, 128.1, 127.4, 127.4, 127.2, 122.2, 117.3, 100.1, 80.8, 80.6, 63.1, 63.0, 62.0, 51.4, 51.2, 50.4, 32.8, 29.8, 29.5, 29.4, 29.2, 28.5, 28.5, 25.7.

#### 3.2.9. General Procedure for Bromination (**70**–**74**)

Compounds **65**–**69** were dissolved in anhydrous DCM (3 mL) with triphenyl phosphine (1.2 equiv.) and carbon tetrabromide (1.2 equiv.). The solution was stirred at room temperature under argon for 5 h. Upon completion, the reaction mixture was concentrated directly onto silica and purified on RP ACC (0–100% EtOAc in hexanes) to yield compounds **70**–**74**. * Peaks in ^1^H and ^13^C NMR are broad and split due to their bulky structure and Boc rotamers. Compounds were sensitive to high temperature, preventing high temperature NMR.

##### Tert-butyl([1,1′-biphenyl]-4-ylmethyl)((8-(3-bromopropyl)-9-methoxy-4-oxo-4*H*-pyrido [1,2-a]pyrimidin-2-yl)methyl)carbamate (**70**)

The title compound was obtained as a clear oil (84 mg, 0.142 mmol, 57.9%). ^1^H NMR (600 MHz, CDCl_3_) δ 8.78 (dd, *J* = 10.4, 7.2 Hz, 1H), 7.61–7.51 (m, 4H), 7.43 (dt, *J* = 7.8, 5.1 Hz, 2H), 7.37–7.27 (m, 3H), 6.98 (dd, *J* = 11.7, 7.3 Hz, 1H), 6.36 (d, *J* = 19.9 Hz, 1H), 4.63 (d, *J* = 45.9 Hz, 2H), 4.46 (d, *J* = 72.5 Hz, 2H), 4.09 (d, *J* = 4.4 Hz, 3H), 3.44 (dt, *J* = 8.2, 4.0 Hz, 2H), 2.95–2.88 (m, 2H), 2.23–2.15 (m, 2H), 1.49 (d, *J* = 34.5 Hz, 9H). ^13^C NMR (151 MHz, CDCl_3_) δ 164.2, 163.9, 158.6, 158.5, 155.9, 155.8, 149.6, 147.3, 140.9, 140.8, 140.5, 139.9, 139.7, 136.9, 136.8, 128.9, 128.9, 128.6, 128.0, 127.4, 127.4, 127.1, 122.3, 116.9, 116.8, 100.5, 100.4, 80.8, 80.6, 62.0, 61.9, 51.5, 51.3, 51.0, 50.4, 44.1, 32.7, 32.2, 32.1, 28.6, 28.5, 28.5.

##### Tert-butyl([1,1′-biphenyl]-4-ylmethyl)((8-(4-bromobutyl)-9-methoxy-4-oxo-4*H*-pyrido [1,2-a]pyrimidin-2-yl)methyl)carbamate (**71**)

The title compound was obtained as a clear oil (80 mg, 0.132 mmol, 53.0%). ^1^H NMR (600 MHz, CDCl_3_) δ 8.78 (dd, *J* = 9.8, 7.1 Hz, 1H), 7.60–7.51 (m, 4H), 7.43 (td, *J* = 7.8, 3.1 Hz, 2H), 7.38–7.27 (m, 3H), 6.96 (dd, *J* = 11.4, 7.2 Hz, 1H), 6.35 (d, *J* = 20.5 Hz, 1H), 4.63 (d, *J* = 45.8 Hz, 2H), 4.47 (d, *J* = 73.5 Hz, 2H), 4.07 (d, *J* = 5.5 Hz, 3H), 3.45 (t, *J* = 6.5 Hz, 2H), 2.79 (td, *J* = 7.9, 4.2 Hz, 2H), 1.97–1.90 (m, 2H), 1.86–1.77 (m, 2H), 1.49 (d, *J* = 34.5 Hz, 10H). ^13^C NMR (151 MHz, CDCl_3_) δ 163.6, 163.4, 158.1, 158.0, 155.4, 148.8, 146.8, 140.3, 139.9, 136.3, 136.3, 128.3, 128.1, 127.5, 126.9, 126.8, 126.6, 123.8, 121.8, 116.2, 99.8, 99.7, 80.2, 80.0, 61.6, 61.4, 51.0, 50.8, 50.5, 49.8, 32.6, 31.6, 28.3, 28.0, 27.9, 27.3.

##### Tert-butyl([1,1′-biphenyl]-4-ylmethyl)((8-(5-bromopentyl)-9-methoxy-4-oxo-4*H*-pyrido [1,2-a]pyrimidin-2-yl)methyl)carbamate (**72**)

The title compound was obtained as a yellow oil (20 mg, 0.032 mmol, 18.0%). ^1^H NMR (600 MHz, CDCl_3_) δ 8.77 (dd, *J* = 9.7, 7.1 Hz, 1H), 7.59–7.51 (m, 4H), 7.47–7.40 (m, 2H), 7.38–7.28 (m, 3H), 6.96 (dd, *J* = 10.5, 7.2 Hz, 1H), 6.35 (d, *J* = 20.1 Hz, 1H), 4.63 (d, *J* = 45.2 Hz, 2H), 4.48 (d, *J* = 74.2 Hz, 2H), 4.07 (d, *J* = 4.5 Hz, 3H), 3.41 (t, *J* = 6.7 Hz, 2H), 2.77 (td, *J* = 7.8, 4.1 Hz, 2H), 1.91 (p, *J* = 7.0 Hz, 2H), 1.67 (p, *J* = 8.8, 7.9 Hz, 2H), 1.57–1.53 (m, 2H), 1.49 (d, *J* = 34.6 Hz, 9H). ^13^C NMR (151 MHz, CDCl_3_) δ 164.1, 158.6, 156.0, 149.3, 147.3, 141.0, 140.8, 140.5, 136.9, 136.8, 128.9, 128.6, 128.1, 127.4, 127.4, 127.2, 122.3, 117.0, 100.2, 80.8, 80.6, 62.0, 51.5, 51.3, 51.1, 50.4, 44.9, 33.6, 32.5, 29.7, 28.7, 28.6, 28.5, 28.0.

##### Tert-butyl([1,1′-biphenyl]-4-ylmethyl)((8-(6-bromohexyl)-9-methoxy-4-oxo-4*H*-pyrido [1,2-a]pyrimidin-2-yl)methyl)carbamate (**73**)

The title compound was obtained as a clear oil (45 mg, 0.071 mmol, 49.5%). ^1^H NMR (600 MHz, CDCl_3_) δ 8.77 (dd, *J* = 9.8, 7.1 Hz, 1H), 7.61–7.51 (m, 4H), 7.43 (td, *J* = 7.7, 3.1 Hz, 2H), 7.38–7.28 (m, 3H), 6.96 (dd, *J* = 10.8, 7.3 Hz, 1H), 6.35 (d, *J* = 20.4 Hz, 1H), 4.63 (d, *J* = 45.7 Hz, 2H), 4.47 (d, *J* = 74.4 Hz, 2H), 4.06 (d, *J* = 4.8 Hz, 3H), 3.40 (t, *J* = 6.7 Hz, 2H), 2.76 (td, *J* = 7.9, 4.0 Hz, 2H), 1.86 (p, *J* = 6.9 Hz, 2H), 1.66 (p, *J* = 8.2, 7.4 Hz, 2H), 1.53–1.46 (m, 2H), 1.49 (d, *J* = 34.5 Hz, 9H), 1.44–1.36 (m, 2H). ^13^C NMR (151 MHz, CDCl_3_) δ 164.1, 163.9, 158.7, 158.6, 156.0, 149.2, 147.3, 141.9, 140.9, 140.8, 140.5, 136.9, 136.9, 128.9, 128.9, 128.6, 128.1, 127.4, 127.4, 127.2, 122.2, 117.0, 100.2, 100.1, 80.8, 80.6, 61.9, 51.5, 51.3, 51.1, 50.4, 33.9, 32.6, 29.8, 29.3, 28.6, 28.6, 28.5, 28.0, 28.0.

##### Tert-butyl([1,1′-biphenyl]-4-ylmethyl)((8-(7-bromoheptyl)-9-methoxy-4-oxo-4*H*-pyrido [1,2-a]pyrimidin-2-yl)methyl)carbamate (**74**)

The title compound was obtained as a clear oil (70 mg, 0.108 mmol, 50.7%). ^1^H NMR (600 MHz, CDCl_3_) δ 8.77 (dd, *J* = 9.9, 7.1 Hz, 1H), 7.61–7.50 (m, 4H), 7.43 (td, *J* = 7.9, 3.2 Hz, 2H), 7.39–7.27 (m, 3H), 6.96 (dd, *J* = 11.0, 7.2 Hz, 1H), 6.34 (d, *J* = 20.7 Hz, 1H), 4.63 (d, *J* = 46.0 Hz, 2H), 4.47 (d, *J* = 74.1 Hz, 2H), 4.06 (d, *J* = 5.1 Hz, 3H), 3.40 (t, *J* = 6.8 Hz, 2H), 2.75 (td, *J* = 8.2, 4.2 Hz, 2H), 1.85 (p, *J* = 6.9 Hz, 2H), 1.64 (p, *J* = 7.7 Hz, 2H), 1.49 (d, *J* = 33.9 Hz, 9H), 1.44–1.41 (m, 2H), 1.41–1.33 (m, 4H). ^13^C NMR (151 MHz, CDCl_3_) δ 164.1, 163.9, 158.7, 158.6, 155.9, 149.2, 147.3, 142.1, 142.0, 140.9, 140.8, 140.5, 140.4, 136.9, 136.8, 128.9, 128.6, 128.0, 127.4, 127.4, 127.1, 122.2, 117.0, 117.0, 100.1, 100.1, 80.8, 80.5, 61.9, 51.5, 51.3, 51.0, 50.3, 34.0, 32.8, 29.8, 29.4, 29.3, 28.6, 28.5, 28.5, 28.0.

#### 3.2.10. General Procedure for Deprotection (**75**–**79**)

Compounds 70–74 were dissolved in anhydrous DCM (2 mL) under argon. 1 mL of boron tribromide (1 M in DCM) was added dropwise to the stirring solution. The reaction was stirred at room temperature for 2–5 h. Upon complete deprotection, the reaction was diluted with 5 mL water and extracted with DCM (3 × 3mL). The combined organic extracts (suspensions) were concentrated to yield the product (LC-MS). The crude product was used as is with no further purification.

2-((([1,1′-biphenyl]-4-ylmethyl)amino)methyl)-8-(3-bromopropyl)-9-hydroxy-4*H*-pyrido [1,2-a]pyrimidin-4-one (**75**)

The title compound was obtained as a clear oil (64 mg, 0.135 mmol, quant.) MS (ES+) *m*/*z* calc. for C_25_H_24_BrN_3_O_2_ [M + H]^+^: 478.11; found: 478.1, 480.1.

2-((([1,1′-biphenyl]-4-ylmethyl)amino)methyl)-8-(4-bromopropyl)-9-hydroxy-4*H*-pyrido [1,2-a]pyrimidin-4-one (**76**)

The title compound was obtained as a clear oil (49 mg, 0.100 mmol, quant.). MS (ES+) *m*/*z* calc. for C_26_H_26_BrN_3_O_2_ [M + H]^+^: 492.13; found: 492.1, 494.1.

2-((([1,1′-biphenyl]-4-ylmethyl)amino)methyl)-8-(5-bromopentyl)-9-hydroxy-4*H*-pyrido [1,2-a]pyrimidin-4-one (**77**)

The title compound was obtained as a clear oil (16 mg, 0.032 mmol, quant.). MS (ES+) *m*/*z* calc. for C_27_H_28_BrN_3_O_2_ [M + H]^+^: 506.14; found: 506.1, 508.1.

2-((([1,1′-biphenyl]-4-ylmethyl)amino)methyl)-8-(6-bromohexyl)-9-hydroxy-4*H*-pyrido [1,2-a]pyrimidin-4-one (**78**)

The title compound was obtained as a clear oil (37 mg, 0.071 mmol, quant.). MS (ES+) *m*/*z* calc. for C_28_H_30_BrN_3_O_2_ [M + H]^+^: 520.16; found: 520.2, 522.2.

2-((([1,1′-biphenyl]-4-ylmethyl)amino)methyl)-8-(7-bromoheptyl)-9-hydroxy-4*H*-pyrido [1,2-a]pyrimidin-4-one (**79**)

The title compound was obtained as a clear oil (58 mg, 0.108 mmol, quant.). MS (ES+) *m*/*z* calc. for C_29_H_32_BrN_3_O_2_ [M + H]^+^: 534.17; found: 534.1, 536.1.

#### 3.2.11. General Procedure for Final Compounds (**80**–**84**)

Sodium methanethiosulfonate (1.5 equiv.) was added to a solution of compounds **70**–**79** in 3 mL EtOH, and the reaction mixture was refluxed for 24 h. After consumption of the starting material, the crude reaction was purified directly by RP ACC (0–100% ACN in 0.1% formic acid) to yield the final catch and anchor compounds **80**–**84**.

##### *S*-(6-(2-((([1,1′-biphenyl]-4-ylmethyl)amino)methyl)-9-hydroxy-4-oxo-4*H*-pyrido [1,2-a]pyrimidin-8-yl)pentyl) methanesulfonothioate (**80**)

The title compound was obtained as a light yellow solid (12 mg, 0.108 mmol, 22.6%). ^1^H NMR (600 MHz, DMSO-d_6_) δ 8.43 (d, *J* = 7.2 Hz, 1H), 8.19 (t, *J* = 1.7 Hz, 1H), 7.67–7.63 (m, 5H), 7.55–7.51 (m, 2H), 7.48–7.43 (m, 3H), 7.39–7.33 (m, 2H), 7.24 (d, *J* = 7.2 Hz, 1H), 6.39 (s, 1H), 4.03 (s, 2H), 3.99 (s, 2H), 3.53 (s, 3H), 3.26 (t, *J* = 7.3 Hz, 2H), 2.85 (dd, *J* = 8.3, 6.7 Hz, 2H), 2.12–2.04 (m, 2H). ^13^C NMR (151 MHz, DMSO-d_6_) δ 163.5, 157.0, 146.6, 143.5, 139.8, 139.6, 129.7, 129.1, 128.9, 128.9, 127.5, 126.6, 118.3, 116.9, 100.0, 55.8, 54.9, 50.2, 35.1, 28.1, 27.6. HRMS (ES+) *m*/*z* calc. for C_26_H_27_N_3_O_4_S_2_ [M + H]⁺: 510.1516; found: 510.1536.

##### *S*-(6-(2-((([1,1′-biphenyl]-4-ylmethyl)amino)methyl)-9-hydroxy-4-oxo-4*H*-pyrido [1,2-a]pyrimidin-8-yl)pentyl) methanesulfonothioate (**81**)

The title compound was obtained as a light yellow solid (9 mg, 0.016 mmol, 20.2%). ^1^H NMR (600 MHz, DMSO-d_6_) δ 8.43 (d, *J* = 7.2 Hz, 1H), 7.66–7.63 (m, 4H), 7.52–7.48 (m, 2H), 7.48–7.44 (m, 2H), 7.38–7.34 (m, 1H), 7.22 (d, *J* = 7.3 Hz, 1H), 6.39 (s, 1H), 3.94 (s, 2H), 3.90 (s, 2H), 3.50 (s, 3H), 3.25 (t, *J* = 6.8 Hz, 2H), 2.76 (t, *J* = 7.0 Hz, 2H), 1.80–1.70 (m, 4H). ^13^C NMR (151 MHz, DMSO-d_6_) δ 163.7, 157.3, 146.5, 143.6, 139.9, 130.4, 130.0, 129.2, 127.7, 126.8, 126.8, 118.7, 117.0, 100.1, 55.9, 51.1, 50.3, 35.4, 28.6, 28.2, 27.1.

##### *S*-(6-(2-((([1,1′-biphenyl]-4-ylmethyl)amino)methyl)-9-hydroxy-4-oxo-4*H*-pyrido [1,2-a]pyrimidin-8-yl)pentyl) methanesulfonothioate (**82**)

The title compound obtained as a white solid (4.5 mg, 0.008 mmol, 26%). ^1^H NMR (600 MHz, CDCl_3_) δ 8.45 (d, *J* = 7.1 Hz, 1H), 7.54 (d, *J* = 2.0 Hz, 4H), 7.51–7.46 (m, 2H), 7.42–7.38 (m, 2H), 7.36–7.32 (m, 1H), 6.97 (d, *J* = 7.2 Hz, 1H), 6.20 (s, 1H), 4.28 (s, 2H), 4.11 (s, 2H), 3.31 (s, 3H), 3.16 (t, *J* = 7.4 Hz, 2H), 2.76 (t, *J* = 7.7 Hz, 2H), 1.82 (p, *J* = 7.5 Hz, 2H), 1.71–1.65 (m, 2H), 1.53–1.47 (m, 2H). ^13^C NMR (151 MHz, CDCl_3_) δ 167.8, 132.1, 131.1, 129.0, 127.9, 127.1, 66.4, 64.5, 50.8, 36.3, 29.4, 28.4, 25.3. *weak ^13^C NMR. HRMS (ES+) *m*/*z* calc. for C28H31N4O4S2 [M + H]⁺: 538.1829; found: 538.1834.

##### *S*-(6-(2-((([1,1′-biphenyl]-4-ylmethyl)amino)methyl)-9-hydroxy-4-oxo-4*H*-pyrido [1,2-a]pyrimidin-8-yl)hexyl) methanesulfonothioate (**83**)

The title compound was obtained as a fluffy yellow solid (17 mg, 0.031 mmol, 43.6%). ^1^H NMR (600 MHz, CDCl_3_) δ 8.42 (d, *J* = 7.2 Hz, 1H), 7.62–7.53 (m, 5H), 7.49–7.44 (m, 2H), 7.43–7.37 (m, 2H), 7.36–7.30 (m, 1H), 6.96 (d, *J* = 7.2 Hz, 1H), 6.16 (s, 1H), 4.39–4.33 (m, 2H), 4.15 (s, 2H), 3.31 (s, 3H), 3.19–3.10 (m, 2H), 2.81–2.68 (m, 2H), 1.87–1.70 (m, 2H), 1.62 (q, *J* = 7.6 Hz, 2H), 1.50–1.34 (m, 4H). ^13^C NMR (151 MHz, CDCl_3_) δ 157.5, 155.2, 147.1, 143.8, 142.6, 139.9, 132.7, 131.4, 131.2, 129.0, 128.0, 127.9, 127.1, 119.0, 117.4, 100.5, 55.4, 50.8, 50.4, 36.5, 29.5, 29.4, 28.8, 28.4, 28.3. HRMS (ES+) *m*/*z* calc. for C29H33N4O4S2 [M + H]⁺: 552.1986; found: 552.1992.

##### *S*-(6-(2-((([1,1′-biphenyl]-4-ylmethyl)amino)methyl)-9-hydroxy-4-oxo-4*H*-pyrido [1,2-a]pyrimidin-8-yl)heptyl) methanesulfonothioate (**84**)

The title compound was obtained as a fluffy yellow solid (11.2 mg, 0.02 mmol, 20.5%). HRMS (ES+) *m*/*z* calc. for C_30_H_35_N_4_O_4_S_2_ [M + H]⁺: 566.2142; found: 566.2144.

## 4. Conclusions

Starting from an in silico lead and by implementing medicinal chemistry wisdom, a potent new class of BoNT/A LC inhibitors was discovered containing a 9-hydroxy-4*H*-pyrido [1,2-a]pyrimidin-4-one (PPO) scaffold. A library of 43 compounds was synthesized and used to elucidate SARs; docking of the inhibitors provided an additional rationale. From this series, a number of compounds provided promising inhibition in a motor neuron cell assay for BoNT/A activity. Notwithstanding this progression, compound **27** displayed an optimum in vitro inhibition of BoNT/A LC and was selected as the foundation structure for catch and anchor inhibitor development. Using computer-aided and structure-based drug design, a series of preliminary targeted covalent inhibitors were designed and synthesized based on **27**. The *k*_inact_/*K*_i_ values were evaluated, and the covalent modification of BoNT/A LC Cys165 was confirmed via mass spectrometry. The trend in *k*_inact_/*K*_i_ values for the series highlighted the importance of optimizing linker length—a one-carbon alteration garnered a seven-fold difference in potency. In summary, small-molecule BoNT/A light inhibition using our “catch-anchor” approach was successfully obtained; however, these efforts are still a work in progress and will require further tuning of the covalent warhead’s reactivity profile to achieve in vivo examination.

## Figures and Tables

**Table 1:** IC_50_ values for the synthesized PPO library (*n* = 3).

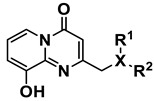			
Entry	X	NR^1^R^2^	IC_50_
15	N	*N*,*N*-dimethyl	>64
16	N	3-fluorobenzyl	0.6 ± 0.1
17	N	4-fluorobenzyl	1.2 ± 0.2
18	N	3-chlorobenzyl	1.7 ± 0.3
19	N	4-chlorobenzyl	1.1 ± 0.1
20	N	3-methoxybenzyl	0.49 ± 0.09
21	N	4-methoxybenzyl	1.9 ± 0.2
22	N	3-methylbenzyl	0.5 ± 0.1
23	N	4-methylbenzyl	1.4 ± 0.9
24	N	3-(methoxycarbonyl)benzyl	0.41 ± 0.08
25	N	4-(methoxycarbonyl)benzyl	>64
26	N	3-phenylbenzyl	0.6 ± 0.1
27	N	4-phenylbenzyl	0.3 ± 0.1
28	N	*N*-methyl-4-phenylbenzyl	12 ± 0.2
29	N	phenethyl	10 ± 1
30	O	benzyl	2.5 ± 0.3
31	O	2-fluorobenzyl	18 ± 2
32	O	3-fluorobenzyl	11 ± 1
33	O	4-fluorobenzyl	15 ± 1
34	O	2-chlorobenzyl	9 ± 2
35	O	3-chlorobenzyl	7.5 ± 0.5
36	O	4-chlorobenzyl	4.13 ± 0.09
37	O	3-hydroxybenzyl	26 ± 2
38	O	3-methylbenzyl	6.6 ± 0.5
39	O	4-methylbenzyl	9.1 ± 0.5
40	O	3-nitrobenzyl	9.7 ± 0.8
41	O	3,5-dimethoxybenzyl	5.2 ± 0.2
42	N	2-isoindolinyl	1.2 ± 0.1
43	N	2-(5-chloro)isoindolinyl	0.5 ± 0.2
44	N	2-(5-bromo)isoindolinyl	0.40 ± 0.02
45	N	2-(5-phenyl)isoindolinyl	0.7 ± 0.1
46	N	1-indolinyl	2.9 ± 0.1
47	N	1-(5-chloro)indolinyl	0.9 ± 0.6
48	N	1-(5-bromo)indolinyl	0.7 ± 0.1
49	N	1-(6-bromo)indolinyl	0.7 ± 0.05
50	N	1-(2-methyl)indolinyl	24 ± 14
51	N	1-(6-methoxy)indolinyl	>64
52	N	1-(methyl-5-carboxylate)indolinyl	1.2 ± 0.7
53	N	1-(methyl-6-carboxylate)indolinyl	1.1 ± 0.1
54	N	1-(6-carboxylate)indolinyl	1.2 ± 0.1
55	N	1-(5-phenyl)indolinyl	0.9 ± 0.3
56	N	1-(6-phenyl)indolinyl	0.6 ± 0.1

**Table 2:** Inhibition kinetics of catch and anchor PPO compounds. Values represent mean ± SD, *n* = 3.

Compound	n	*k*_inact_ (s^−1^)	*K*_i_ (µM)	*k*_inact_/*K*_i_ (M^−1^ s^−1^)
**80**	3	0.0016 ± 0.0002	3.9 ± 0.5	400 ± 60
**81**	4	0.0015 ± 0.0001	0.57 ± 0.02	2810 ± 150
**82**	5	0.0024 ± 0.0002	1.87 ± 0.11	1260 ± 130
**83**	6	0.0028 ± 0.0002	3.4 ± 0.5	820 ± 30
**84**	7	0.0038 ± 0.0008	32 ± 6	120 ± 30
**85**	--	--	--	514 ± 17

## Data Availability

The data presented in this study are available on request from the corresponding author.

## Supplementary Materials

The following supporting information can be downloaded at: https://www.mdpi.com/article/10.3390/ijms24054303/s1.
