# Supplementary material for: Transformation of a Metal Chelate into a “Catch and Anchor” Inhibitor of Botulinum A Protease
Source: Int J Mol Sci. 2023 Feb 21;24(5):4303. doi: 10.3390/ijms24054303 (PMC10001950; doi:10.3390/ijms24054303)
Supplement: Supplementary file 1 [file ijms-24-04303-s001.zip › ijms-2215502-supplementary.pdf]

Transformation of a Metal Chelate into a “Catch and Anchor” Inhibitor of Botulinum A Protease

Lucy Lin, Ealin N. Patel, Alexander L. Nielsen, Lewis D. Turner, William H. Tepp, Kong Nguyen, Sabine Pellett, Kim D. Janda*

*Corresponding author: kdjanda@scripps.edu

Figure S1. Cytotoxicity of the PPO compounds

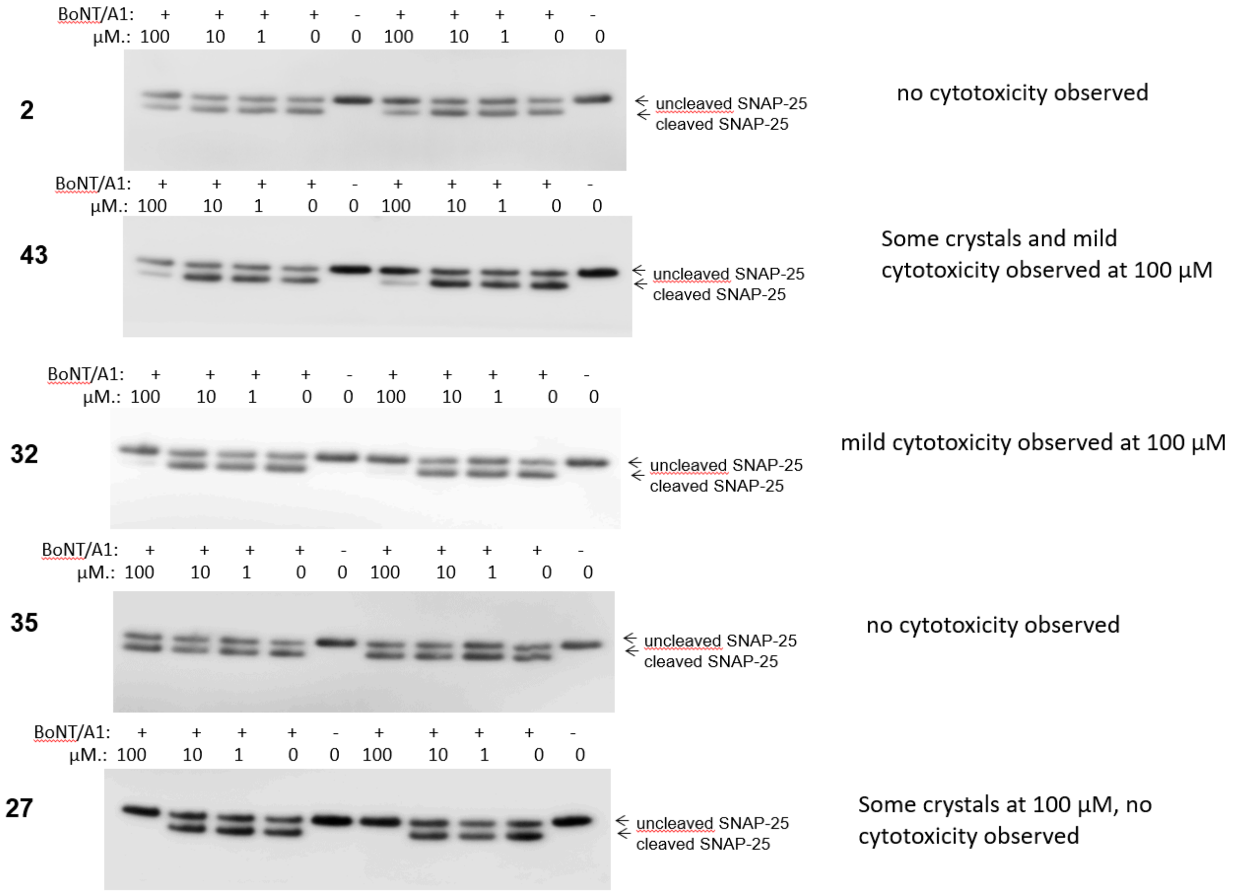

Figure S2. Activity Screen of Bifunctional inhibitors

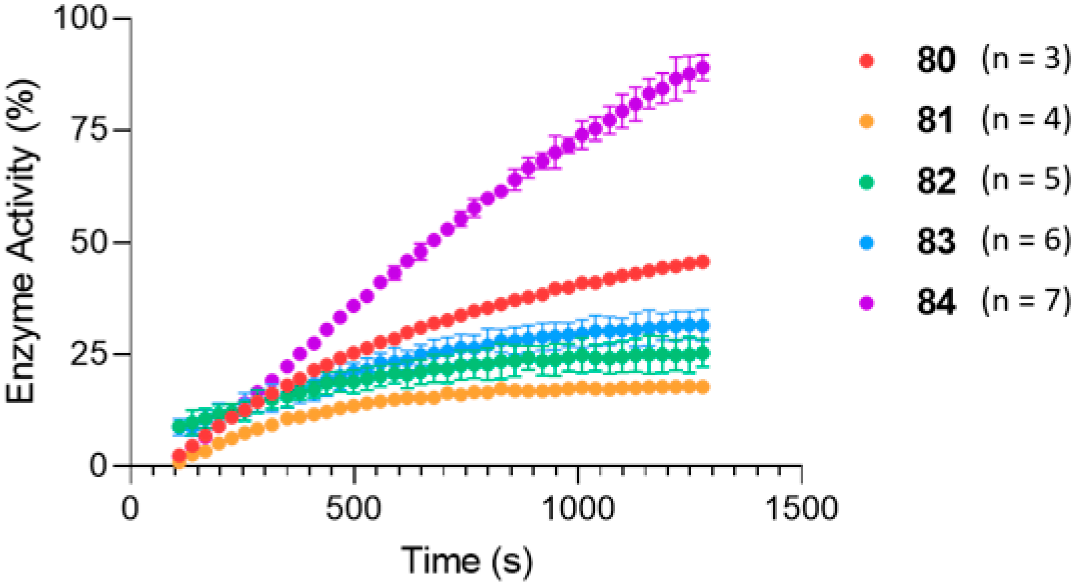

Screened at 4 µM against 10 nM BoNT/A LC, at 37 °C¶

^{1}H NMR of **2**

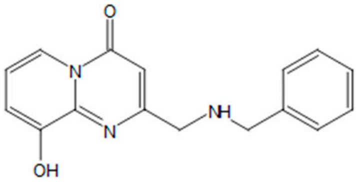

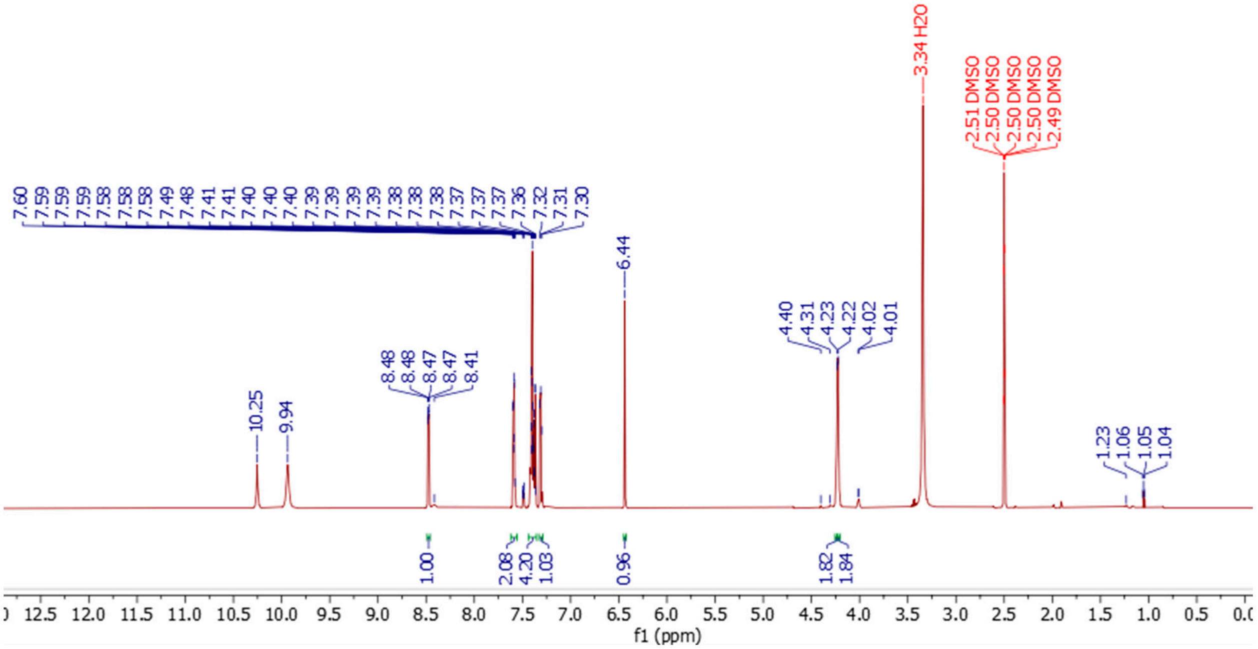

7.60
7.59
7.59
7.59
7.58
7.58
7.58
7.49
7.48
7.41
7.41
7.40
7.40
7.40
7.39
7.39
7.39
7.39
7.38
7.38
7.38
7.37
7.37
7.37
7.36
7.32
7.31
7.30
6.44
10.25
9.94
8.48
8.48
8.47
8.47
8.41
4.40
4.31
4.23
4.22
4.02
4.01
3.34 H2O
2.51 DMSO
2.50 DMSO
2.50 DMSO
2.50 DMSO
2.49 DMSO
1.23
1.06
1.05
1.04
1.00
2.08
4.20
1.03
0.96
1.82
1.84
12.5 12.0 11.5 11.0 10.5 10.0 9.5 9.0 8.5 8.0 7.5 7.0 6.5 6.0 5.5 5.0 4.5 4.0 3.5 3.0 2.5 2.0 1.5 1.0 0.5 0.0
f1 (ppm)

^{13}C NMR of **2**

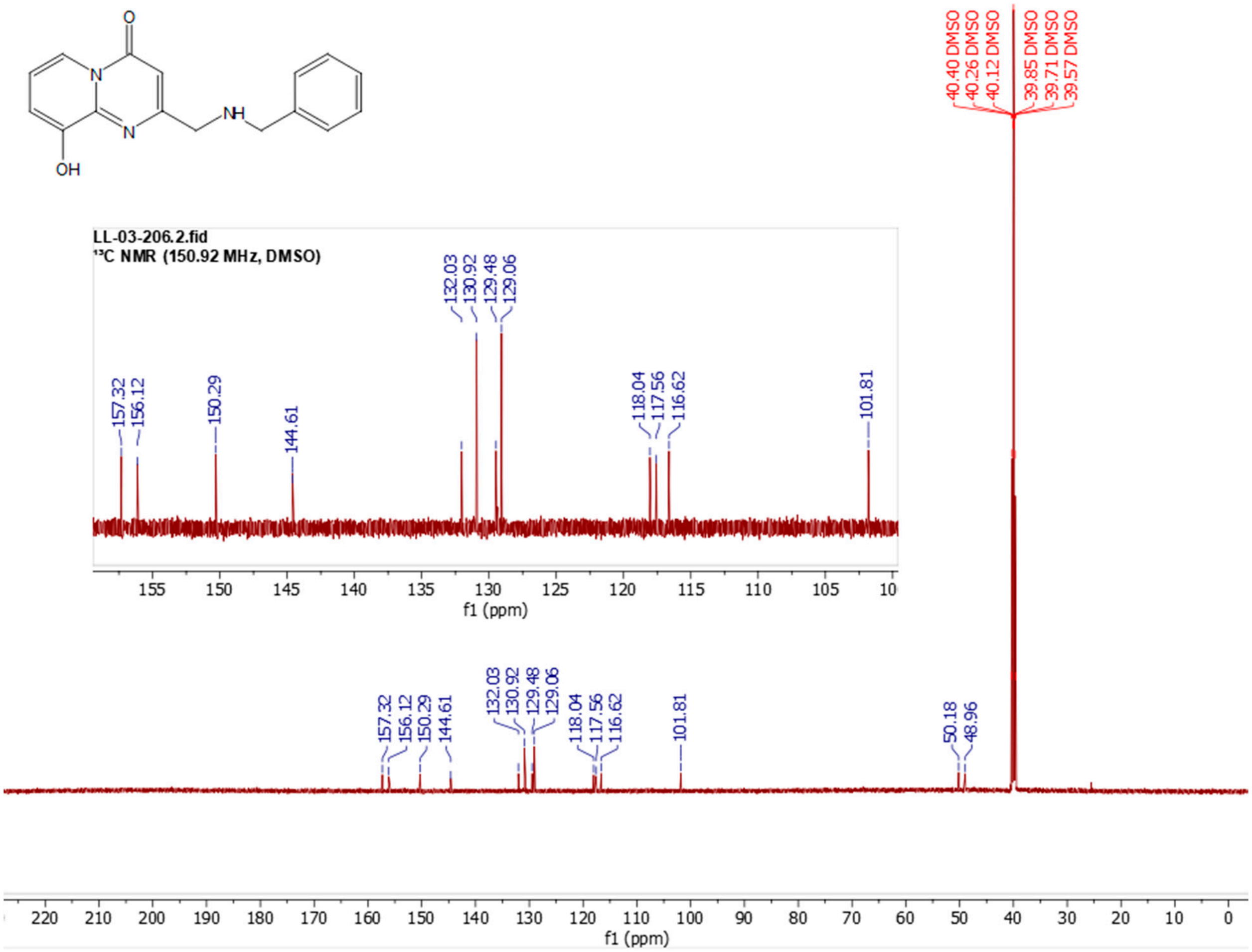

O
N
N
NH
OH
40.40 DMSO
40.26 DMSO
40.12 DMSO
39.85 DMSO
39.71 DMSO
39.57 DMSO
LL-03-206.2.fid
13C NMR (150.92 MHz, DMSO)
157.32
156.12
150.29
144.61
132.03
130.92
129.48
129.06
118.04
117.56
116.62
101.81
155 150 145 140 135 130 125 120 115 110 105 10
f1 (ppm)
50.18
48.96
220 210 200 190 180 170 160 150 140 130 120 110 100 90 80 70 60 50 40 30 20 10 0
f1 (ppm)

^{1}H NMR of **6**

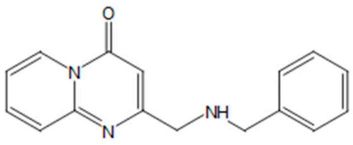

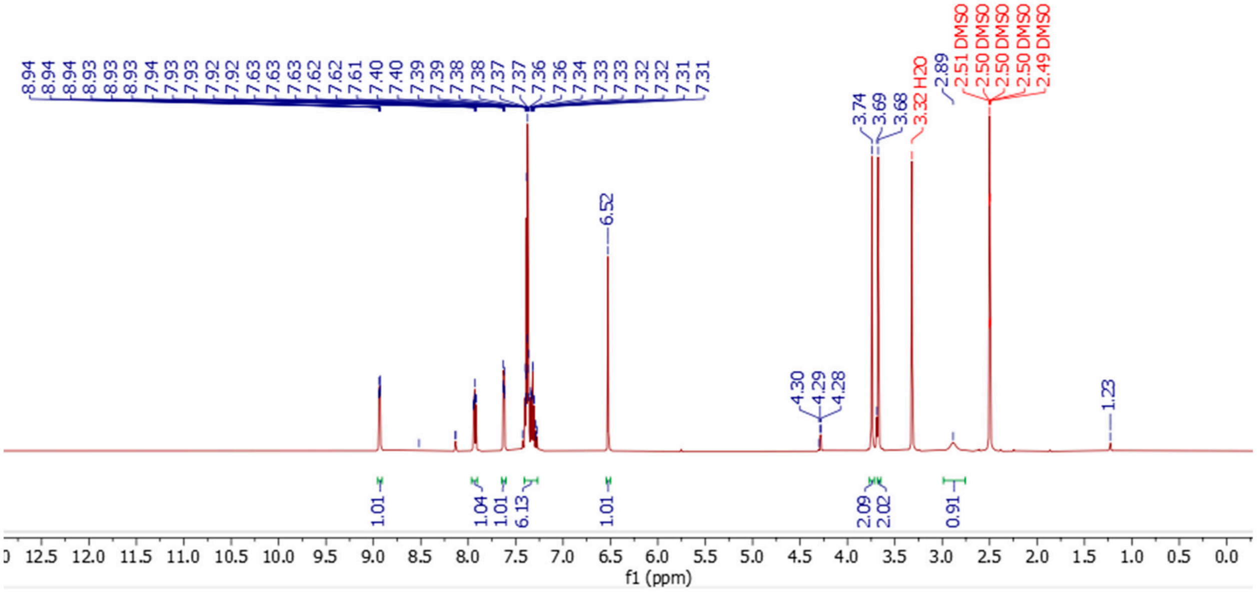

8.94
8.94
8.94
8.93
8.93
8.93
7.94
7.93
7.93
7.92
7.92
7.63
7.63
7.63
7.62
7.62
7.61
7.40
7.40
7.39
7.39
7.38
7.38
7.37
7.37
7.36
7.36
7.34
7.33
7.33
7.32
7.32
7.31
7.31
6.52
4.30
4.29
4.28
3.74
3.69
3.68
3.32 H2O
2.89
2.51 DMSO
2.50 DMSO
2.50 DMSO
2.50 DMSO
2.49 DMSO
1.23
1.01
1.04
1.01
6.13
1.01
2.09
2.02
0.91
f1 (ppm)

^{13}C NMR of **6**

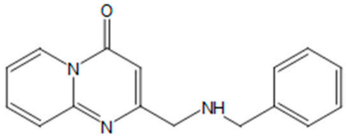
O
N
N
NH

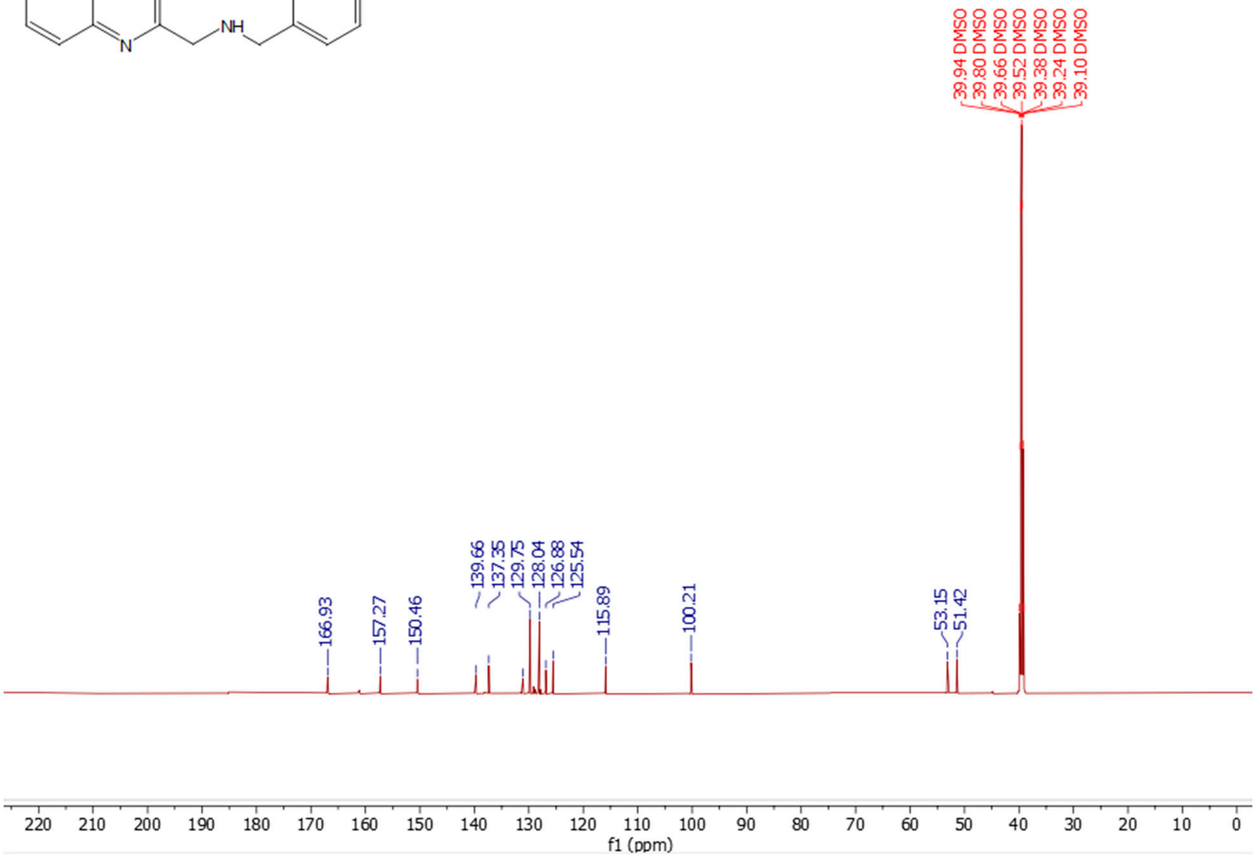
39.94 DMSO
39.80 DMSO
39.66 DMSO
39.52 DMSO
39.38 DMSO
39.24 DMSO
39.10 DMSO
166.93
157.27
150.46
139.66
137.35
129.75
128.04
126.88
125.54
115.89
100.21
53.15
51.42
220 210 200 190 180 170 160 150 140 130 120 110 100 90 80 70 60 50 40 30 20 10 0
f1 (ppm)

^{1}H NMR of **11**

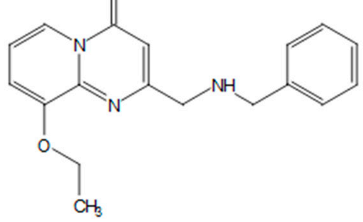

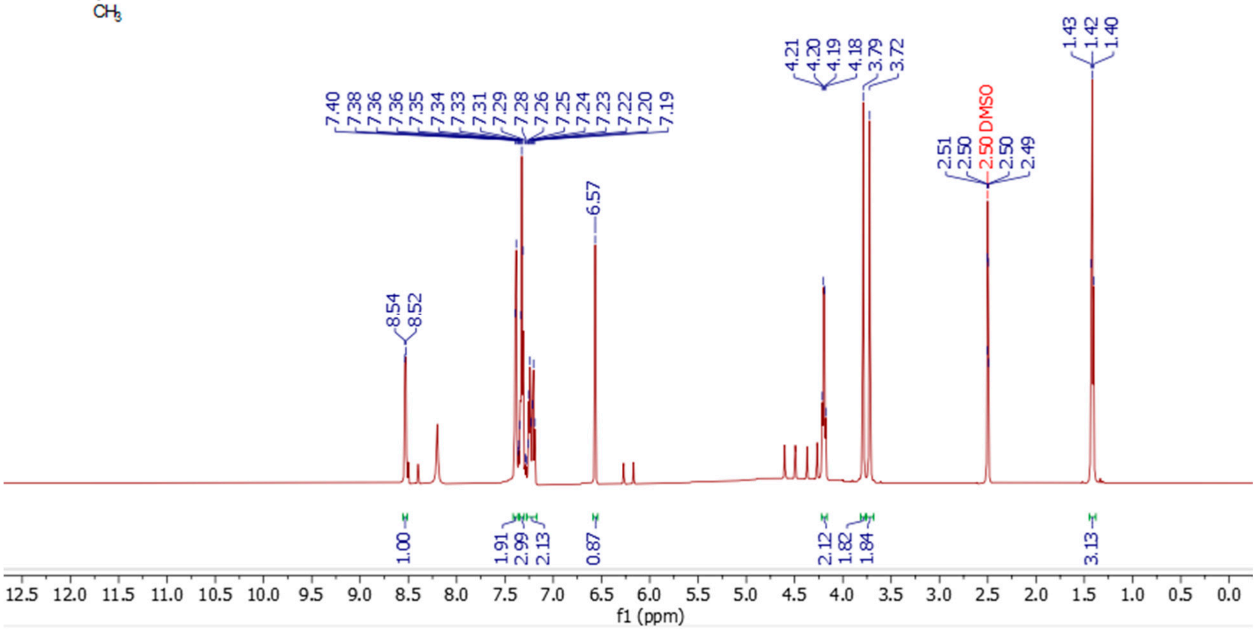

7.40 7.38 7.36 7.36 7.35 7.34 7.33 7.31 7.29 7.28 7.26 7.25 7.24 7.23 7.22 7.20 7.19
6.57
8.54 8.52
4.21 4.20 4.19 4.18 3.79 3.72
2.51 2.50 2.50 DMSO 2.50 2.49
1.43 1.42 1.40
1.00
1.91 2.99 2.13
0.87
2.12 1.82 1.84
3.13
12.5 12.0 11.5 11.0 10.5 10.0 9.5 9.0 8.5 8.0 7.5 7.0 6.5 6.0 5.5 5.0 4.5 4.0 3.5 3.0 2.5 2.0 1.5 1.0 0.5 0.0
f1 (ppm)

^{13}CNMR of **11**

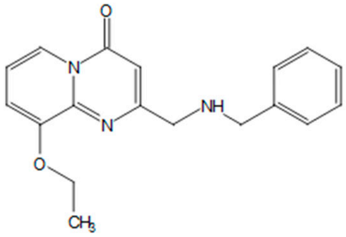

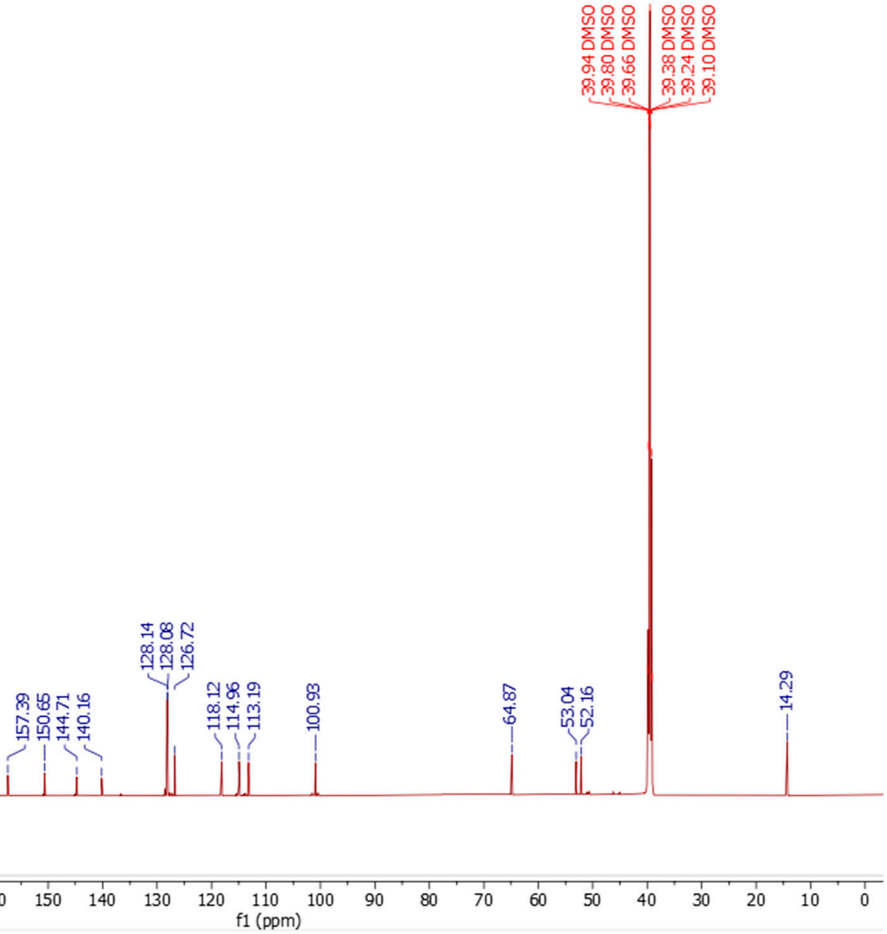
39.94 DMSO
39.80 DMSO
39.66 DMSO
39.38 DMSO
39.24 DMSO
39.10 DMSO
165.38
157.39
150.65
144.71
140.16
128.14
128.08
126.72
118.12
114.96
113.19
100.93
64.87
53.04
52.16
14.29
220 210 200 190 180 170 160 150 140 130 120 110 100 90 80 70 60 50 40 30 20 10 0
f1 (ppm)

^{1}H NMR of **13**

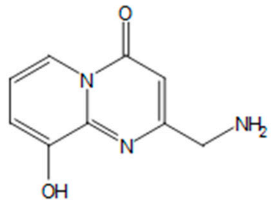
O
N
N
NH2
OH

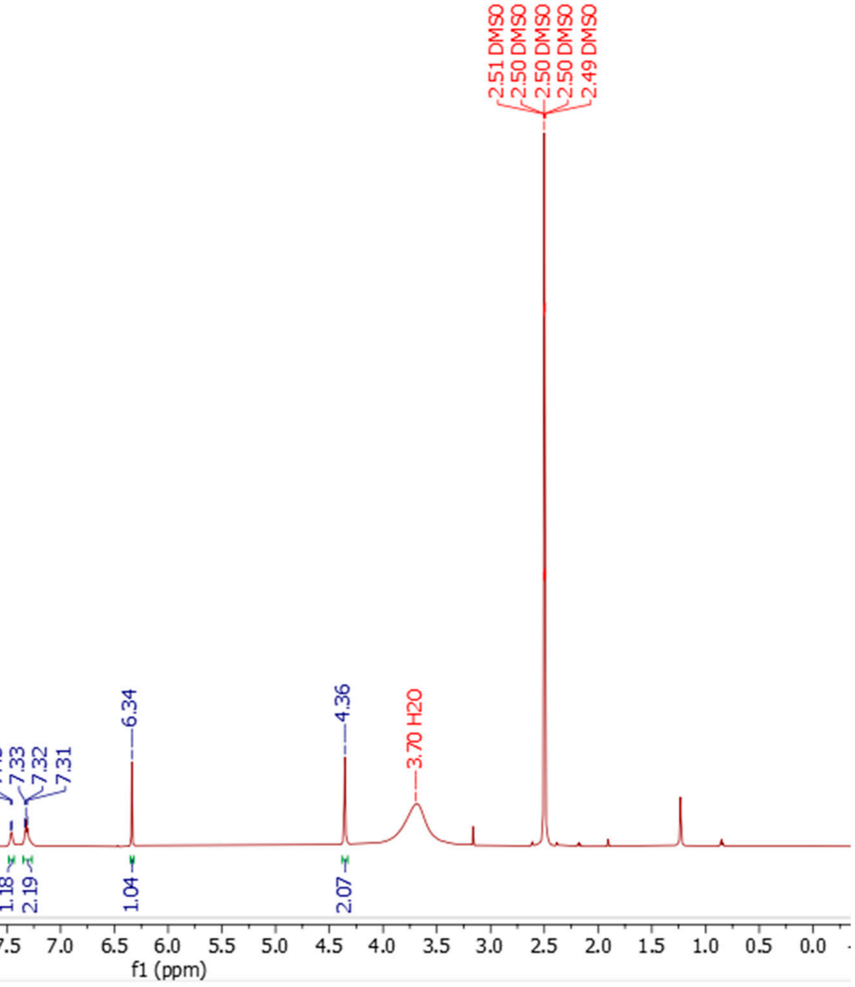
2.51 DMSO
2.50 DMSO
2.50 DMSO
2.50 DMSO
2.49 DMSO
8.52
8.52
8.51
8.51
8.49
7.46
7.45
7.33
7.32
7.31
6.34
4.36
3.70 H2O
1.04
1.18
2.19
1.04
2.07
10.5 10.0 9.5 9.0 8.5 8.0 7.5 7.0 6.5 6.0 5.5 5.0 4.5 4.0 3.5 3.0 2.5 2.0 1.5 1.0 0.5 0.0
f1 (ppm)

^{13}C NMR of **13**

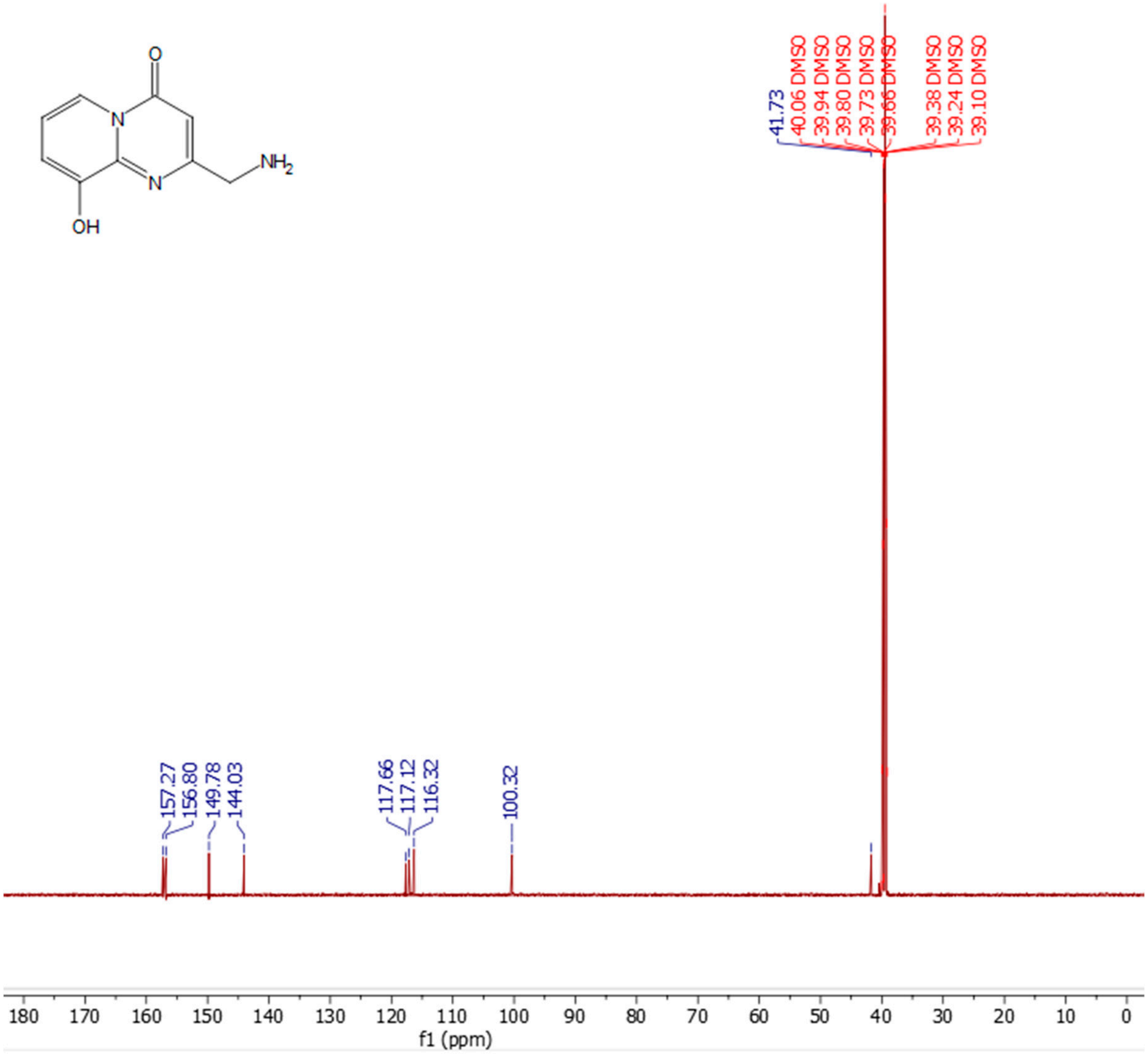

O
N
N
NH2
OH
41.73
40.06 DMSO
39.94 DMSO
39.80 DMSO
39.73 DMSO
39.66 DMSO
39.38 DMSO
39.24 DMSO
39.10 DMSO
157.27
156.80
149.78
144.03
117.66
117.12
116.32
100.32
180 170 160 150 140 130 120 110 100 90 80 70 60 50 40 30 20 10 0
f1 (ppm)

^{1}H NMR of **14**

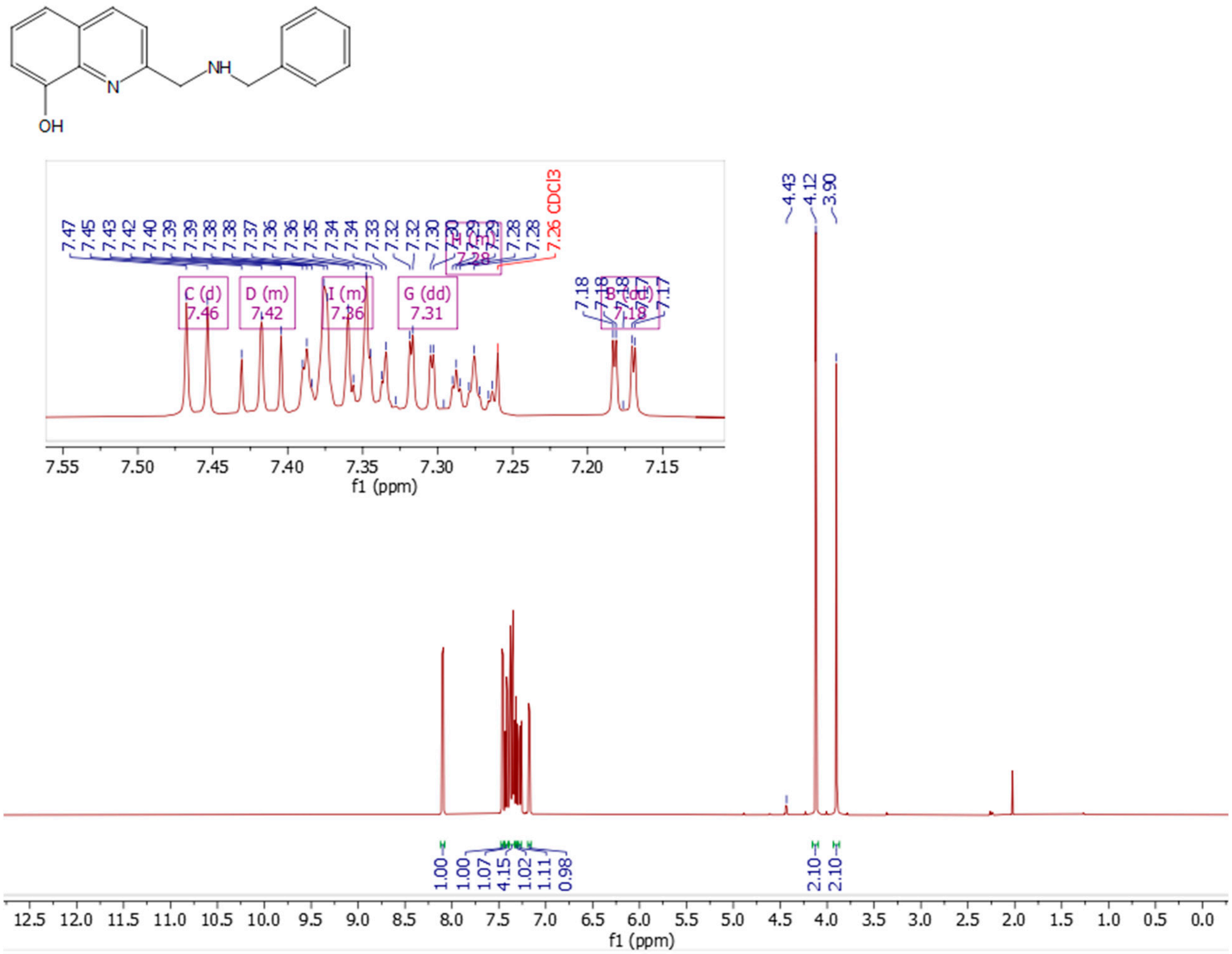

^{13}C NMR of **14**

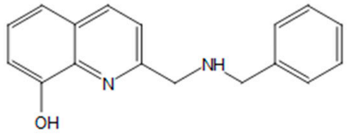

NH
N
OH

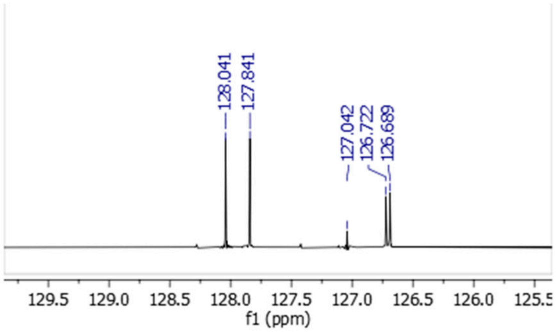

128.041
127.841
127.042
126.722
126.689
129.5
129.0
128.5
128.0
127.5
127.0
126.5
126.0
125.5
f1 (ppm)

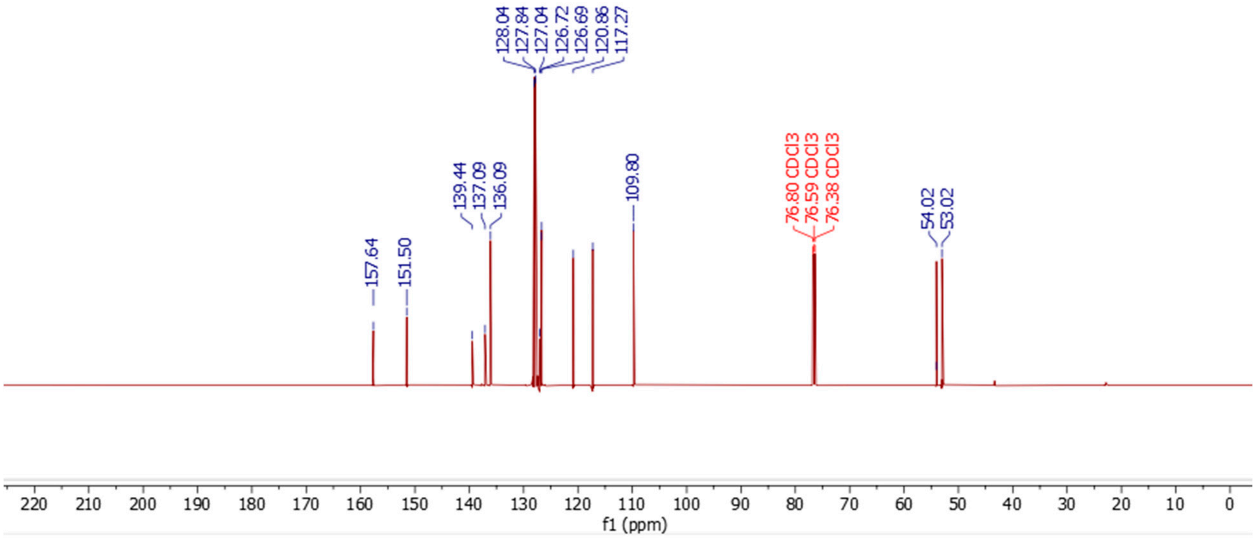

128.04
127.84
127.04
126.72
126.69
120.86
117.27
157.64
151.50
139.44
137.09
136.09
109.80
76.80 CDCl3
76.59 CDCl3
76.38 CDCl3
54.02
53.02
220
210
200
190
180
170
160
150
140
130
120
110
100
90
80
70
60
50
40
30
20
10
0
f1 (ppm)

^{1}H NMR of **16**

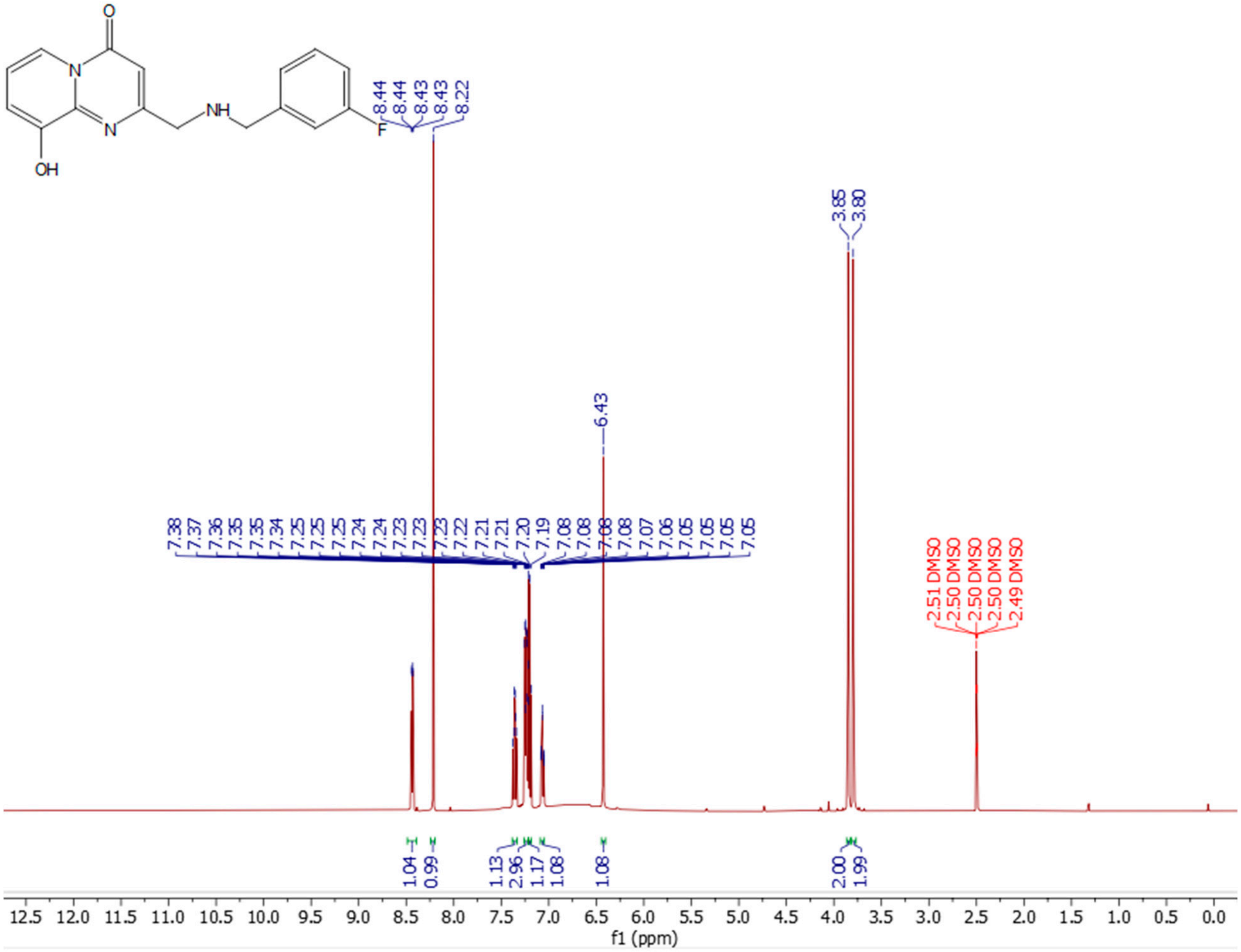

OH
N
NH
F
O
8.44
8.44
8.43
8.43
8.22
3.85
3.80
6.43
7.38
7.37
7.36
7.35
7.35
7.34
7.25
7.25
7.25
7.25
7.24
7.24
7.23
7.23
7.23
7.22
7.21
7.21
7.20
7.19
7.08
7.08
7.08
7.08
7.07
7.06
7.05
7.05
7.05
7.05
7.05
2.51 DMSO
2.50 DMSO
2.50 DMSO
2.50 DMSO
2.49 DMSO
1.04
0.99
1.13
2.96
1.17
1.08
1.08
2.00
1.99
12.5 12.0 11.5 11.0 10.5 10.0 9.5 9.0 8.5 8.0 7.5 7.0 6.5 6.0 5.5 5.0 4.5 4.0 3.5 3.0 2.5 2.0 1.5 1.0 0.5 0.0
f1 (ppm)

^{13}C NMR of **16**

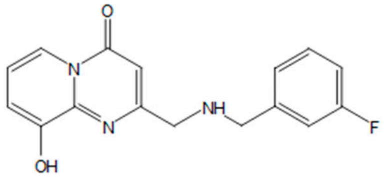

O
N
N
NH
F
OH

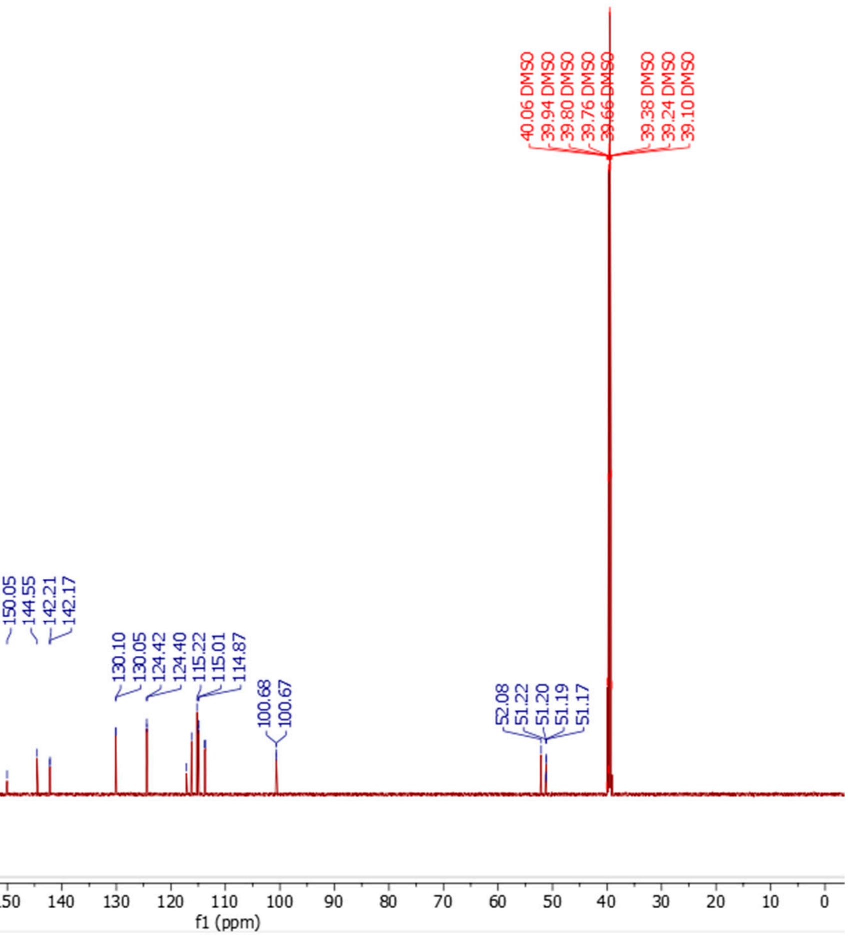

40.06 DMSO
39.94 DMSO
39.80 DMSO
39.76 DMSO
39.66 DMSO
39.38 DMSO
39.24 DMSO
39.10 DMSO
163.84
163.04
162.97
161.43
157.27
150.05
144.55
142.21
142.17
130.10
130.05
124.42
124.40
115.22
115.01
114.87
100.68
100.67
52.08
51.22
51.20
51.19
51.17
220 210 200 190 180 170 160 150 140 130 120 110 100 90 80 70 60 50 40 30 20 10 0
f1 (ppm)

^1H NMR of **20**

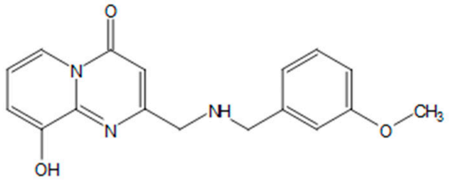

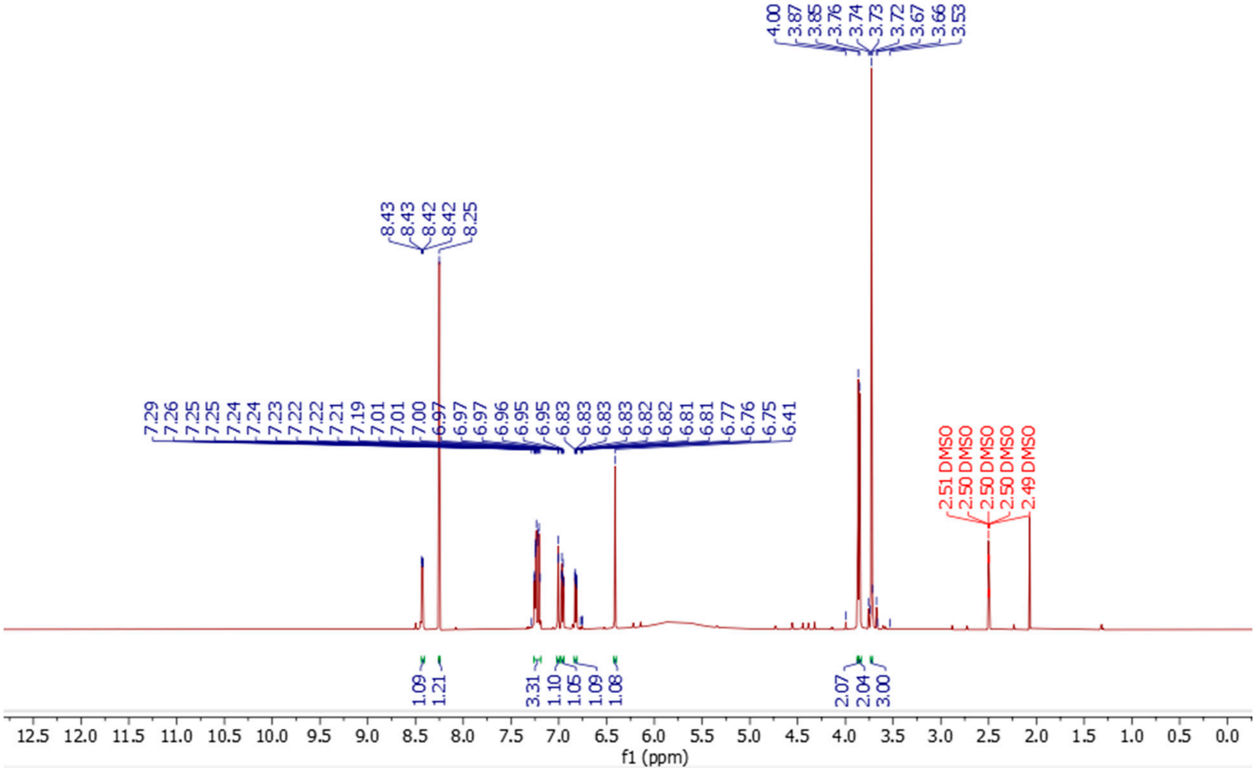

4.00
3.87
3.85
3.76
3.74
3.73
3.72
3.67
3.66
3.53
8.43
8.43
8.42
8.42
8.25
7.29
7.26
7.25
7.25
7.24
7.24
7.23
7.22
7.22
7.21
7.19
7.01
7.00
6.97
6.97
6.97
6.96
6.95
6.95
6.83
6.83
6.83
6.83
6.82
6.82
6.81
6.81
6.77
6.76
6.75
6.41
2.51 DMSO
2.50 DMSO
2.50 DMSO
2.50 DMSO
2.49 DMSO
1.09
1.21
3.31
1.10
1.05
1.09
1.08
2.07
2.04
3.00
12.5 12.0 11.5 11.0 10.5 10.0 9.5 9.0 8.5 8.0 7.5 7.0 6.5 6.0 5.5 5.0 4.5 4.0 3.5 3.0 2.5 2.0 1.5 1.0 0.5 0.0
f1 (ppm)

^{13}C NMR of **20**

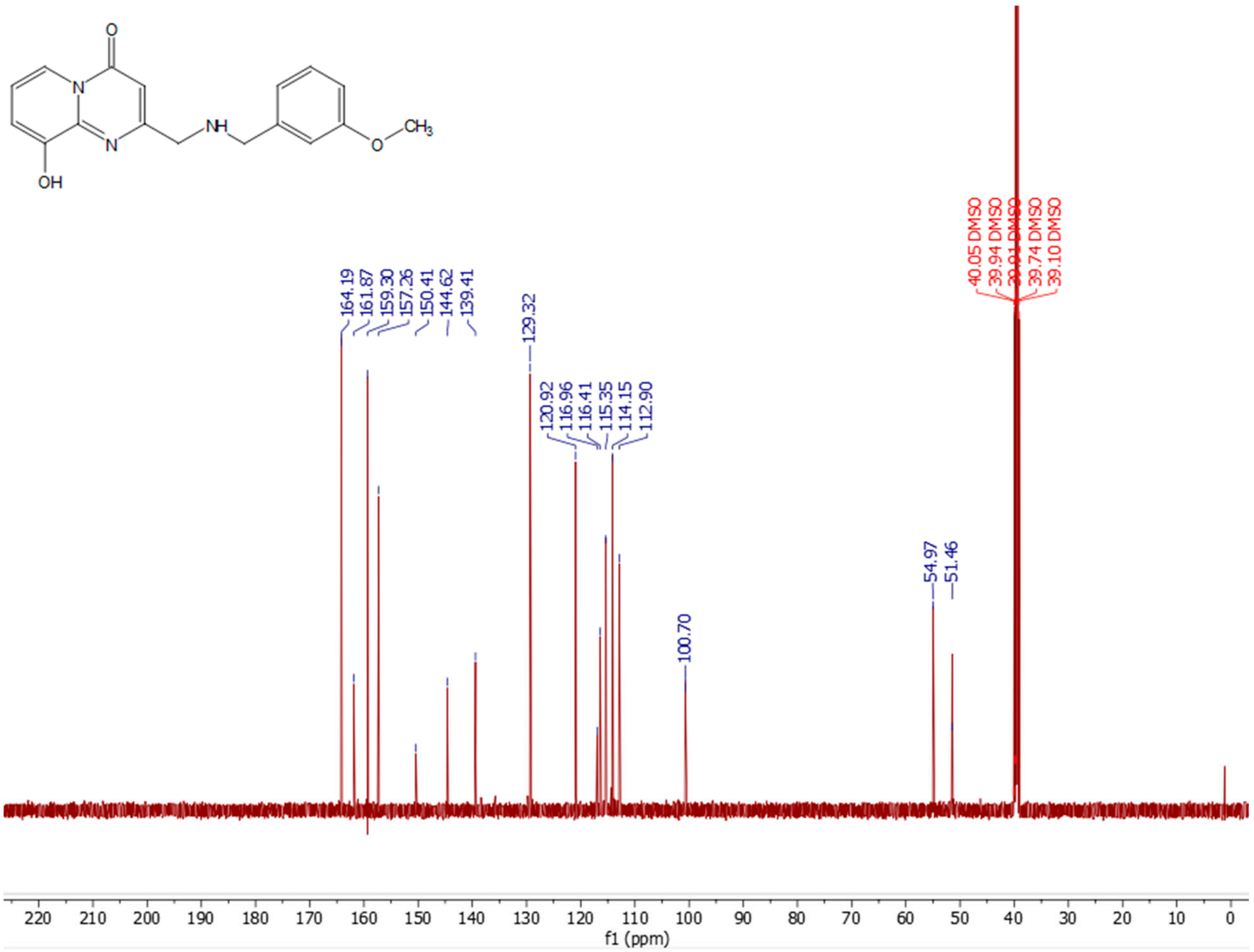

O
N
NH
N
O
CH3
OH
164.19
161.87
159.30
157.26
150.41
144.62
139.41
129.32
120.92
116.96
116.41
115.35
114.15
112.90
100.70
54.97
51.46
40.05 DMSO
39.94 DMSO
39.91 DMSO
39.74 DMSO
39.10 DMSO
220 210 200 190 180 170 160 150 140 130 120 110 100 90 80 70 60 50 40 30 20 10 0
f1 (ppm)

^{1}H NMR of **21**

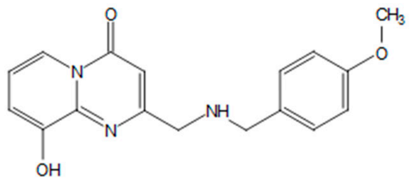

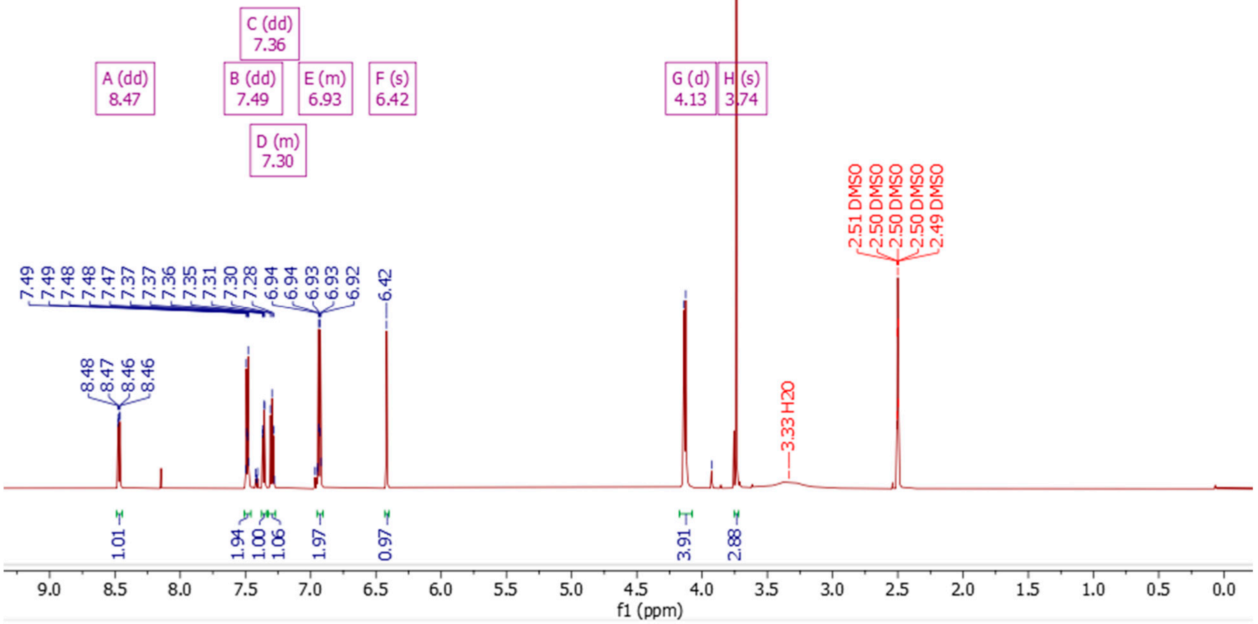

A (dd) 8.47
B (dd) 7.49
C (dd) 7.36
D (m) 7.30
E (m) 6.93
F (s) 6.42
G (d) 4.13
H (s) 3.74
3.33 H2O
2.51 DMSO
2.50 DMSO
2.50 DMSO
2.50 DMSO
2.49 DMSO
f1 (ppm)

^{13}C NMR of **21**

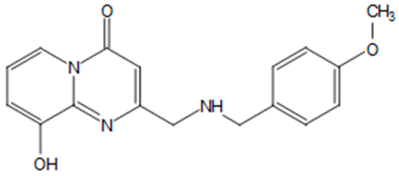
O
CH3
O
N
NH
N
OH

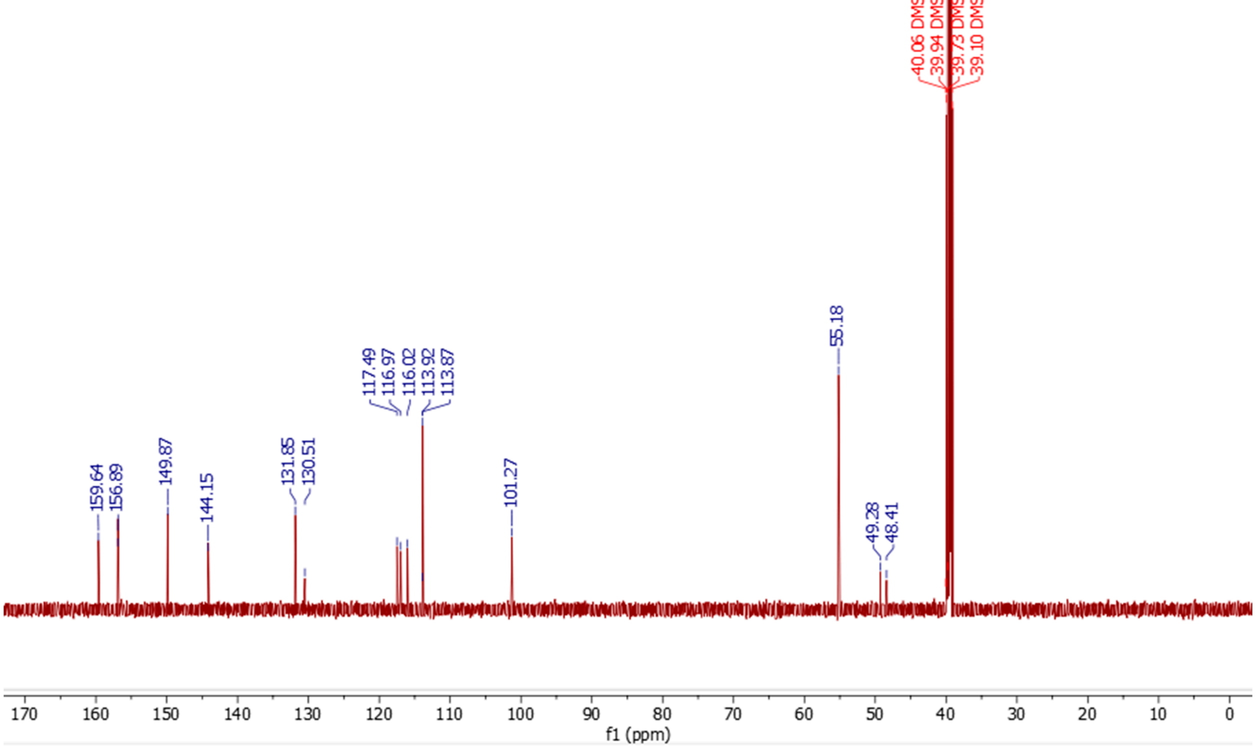
40.06 DMSO
39.94 DMSO
39.73 DMSO
39.10 DMSO
55.18
117.49
116.97
116.02
113.92
113.87
159.64
156.89
149.87
144.15
131.85
130.51
101.27
49.28
48.41
170
160
150
140
130
120
110
100
90
80
70
60
50
40
30
20
10
0
f1 (ppm)

^{1}H NMR of **23**

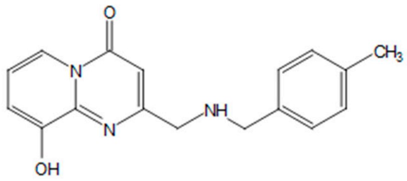

O
N
N
NH
CH3
OH

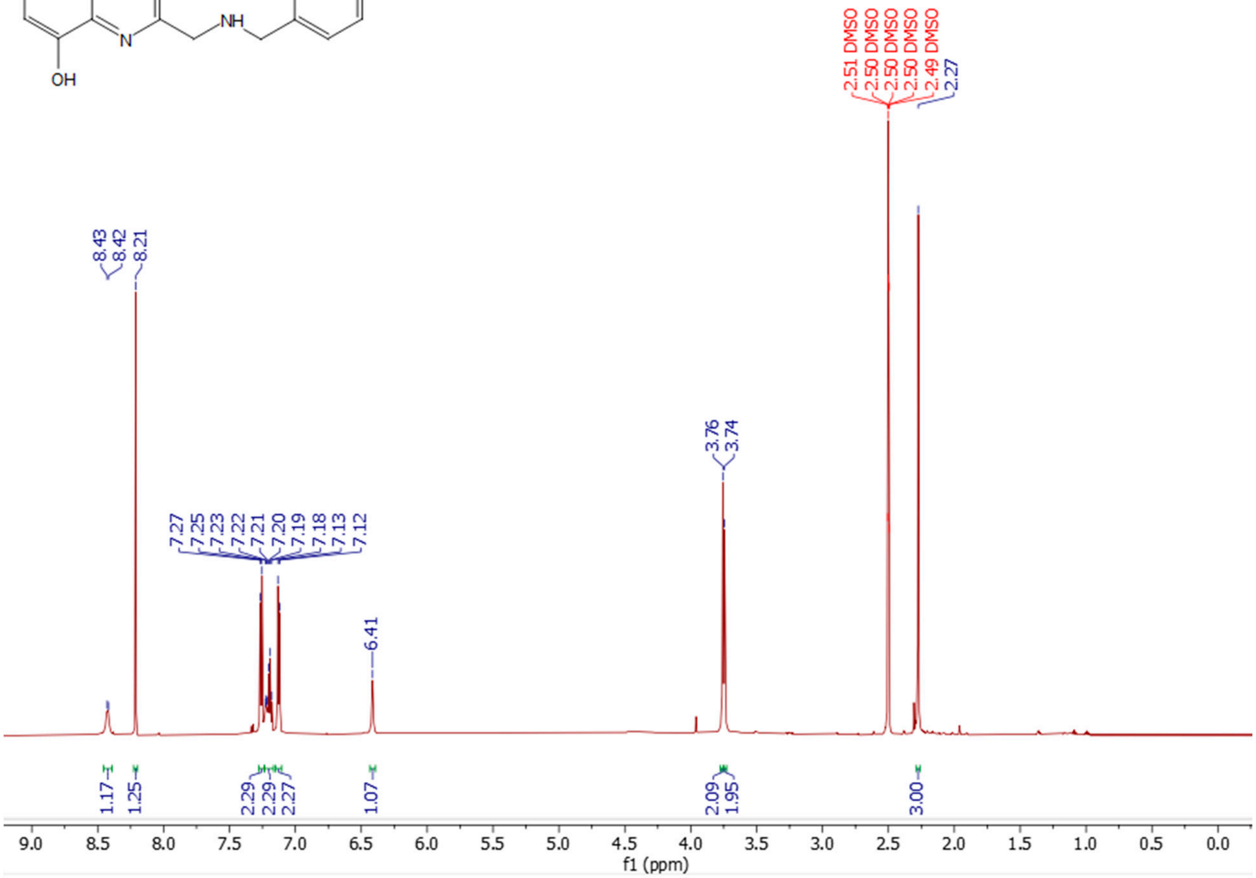

2.51 DMSO
2.50 DMSO
2.50 DMSO
2.50 DMSO
2.49 DMSO
2.27
8.43
8.42
8.21
3.76
3.74
7.27
7.25
7.23
7.22
7.21
7.20
7.19
7.18
7.13
7.12
6.41
1.17
1.25
2.29
2.29
2.27
1.07
2.09
1.95
3.00
9.0 8.5 8.0 7.5 7.0 6.5 6.0 5.5 5.0 4.5 4.0 3.5 3.0 2.5 2.0 1.5 1.0 0.5 0.0
f1 (ppm)

^{13}C NMR of **23**

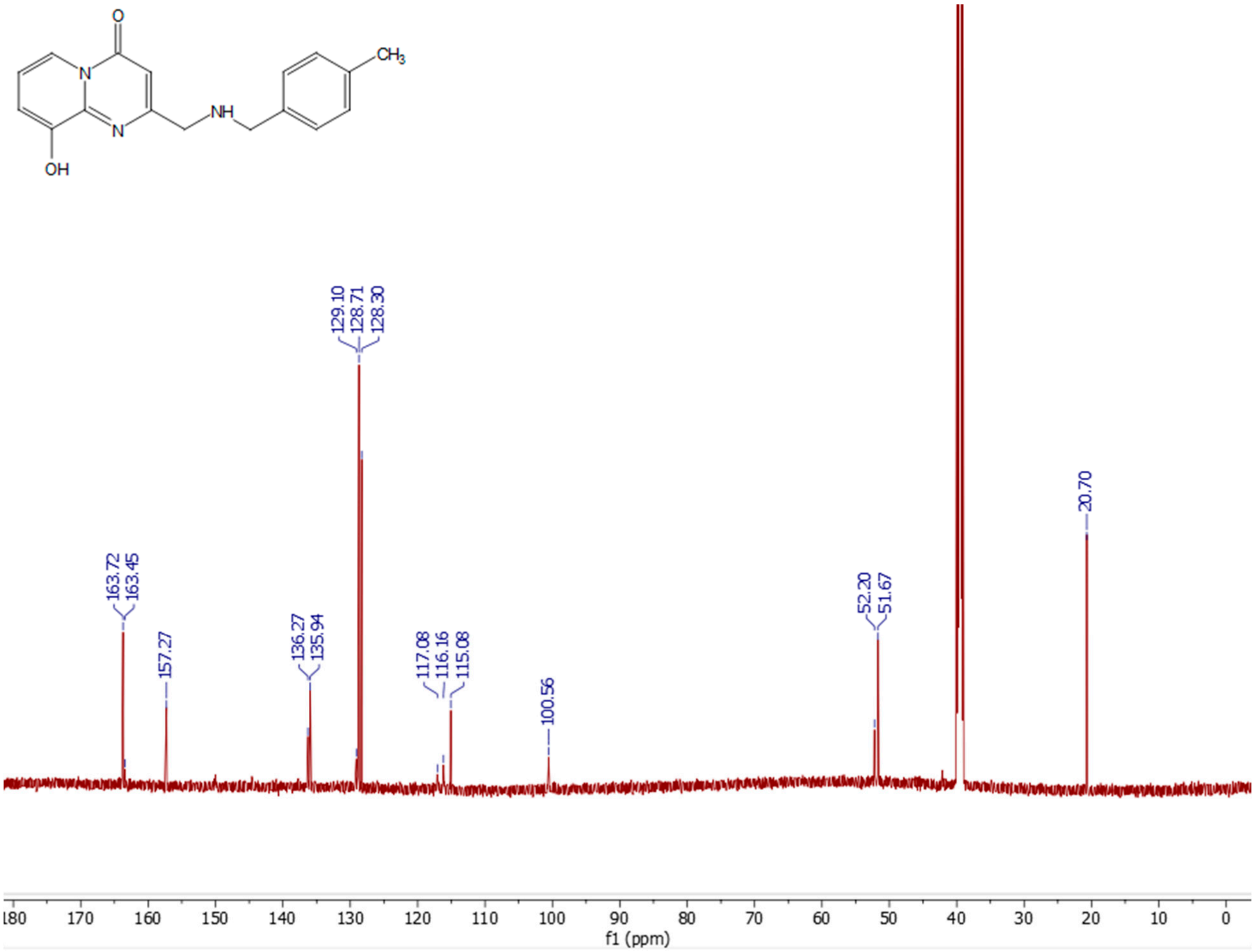

O
N
N
NH
CH3
OH
163.72
163.45
157.27
136.27
135.94
129.10
128.71
128.30
117.08
116.16
115.08
100.56
52.20
51.67
20.70
180
170
160
150
140
130
120
110
100
90
80
70
60
50
40
30
20
10
0
f1 (ppm)

^{1}H NMR of **24**

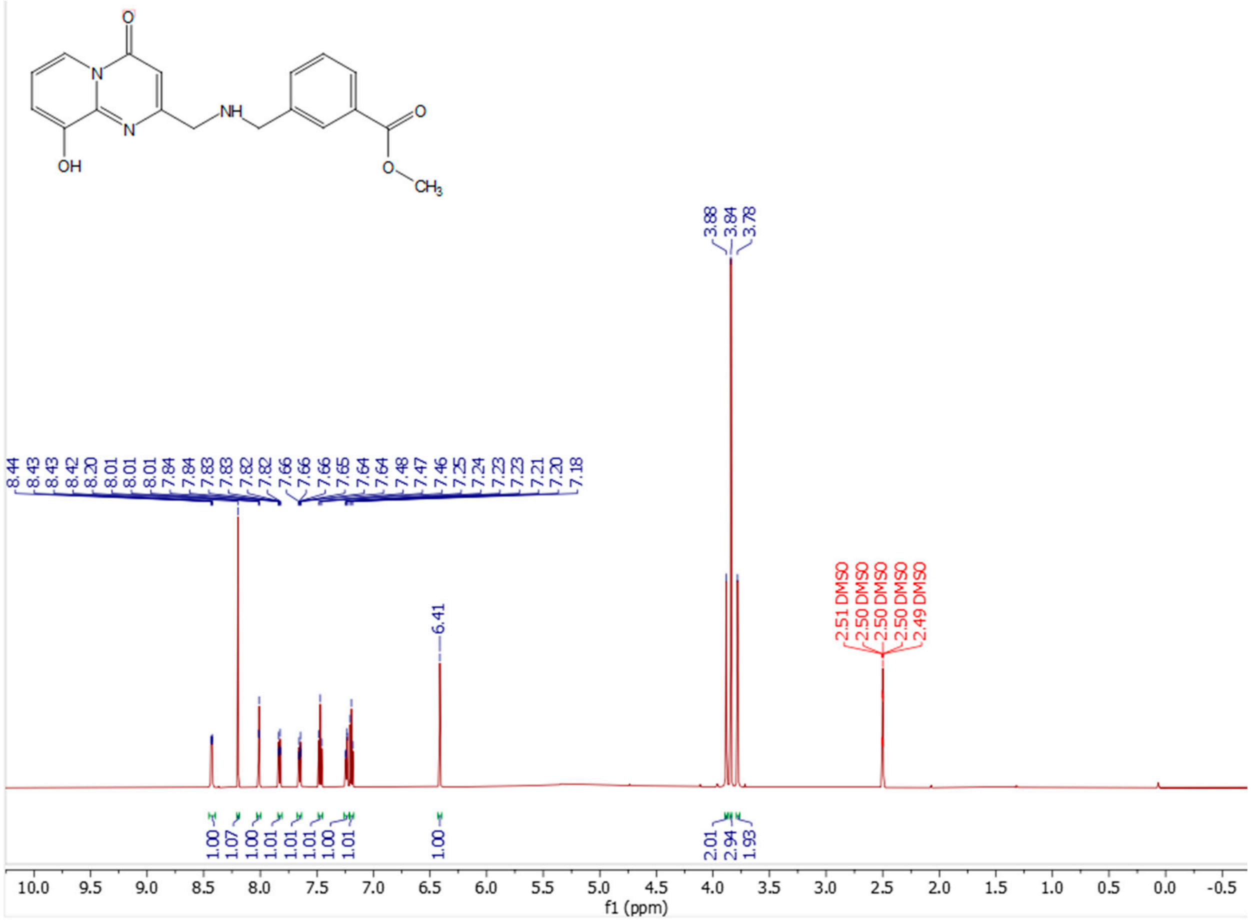

O
N
NH
N
O
OH
O
CH3
3.88
3.84
3.78
8.44
8.43
8.43
8.42
8.20
8.01
8.01
7.84
7.84
7.83
7.83
7.82
7.82
7.66
7.66
7.66
7.65
7.64
7.64
7.48
7.47
7.46
7.25
7.24
7.23
7.23
7.21
7.20
7.18
6.41
2.51 DMSO
2.50 DMSO
2.50 DMSO
2.50 DMSO
2.49 DMSO
1.00
1.07
1.00
1.01
1.01
1.00
1.01
1.00
2.01
2.94
1.93
10.0 9.5 9.0 8.5 8.0 7.5 7.0 6.5 6.0 5.5 5.0 4.5 4.0 3.5 3.0 2.5 2.0 1.5 1.0 0.5 0.0 -0.5
f1 (ppm)

^{13}C NMR of **24**

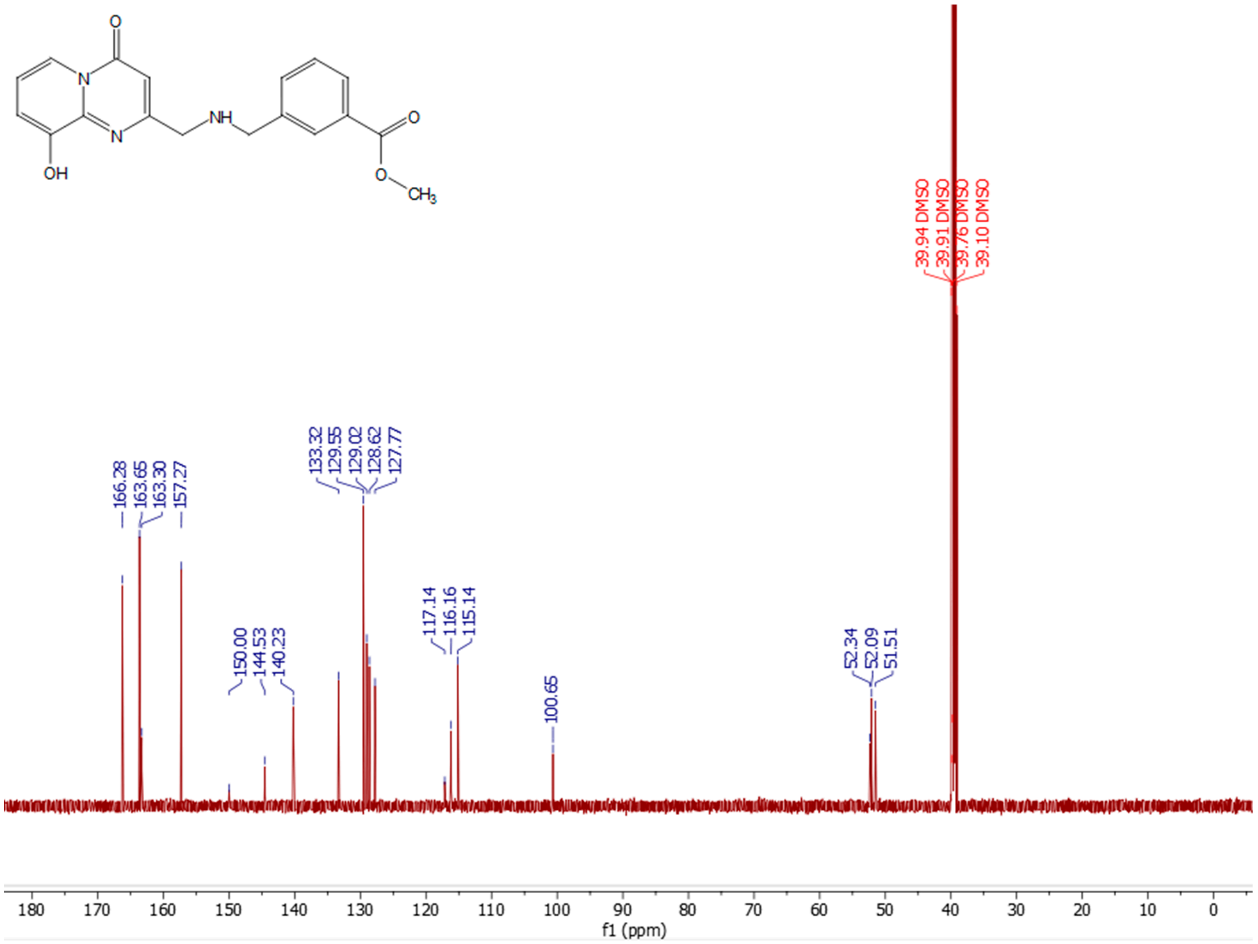

39.94 DMSO
39.91 DMSO
39.76 DMSO
39.10 DMSO
166.28
163.65
163.30
157.27
150.00
144.53
140.23
133.32
129.55
129.02
128.62
127.77
117.14
116.16
115.14
100.65
52.34
52.09
51.51
180 170 160 150 140 130 120 110 100 90 80 70 60 50 40 30 20 10 0
f1 (ppm)

^{1}H NMR of **25**

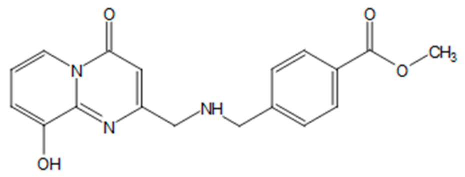

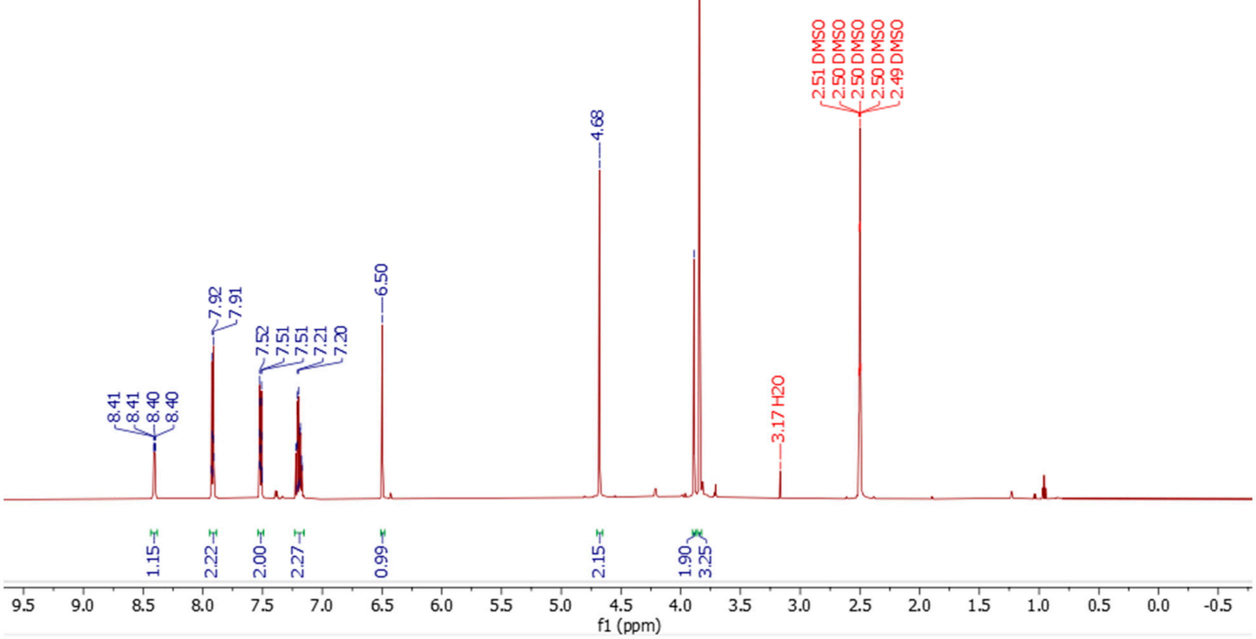

8.41
8.41
8.40
8.40
7.92
7.91
7.52
7.51
7.51
7.21
7.20
6.50
4.68
3.17 H2O
2.51 DMSO
2.50 DMSO
2.50 DMSO
2.50 DMSO
2.49 DMSO
1.15
2.22
2.00
2.27
0.99
2.15
1.90
3.25
9.5 9.0 8.5 8.0 7.5 7.0 6.5 6.0 5.5 5.0 4.5 4.0 3.5 3.0 2.5 2.0 1.5 1.0 0.5 0.0 -0.5
f1 (ppm)

^{13}C NMR of **25**

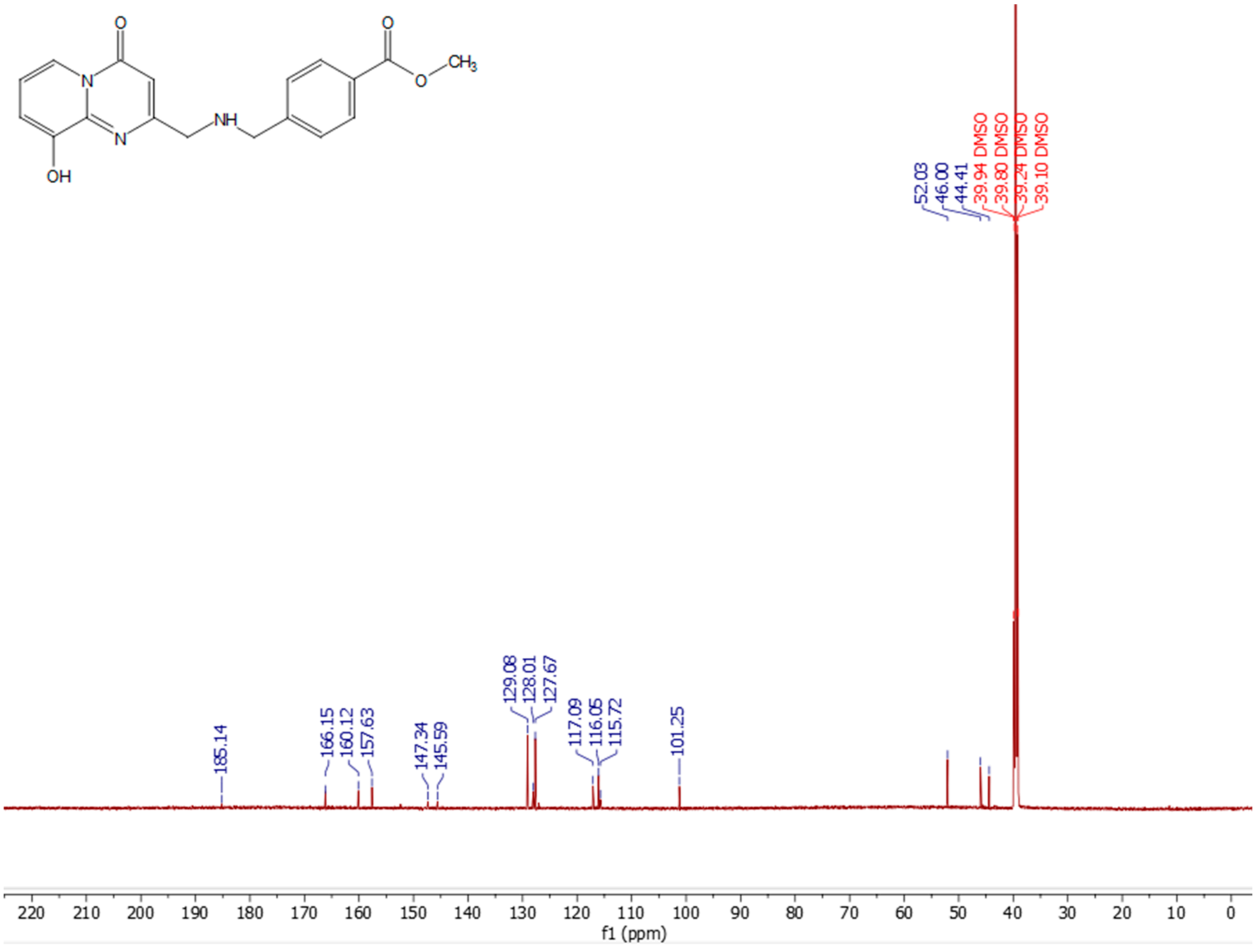

OH
N
N
O
NH
O
O
CH3
52.03
46.00
44.41
39.94 DMSO
39.80 DMSO
39.24 DMSO
39.10 DMSO
185.14
166.15
160.12
157.63
147.34
145.59
129.08
128.01
127.67
117.09
116.05
115.72
101.25
220 210 200 190 180 170 160 150 140 130 120 110 100 90 80 70 60 50 40 30 20 10 0
f1 (ppm)

^{1}H NMR of **26**

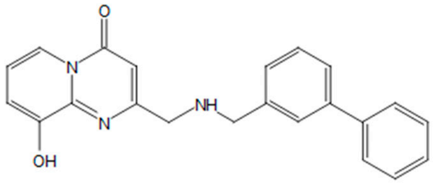

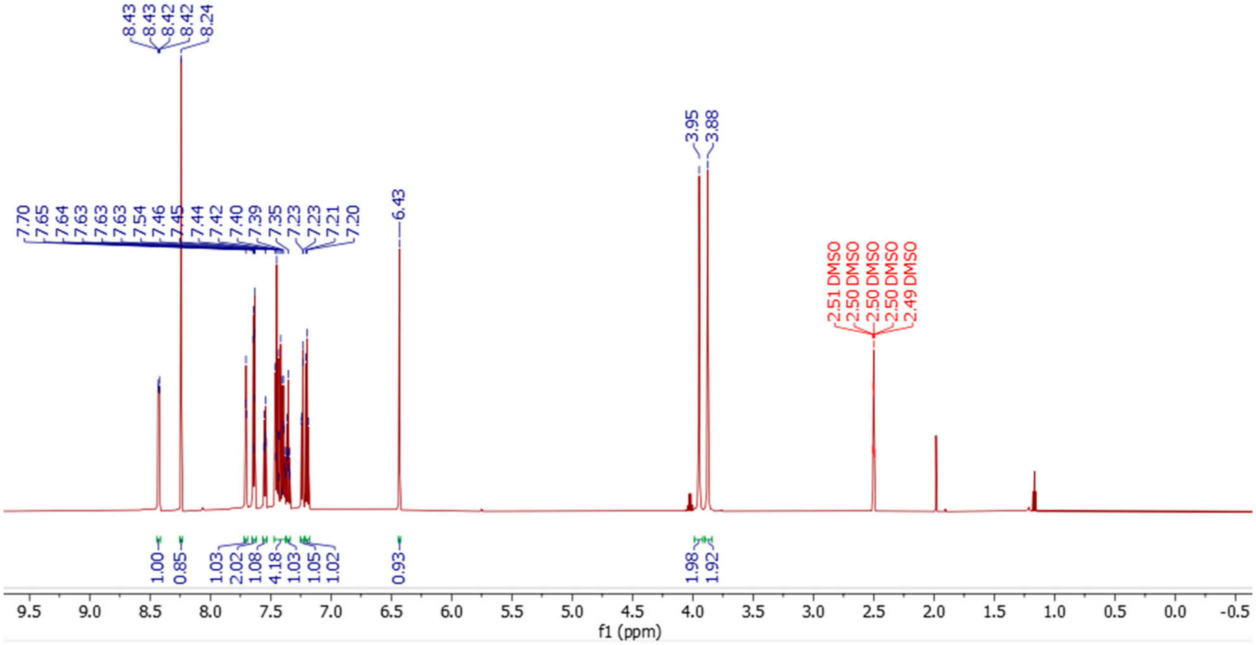

8.43
8.43
8.42
8.42
8.24
7.70
7.65
7.64
7.63
7.63
7.63
7.54
7.46
7.45
7.44
7.42
7.40
7.39
7.35
7.23
7.23
7.21
7.20
6.43
3.95
3.88
2.51 DMSO
2.50 DMSO
2.50 DMSO
2.50 DMSO
2.49 DMSO
1.00
0.85
1.03
2.02
1.08
4.18
1.03
1.05
1.02
0.93
1.98
1.92
9.5 9.0 8.5 8.0 7.5 7.0 6.5 6.0 5.5 5.0 4.5 4.0 3.5 3.0 2.5 2.0 1.5 1.0 0.5 0.0 -0.5
f1 (ppm)

^{13}C NMR of **26**

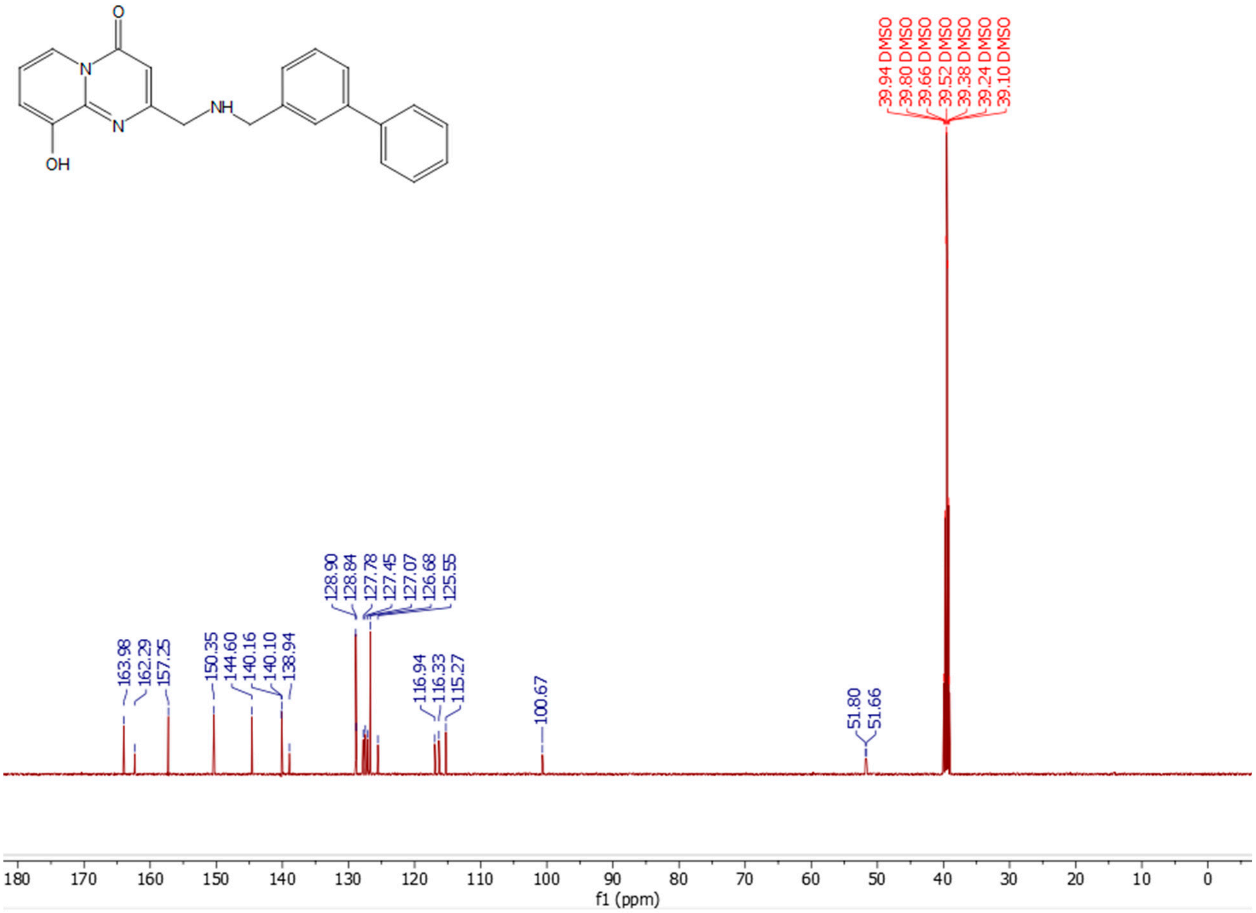

O
N
N
NH
OH
39.94 DMSO
39.80 DMSO
39.66 DMSO
39.52 DMSO
39.38 DMSO
39.24 DMSO
39.10 DMSO
128.90
128.84
127.78
127.45
127.07
126.68
125.55
163.98
162.29
157.25
150.35
144.60
140.16
140.10
138.94
116.94
116.33
115.27
100.67
51.80
51.66
180 170 160 150 140 130 120 110 100 90 80 70 60 50 40 30 20 10 0
f1 (ppm)

^{1}H NMR of **27**

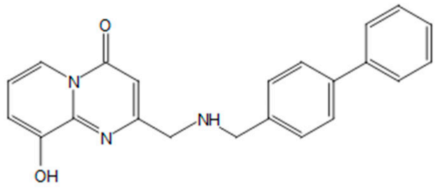

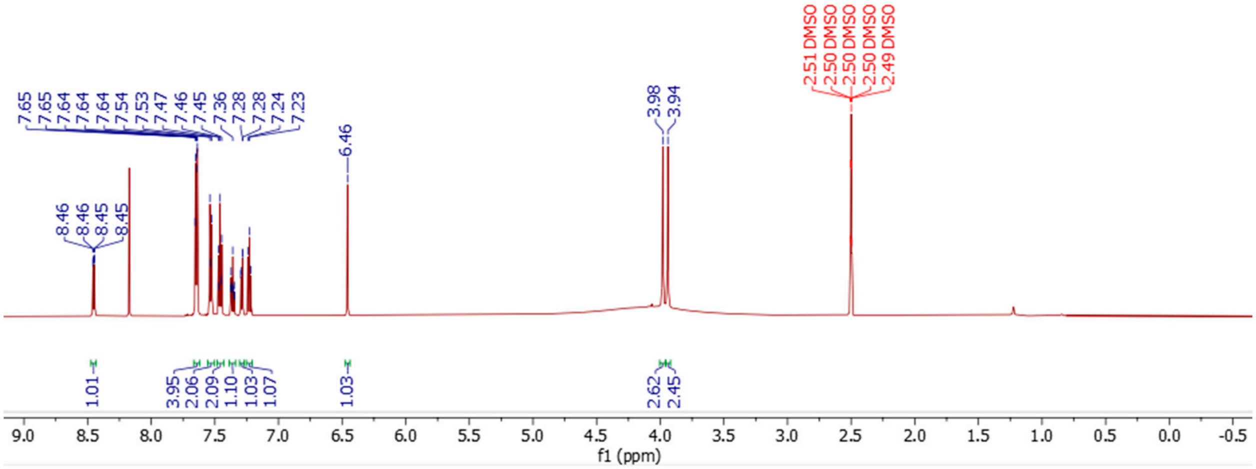

7.65
7.65
7.64
7.64
7.64
7.54
7.53
7.47
7.46
7.45
7.36
7.28
7.28
7.24
7.23
8.46
8.46
8.45
8.45
6.46
3.98
3.94
2.51 DMSO
2.50 DMSO
2.50 DMSO
2.50 DMSO
2.49 DMSO
1.01
3.95
2.06
2.09
1.10
1.03
1.07
1.03
2.62
2.45
9.0 8.5 8.0 7.5 7.0 6.5 6.0 5.5 5.0 4.5 4.0 3.5 3.0 2.5 2.0 1.5 1.0 0.5 0.0 -0.5
f1 (ppm)

^{13}C NMR of **27**

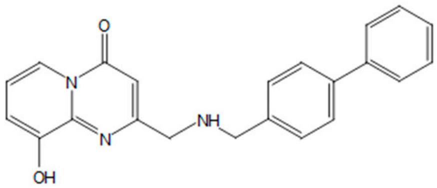

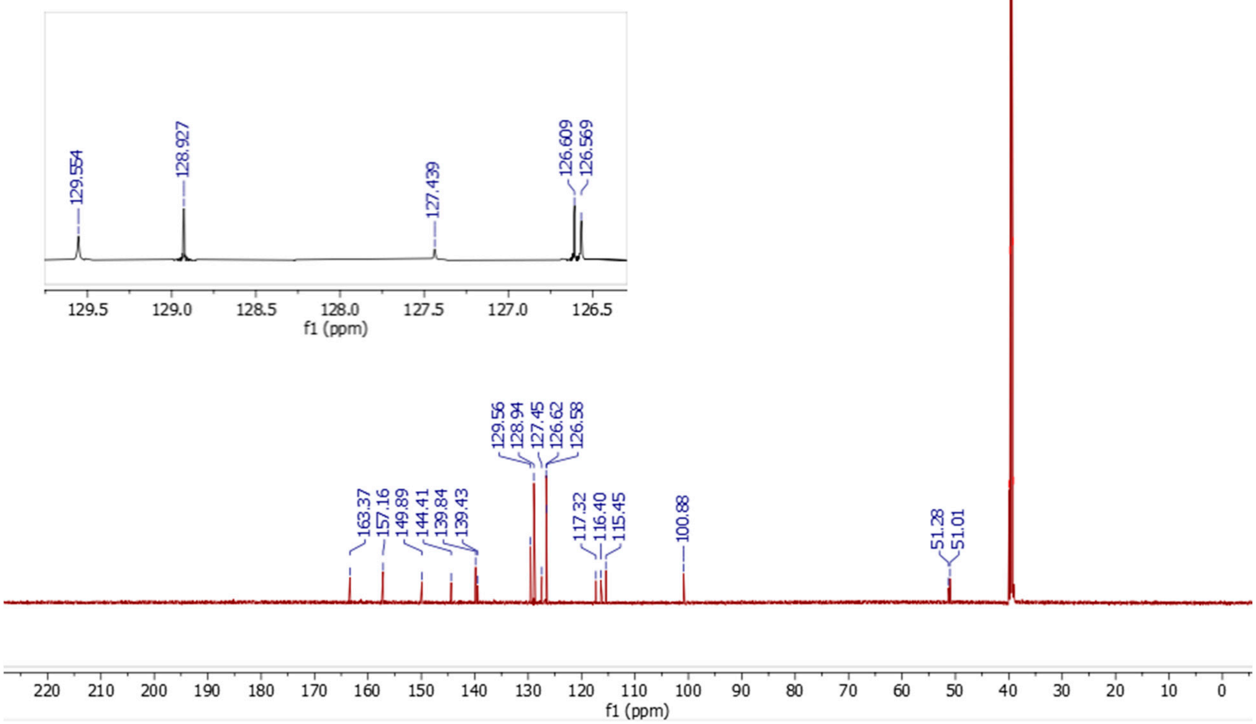

39.94 DMSO
39.80 DMSO
39.66 DMSO
39.38 DMSO
39.24 DMSO
39.10 DMSO
129.554
128.927
127.439
126.609
126.569
129.5
129.0
128.5
128.0
127.5
127.0
126.5
f1 (ppm)
129.56
128.94
127.45
126.62
126.58
163.37
157.16
149.89
144.41
139.84
139.43
117.32
116.40
115.45
100.88
51.28
51.01
220
210
200
190
180
170
160
150
140
130
120
110
100
90
80
70
60
50
40
30
20
10
0
f1 (ppm)

^{1}H NMR of **28**

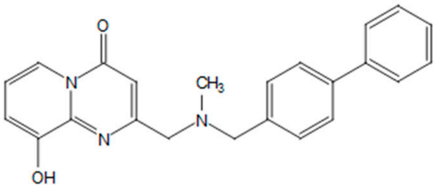

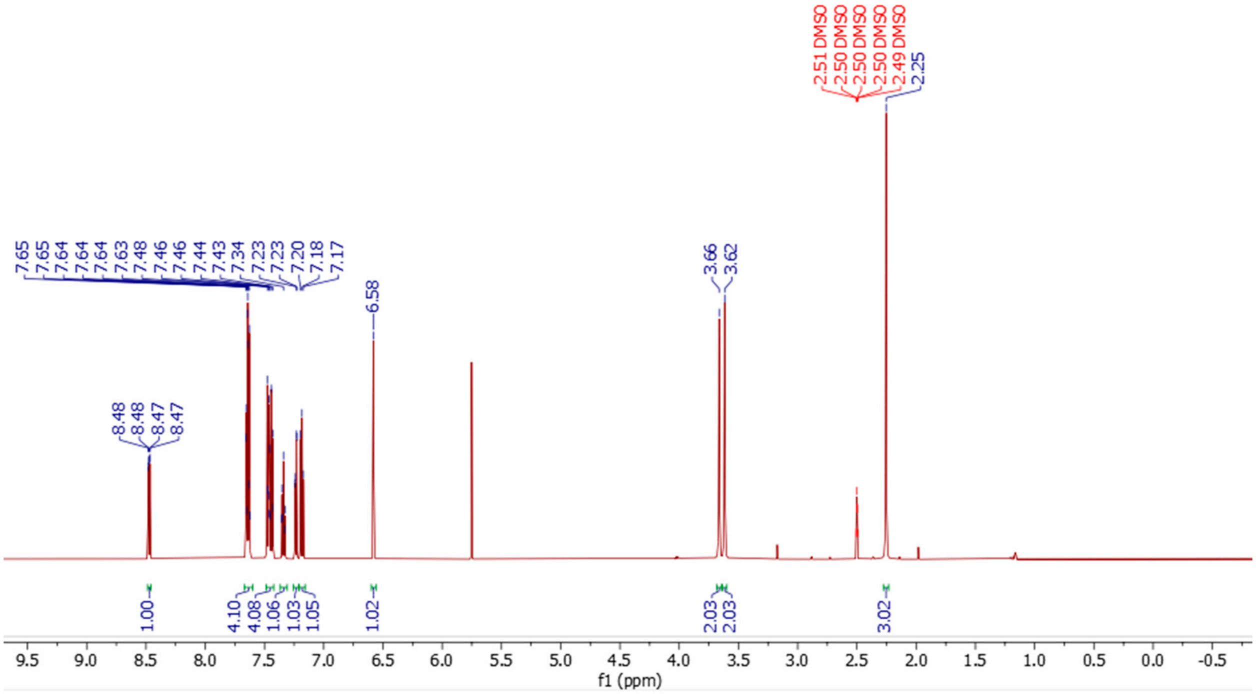

2.51 DMSO
2.50 DMSO
2.50 DMSO
2.50 DMSO
2.49 DMSO
2.25
7.65
7.65
7.64
7.64
7.64
7.63
7.48
7.46
7.46
7.44
7.43
7.34
7.23
7.23
7.20
7.18
7.17
6.58
3.66
3.62
8.48
8.48
8.47
8.47
1.00
4.10
4.08
1.06
1.03
1.05
1.02
2.03
2.03
3.02
9.5
9.0
8.5
8.0
7.5
7.0
6.5
6.0
5.5
5.0
4.5
4.0
3.5
3.0
2.5
2.0
1.5
1.0
0.5
0.0
-0.5
f1 (ppm)

^{13}C NMR of **28**

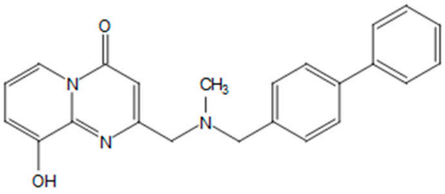

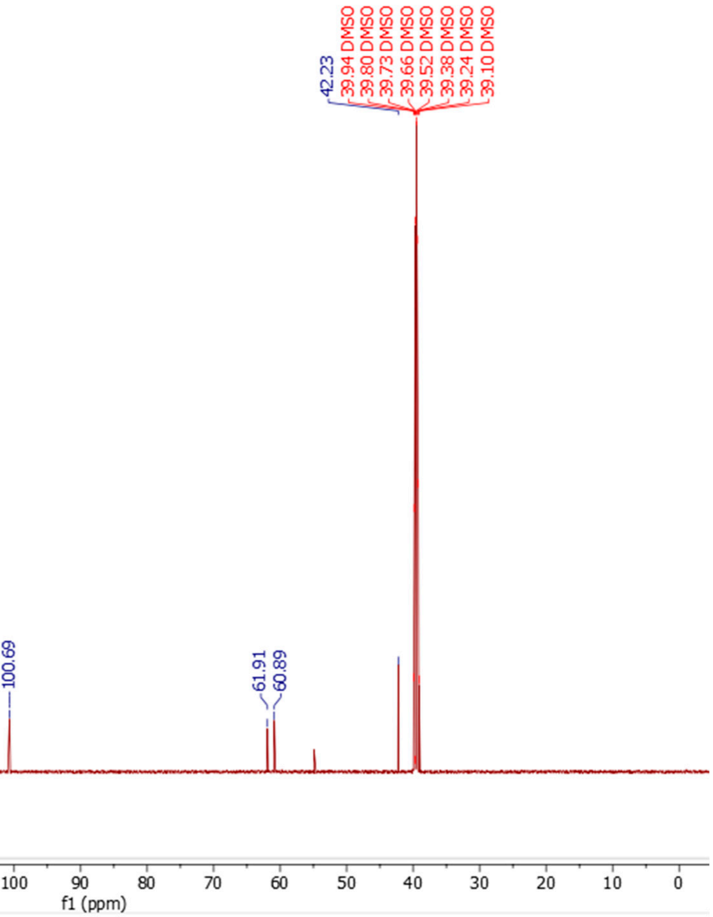

42.23
39.94 DMSO
39.80 DMSO
39.73 DMSO
39.66 DMSO
39.52 DMSO
39.38 DMSO
39.24 DMSO
39.10 DMSO
129.20
128.89
127.29
126.56
164.10
157.47
149.85
144.52
139.96
138.90
138.03
117.39
115.98
115.37
100.69
61.91
60.89
180 170 160 150 140 130 120 110 100 90 80 70 60 50 40 30 20 10 0
f1 (ppm)

^1H NMR of **29**

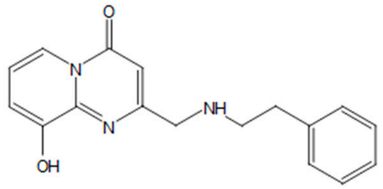

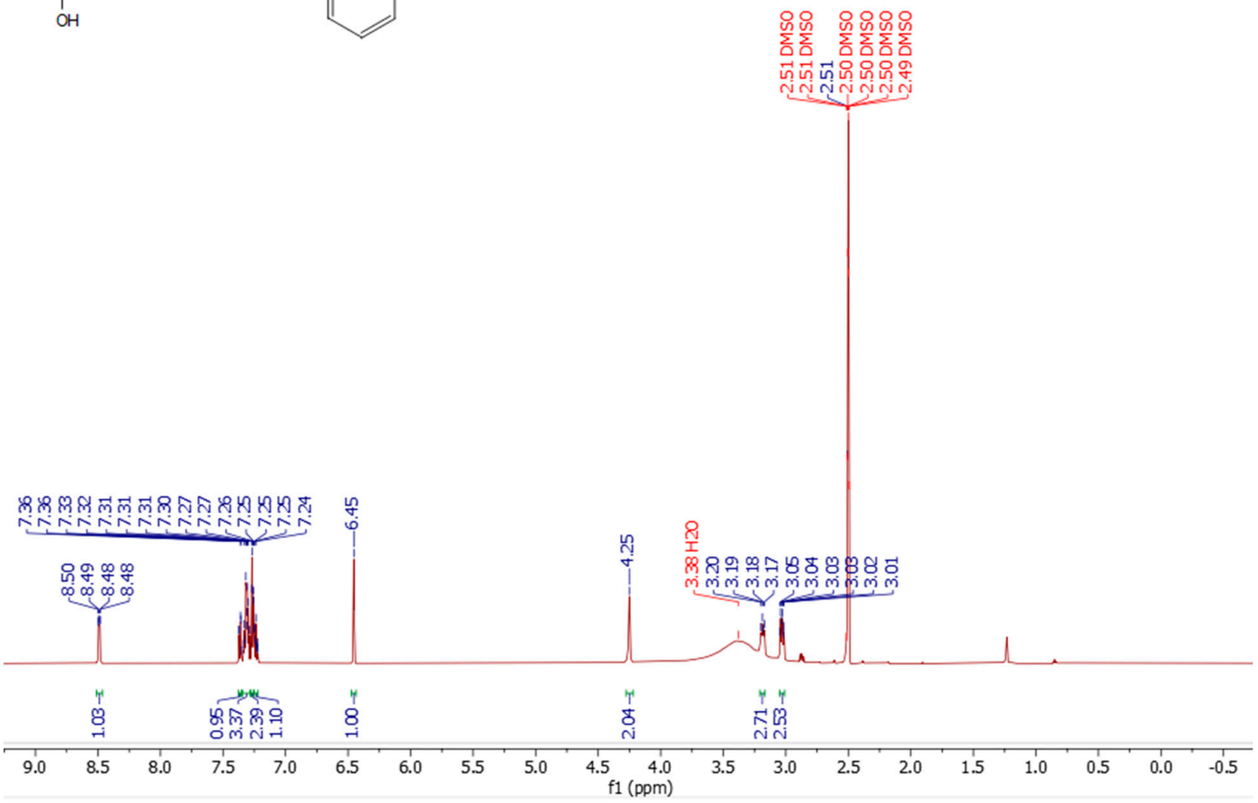
2.51 DMSO
2.51 DMSO
2.51
2.50 DMSO
2.50 DMSO
2.50 DMSO
2.49 DMSO
7.36
7.36
7.33
7.32
7.31
7.31
7.31
7.30
7.27
7.27
7.26
7.25
7.25
7.25
7.24
6.45
8.50
8.49
8.48
8.48
4.25
3.38 H2O
3.20
3.19
3.18
3.17
3.05
3.04
3.03
3.03
3.02
3.01
1.03
0.95
3.37
2.39
1.10
1.00
2.04
2.71
2.53
9.0
8.5
8.0
7.5
7.0
6.5
6.0
5.5
5.0
4.5
4.0
3.5
3.0
2.5
2.0
1.5
1.0
0.5
0.0
-0.5
f1 (ppm)

^{13}C NMR of **29**

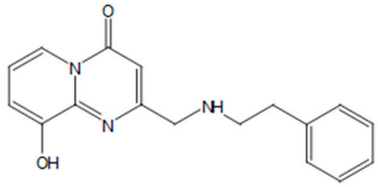

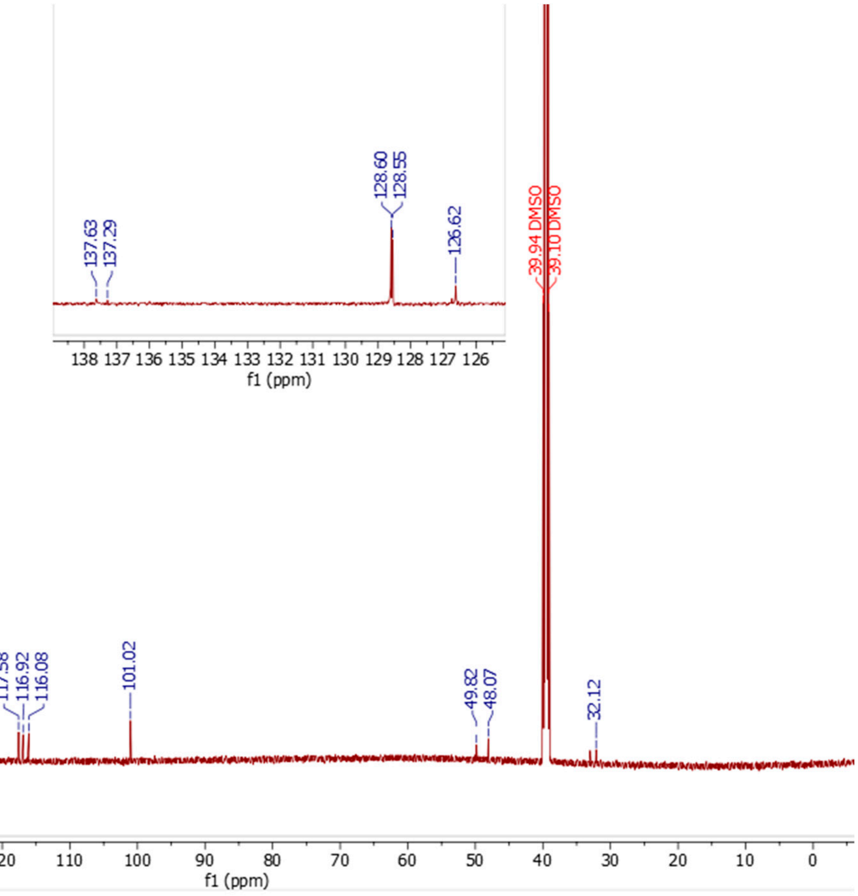

137.63
137.29
128.60
128.55
126.62
39.94 DMSO
39.10 DMSO
138 137 136 135 134 133 132 131 130 129 128 127 126
f1 (ppm)
156.94
149.83
144.26
137.63
137.29
128.60
128.55
126.62
117.58
116.92
116.08
101.02
49.82
48.07
32.12
170 160 150 140 130 120 110 100 90 80 70 60 50 40 30 20 10 0
f1 (ppm)

^{1}H NMR of **30**

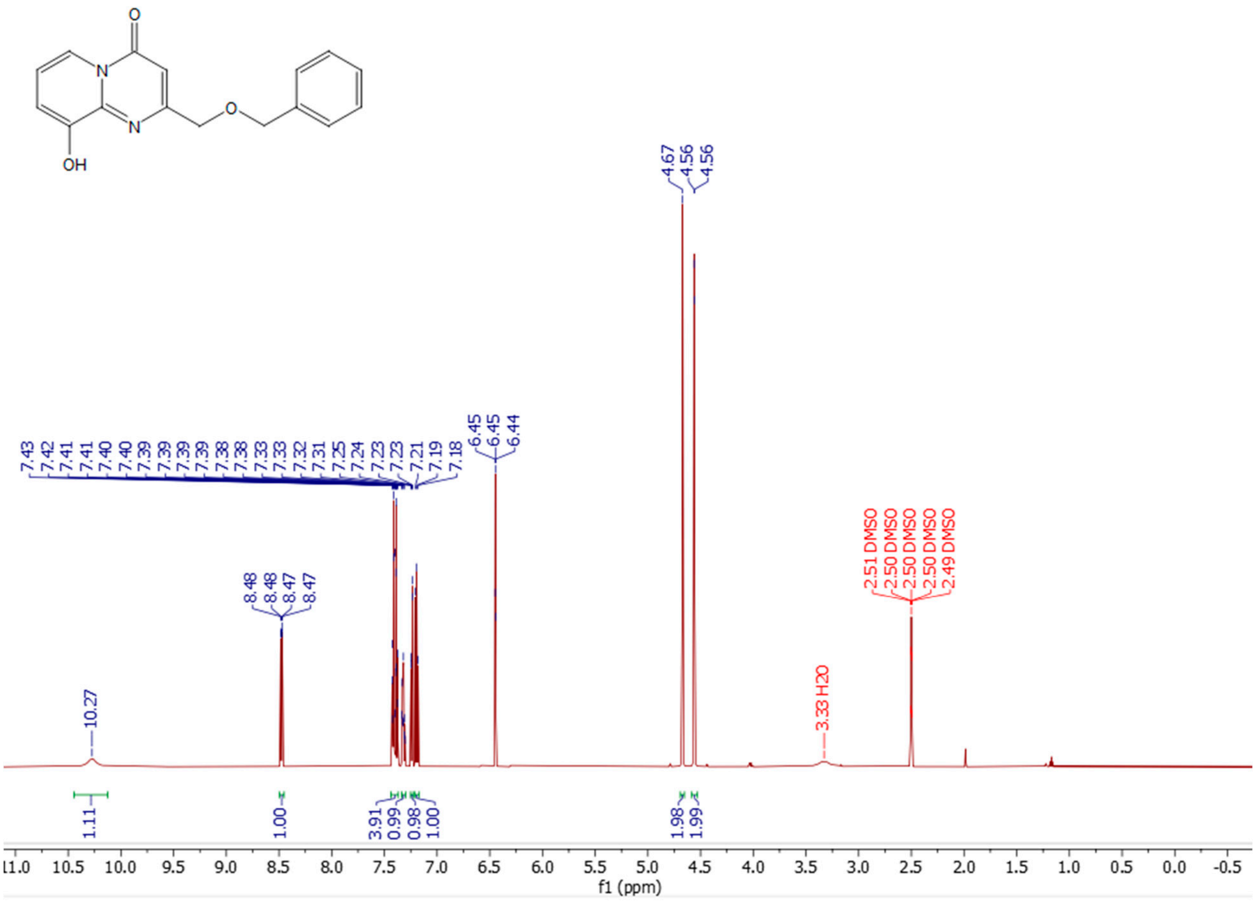

O
N
N
O
OH
4.67
4.56
4.56
7.43
7.42
7.41
7.41
7.40
7.40
7.39
7.39
7.39
7.39
7.38
7.38
7.33
7.33
7.32
7.31
7.25
7.24
7.23
7.23
7.21
7.19
7.18
6.45
6.45
6.44
2.51 DMSO
2.50 DMSO
2.50 DMSO
2.50 DMSO
2.49 DMSO
8.48
8.48
8.47
8.47
10.27
3.33 H2O
1.11
1.00
3.91
0.99
0.98
1.00
1.98
1.99
11.0 10.5 10.0 9.5 9.0 8.5 8.0 7.5 7.0 6.5 6.0 5.5 5.0 4.5 4.0 3.5 3.0 2.5 2.0 1.5 1.0 0.5 0.0 -0.5
f1 (ppm)

^{13}C NMR of **30**

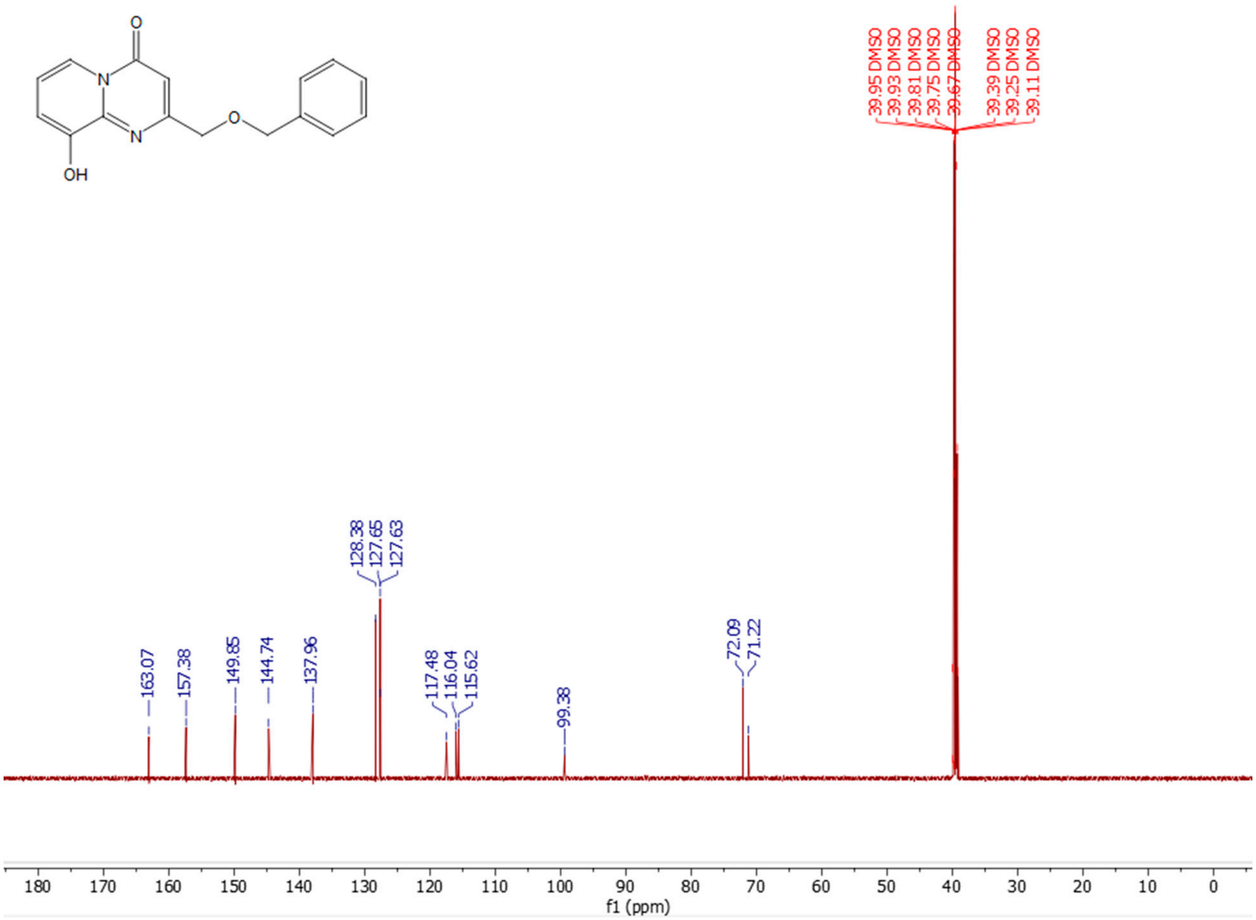

O
N
N
OH
O
39.95 DMSO
39.93 DMSO
39.81 DMSO
39.75 DMSO
39.67 DMSO
39.39 DMSO
39.25 DMSO
39.11 DMSO
163.07
157.38
149.85
144.74
137.96
128.38
127.65
127.63
117.48
116.04
115.62
99.38
72.09
71.22
180
170
160
150
140
130
120
110
100
90
80
70
60
50
40
30
20
10
0
f1 (ppm)

^{1}H NMR of **31**

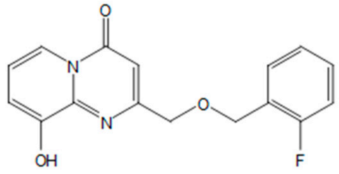

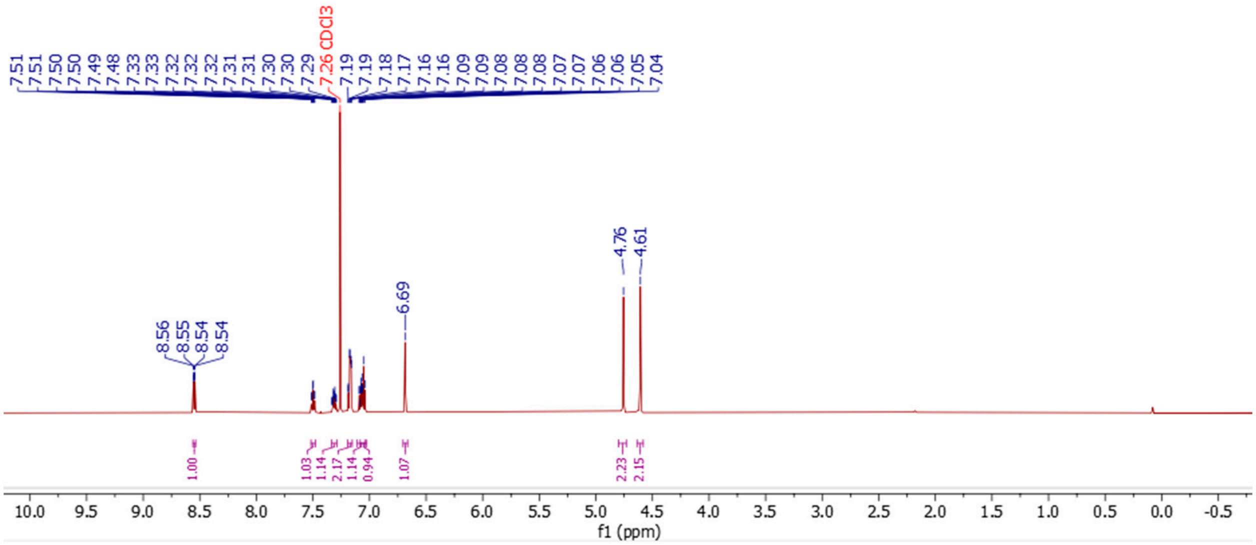

7.51
7.51
7.50
7.49
7.48
7.33
7.33
7.32
7.32
7.32
7.31
7.31
7.30
7.30
7.29
7.26 CDCl3
7.19
7.19
7.18
7.17
7.16
7.16
7.09
7.09
7.08
7.08
7.08
7.07
7.07
7.06
7.06
7.05
7.04
4.76
4.61
6.69
8.56
8.55
8.54
8.54
1.00
1.03
1.14
2.17
1.14
0.94
1.07
2.23
2.15
10.0 9.5 9.0 8.5 8.0 7.5 7.0 6.5 6.0 5.5 5.0 4.5 4.0 3.5 3.0 2.5 2.0 1.5 1.0 0.5 0.0 -0.5
f1 (ppm)

^{13}C NMR of **31**

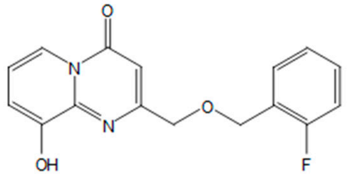

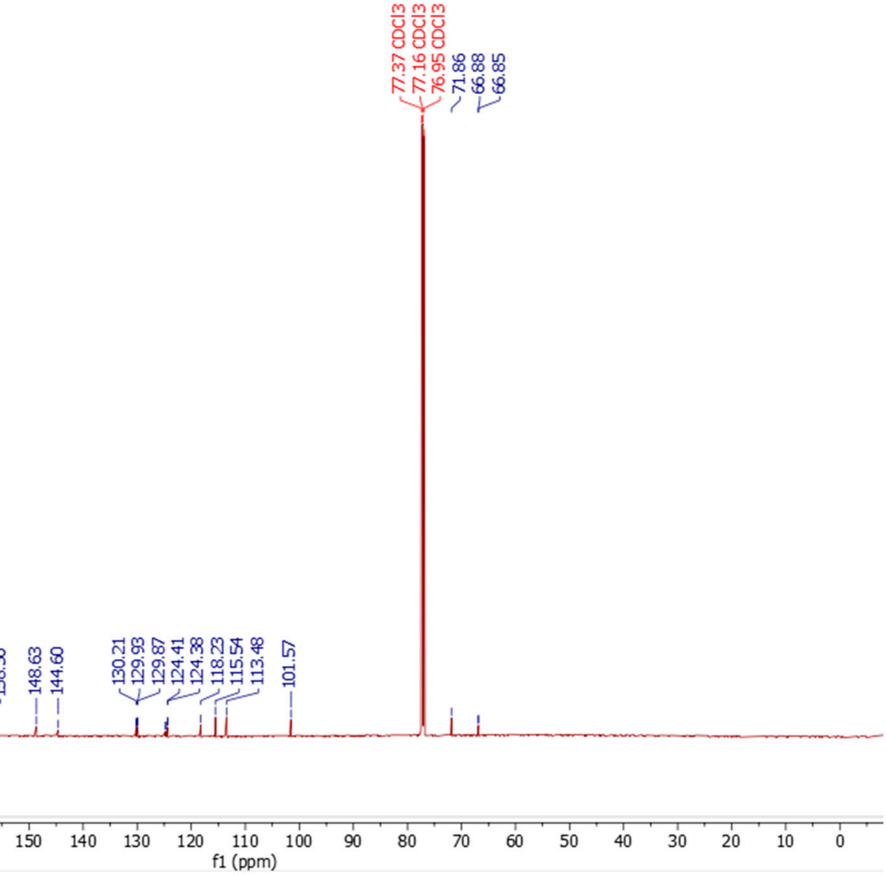
77.37 CDCl3
77.16 CDCl3
76.95 CDCl3
71.86
66.88
66.85
162.88
161.72
160.08
158.30
148.63
144.60
130.21
129.93
129.87
124.41
124.38
118.23
115.54
113.48
101.57
220 210 200 190 180 170 160 150 140 130 120 110 100 90 80 70 60 50 40 30 20 10 0
f1 (ppm)

^{1}H NMR of **32**

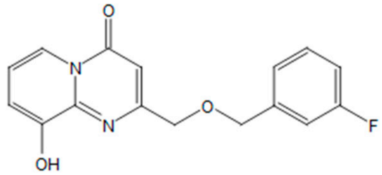

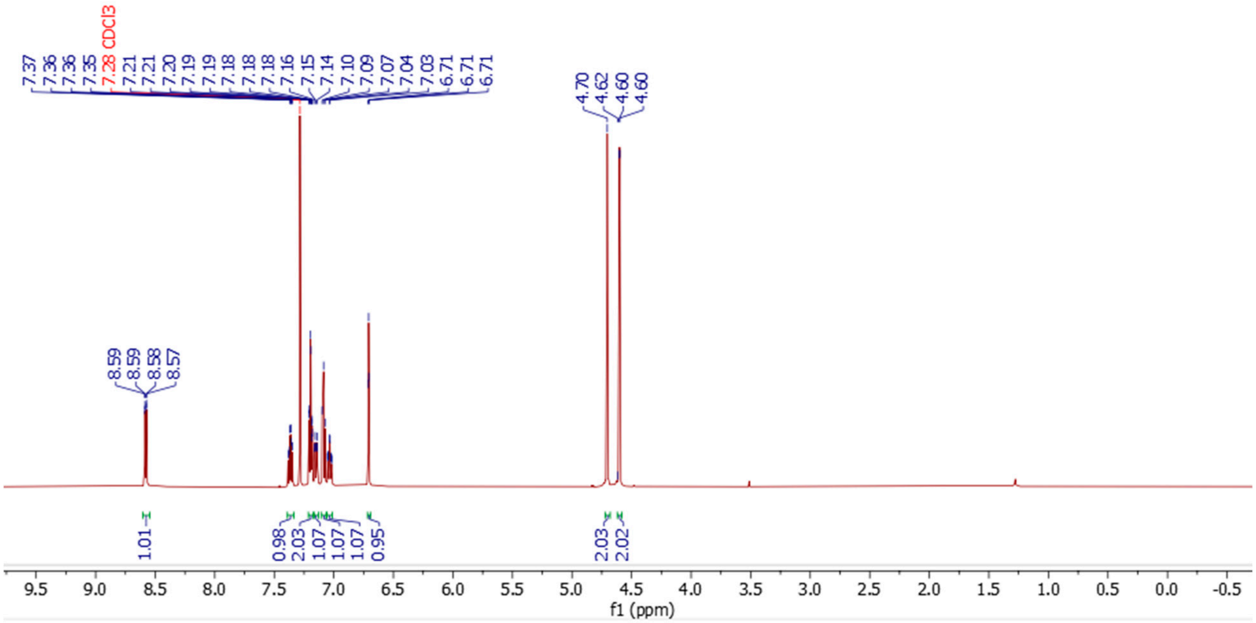

7.37
7.36
7.36
7.35
7.28 CDCl3
7.21
7.21
7.20
7.19
7.19
7.18
7.18
7.18
7.16
7.15
7.14
7.10
7.09
7.07
7.04
7.03
6.71
6.71
6.71
4.70
4.62
4.60
4.60
8.59
8.59
8.58
8.57
1.01
0.98
2.03
1.07
1.07
1.07
0.95
2.03
2.02
9.5
9.0
8.5
8.0
7.5
7.0
6.5
6.0
5.5
5.0
4.5
4.0
3.5
3.0
2.5
2.0
1.5
1.0
0.5
0.0
-0.5
f1 (ppm)

^{13}C NMR of **32**

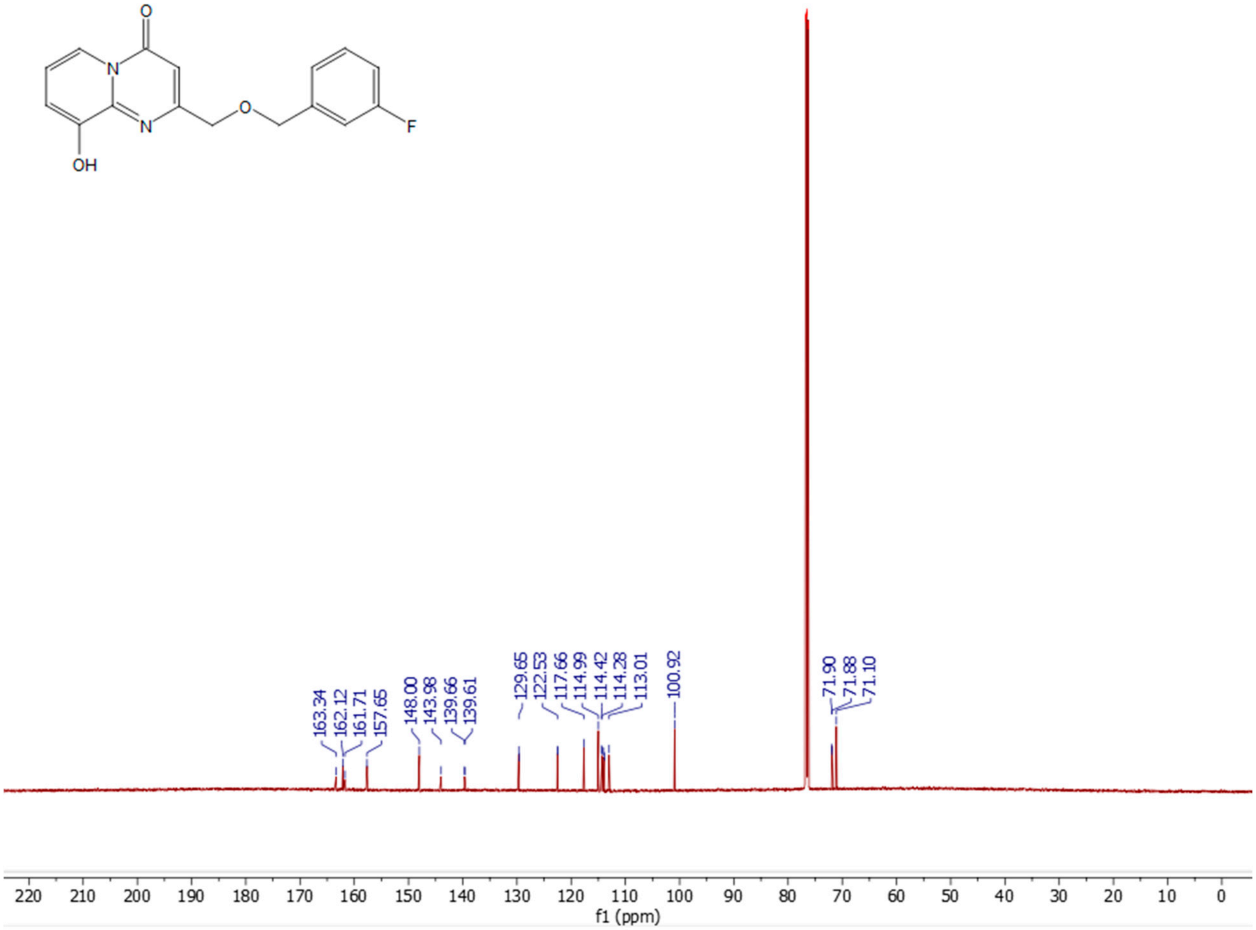

O
N
N
O
F
OH
163.34
162.12
161.71
157.65
148.00
143.98
139.66
139.61
129.65
122.53
117.66
114.99
114.42
114.28
113.01
100.92
71.90
71.88
71.10
220
210
200
190
180
170
160
150
140
130
120
110
100
90
80
70
60
50
40
30
20
10
0
f1 (ppm)

^{1}H NMR of **33**

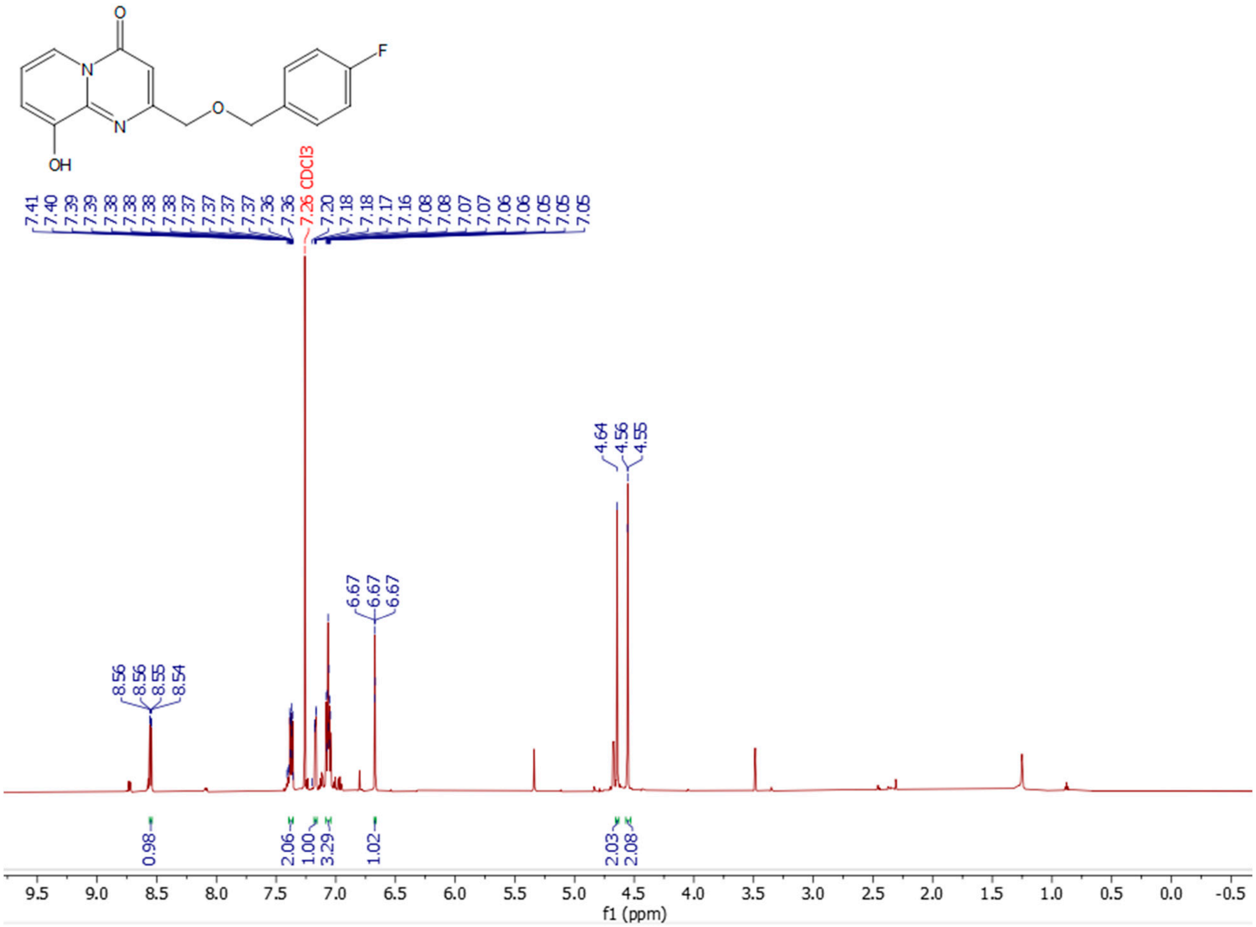

O
N
N
OH
O
F
7.41
7.40
7.39
7.39
7.38
7.38
7.38
7.38
7.37
7.37
7.37
7.36
7.36
7.26 CDCl3
7.20
7.18
7.18
7.17
7.16
7.08
7.08
7.07
7.07
7.06
7.06
7.05
7.05
7.05
4.64
4.56
4.55
6.67
6.67
6.67
8.56
8.56
8.55
8.54
0.98
2.06
1.00
3.29
1.02
2.03
2.08
9.5 9.0 8.5 8.0 7.5 7.0 6.5 6.0 5.5 5.0 4.5 4.0 3.5 3.0 2.5 2.0 1.5 1.0 0.5 0.0 -0.5
f1 (ppm)

^{13}C NMR of **33**

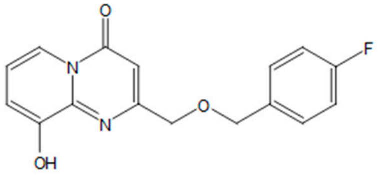
O
N
N
OH
O
F

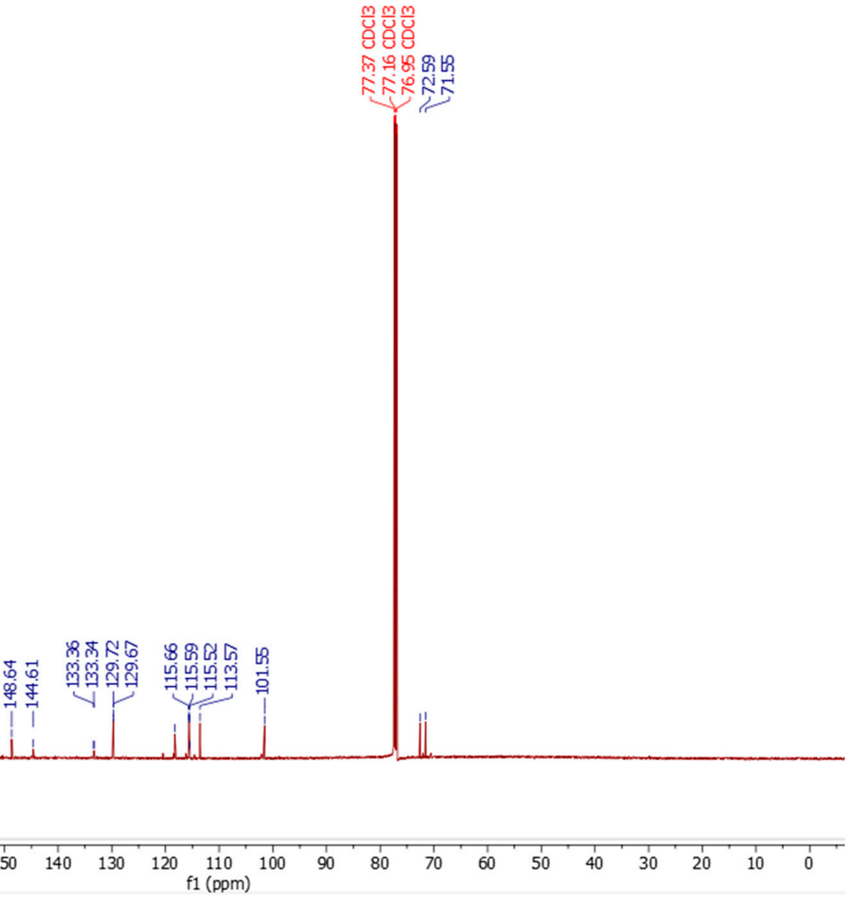
77.37 CDCl3
77.16 CDCl3
76.95 CDCl3
72.59
71.55
163.49
162.91
161.86
158.31
148.64
144.61
133.36
133.34
129.72
129.67
115.66
115.59
115.52
113.57
101.55
220 210 200 190 180 170 160 150 140 130 120 110 100 90 80 70 60 50 40 30 20 10 0
f1 (ppm)

^{1}H NMR of **34**

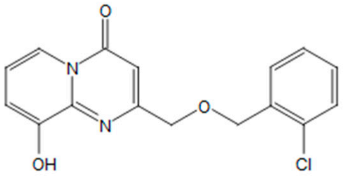

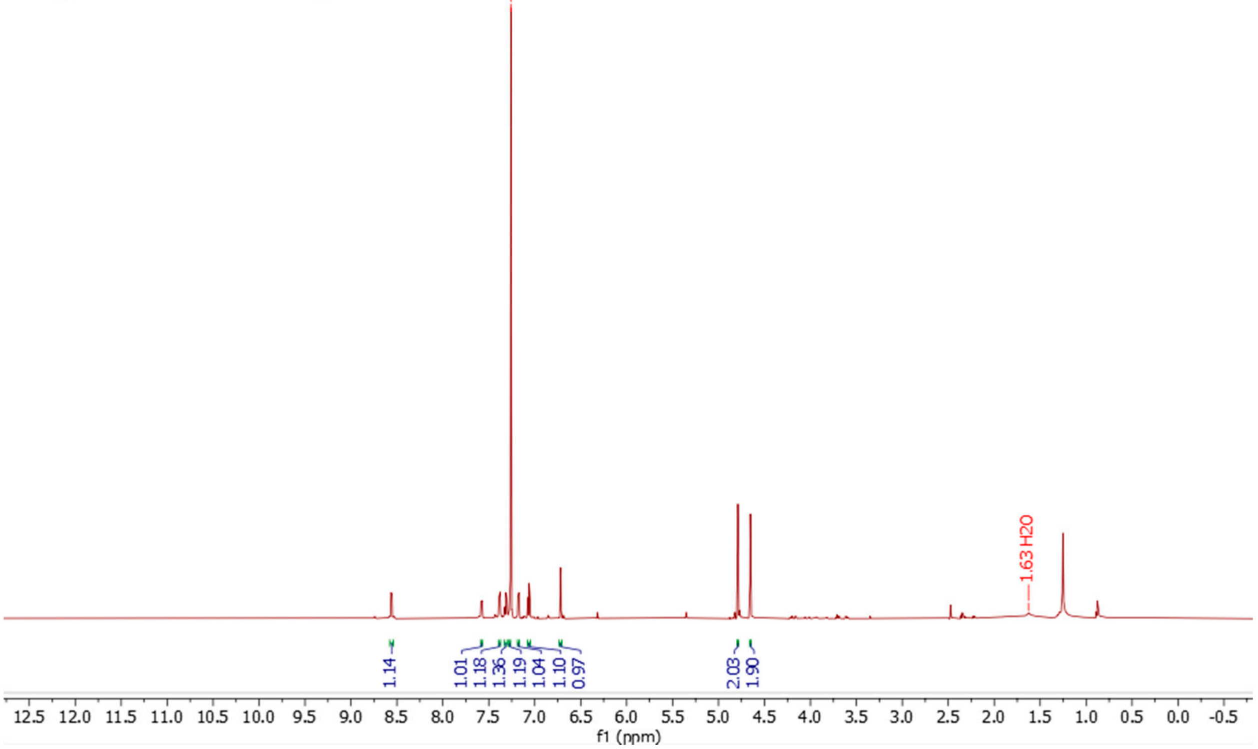
7.26 CDCl3
1.63 H2O
1.14
1.01
1.18
1.36
1.19
1.04
1.10
0.97
2.03
1.90
12.5 12.0 11.5 11.0 10.5 10.0 9.5 9.0 8.5 8.0 7.5 7.0 6.5 6.0 5.5 5.0 4.5 4.0 3.5 3.0 2.5 2.0 1.5 1.0 0.5 0.0 -0.5
f1 (ppm)

^{13}C NMR of **34**

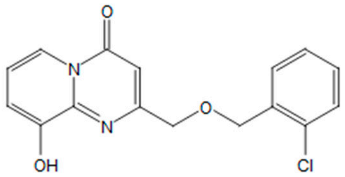

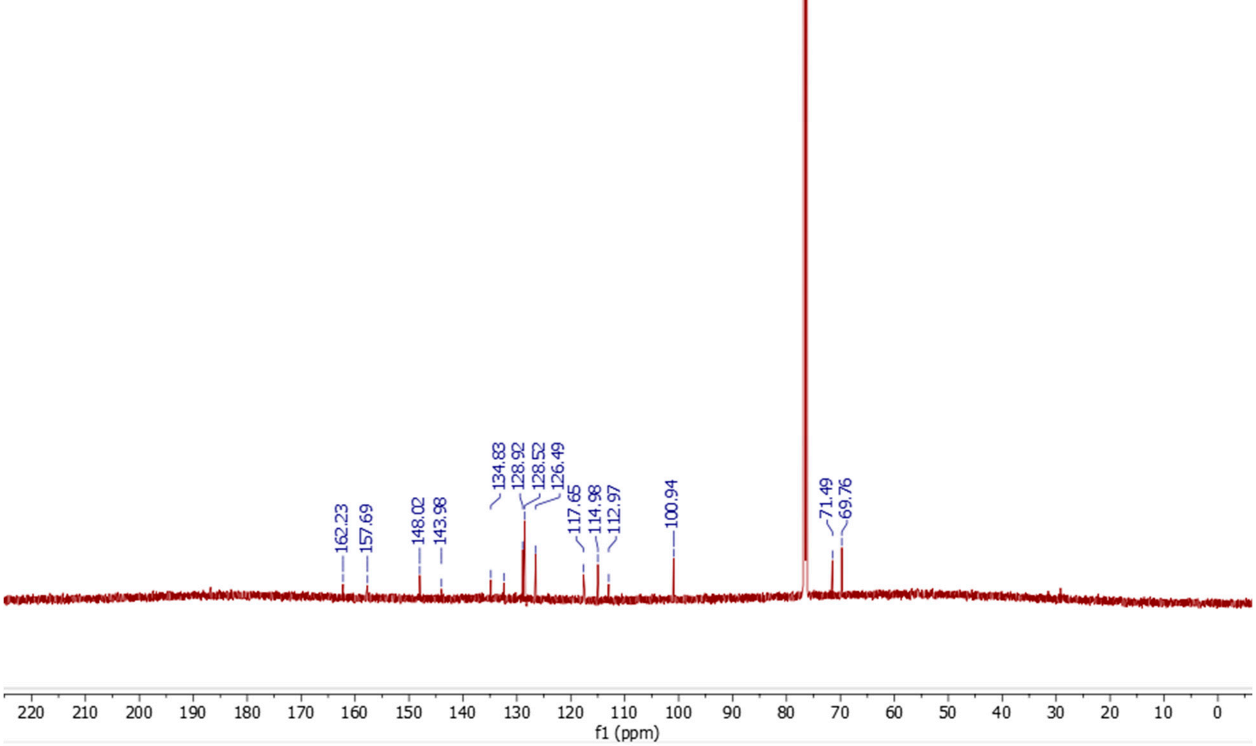

162.23
157.69
148.02
143.98
134.83
128.92
128.52
126.49
117.65
114.98
112.97
100.94
71.49
69.76
220 210 200 190 180 170 160 150 140 130 120 110 100 90 80 70 60 50 40 30 20 10 0
f1 (ppm)

^{1}H NMR of **35**

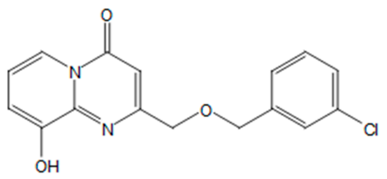

O
N
N
O
Cl
OH

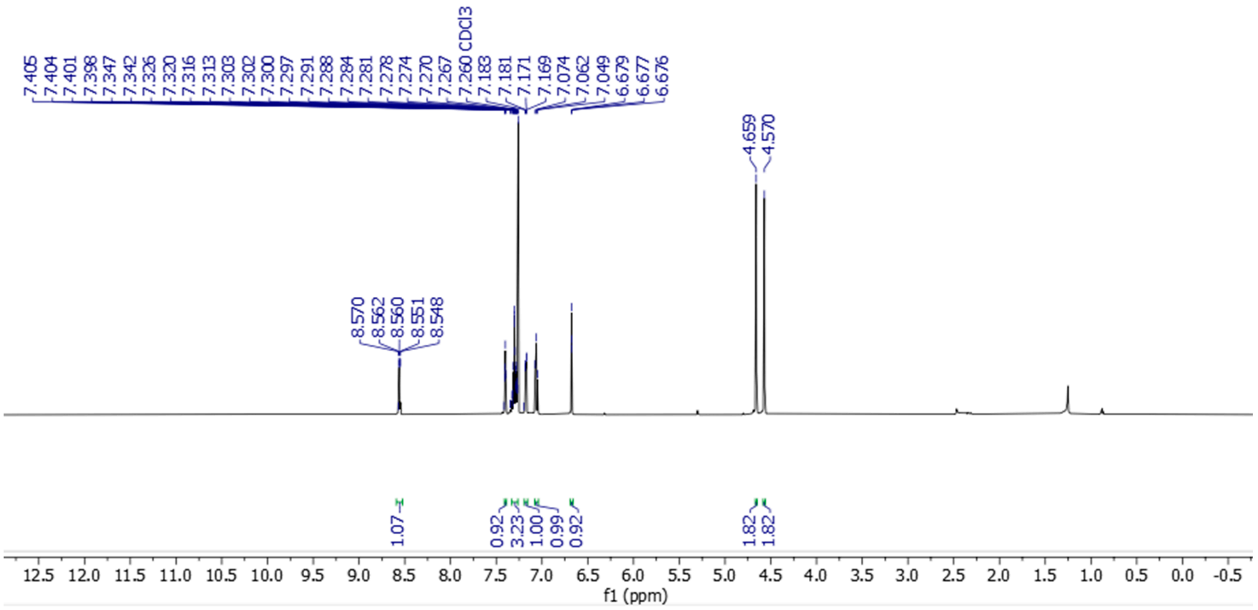

7.405
7.404
7.401
7.398
7.347
7.342
7.326
7.320
7.316
7.313
7.303
7.302
7.300
7.297
7.291
7.288
7.284
7.281
7.278
7.274
7.270
7.267
7.260 CDCl3
7.183
7.181
7.171
7.169
7.074
7.062
7.049
6.679
6.677
6.676
4.659
4.570
8.570
8.562
8.560
8.551
8.548
1.07
0.92
3.23
1.00
0.99
0.92
1.82
1.82
12.5 12.0 11.5 11.0 10.5 10.0 9.5 9.0 8.5 8.0 7.5 7.0 6.5 6.0 5.5 5.0 4.5 4.0 3.5 3.0 2.5 2.0 1.5 1.0 0.5 0.0 -0.5
f1 (ppm)

^{13}C NMR of **35**

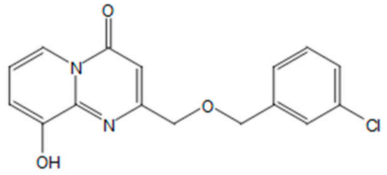

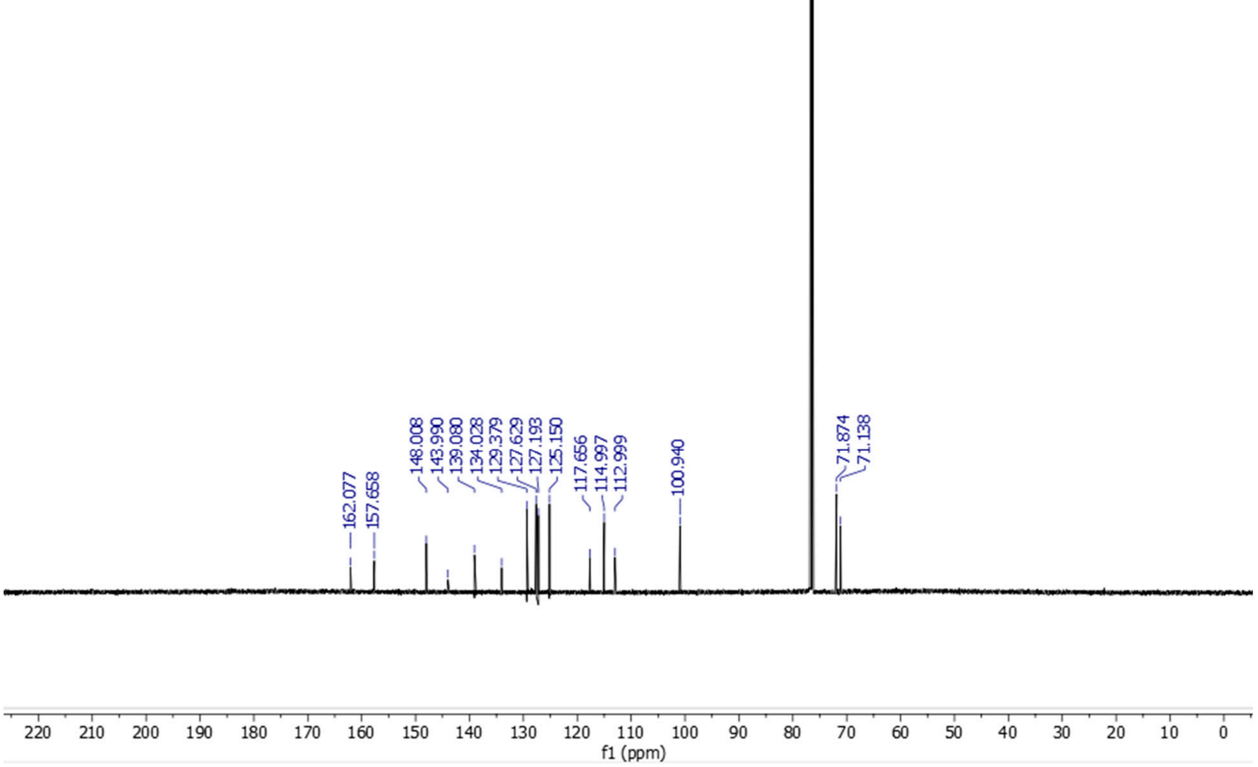

162.077
157.658
148.008
143.990
139.080
134.028
129.379
127.629
127.193
125.150
117.656
114.997
112.999
100.940
71.874
71.138
220 210 200 190 180 170 160 150 140 130 120 110 100 90 80 70 60 50 40 30 20 10 0
f1 (ppm)

^{1}H NMR of **38**

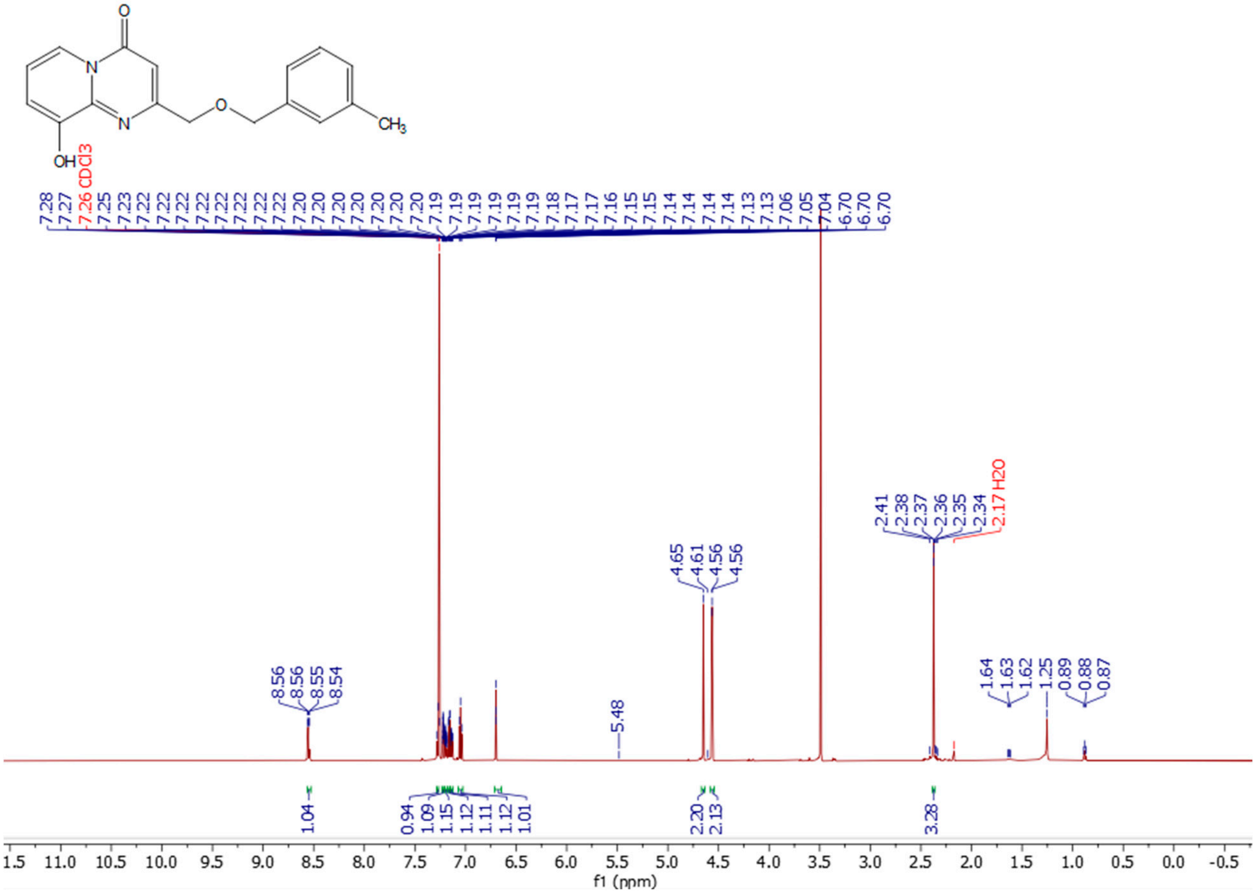

^{13}C NMR of **38**

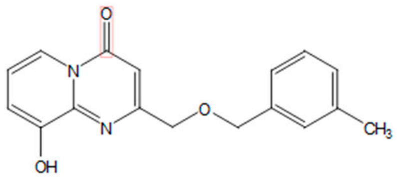
O
N
N
OH
O
CH3

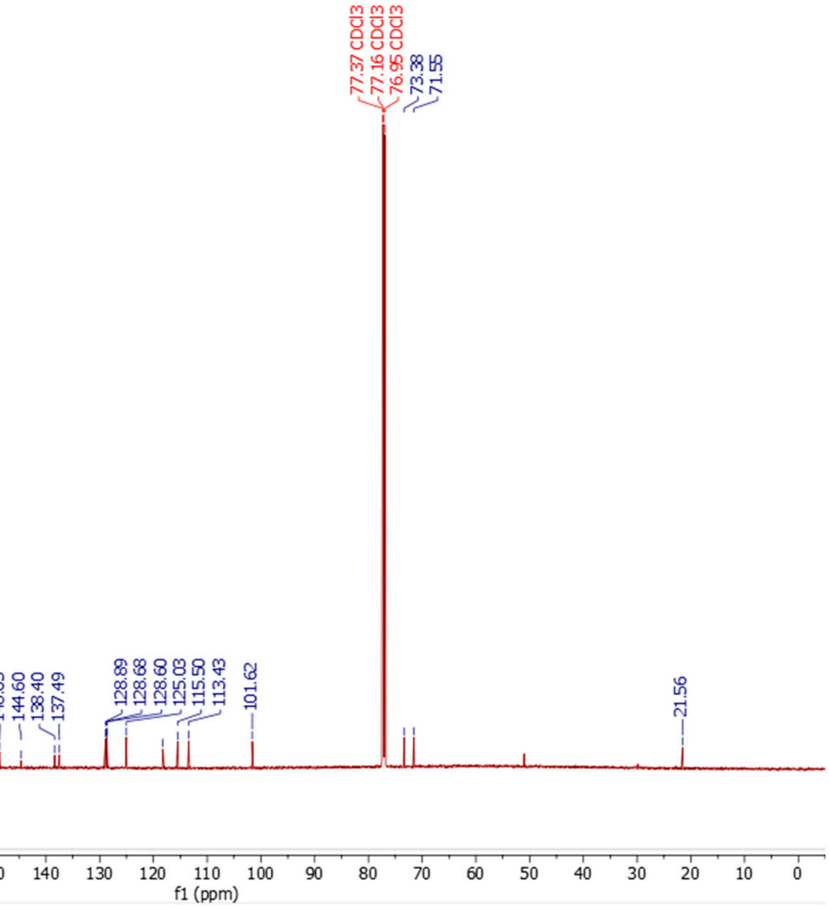
77.37 CDCl3
77.16 CDCl3
76.95 CDCl3
73.38
71.55
163.14
158.33
148.63
144.60
138.40
137.49
128.89
128.68
128.60
125.03
115.50
113.43
101.62
21.56
220
210
200
190
180
170
160
150
140
130
120
110
100
90
80
70
60
50
40
30
20
10
0
f1 (ppm)

^{1}H NMR of **39**

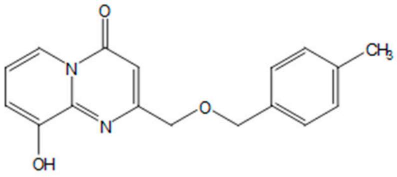

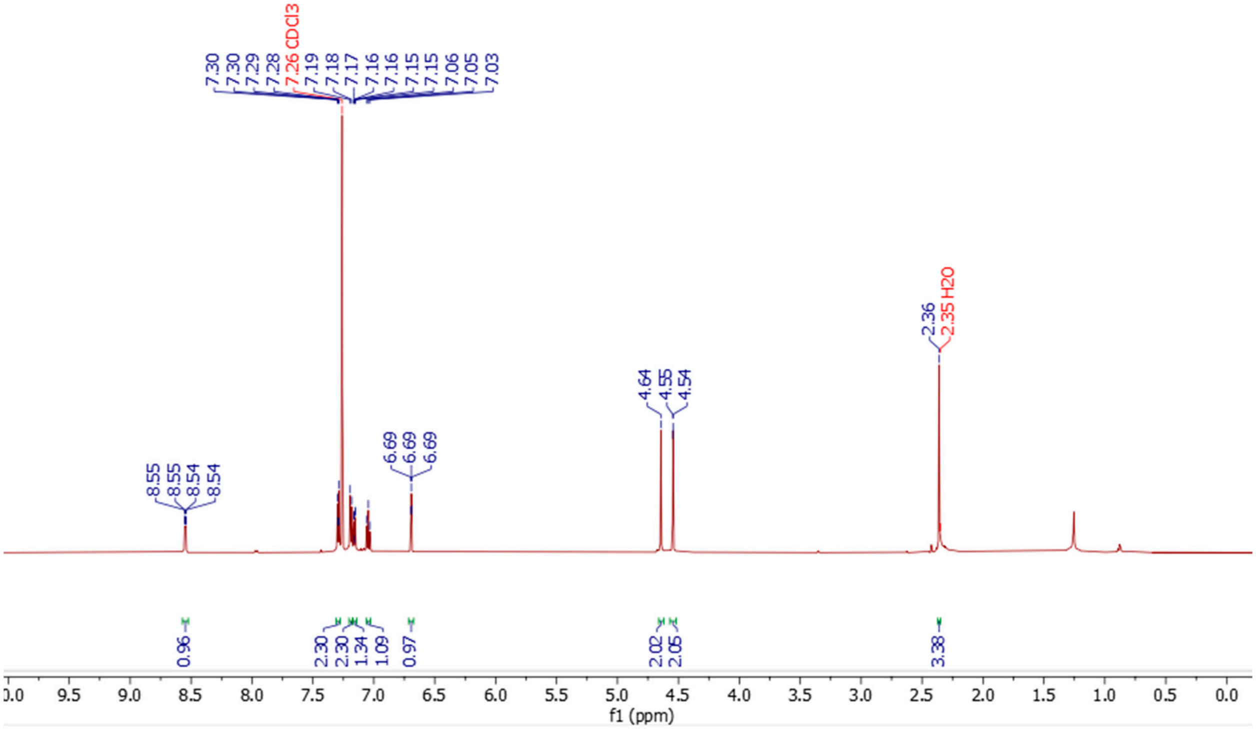

7.30
7.30
7.29
7.28
7.26 CDCl3
7.19
7.18
7.17
7.16
7.16
7.15
7.15
7.06
7.05
7.03
2.36
2.35 H2O
4.64
4.55
4.54
6.69
6.69
6.69
8.55
8.55
8.54
8.54
0.96
2.30
2.30
1.34
1.09
0.97
2.02
2.05
3.38
f1 (ppm)

^{13}C NMR of **39**

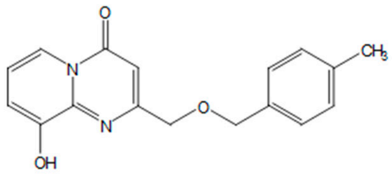

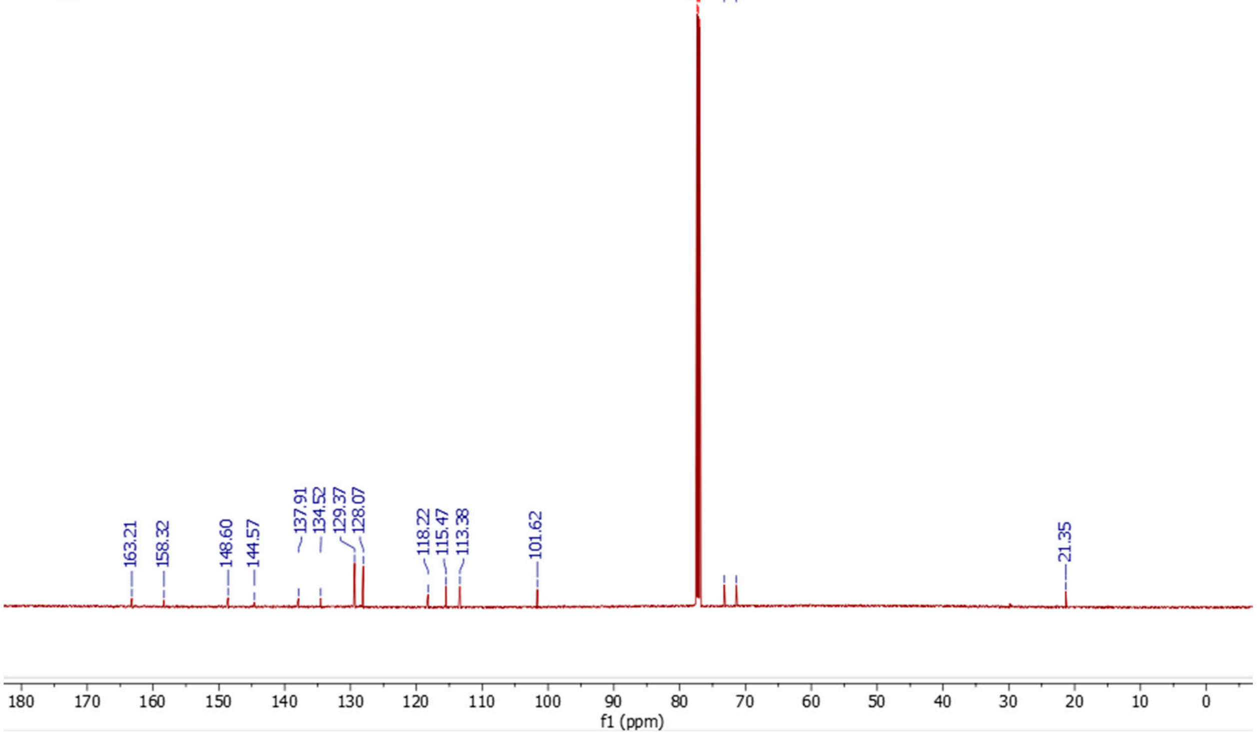
77.37 CDCl3
77.16 CDCl3
76.95 CDCl3
73.20
71.37
163.21
158.32
148.60
144.57
137.91
134.52
129.37
128.07
118.22
115.47
113.38
101.62
21.35
180
170
160
150
140
130
120
110
100
90
80
70
60
50
40
30
20
10
0
f1 (ppm)

^{1}H NMR of **40**

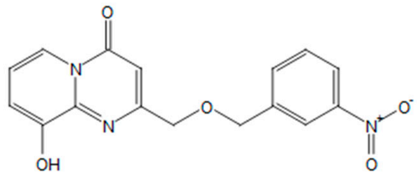

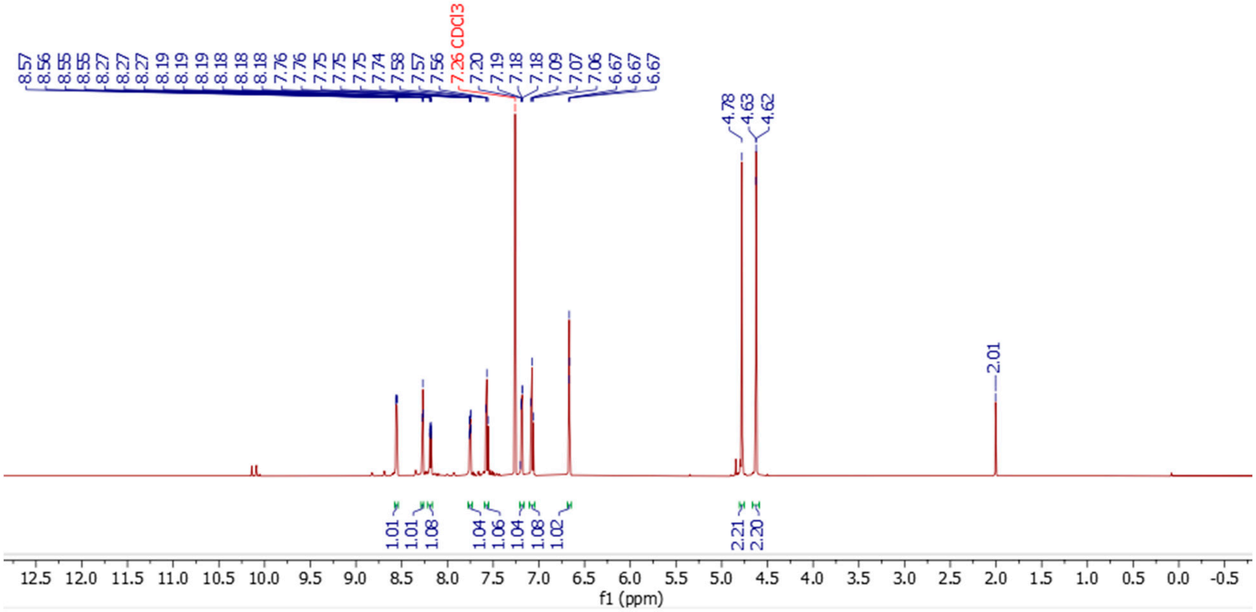

^{13}C NMR of **40**

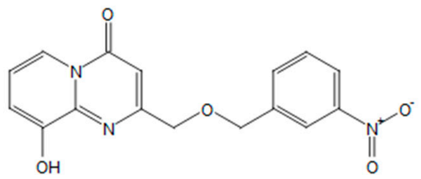
O
N
N
O
OH
N+
O-
O

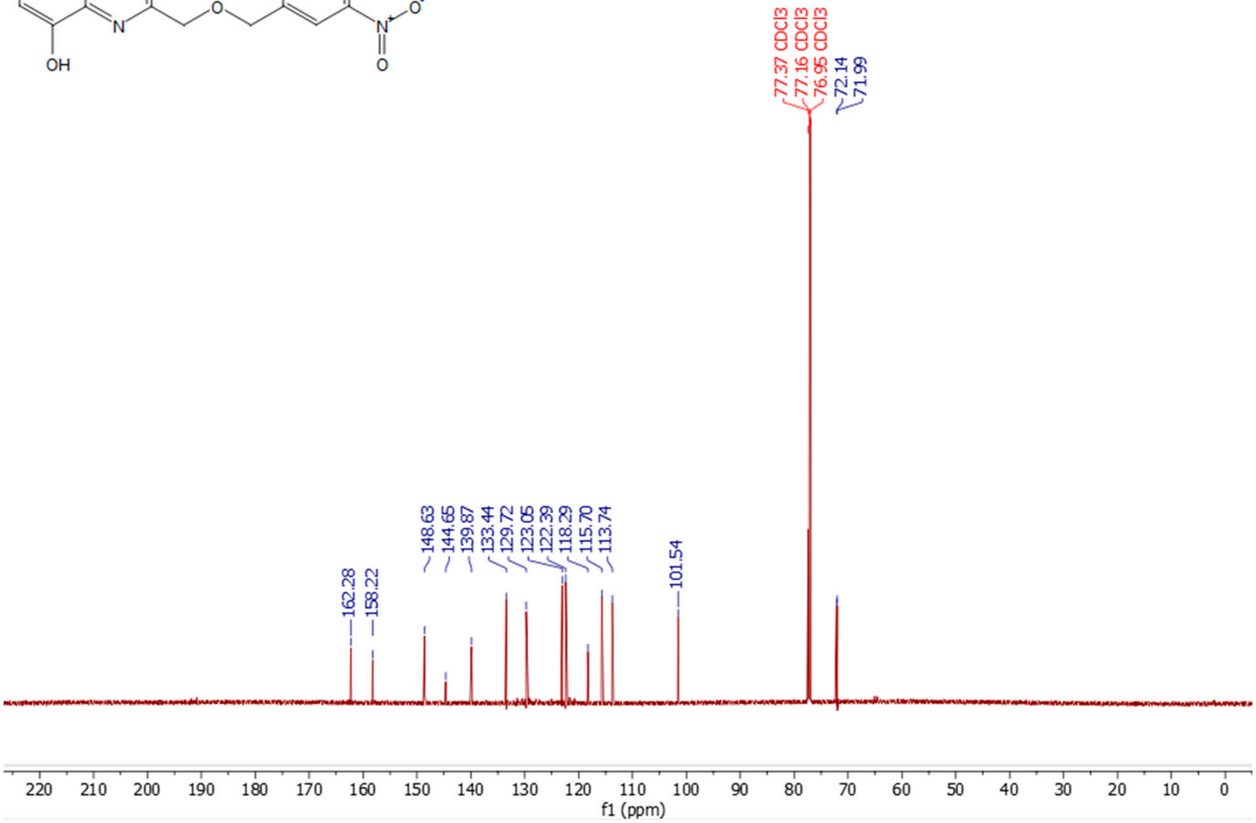
77.37 CDCl3
77.16 CDCl3
76.95 CDCl3
72.14
71.99
162.28
158.22
148.63
144.65
139.87
133.44
129.72
123.05
122.39
118.29
115.70
113.74
101.54
220 210 200 190 180 170 160 150 140 130 120 110 100 90 80 70 60 50 40 30 20 10 0
f1 (ppm)

^{1}H NMR of **41**

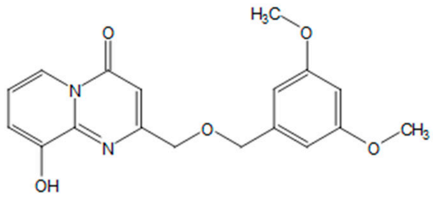

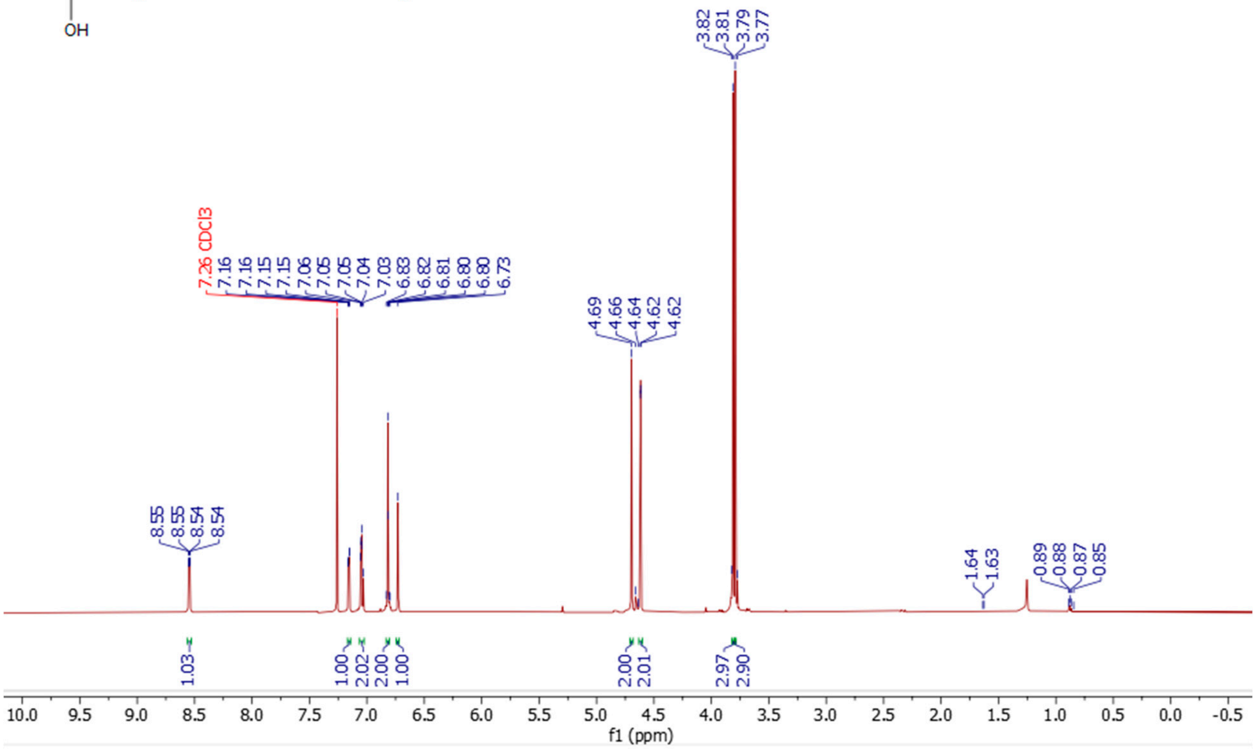

7.26 CDCl3
7.16
7.16
7.15
7.15
7.06
7.05
7.05
7.04
7.03
6.83
6.82
6.81
6.80
6.80
6.73
4.69
4.66
4.64
4.62
4.62
3.82
3.81
3.79
3.77
8.55
8.55
8.54
8.54
1.64
1.63
0.89
0.88
0.87
0.85
1.03
1.00
2.02
2.00
1.00
2.00
2.01
2.97
2.90
10.0
9.5
9.0
8.5
8.0
7.5
7.0
6.5
6.0
5.5
5.0
4.5
4.0
3.5
3.0
2.5
2.0
1.5
1.0
0.5
0.0
-0.5
f1 (ppm)

^{13}C NMR of **41**

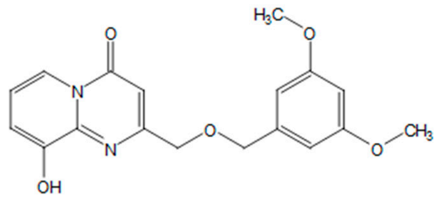

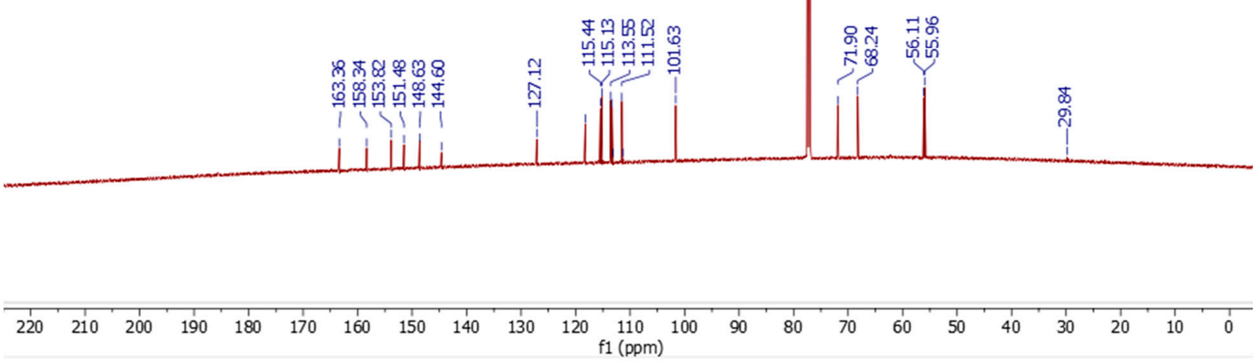
163.36
158.34
153.82
151.48
148.63
144.60
127.12
115.44
115.13
113.55
111.52
101.63
71.90
68.24
56.11
55.96
29.84
220 210 200 190 180 170 160 150 140 130 120 110 100 90 80 70 60 50 40 30 20 10 0
f1 (ppm)

^{1}H NMR of **43**

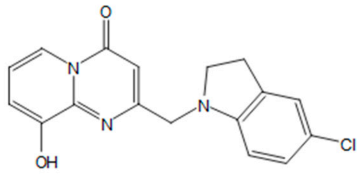

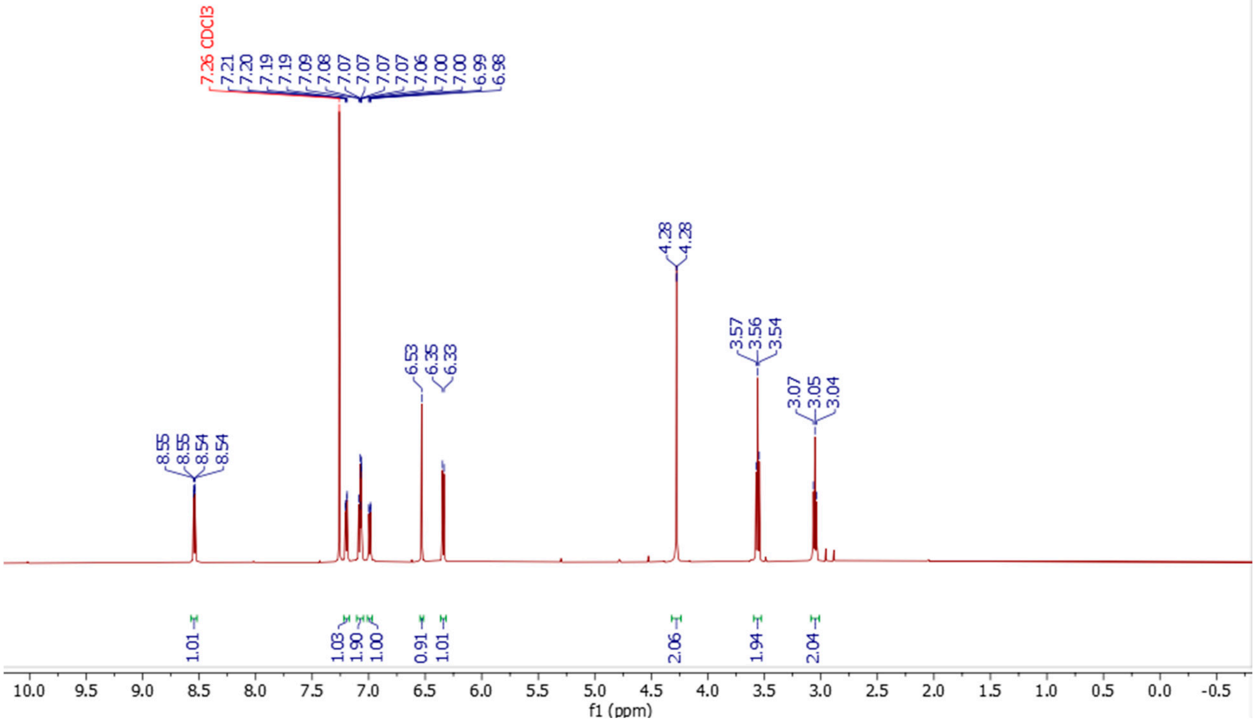

7.26 CDCl3
7.21
7.20
7.19
7.19
7.09
7.08
7.07
7.07
7.07
7.07
7.06
7.00
7.00
6.99
6.98
4.28
4.28
3.57
3.56
3.54
6.53
6.35
6.33
3.07
3.05
3.04
8.55
8.55
8.54
8.54
1.01
1.03
1.90
1.00
0.91
1.01
2.06
1.94
2.04
10.0 9.5 9.0 8.5 8.0 7.5 7.0 6.5 6.0 5.5 5.0 4.5 4.0 3.5 3.0 2.5 2.0 1.5 1.0 0.5 0.0 -0.5
f1 (ppm)

^{13}C NMR of **43**

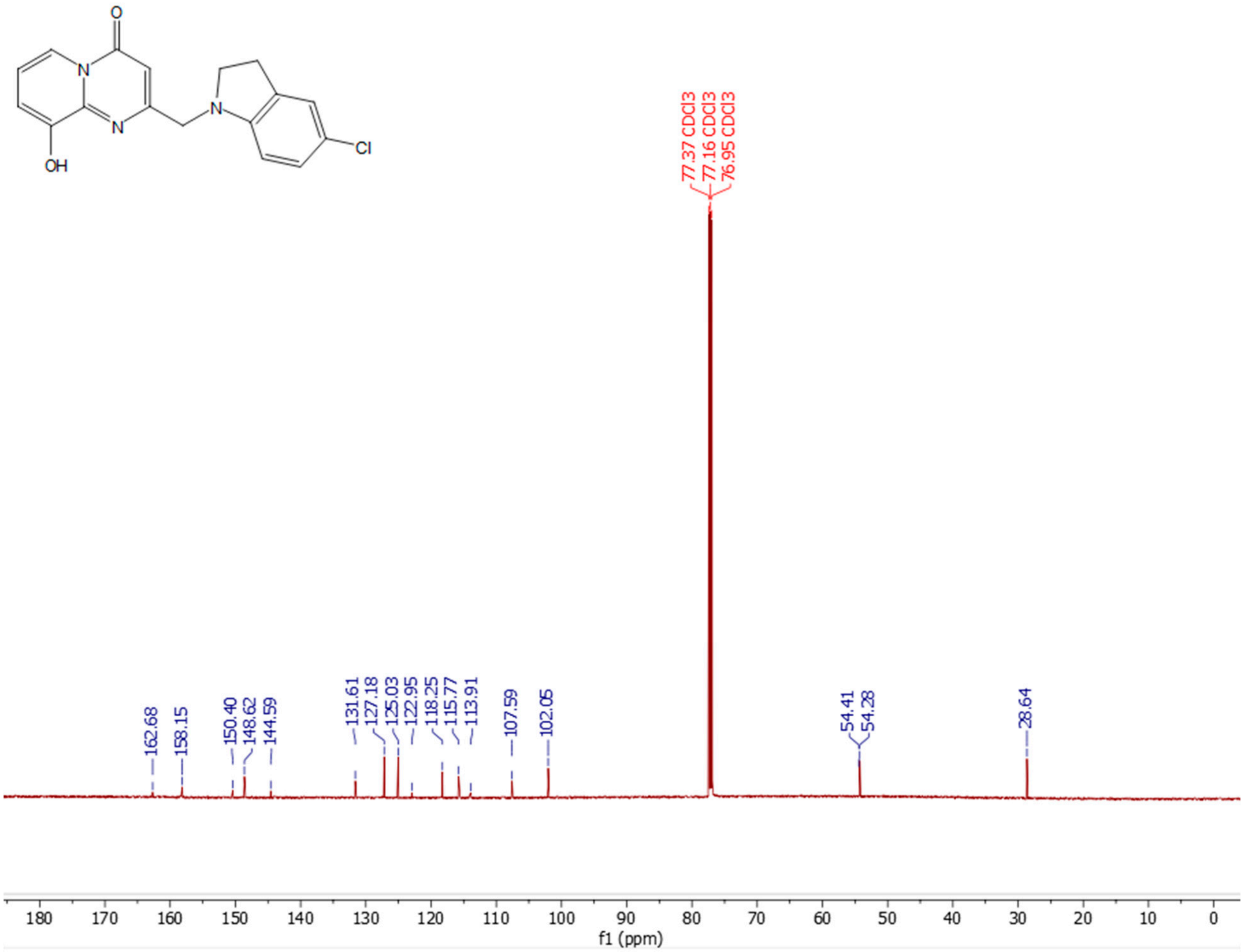

77.37 CDCl3
77.16 CDCl3
76.95 CDCl3
162.68
158.15
150.40
148.62
144.59
131.61
127.18
125.03
122.95
118.25
115.77
113.91
107.59
102.05
54.41
54.28
28.64
180 170 160 150 140 130 120 110 100 90 80 70 60 50 40 30 20 10 0
f1 (ppm)

^{1}H NMR of **44**

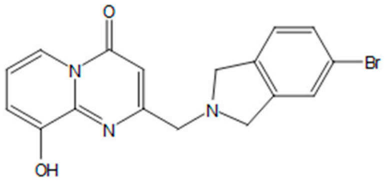

O
N
N
N
Br
OH

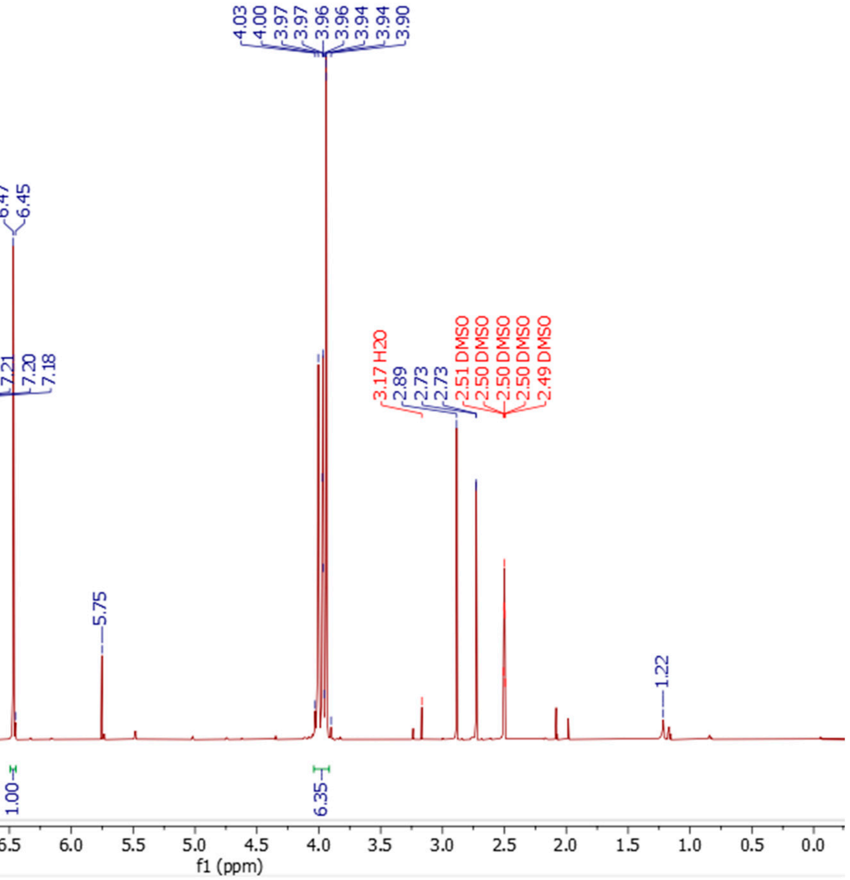

8.48
8.48
8.47
8.47
7.95
7.47
7.46
7.39
7.38
7.37
7.37
7.25
7.25
7.24
7.24
7.22
7.21
7.21
7.20
7.18
6.47
6.45
5.75
4.03
4.00
3.97
3.97
3.96
3.96
3.94
3.94
3.90
3.17 H2O
2.89
2.73
2.73
2.51 DMSO
2.50 DMSO
2.50 DMSO
2.50 DMSO
2.49 DMSO
1.22
1.00
1.02
1.03
0.99
2.05
1.00
6.35
9.5
9.0
8.5
8.0
7.5
7.0
6.5
6.0
5.5
5.0
4.5
4.0
3.5
3.0
2.5
2.0
1.5
1.0
0.5
0.0
f1 (ppm)

^{13}C NMR of **44**

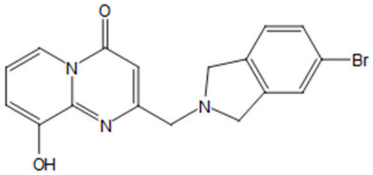

O
N
N
N
Br
OH

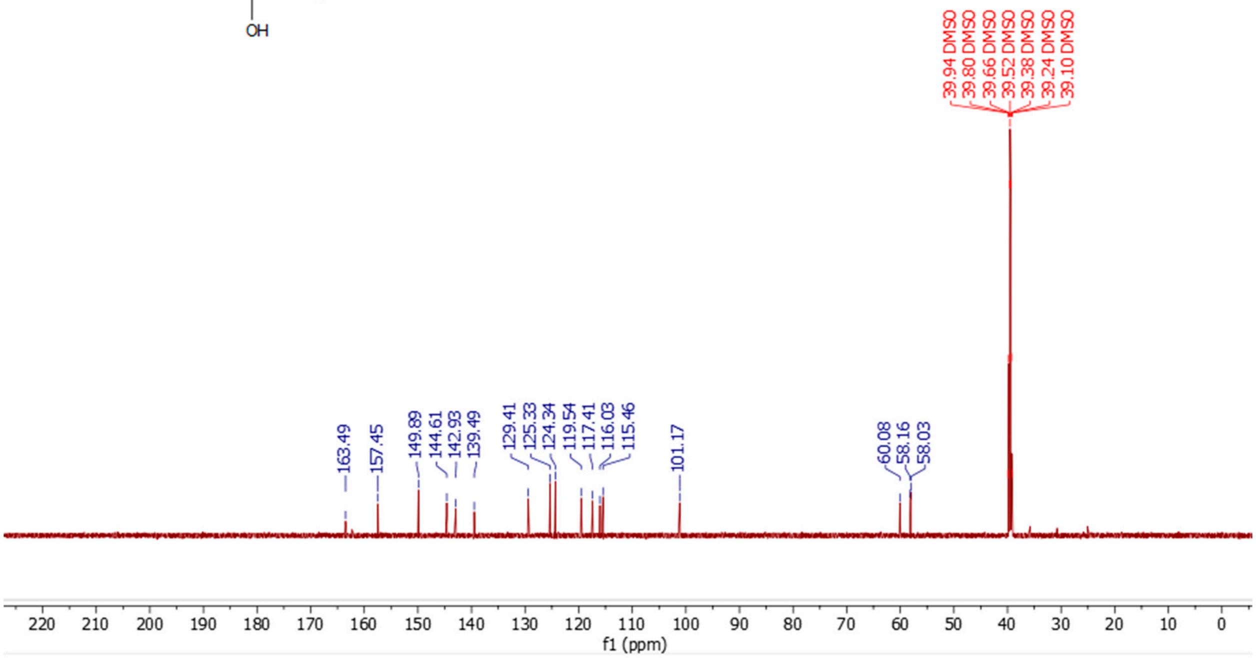

39.94 DMSO
39.80 DMSO
39.66 DMSO
39.52 DMSO
39.38 DMSO
39.24 DMSO
39.10 DMSO
163.49
157.45
149.89
144.61
142.93
139.49
129.41
125.33
124.34
119.54
117.41
116.03
115.46
101.17
60.08
58.16
58.03
220 210 200 190 180 170 160 150 140 130 120 110 100 90 80 70 60 50 40 30 20 10 0
f1 (ppm)

^{1}H NMR of **45**

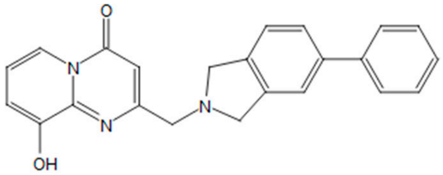

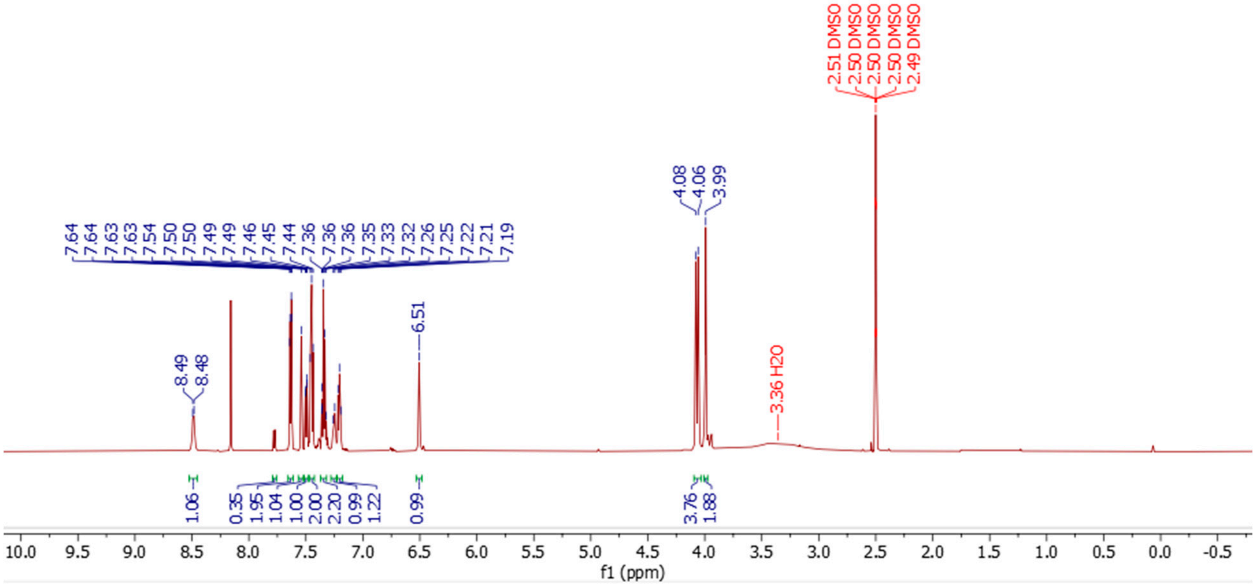

2.51 DMSO
2.50 DMSO
2.50 DMSO
2.50 DMSO
2.49 DMSO
4.08
4.06
3.99
7.64
7.64
7.63
7.63
7.54
7.50
7.50
7.49
7.49
7.46
7.45
7.44
7.36
7.36
7.36
7.35
7.33
7.32
7.26
7.25
7.22
7.21
7.19
6.51
8.49
8.48
3.36 H2O
1.06
0.35
1.95
1.04
1.00
2.00
2.20
0.99
1.22
0.99
3.76
1.88
10.0 9.5 9.0 8.5 8.0 7.5 7.0 6.5 6.0 5.5 5.0 4.5 4.0 3.5 3.0 2.5 2.0 1.5 1.0 0.5 0.0 -0.5
f1 (ppm)

^{13}C NMR of **45**

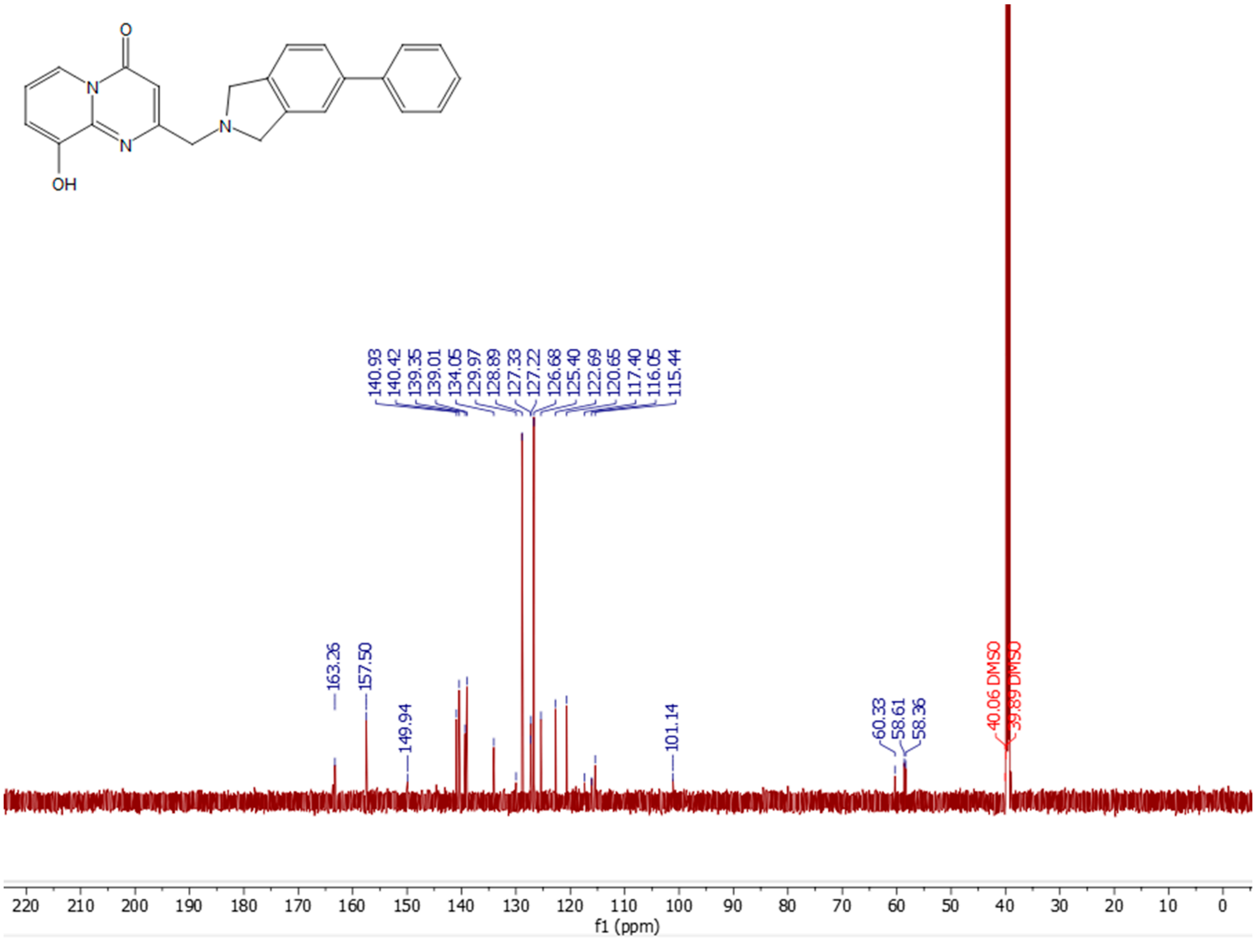

O
N
N
N
OH
140.93
140.42
139.35
139.01
134.05
129.97
128.89
127.33
127.22
126.68
125.40
122.69
120.65
117.40
116.05
115.44
163.26
157.50
149.94
101.14
60.33
58.61
58.36
40.06 DMSO
39.89 DMSO
220 210 200 190 180 170 160 150 140 130 120 110 100 90 80 70 60 50 40 30 20 10 0
f1 (ppm)

^{1}H NMR of **46**

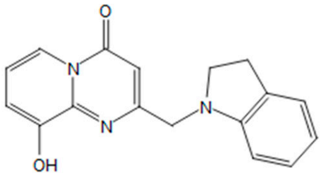
O
N
N
N
OH

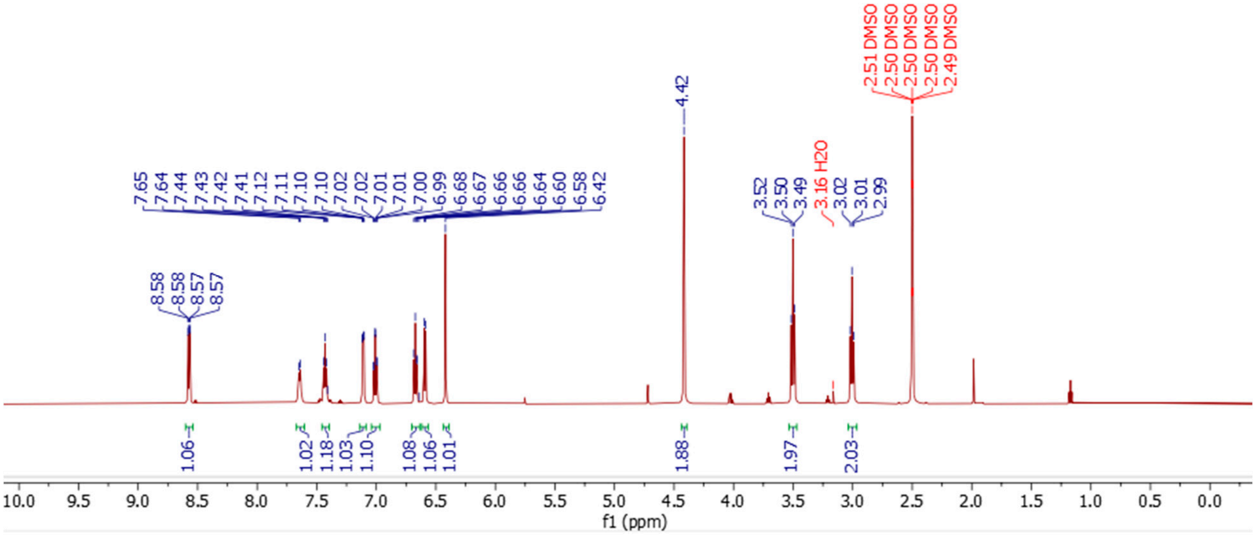
2.51 DMSO
2.50 DMSO
2.50 DMSO
2.50 DMSO
2.49 DMSO
4.42
7.65
7.64
7.44
7.43
7.42
7.41
7.12
7.11
7.10
7.10
7.02
7.02
7.01
7.01
7.00
6.99
6.68
6.67
6.66
6.66
6.64
6.60
6.58
6.42
3.52
3.50
3.49
3.16 H2O
3.02
3.01
2.99
8.58
8.58
8.57
8.57
1.06
1.02
1.18
1.03
1.10
1.08
1.06
1.01
1.88
1.97
2.03
10.0
9.5
9.0
8.5
8.0
7.5
7.0
6.5
6.0
5.5
5.0
4.5
4.0
3.5
3.0
2.5
2.0
1.5
1.0
0.5
0.0
f1 (ppm)

^{13}C NMR of **46**

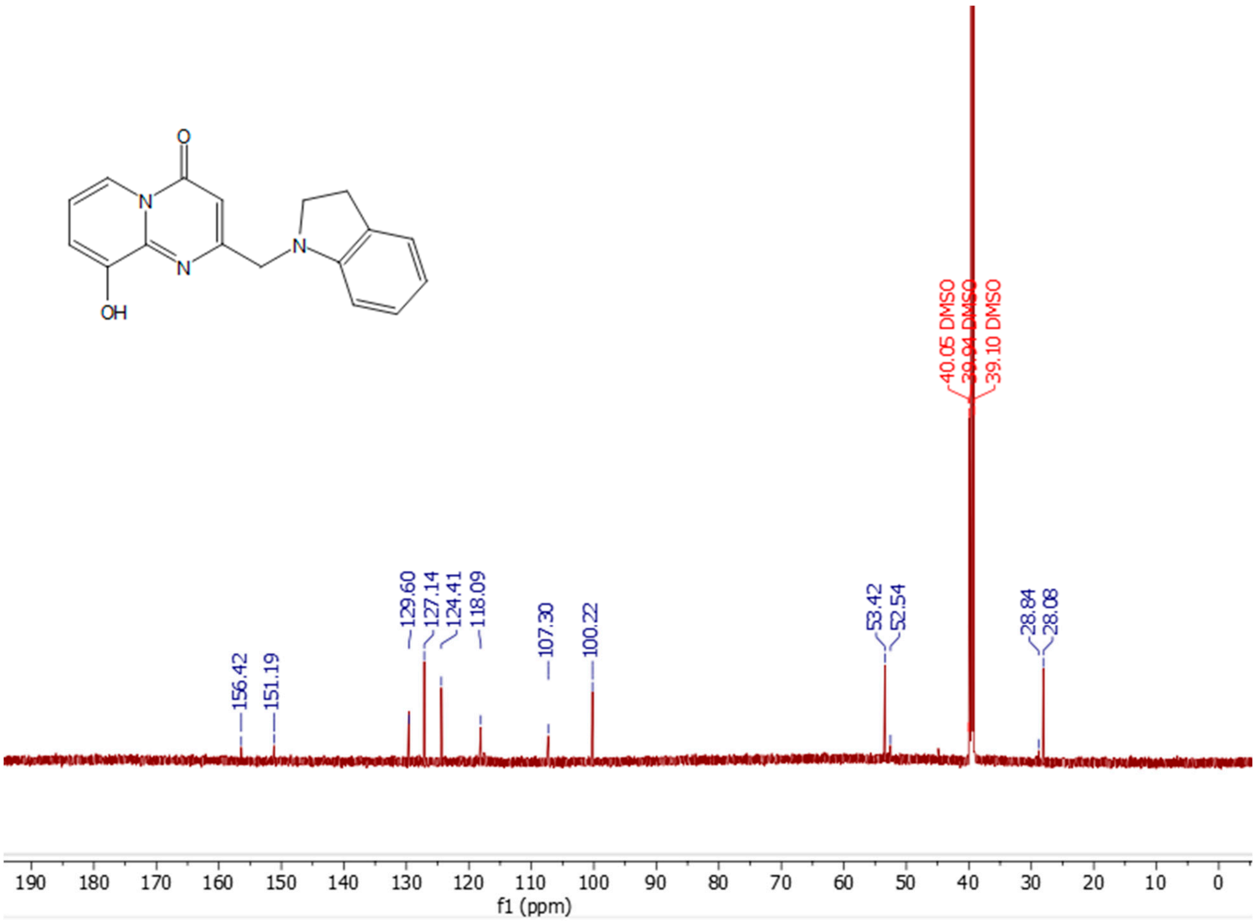

OH
N
N
N
O
156.42
151.19
129.60
127.14
124.41
118.09
107.30
100.22
53.42
52.54
40.05 DMSO
39.94 DMSO
39.10 DMSO
28.84
28.08
190
180
170
160
150
140
130
120
110
100
90
80
70
60
50
40
30
20
10
0
f1 (ppm)

^{1}H NMR of **47**

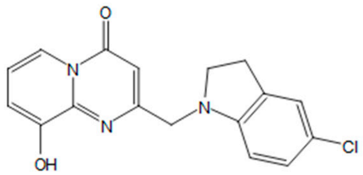
O
N
N
N
OH
Cl

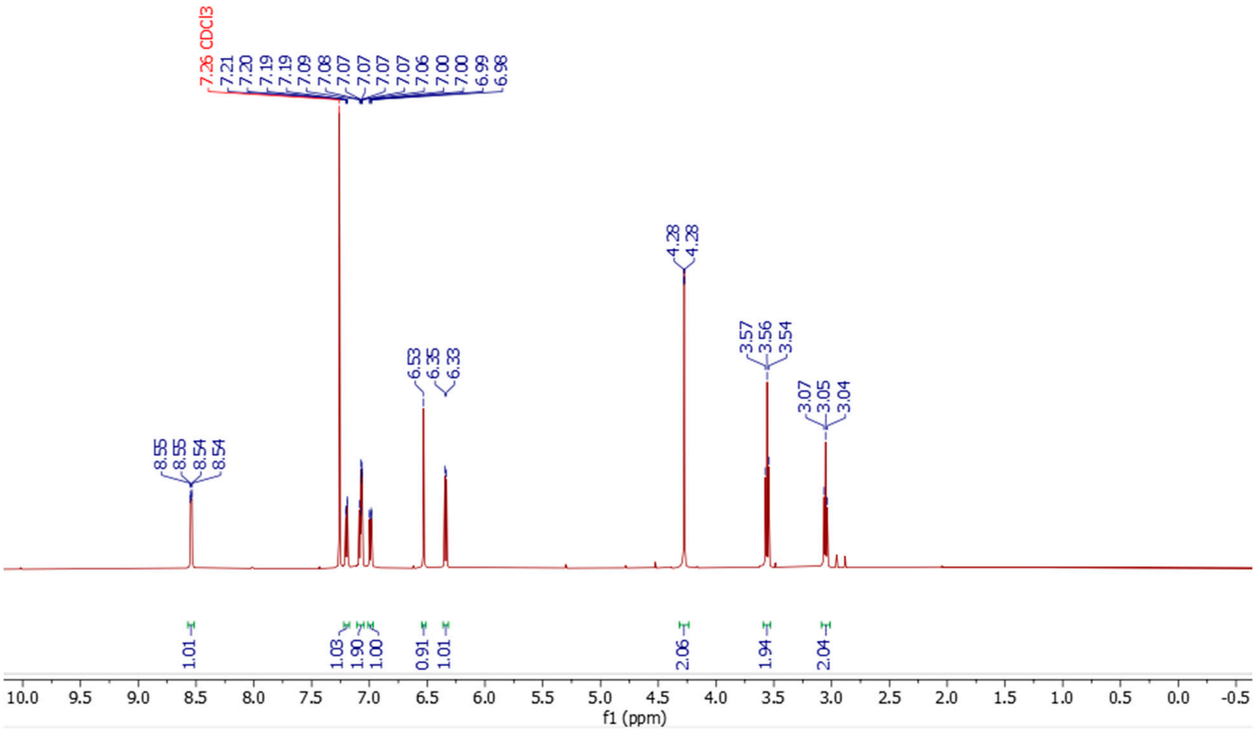
7.26 CDCl3
7.21
7.20
7.19
7.19
7.09
7.08
7.07
7.07
7.07
7.07
7.06
7.00
7.00
7.00
6.99
6.98
4.28
4.28
3.57
3.56
3.54
6.53
6.35
6.33
3.07
3.05
3.04
8.55
8.55
8.54
8.54
1.01
1.03
1.90
1.00
0.91
1.01
2.06
1.94
2.04
10.0
9.5
9.0
8.5
8.0
7.5
7.0
6.5
6.0
5.5
5.0
4.5
4.0
3.5
3.0
2.5
2.0
1.5
1.0
0.5
0.0
-0.5
f1 (ppm)

^{13}C NMR of **47**

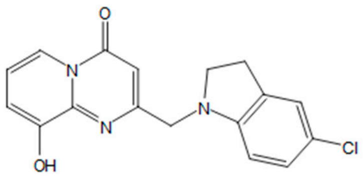

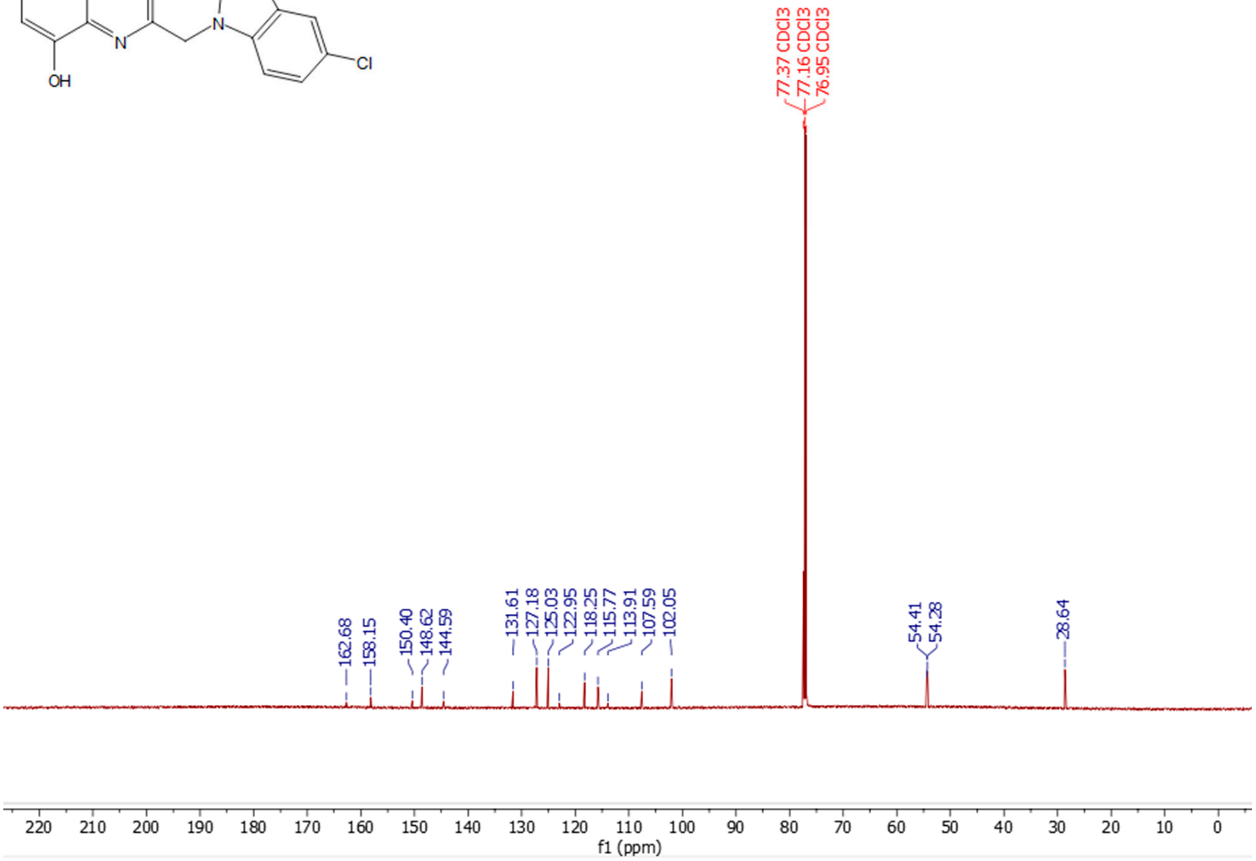
77.37 CDCl3
77.16 CDCl3
76.95 CDCl3
162.68
158.15
150.40
148.62
144.59
131.61
127.18
125.03
122.95
118.25
115.77
113.91
107.59
102.05
54.41
54.28
28.64
220 210 200 190 180 170 160 150 140 130 120 110 100 90 80 70 60 50 40 30 20 10 0
f1 (ppm)

^{1}H NMR of **48**

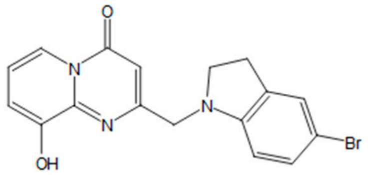

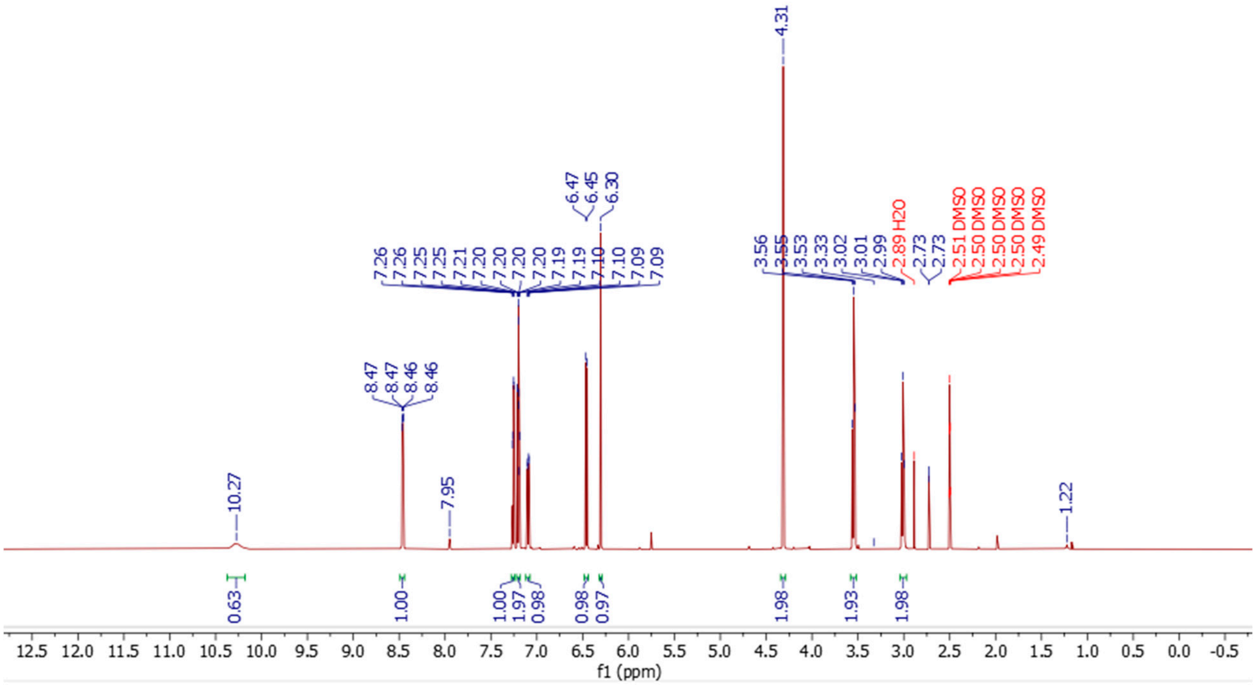

4.31
6.47
6.45
6.30
7.26
7.26
7.25
7.25
7.21
7.20
7.20
7.20
7.20
7.19
7.19
7.10
7.10
7.09
7.09
3.56
3.55
3.53
3.33
3.02
3.01
2.99
2.89 H2O
2.73
2.73
2.51 DMSO
2.50 DMSO
2.50 DMSO
2.50 DMSO
2.49 DMSO
8.47
8.47
8.46
8.46
10.27
7.95
1.22
0.63
1.00
1.00
1.97
0.98
0.98
0.97
1.98
1.93
1.98
12.5 12.0 11.5 11.0 10.5 10.0 9.5 9.0 8.5 8.0 7.5 7.0 6.5 6.0 5.5 5.0 4.5 4.0 3.5 3.0 2.5 2.0 1.5 1.0 0.5 0.0 -0.5
f1 (ppm)

^{13}C NMR of **48**

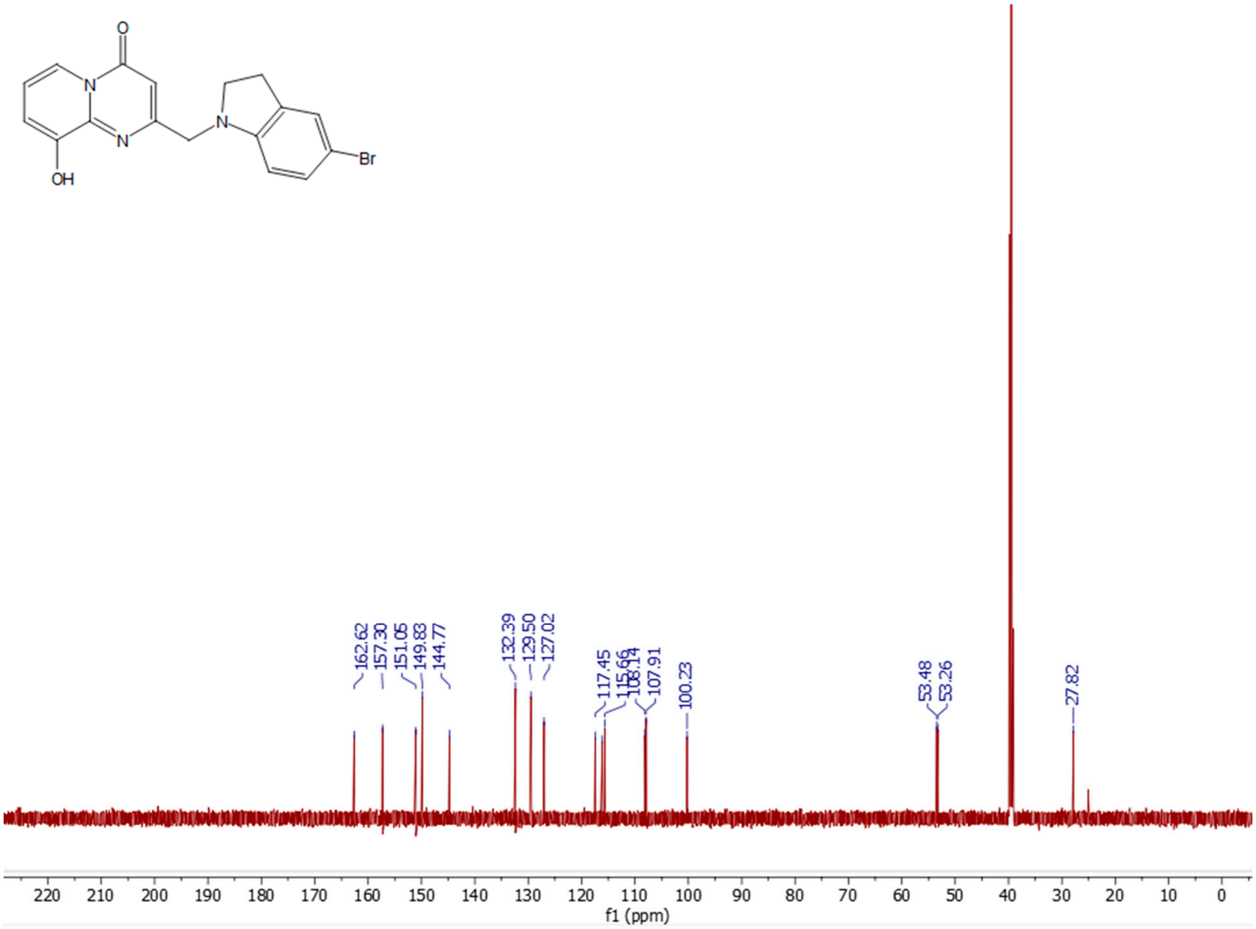

O
N
N
OH
N
Br
162.62
157.30
151.05
149.83
144.77
132.39
129.50
127.02
117.45
115.66
107.91
100.23
53.48
53.26
27.82
220
210
200
190
180
170
160
150
140
130
120
110
100
90
80
70
60
50
40
30
20
10
0
f1 (ppm)

^{1}H NMR of **49**

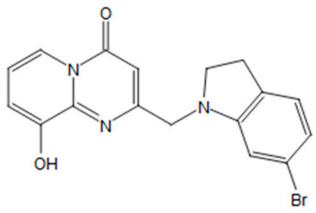

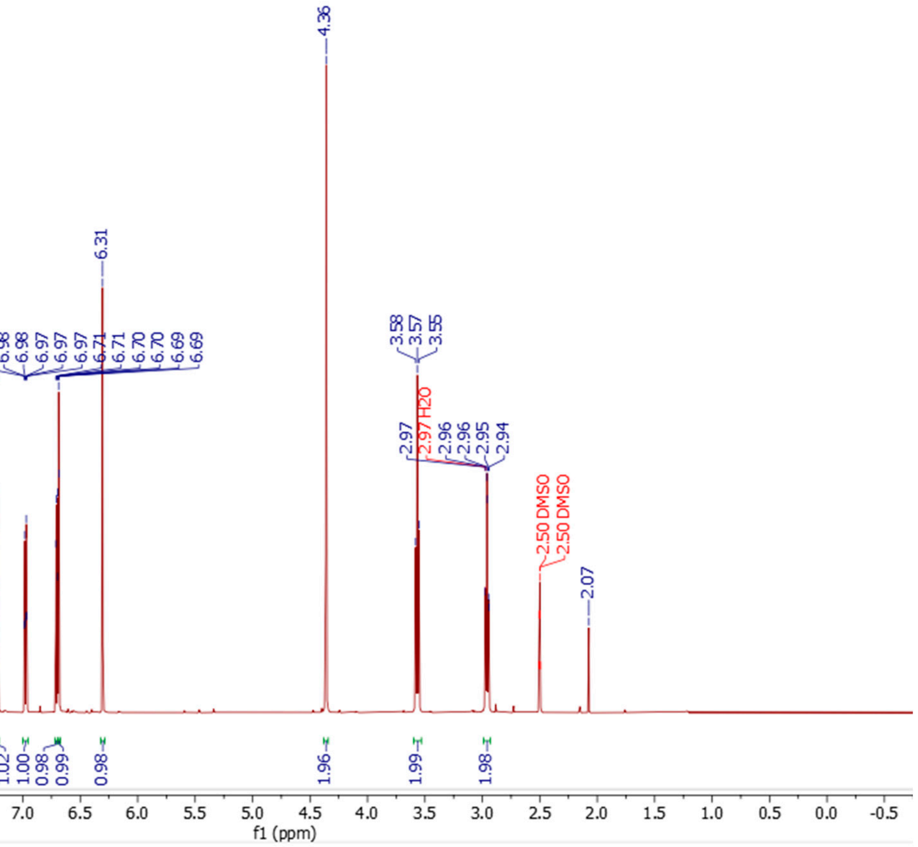

4.36
6.31
7.30
7.30
7.29
7.29
7.24
7.23
7.22
6.98
6.98
6.98
6.97
6.97
6.97
6.71
6.71
6.70
6.70
6.69
6.69
3.58
3.57
3.55
2.97
2.97 H2O
2.96
2.96
2.95
2.94
8.48
8.48
8.47
8.47
2.50 DMSO
2.50 DMSO
2.07
1.00
1.03
1.02
1.00
0.98
0.99
0.98
1.96
1.99
1.98
10.0
9.5
9.0
8.5
8.0
7.5
7.0
6.5
6.0
5.5
5.0
4.5
4.0
3.5
3.0
2.5
2.0
1.5
1.0
0.5
0.0
-0.5
f1 (ppm)

^{13}C NMR of **49**

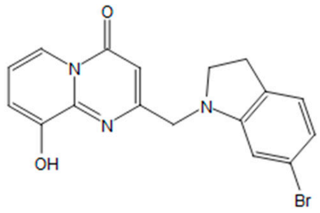
O
N
N
N
OH
Br

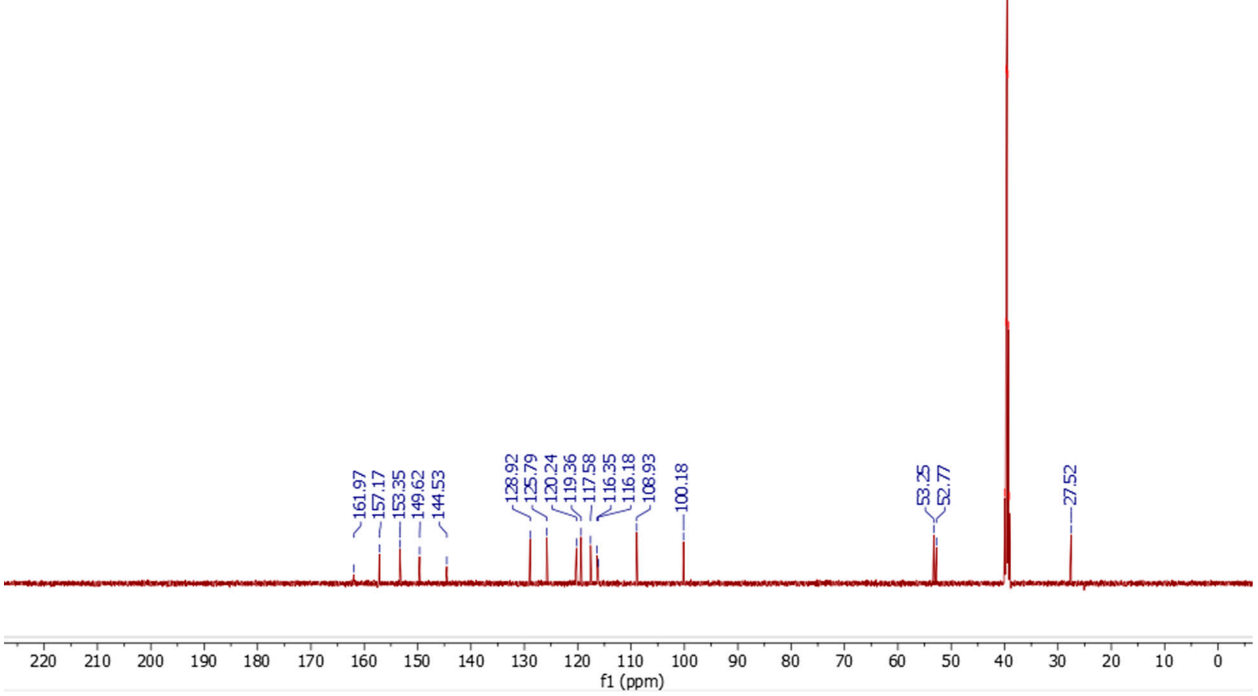
39.94 DMSO
39.80 DMSO
39.66 DMSO
39.52 DMSO
39.38 DMSO
39.24 DMSO
39.10 DMSO
161.97
157.17
153.35
149.62
144.53
128.92
125.79
120.24
119.36
117.58
116.35
116.18
108.93
100.18
53.25
52.77
27.52
220 210 200 190 180 170 160 150 140 130 120 110 100 90 80 70 60 50 40 30 20 10 0
f1 (ppm)

^{1}H NMR of **50**

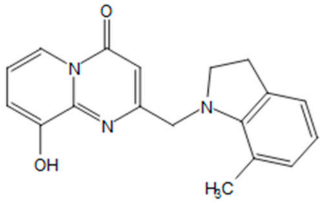

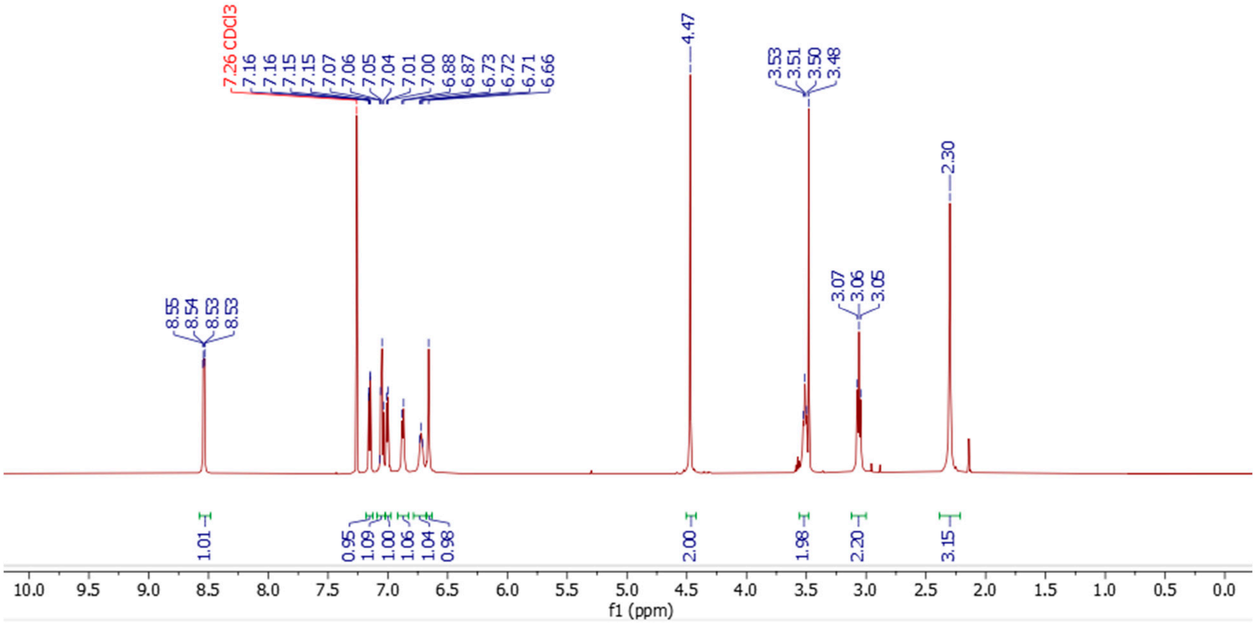

7.26 CDCl3
7.16
7.16
7.15
7.15
7.07
7.06
7.05
7.04
7.01
7.00
6.88
6.87
6.73
6.72
6.71
6.66
4.47
3.53
3.51
3.50
3.48
2.30
3.07
3.06
3.05
8.55
8.54
8.53
8.53
1.01
0.95
1.09
1.00
1.06
1.04
0.98
2.00
1.98
2.20
3.15
10.0 9.5 9.0 8.5 8.0 7.5 7.0 6.5 6.0 5.5 5.0 4.5 4.0 3.5 3.0 2.5 2.0 1.5 1.0 0.5 0.0
f1 (ppm)

^{13}C NMR of **50**

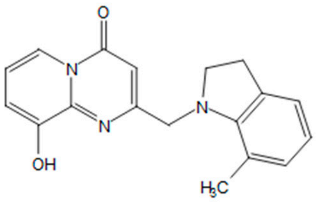

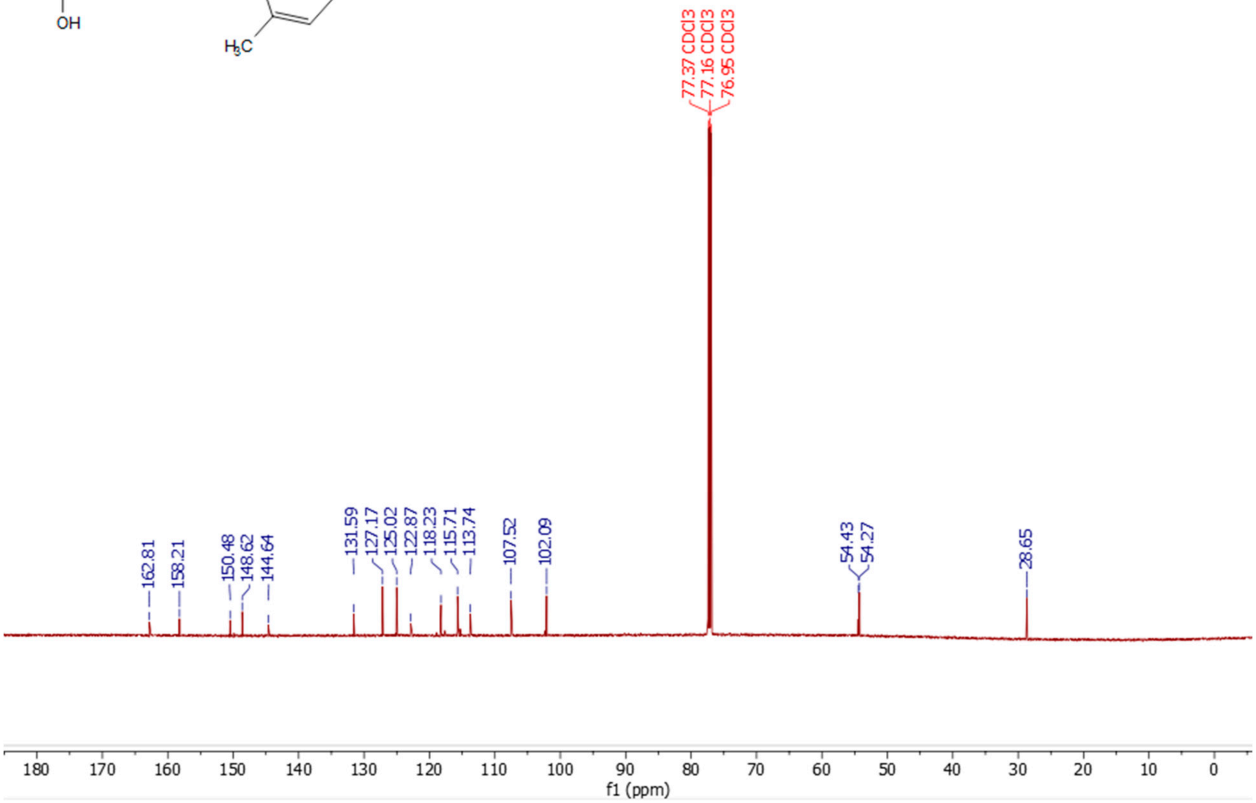

^{1}H NMR of **51**

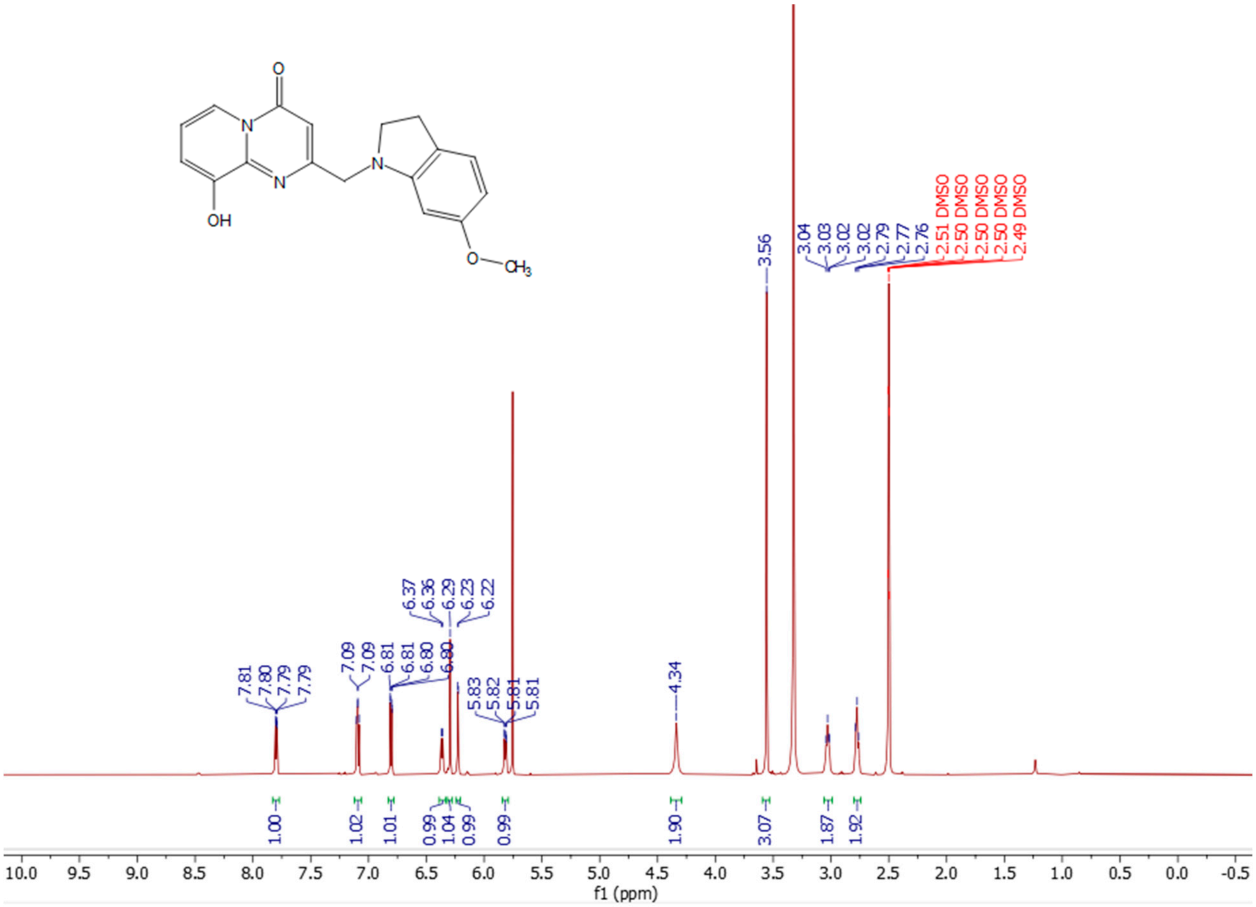

O
N
N
N
OH
O—CH3
3.56
3.04
3.03
3.02
3.02
2.79
2.77
2.76
2.51 DMSO
2.50 DMSO
2.50 DMSO
2.50 DMSO
2.49 DMSO
6.37
6.36
6.29
6.23
6.22
7.81
7.80
7.79
7.79
7.09
7.09
6.81
6.81
6.80
6.80
5.83
5.82
5.81
5.81
4.34
1.00
1.02
1.01
0.99
1.04
0.99
0.99
1.90
3.07
1.87
1.92
10.0 9.5 9.0 8.5 8.0 7.5 7.0 6.5 6.0 5.5 5.0 4.5 4.0 3.5 3.0 2.5 2.0 1.5 1.0 0.5 0.0 -0.5
f1 (ppm)

^{13}C NMR of **51**

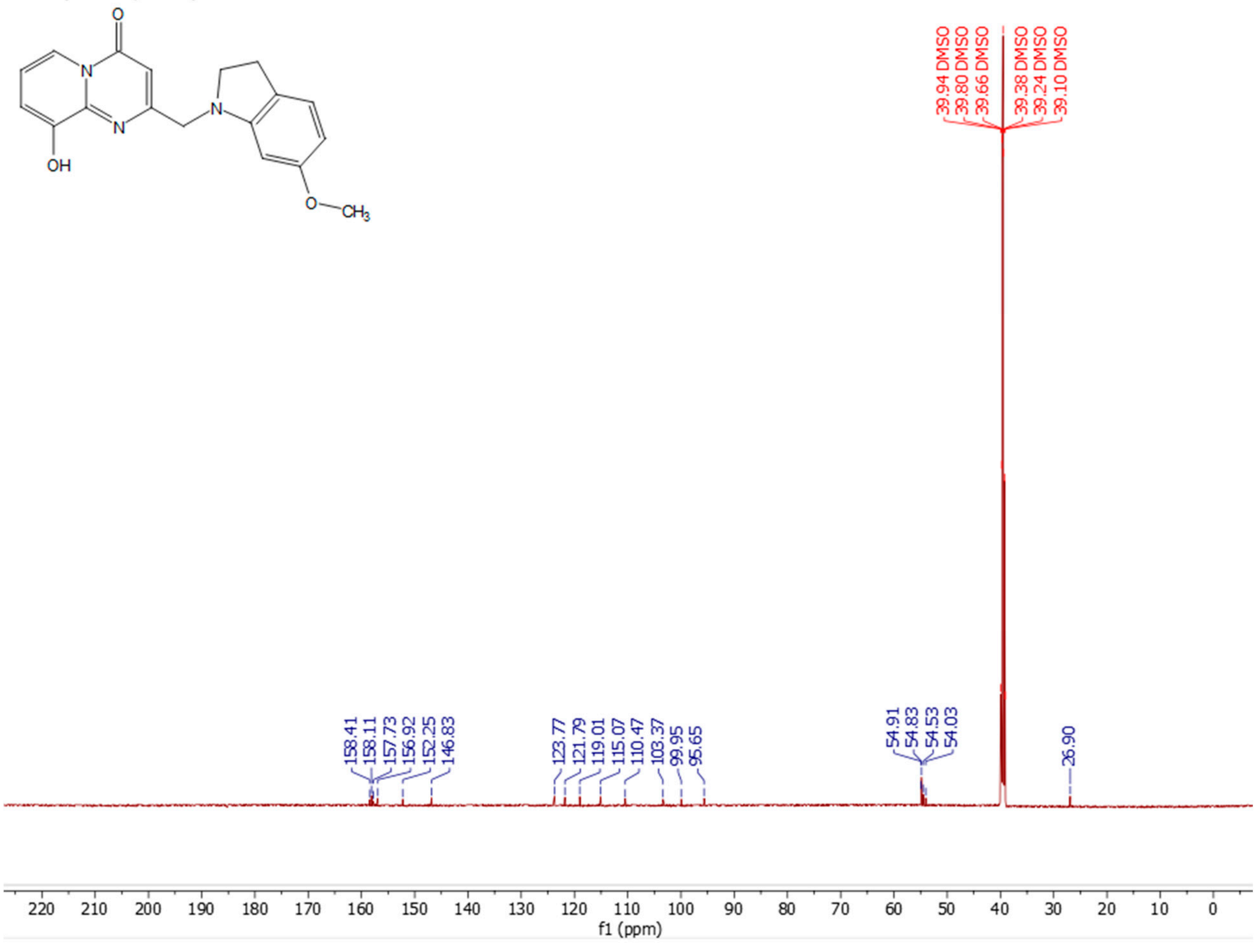

O
N
N
N
OH
O
CH3
39.94 DMSO
39.80 DMSO
39.66 DMSO
39.38 DMSO
39.24 DMSO
39.10 DMSO
158.41
158.11
157.73
156.92
152.25
146.83
123.77
121.79
119.01
115.07
110.47
103.37
99.95
95.65
54.91
54.83
54.53
54.03
26.90
220 210 200 190 180 170 160 150 140 130 120 110 100 90 80 70 60 50 40 30 20 10 0
f1 (ppm)

^{1}H NMR of **52**

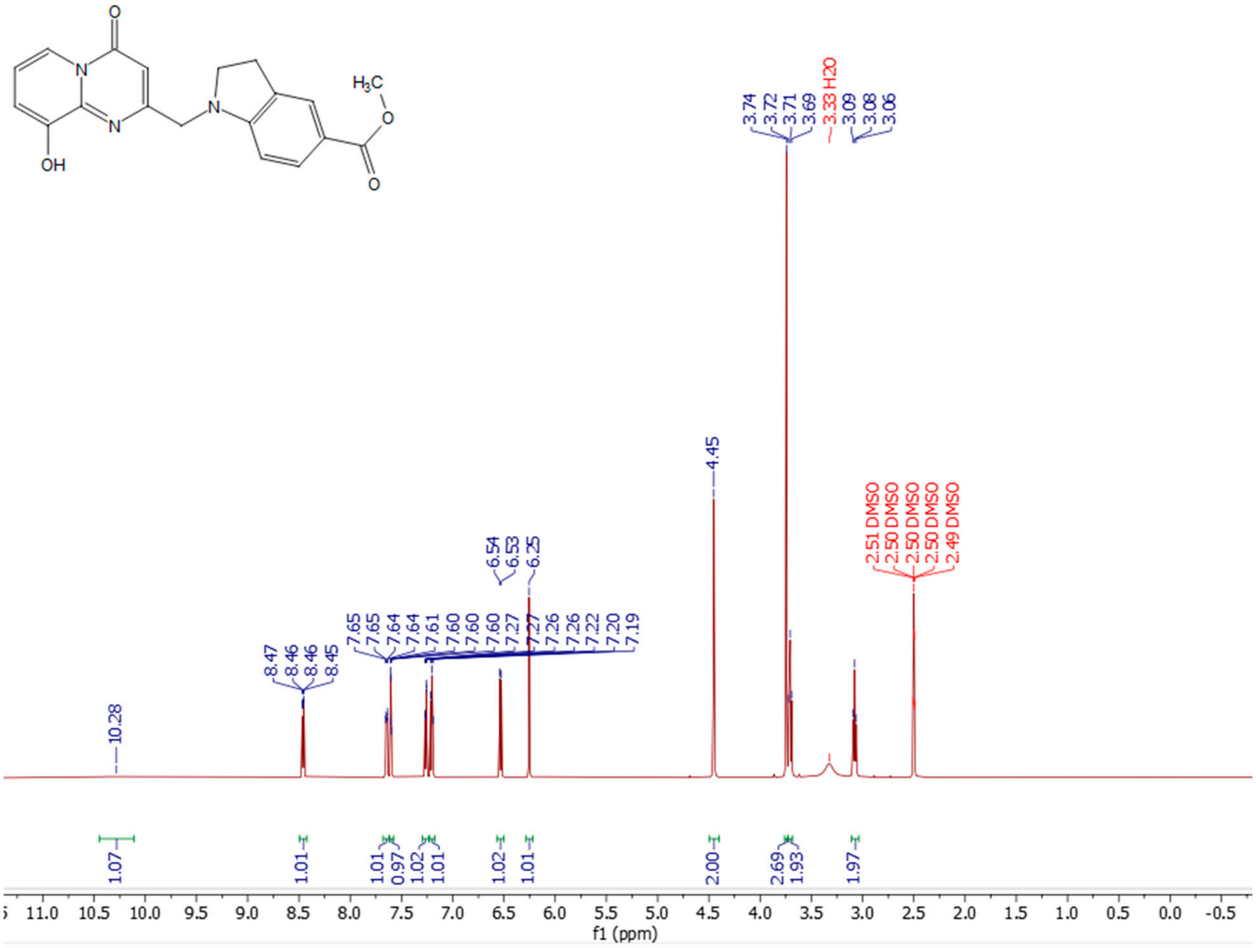

O
N
N
OH
N
H_3C
O
O
3.74
3.72
3.71
3.69
3.33 H2O
3.09
3.08
3.06
4.45
2.51 DMSO
2.50 DMSO
2.50 DMSO
2.50 DMSO
2.49 DMSO
6.54
6.53
6.25
7.65
7.65
7.64
7.64
7.61
7.60
7.60
7.60
7.60
7.27
7.27
7.26
7.26
7.22
7.20
7.19
8.47
8.46
8.46
8.45
10.28
1.07
1.01
1.01
0.97
1.02
1.01
1.02
1.01
2.00
2.69
1.93
1.97
11.0 10.5 10.0 9.5 9.0 8.5 8.0 7.5 7.0 6.5 6.0 5.5 5.0 4.5 4.0 3.5 3.0 2.5 2.0 1.5 1.0 0.5 0.0 -0.5
f1 (ppm)

^{13}C NMR of **52**

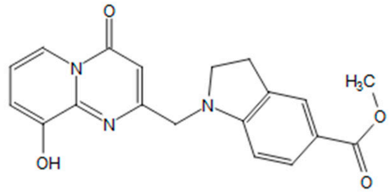
O
N
N
OH
N
H3C
O
O

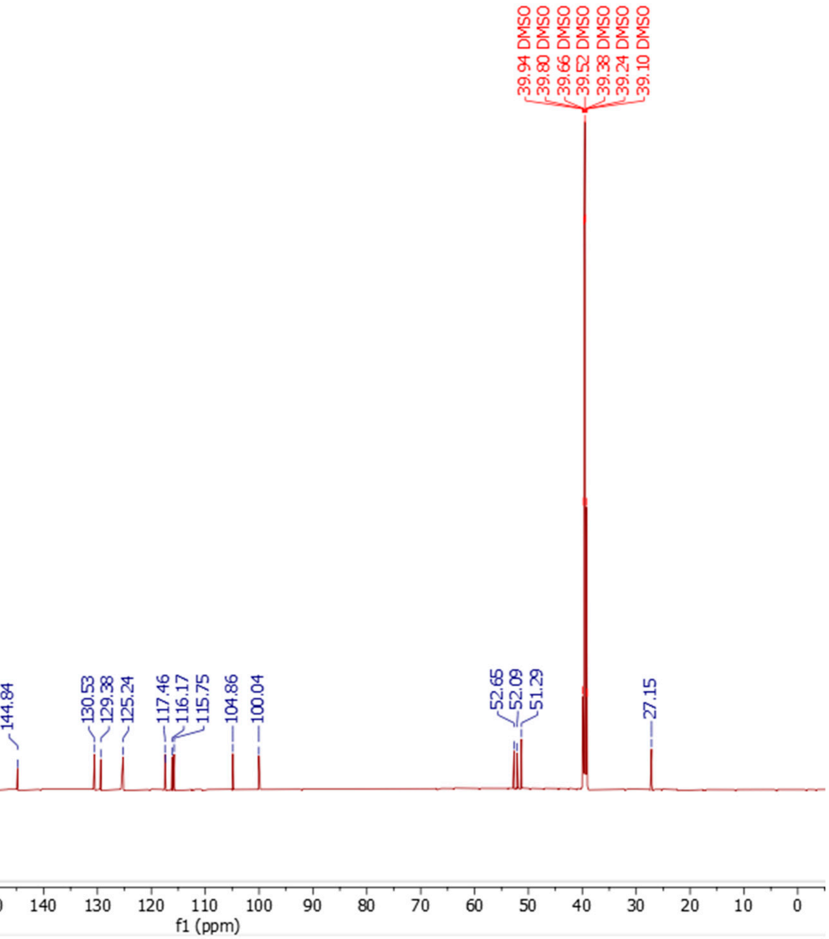
39.94 DMSO
39.80 DMSO
39.66 DMSO
39.52 DMSO
39.38 DMSO
39.24 DMSO
39.10 DMSO
166.31
162.09
157.27
155.49
149.84
144.84
130.53
129.38
125.24
117.46
116.17
115.75
104.86
100.04
52.65
52.09
51.29
27.15
220 210 200 190 180 170 160 150 140 130 120 110 100 90 80 70 60 50 40 30 20 10 0
f1 (ppm)

^{1}H NMR of **53**

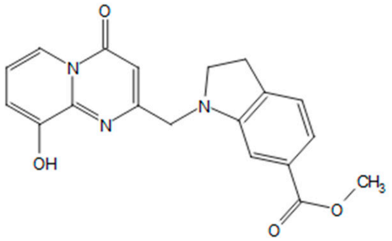

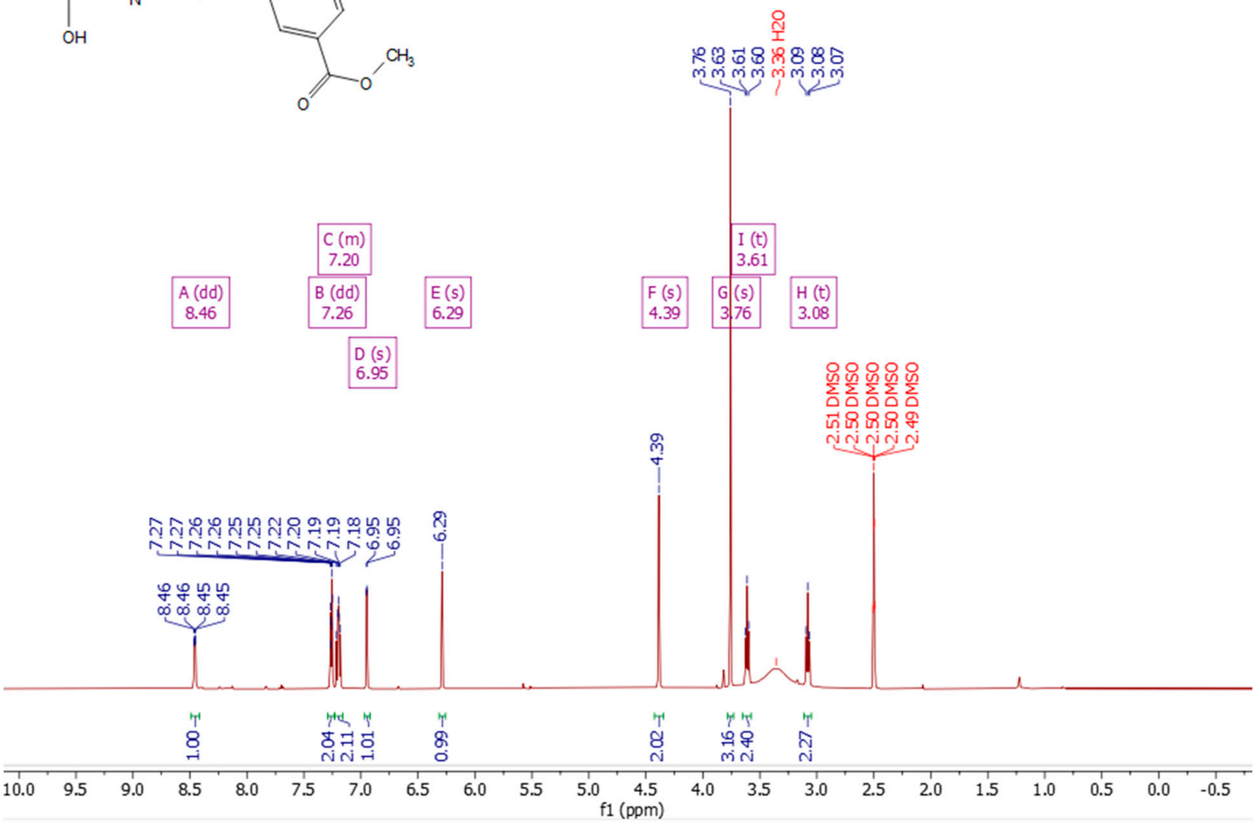
3.76
3.63
3.61
3.60
3.36 H2O
3.09
3.08
3.07
C (m)
7.20
I (t)
3.61
A (dd)
8.46
B (dd)
7.26
E (s)
6.29
F (s)
4.39
G (s)
3.76
H (t)
3.08
D (s)
6.95
2.51 DMSO
2.50 DMSO
2.50 DMSO
2.50 DMSO
2.49 DMSO
4.39
7.27
7.27
7.26
7.26
7.25
7.25
7.22
7.20
7.19
7.19
7.18
6.95
6.95
6.29
8.46
8.46
8.45
8.45
1.00
2.04
2.11
1.01
0.99
2.02
3.16
2.40
2.27
10.0 9.5 9.0 8.5 8.0 7.5 7.0 6.5 6.0 5.5 5.0 4.5 4.0 3.5 3.0 2.5 2.0 1.5 1.0 0.5 0.0 -0.5
f1 (ppm)

^{13}C NMR of **53**

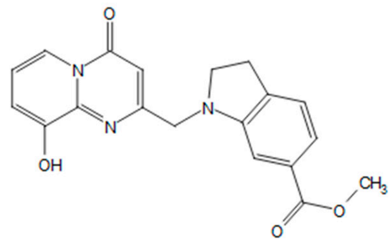

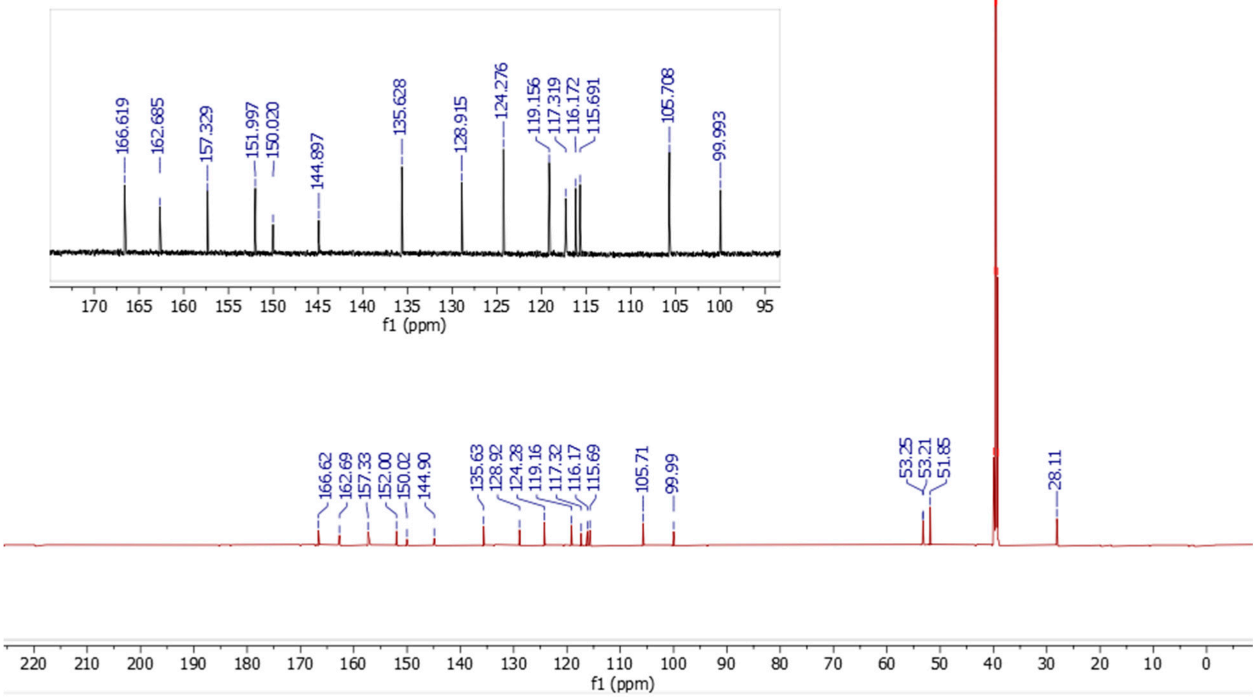

39.94 DMSO
39.80 DMSO
39.66 DMSO
39.52 DMSO
39.38 DMSO
39.24 DMSO
39.10 DMSO
166.619
162.685
157.329
151.997
150.020
144.897
135.628
128.915
124.276
119.156
117.319
116.172
115.691
105.708
99.993
f1 (ppm)
166.62
162.69
157.33
152.00
150.02
144.90
135.63
128.92
124.28
119.16
117.32
116.17
115.69
105.71
99.99
53.25
53.21
51.85
28.11
f1 (ppm)

^{1}H NMR of **54**

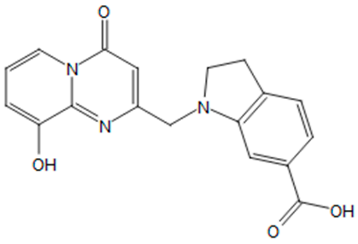

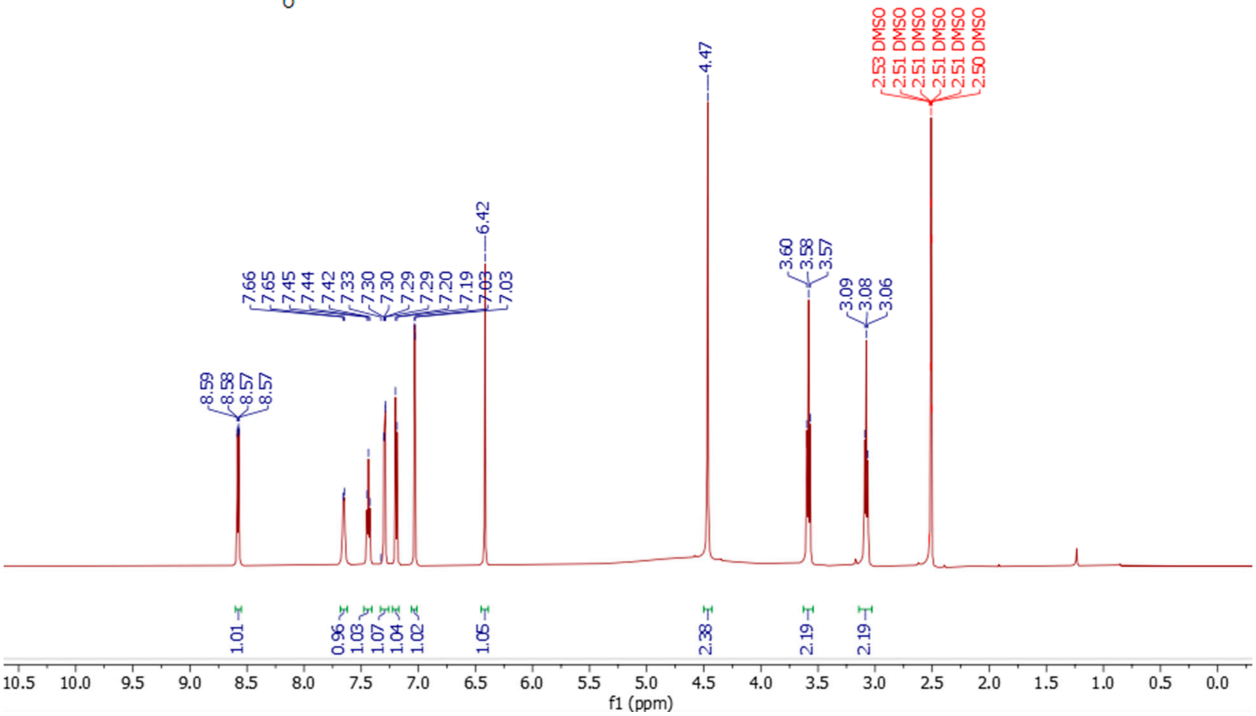

8.59
8.58
8.57
8.57
7.66
7.65
7.45
7.44
7.42
7.33
7.30
7.30
7.29
7.29
7.20
7.19
7.03
7.03
6.42
4.47
3.60
3.58
3.57
3.09
3.08
3.06
2.53 DMSO
2.51 DMSO
2.51 DMSO
2.51 DMSO
2.51 DMSO
2.50 DMSO
1.01
0.96
1.03
1.07
1.04
1.02
1.05
2.38
2.19
2.19
10.5 10.0 9.5 9.0 8.5 8.0 7.5 7.0 6.5 6.0 5.5 5.0 4.5 4.0 3.5 3.0 2.5 2.0 1.5 1.0 0.5 0.0
f1 (ppm)

^{13}C NMR of **54**

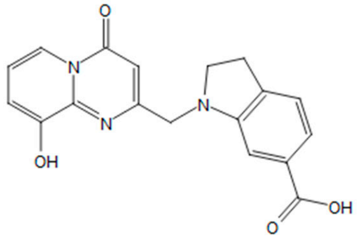

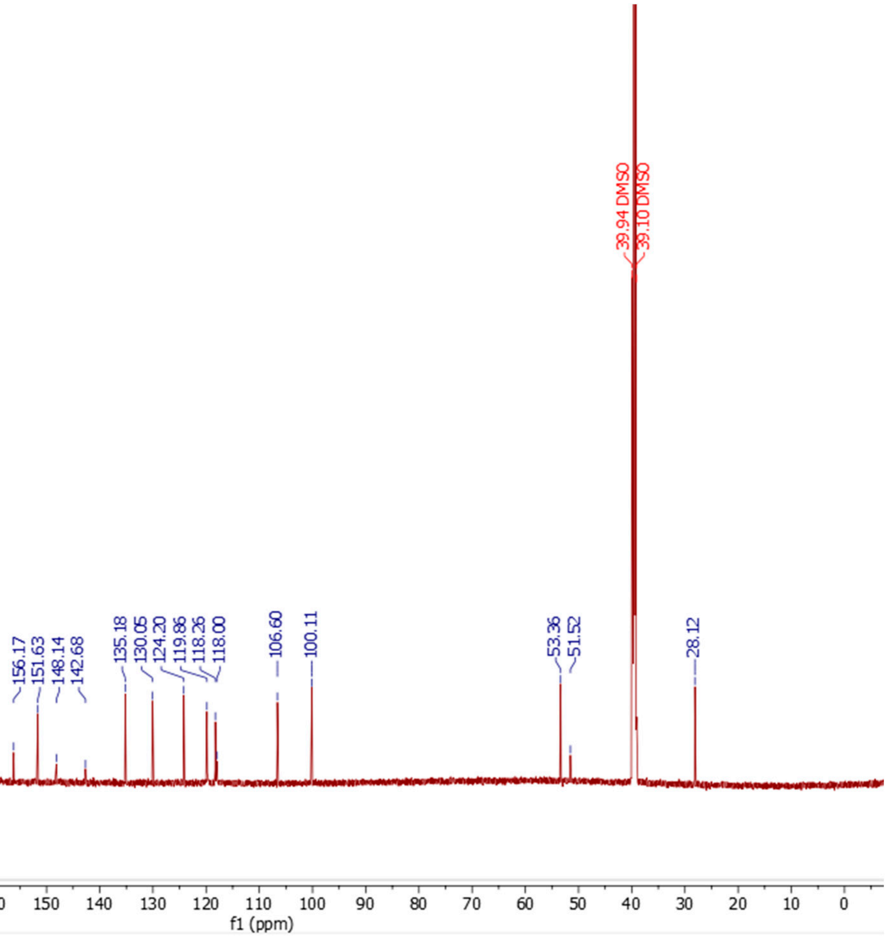
39.94 DMSO
39.10 DMSO
167.66
156.17
151.63
148.14
142.68
135.18
130.05
124.20
119.86
118.26
118.00
106.60
100.11
53.36
51.52
28.12
220 210 200 190 180 170 160 150 140 130 120 110 100 90 80 70 60 50 40 30 20 10 0
f1 (ppm)

^{1}H NMR of **55**

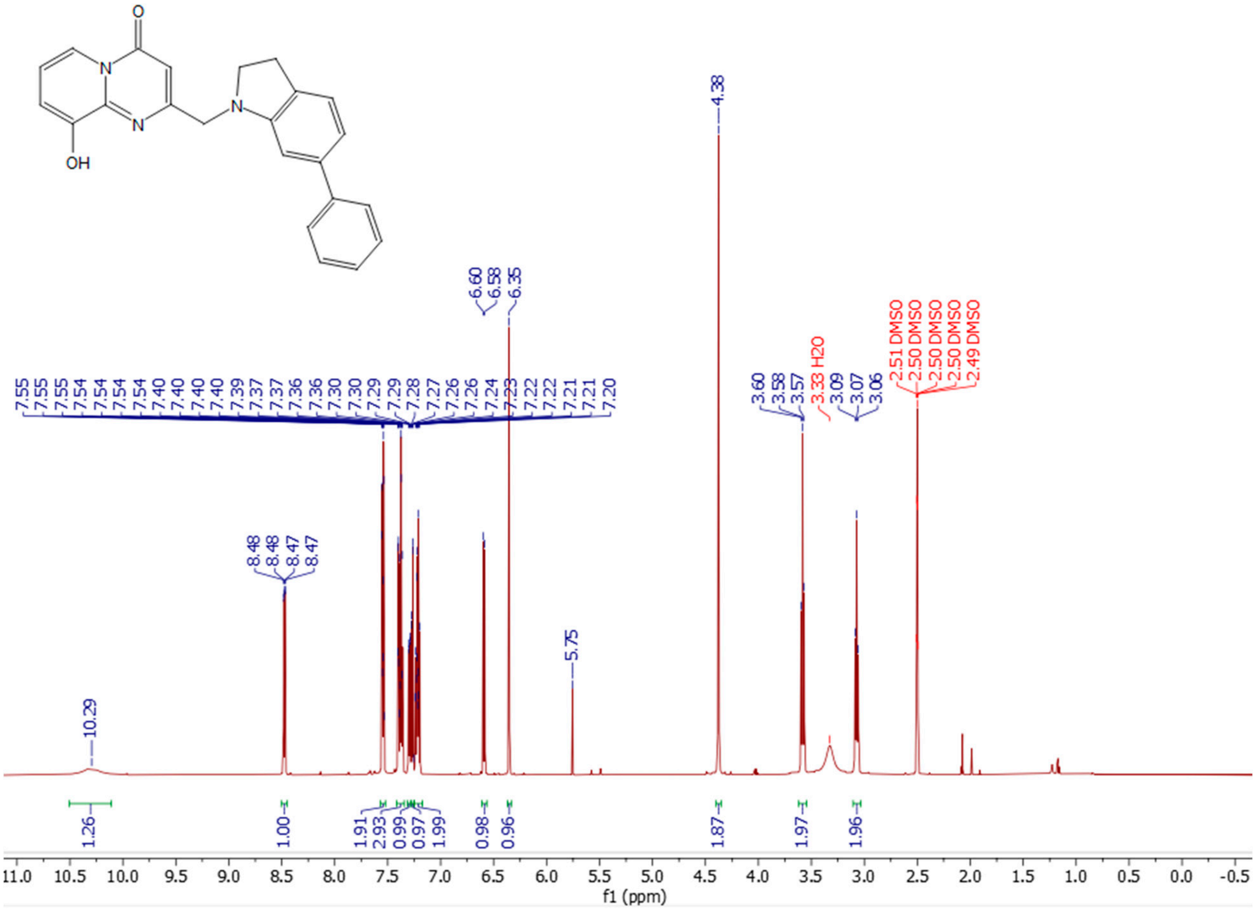
O
N
N
N
OH
10.29
8.48
8.48
8.47
8.47
7.55
7.55
7.55
7.54
7.54
7.54
7.40
7.40
7.40
7.39
7.37
7.37
7.36
7.36
7.30
7.30
7.29
7.29
7.28
7.27
7.26
7.26
7.24
7.23
7.22
7.22
7.21
7.21
7.20
6.60
6.58
6.35
5.75
4.38
3.60
3.58
3.57
3.33 H2O
3.09
3.07
3.06
2.51 DMSO
2.50 DMSO
2.50 DMSO
2.50 DMSO
2.49 DMSO
1.26
1.00
1.91
2.93
0.99
0.97
1.99
0.98
0.96
1.87
1.97
1.96
11.0 10.5 10.0 9.5 9.0 8.5 8.0 7.5 7.0 6.5 6.0 5.5 5.0 4.5 4.0 3.5 3.0 2.5 2.0 1.5 1.0 0.5 0.0 -0.5
f1 (ppm)

^{13}C NMR of **55**

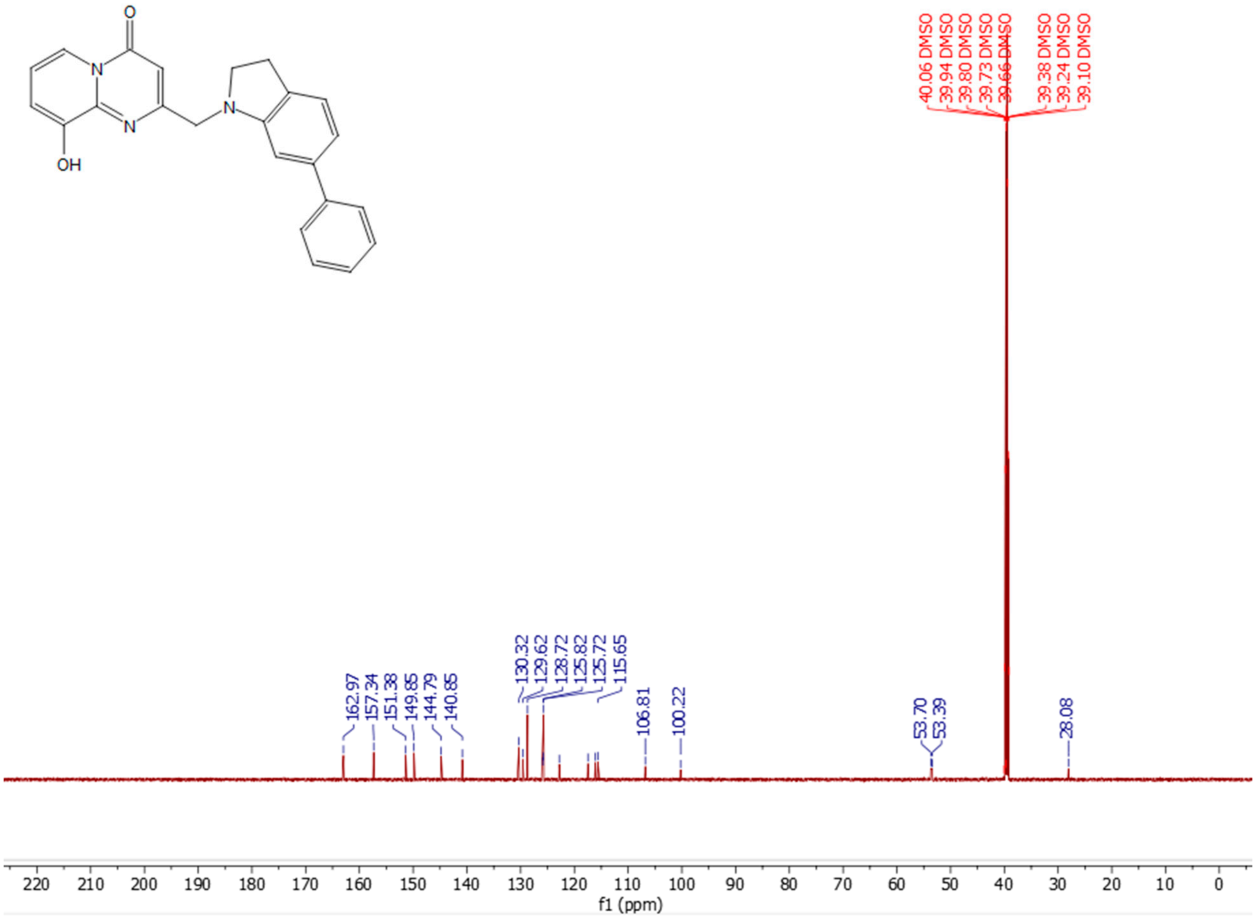
O
N
N
N
OH
40.06 DMSO
39.94 DMSO
39.80 DMSO
39.73 DMSO
39.66 DMSO
39.38 DMSO
39.24 DMSO
39.10 DMSO
162.97
157.34
151.38
149.85
144.79
140.85
130.32
129.62
128.72
125.82
125.72
115.65
106.81
100.22
53.70
53.39
28.08
220 210 200 190 180 170 160 150 140 130 120 110 100 90 80 70 60 50 40 30 20 10 0
f1 (ppm)

^{1}H NMR of **56**

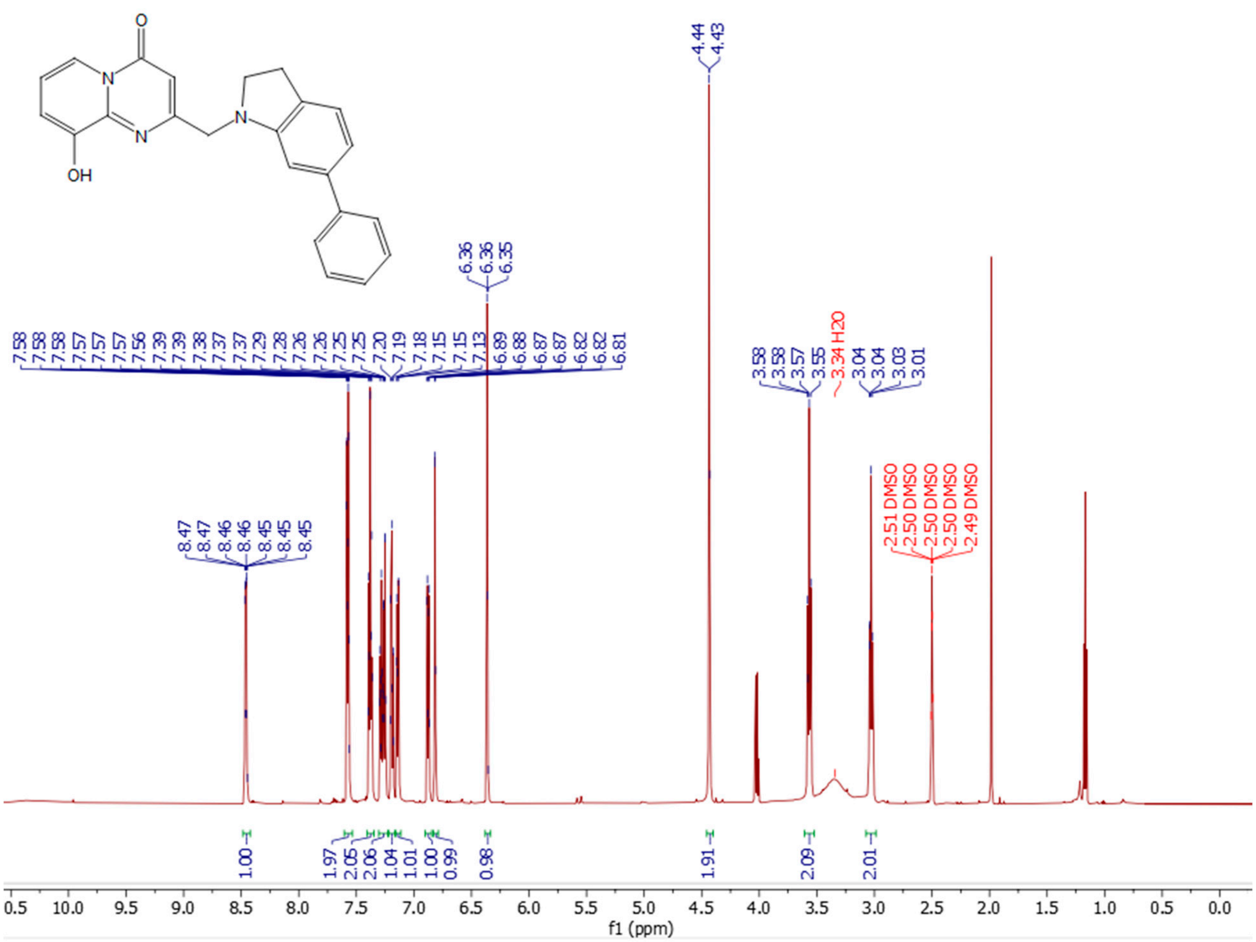

^{13}C NMR of **56**

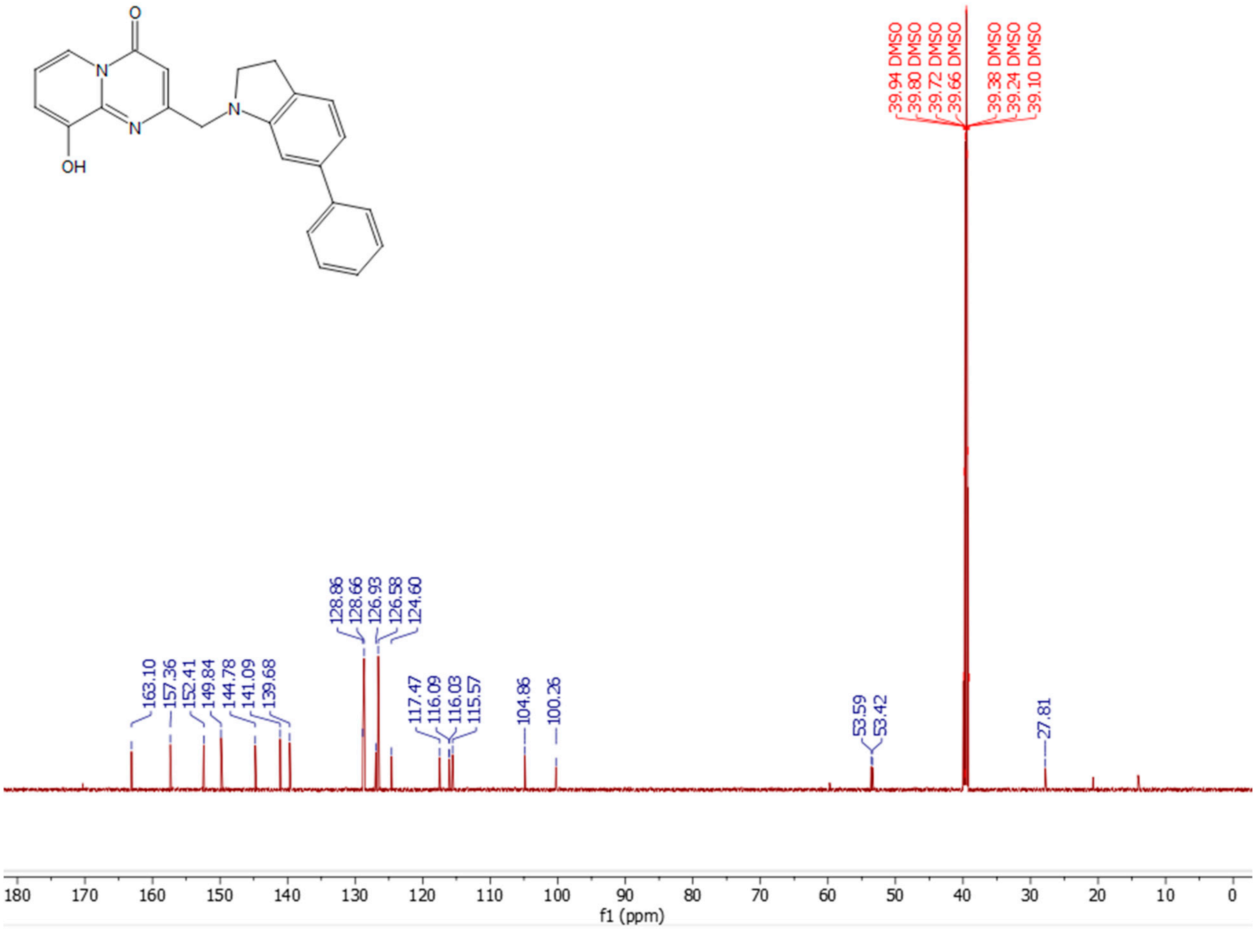
39.94 DMSO
39.80 DMSO
39.72 DMSO
39.66 DMSO
39.38 DMSO
39.24 DMSO
39.10 DMSO
163.10
157.36
152.41
149.84
144.78
141.09
139.68
128.86
128.66
126.93
126.58
124.60
117.47
116.09
116.03
115.57
104.86
100.26
53.59
53.42
27.81
180 170 160 150 140 130 120 110 100 90 80 70 60 50 40 30 20 10 0
f1 (ppm)

^{1}H NMR of **57**

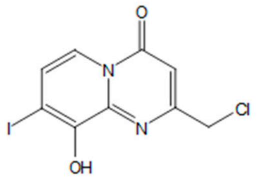
O
N
N
Cl
I
OH

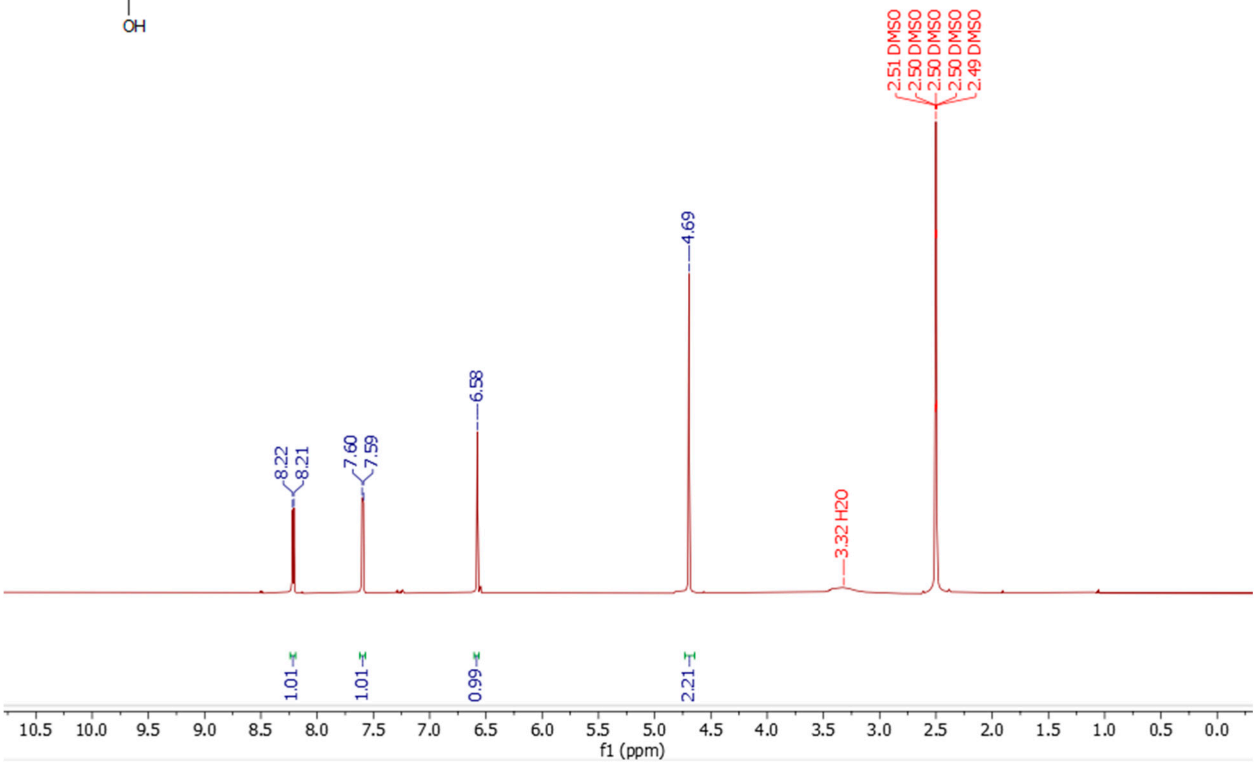
2.51 DMSO
2.50 DMSO
2.50 DMSO
2.50 DMSO
2.49 DMSO
4.69
6.58
7.60
7.59
8.22
8.21
3.32 H2O
1.01
1.01
0.99
2.21
10.5 10.0 9.5 9.0 8.5 8.0 7.5 7.0 6.5 6.0 5.5 5.0 4.5 4.0 3.5 3.0 2.5 2.0 1.5 1.0 0.5 0.0
f1 (ppm)

^{13}C NMR of **57**

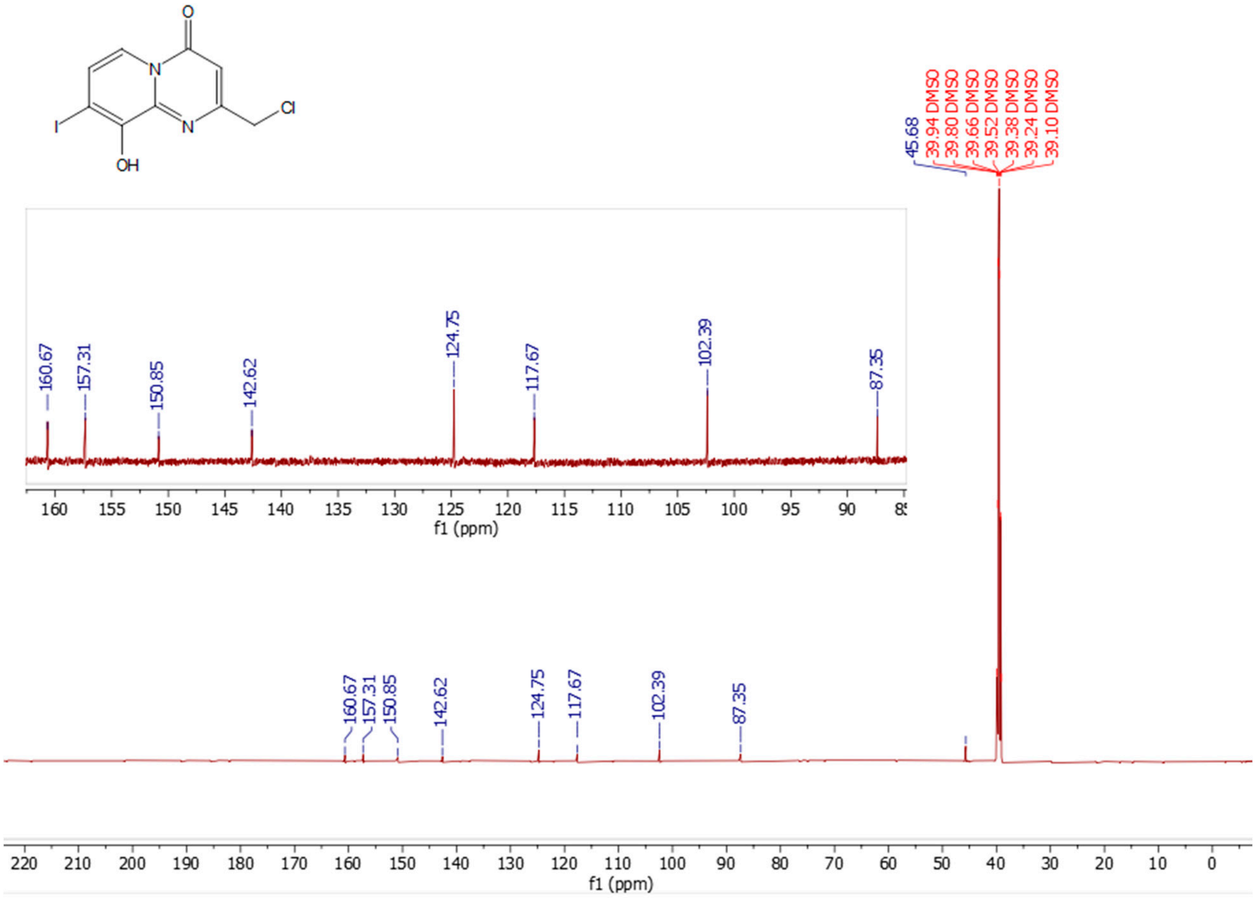

O
N
N
Cl
I
OH
45.68
39.94 DMSO
39.80 DMSO
39.66 DMSO
39.52 DMSO
39.38 DMSO
39.24 DMSO
39.10 DMSO
160.67
157.31
150.85
142.62
124.75
117.67
102.39
87.35
f1 (ppm)

^{1}H NMR of **58**

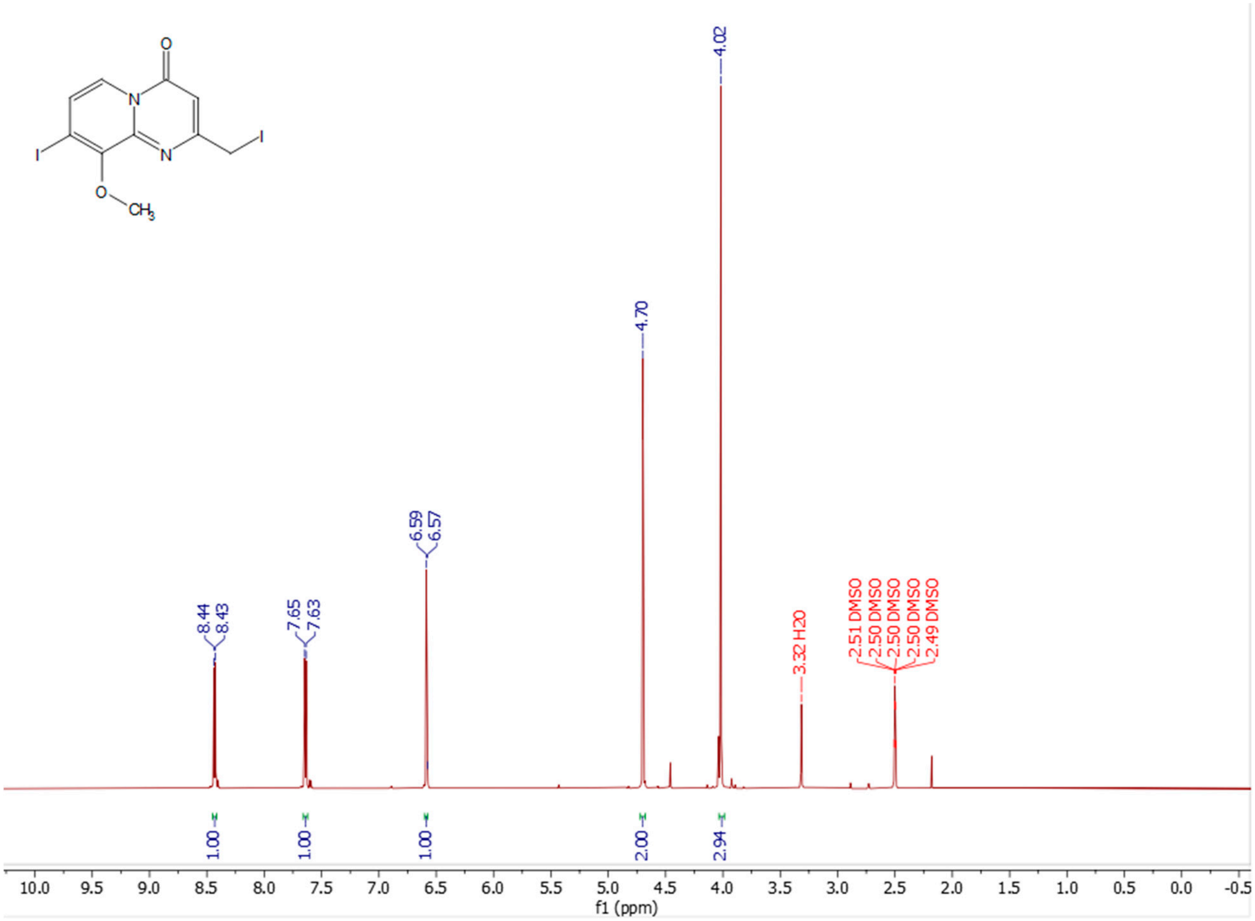

O
N
N
I
I
O
CH3
4.02
4.70
6.59
6.57
8.44
8.43
7.65
7.63
3.32 H2O
2.51 DMSO
2.50 DMSO
2.50 DMSO
2.50 DMSO
2.49 DMSO
1.00
1.00
1.00
2.00
2.94
10.0
9.5
9.0
8.5
8.0
7.5
7.0
6.5
6.0
5.5
5.0
4.5
4.0
3.5
3.0
2.5
2.0
1.5
1.0
0.5
0.0
-0.5
f1 (ppm)

^{13}C NMR of **58**

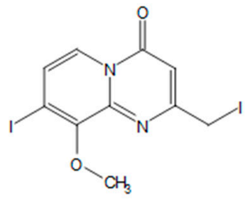
O
N
N
I
I
O
CH3

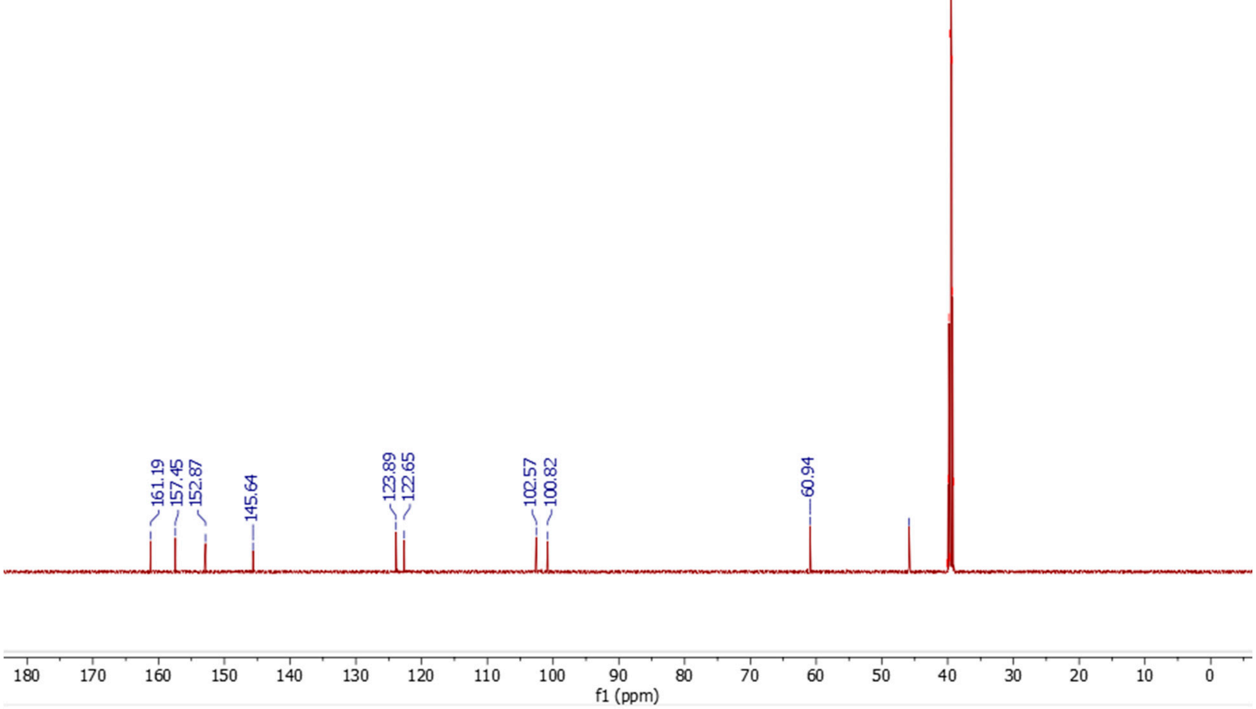
45.87
40.06 DMSO
39.94 DMSO
39.80 DMSO
39.73 DMSO
39.66 DMSO
39.52 DMSO
39.38 DMSO
39.24 DMSO
39.10 DMSO
161.19
157.45
152.87
145.64
123.89
122.65
102.57
100.82
60.94
180 170 160 150 140 130 120 110 100 90 80 70 60 50 40 30 20 10 0
f1 (ppm)

^{1}H NMR of **59**

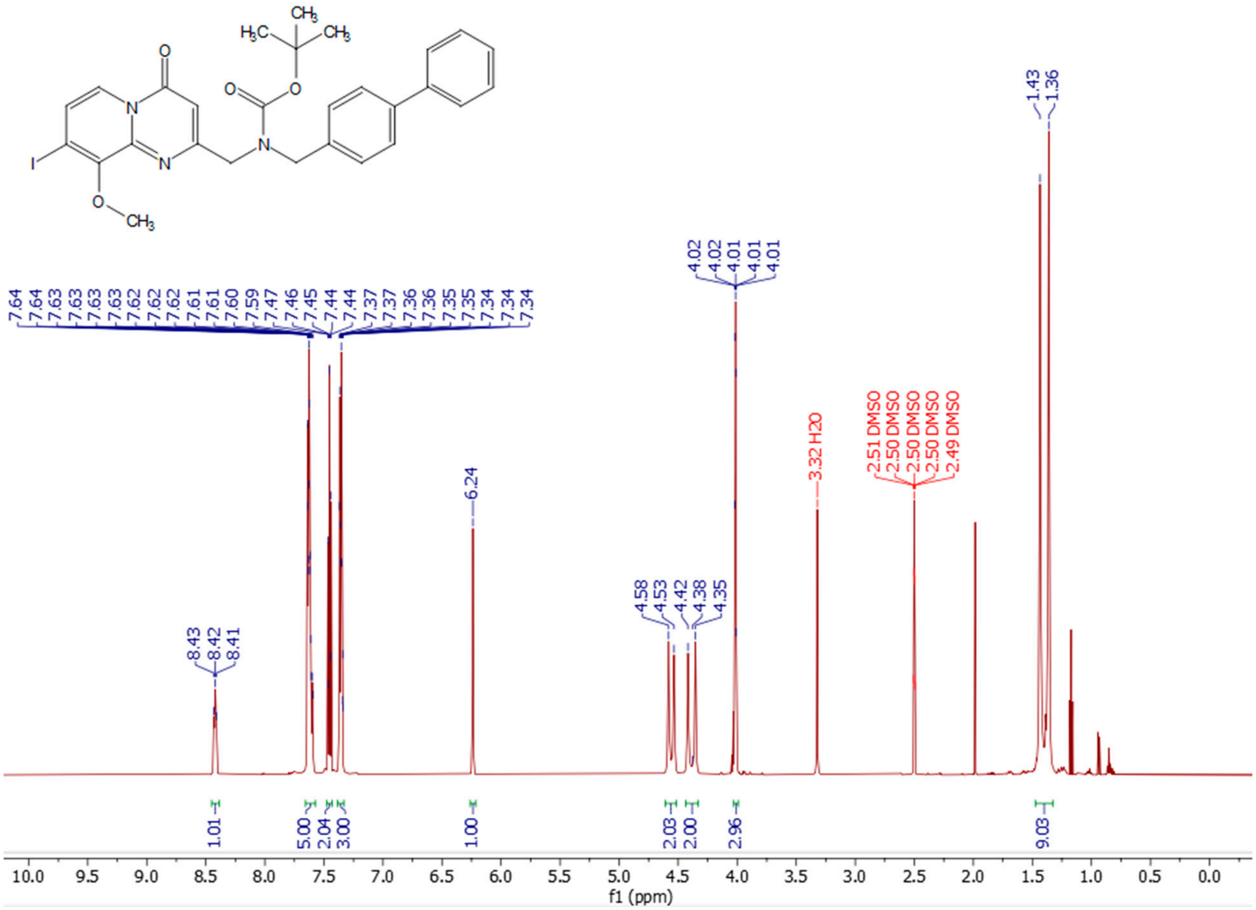

H_3C
CH_3
CH_3
O
O
O
N
N
N
I
O
CH_3
7.64
7.64
7.63
7.63
7.63
7.62
7.62
7.62
7.61
7.61
7.60
7.59
7.47
7.46
7.45
7.44
7.44
7.37
7.37
7.36
7.36
7.35
7.35
7.34
7.34
7.34
4.02
4.02
4.01
4.01
4.01
1.43
1.36
6.24
3.32 H2O
2.51 DMSO
2.50 DMSO
2.50 DMSO
2.50 DMSO
2.49 DMSO
8.43
8.42
8.41
4.58
4.53
4.42
4.38
4.35
1.01
5.00
2.04
3.00
1.00
2.03
2.00
2.96
9.03
10.0
9.5
9.0
8.5
8.0
7.5
7.0
6.5
6.0
5.5
5.0
4.5
4.0
3.5
3.0
2.5
2.0
1.5
1.0
0.5
0.0
f1 (ppm)

¹³C NMR of **59**

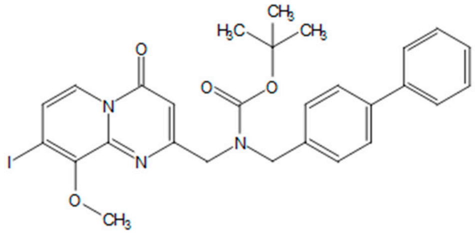

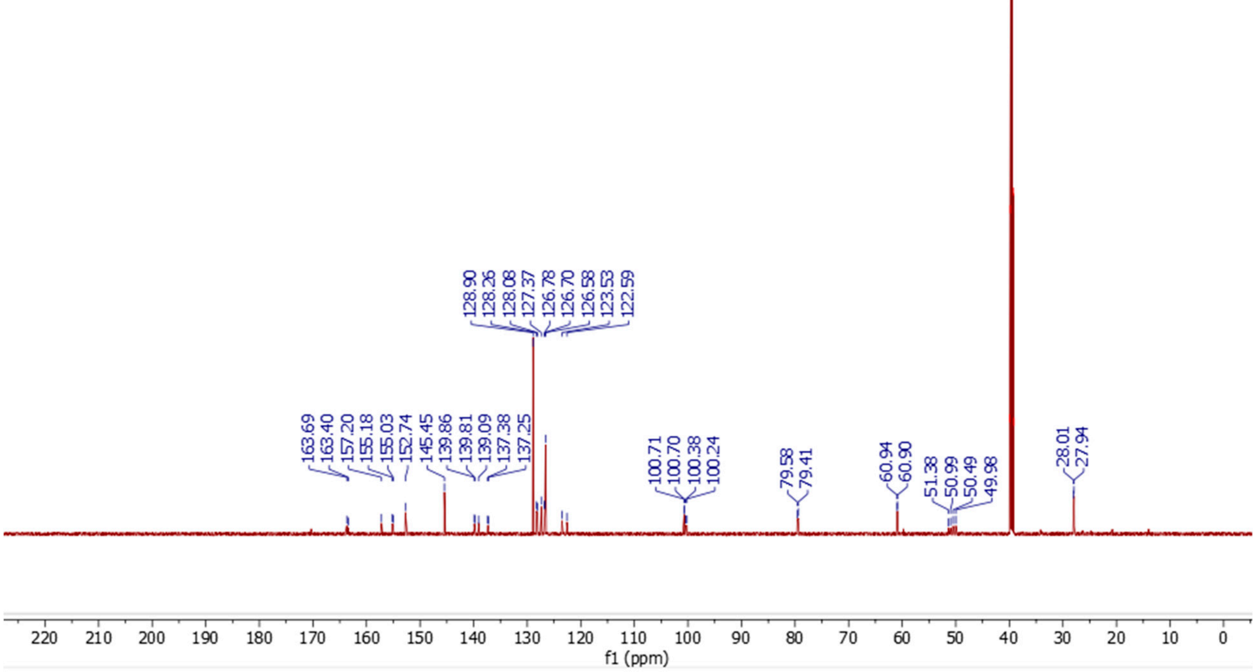
39.96 DMSO
39.82 DMSO
39.68 DMSO
39.41 DMSO
39.27 DMSO
39.13 DMSO
128.90
128.26
128.08
127.37
126.78
126.70
126.58
123.53
122.59
163.69
163.40
157.20
155.18
155.03
152.74
145.45
139.86
139.81
139.09
137.38
137.25
100.71
100.70
100.38
100.24
79.58
79.41
60.94
60.90
51.38
50.99
50.49
49.98
28.01
27.94
220 210 200 190 180 170 160 150 140 130 120 110 100 90 80 70 60 50 40 30 20 10 0
f1 (ppm)

^{1}H NMR of **60**

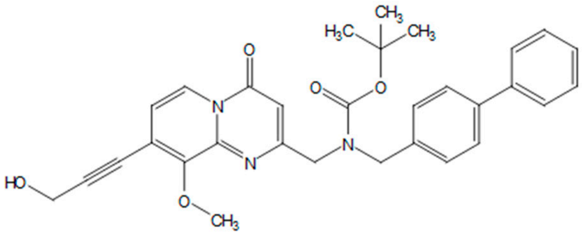
HO
O
N
N
O
O
N
O
CH3
H3C
CH3
CH3

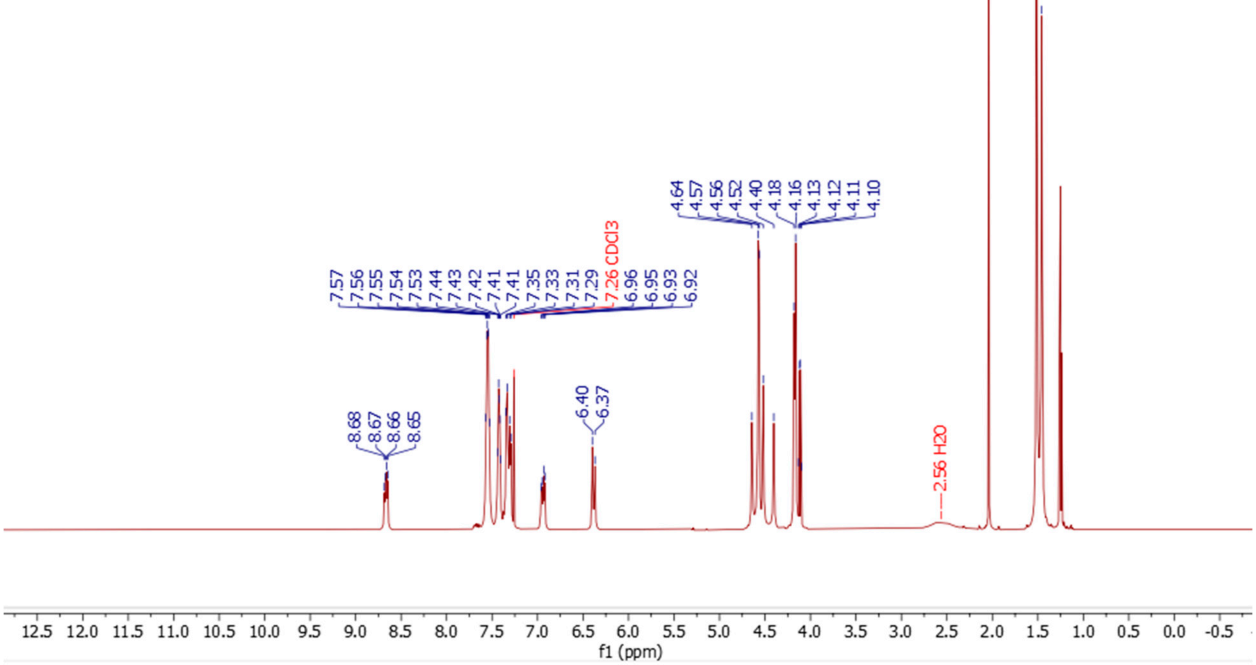
1.51
1.46
4.64
4.57
4.56
4.52
4.40
4.18
4.16
4.13
4.12
4.11
4.10
7.57
7.56
7.55
7.54
7.53
7.44
7.43
7.42
7.41
7.41
7.35
7.33
7.31
7.29
7.26 CDCl3
6.96
6.95
6.93
6.92
8.68
8.67
8.66
8.65
6.40
6.37
2.56 H2O
12.5 12.0 11.5 11.0 10.5 10.0 9.5 9.0 8.5 8.0 7.5 7.0 6.5 6.0 5.5 5.0 4.5 4.0 3.5 3.0 2.5 2.0 1.5 1.0 0.5 0.0 -0.5
f1 (ppm)

^{13}C NMR of **60**

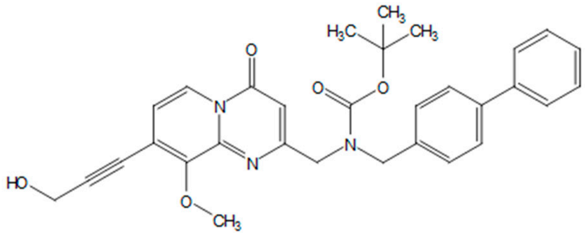

HO
N
N
O
O
O
O
CH3
H3C
CH3
CH3

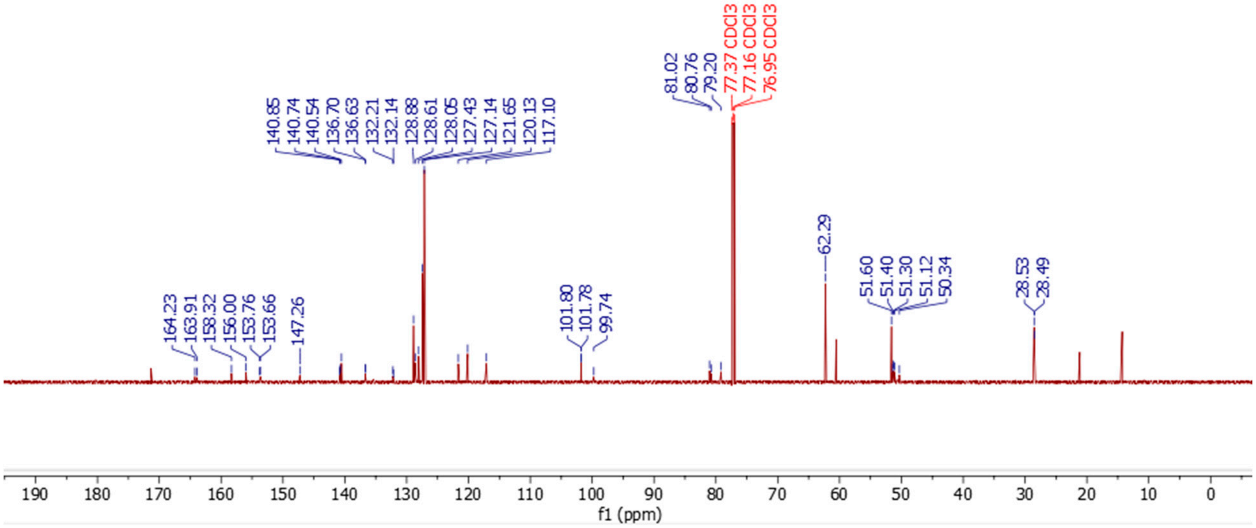

164.23
163.91
158.32
156.00
153.76
153.66
147.26
140.85
140.74
140.54
136.70
136.63
132.21
132.14
128.88
128.61
128.05
127.43
127.14
121.65
120.13
117.10
101.80
101.78
99.74
81.02
80.76
79.20
77.37 CDCl3
77.16 CDCl3
76.95 CDCl3
62.29
51.60
51.40
51.30
51.12
50.34
28.53
28.49
190
180
170
160
150
140
130
120
110
100
90
80
70
60
50
40
30
20
10
0
f1 (ppm)

^{1}H NMR of **61**

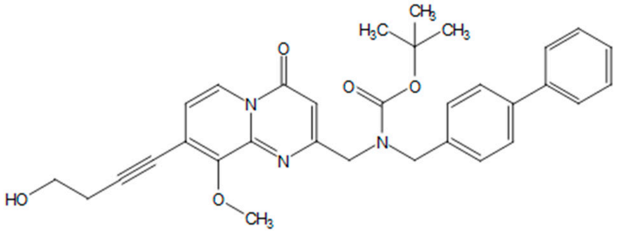

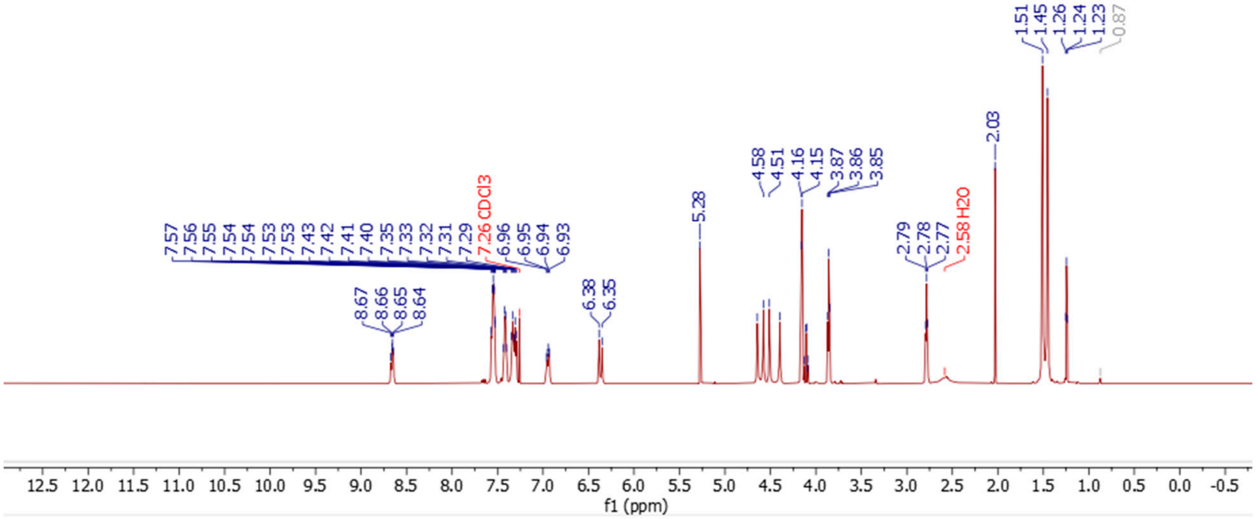

7.57
7.56
7.55
7.54
7.54
7.53
7.53
7.43
7.42
7.41
7.40
7.35
7.33
7.32
7.31
7.29
7.26 CDCl3
6.96
6.95
6.94
6.93
8.67
8.66
8.65
8.64
6.38
6.35
5.28
4.58
4.51
4.16
4.15
3.87
3.86
3.85
2.79
2.78
2.77
2.58 H2O
2.03
1.51
1.45
1.26
1.24
1.23
0.87
12.5 12.0 11.5 11.0 10.5 10.0 9.5 9.0 8.5 8.0 7.5 7.0 6.5 6.0 5.5 5.0 4.5 4.0 3.5 3.0 2.5 2.0 1.5 1.0 0.5 0.0 -0.5
f1 (ppm)

^{13}C NMR of **61**

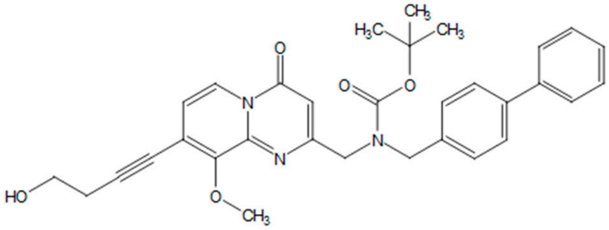

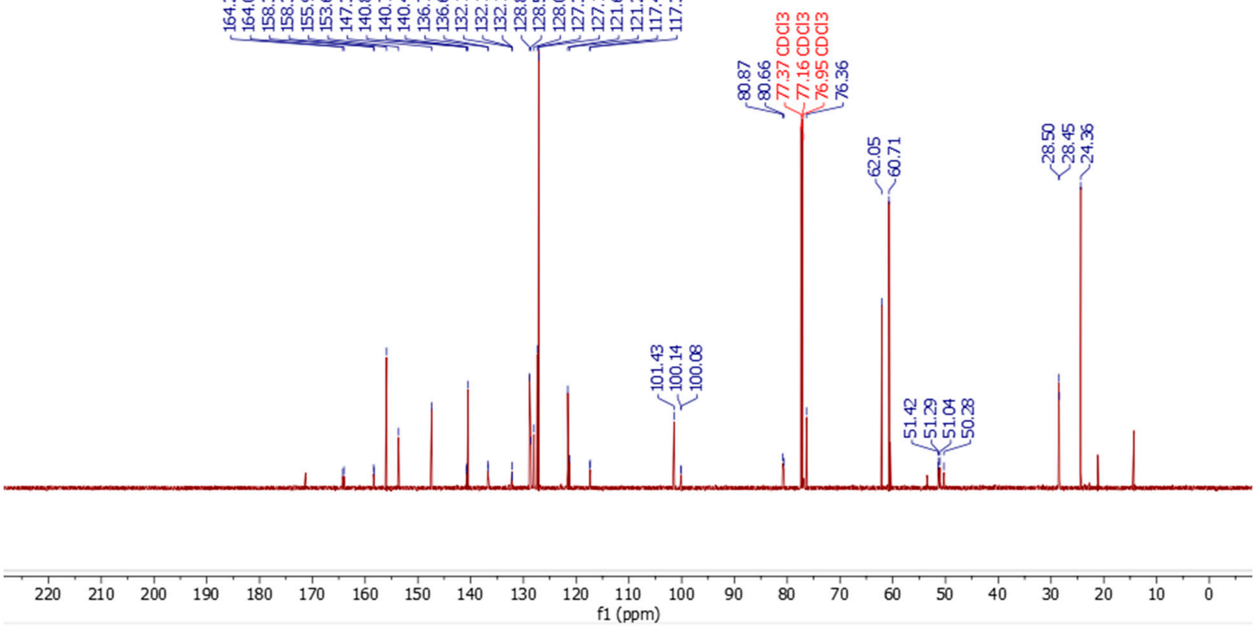
164.25
164.00
158.39
158.31
155.92
153.66
147.38
140.84
140.73
140.47
136.75
136.65
132.16
132.14
132.12
128.85
128.56
128.01
127.38
127.10
121.60
121.22
117.41
117.32
80.87
80.66
77.37 CDCl3
77.16 CDCl3
76.95 CDCl3
76.36
62.05
60.71
28.50
28.45
24.36
101.43
100.14
100.08
51.42
51.29
51.04
50.28
220 210 200 190 180 170 160 150 140 130 120 110 100 90 80 70 60 50 40 30 20 10 0
f1 (ppm)

^{1}H NMR of **62**

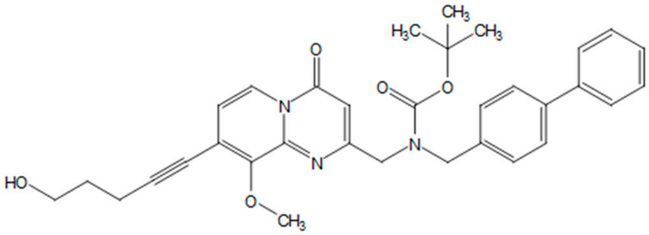

HO
O
CH3
N
N
O
O
O
N
H3C
CH3
CH3

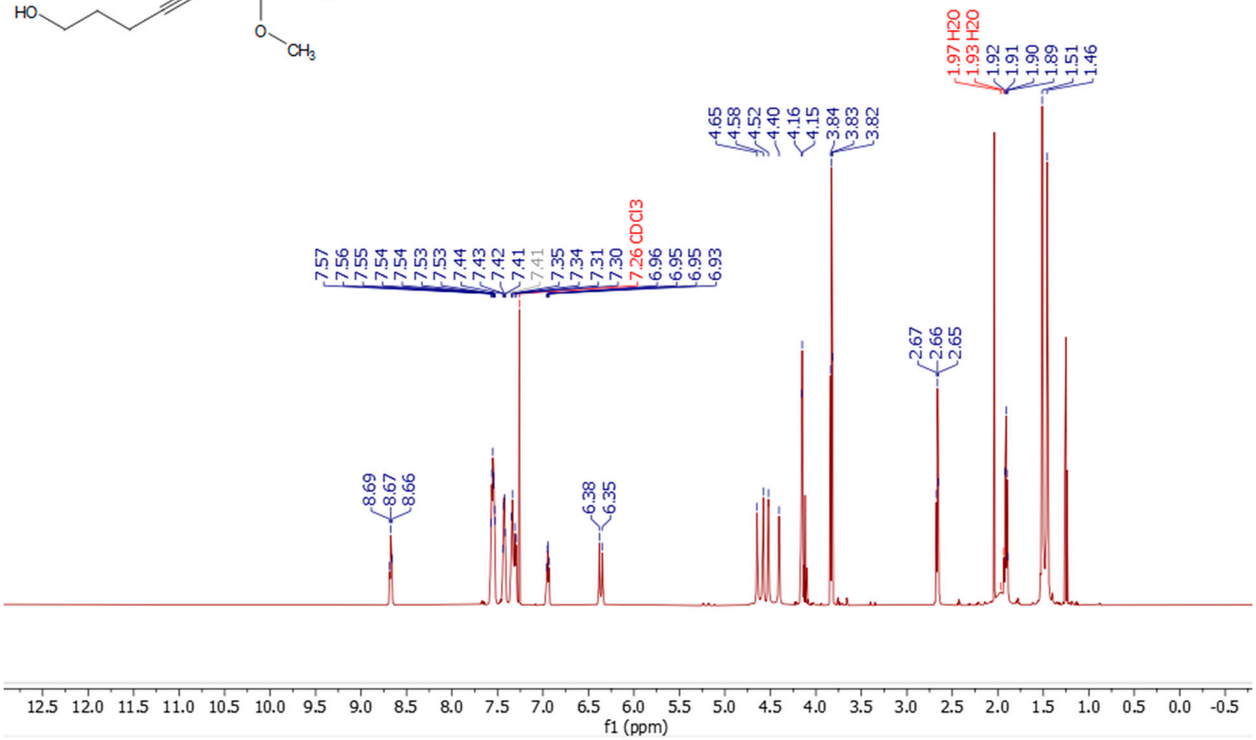

7.57
7.56
7.55
7.54
7.54
7.53
7.53
7.44
7.43
7.42
7.41
7.41
7.35
7.34
7.31
7.30
7.26 CDCl3
6.96
6.95
6.95
6.93
4.65
4.58
4.52
4.40
4.16
4.15
3.84
3.83
3.82
1.97 H2O
1.93 H2O
1.92
1.91
1.90
1.89
1.51
1.46
8.69
8.67
8.66
6.38
6.35
2.67
2.66
2.65
12.5 12.0 11.5 11.0 10.5 10.0 9.5 9.0 8.5 8.0 7.5 7.0 6.5 6.0 5.5 5.0 4.5 4.0 3.5 3.0 2.5 2.0 1.5 1.0 0.5 0.0 -0.5
f1 (ppm)

^{13}C NMR of **62**

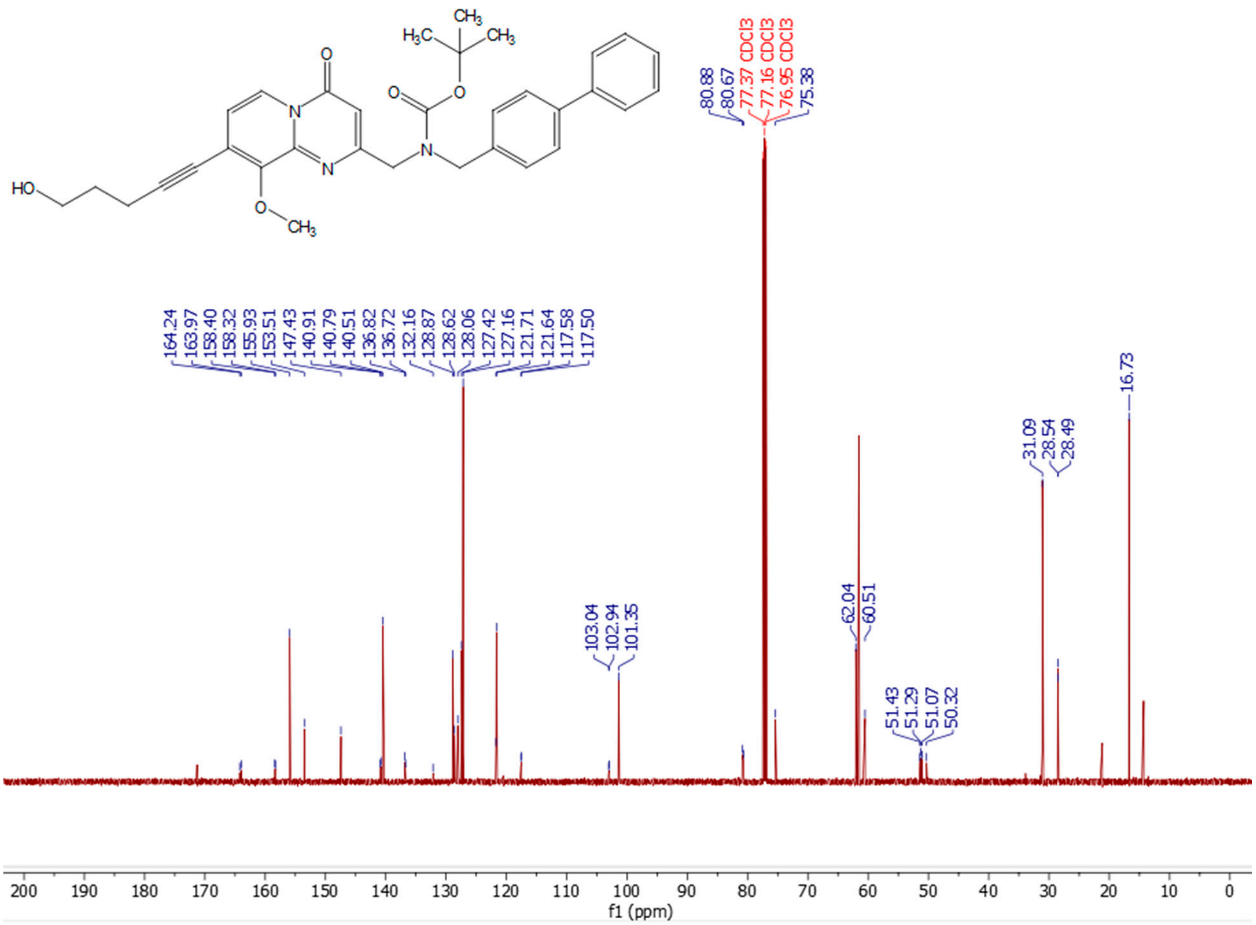
HO
O
CH3
N
N
O
O
N
O
O
H3C
CH3
CH3
164.24
163.97
158.40
158.32
155.93
153.51
147.43
140.91
140.79
140.51
136.82
136.72
132.16
128.87
128.62
128.06
127.42
127.16
121.71
121.64
117.58
117.50
103.04
102.94
101.35
80.88
80.67
77.37 CDCl3
77.16 CDCl3
76.95 CDCl3
75.38
62.04
60.51
51.43
51.29
51.07
50.32
31.09
28.54
28.49
16.73
200
190
180
170
160
150
140
130
120
110
100
90
80
70
60
50
40
30
20
10
0
f1 (ppm)

^{1}H NMR of **63**

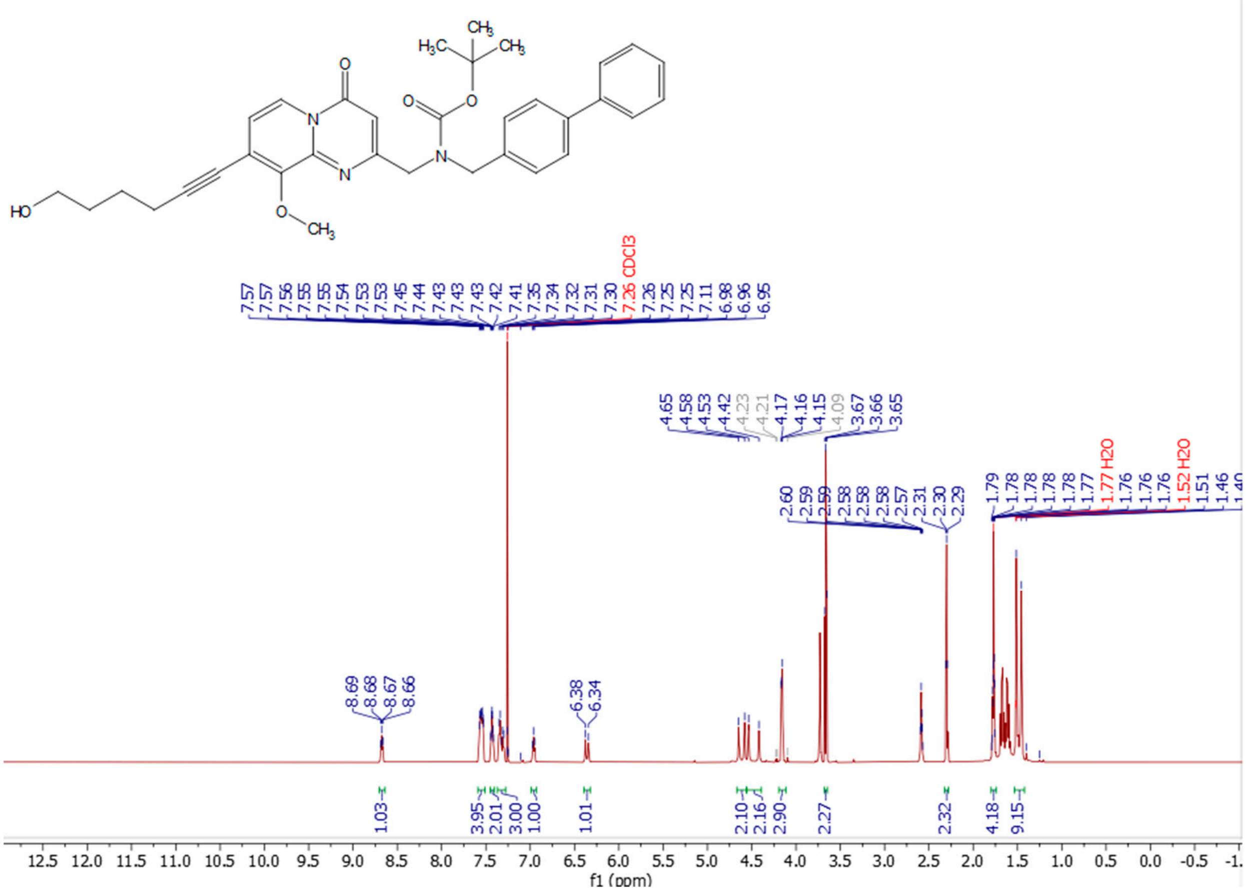

^{13}C NMR of **63**

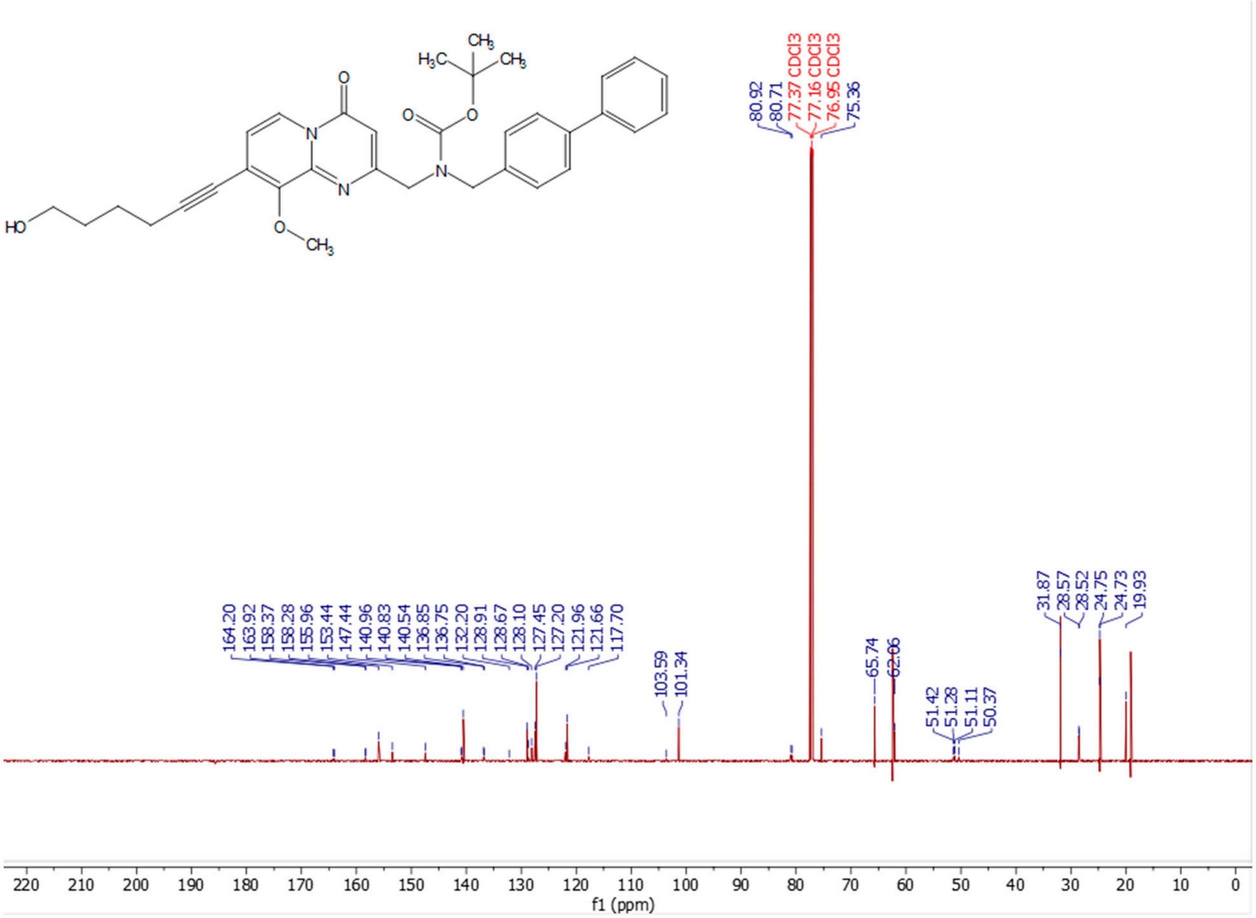

HO
O
CH3
N
N
O
O
N
H3C
CH3
CH3
80.92
80.71
77.37 CDCl3
77.16 CDCl3
76.95 CDCl3
75.36
164.20
163.92
158.37
158.28
155.96
153.44
147.44
140.96
140.83
140.54
136.85
136.75
132.20
128.91
128.67
128.10
127.45
127.20
121.96
121.66
117.70
103.59
101.34
65.74
62.06
51.42
51.28
51.11
50.37
31.87
28.57
28.52
24.75
24.73
19.93
220 210 200 190 180 170 160 150 140 130 120 110 100 90 80 70 60 50 40 30 20 10 0
f1 (ppm)

^{1}H NMR of **64**

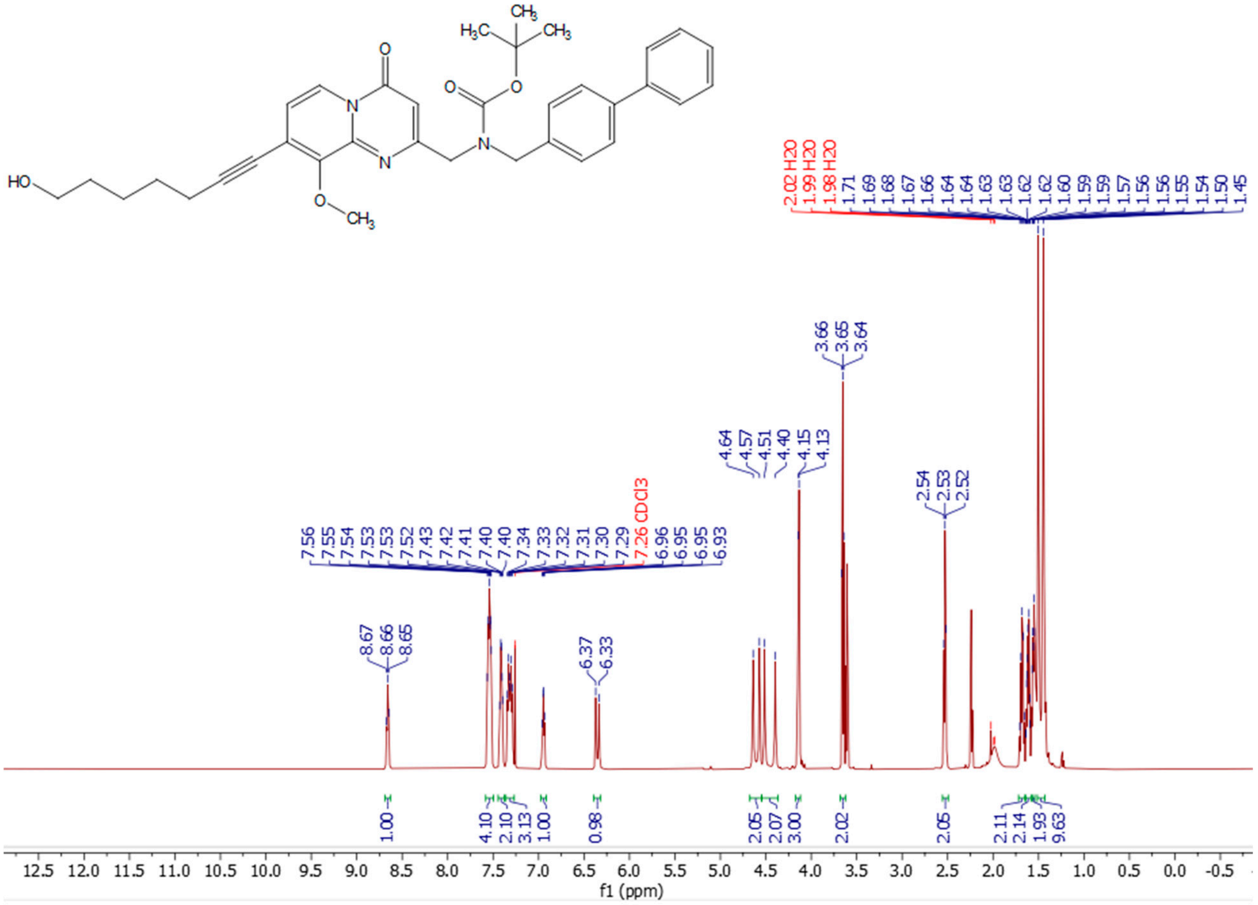

^{13}C NMR of **64**

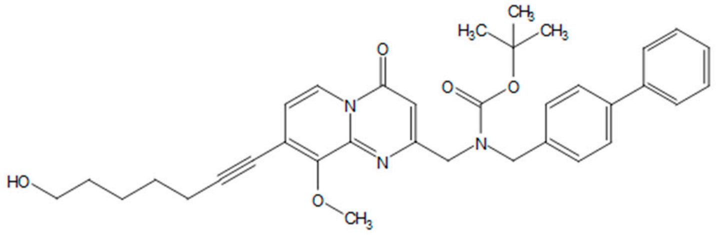

HO
O
CH3
H3C
CH3
O
N
N
O
O
N
O
CH3

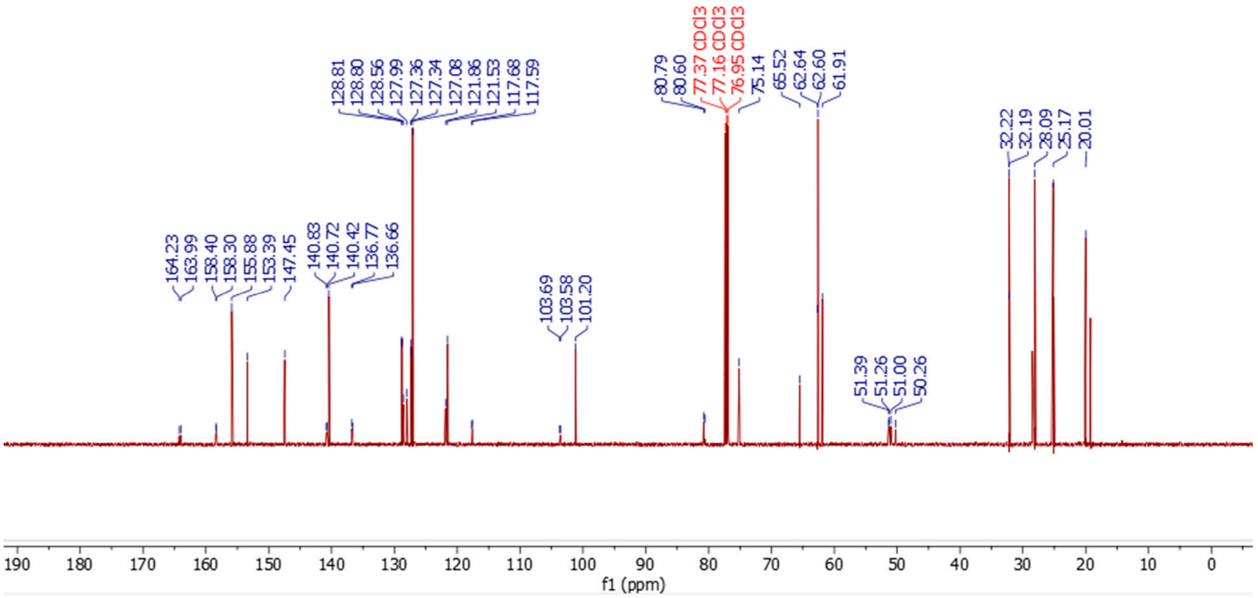

164.23
163.99
158.40
158.30
155.88
153.39
147.45
140.83
140.72
140.42
136.77
136.66
128.81
128.80
128.56
127.99
127.36
127.34
127.08
121.86
121.53
117.68
117.59
103.69
103.58
101.20
80.79
80.60
77.37 CDCl3
77.16 CDCl3
76.95 CDCl3
75.14
65.52
62.64
62.60
61.91
51.39
51.26
51.00
50.26
32.22
32.19
28.09
25.17
20.01
190 180 170 160 150 140 130 120 110 100 90 80 70 60 50 40 30 20 10 0
f1 (ppm)

^{1}H NMR of **65**

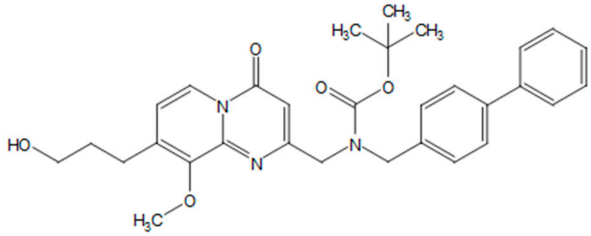
HO
O
N
N
O
O
O
N
CH_3
H_3C
CH_3
H_3C

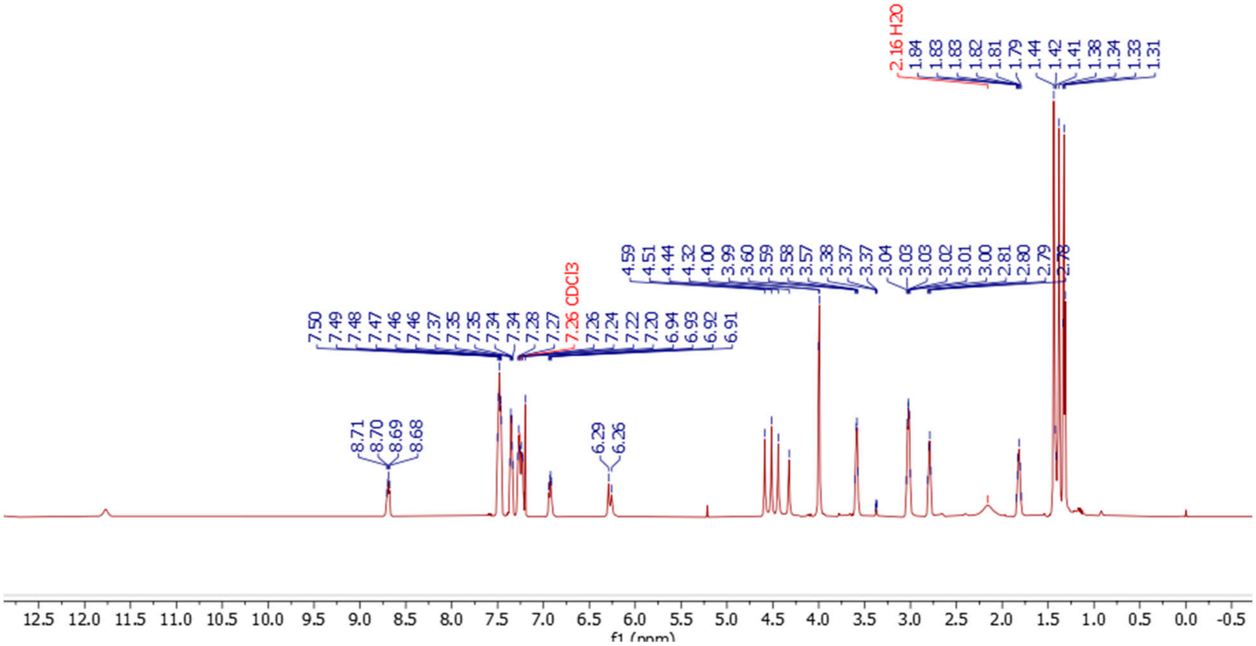
2.16 H2O
1.84
1.83
1.83
1.82
1.81
1.79
1.44
1.42
1.41
1.38
1.34
1.33
1.31
4.59
4.51
4.44
4.32
4.00
3.99
3.60
3.59
3.58
3.57
3.38
3.37
3.37
3.04
3.03
3.03
3.02
3.01
3.00
2.81
2.80
2.79
2.78
7.50
7.49
7.48
7.47
7.46
7.46
7.37
7.35
7.35
7.34
7.34
7.28
7.27
7.26 CDCl3
7.26
7.24
7.22
7.20
6.94
6.93
6.92
6.91
8.71
8.70
8.69
8.68
6.29
6.26
12.5 12.0 11.5 11.0 10.5 10.0 9.5 9.0 8.5 8.0 7.5 7.0 6.5 6.0 5.5 5.0 4.5 4.0 3.5 3.0 2.5 2.0 1.5 1.0 0.5 0.0 -0.5
f1 (ppm)

^{13}C NMR of **65**

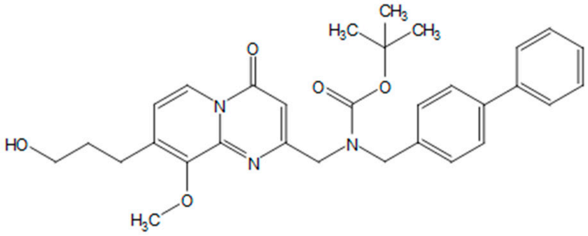
O
O
O
N
N
N
HO
H_3C
O
CH_3
H_3C
CH_3
O

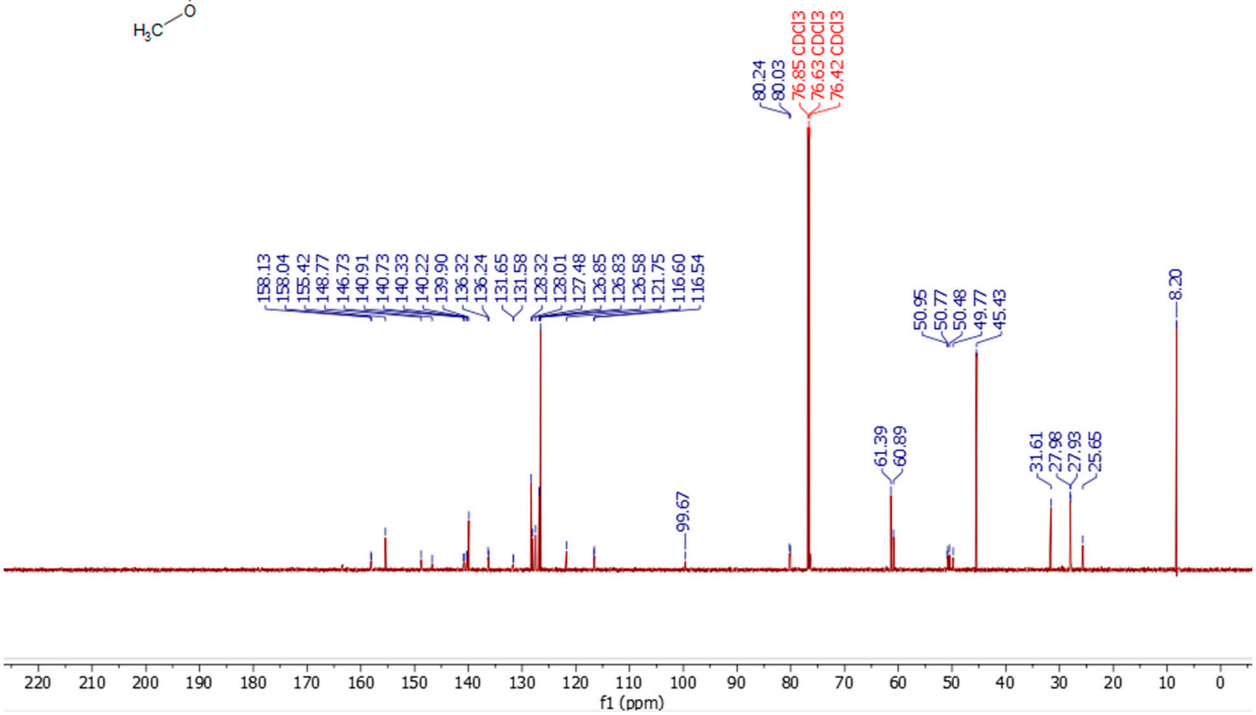
158.13
158.04
155.42
148.77
146.73
140.91
140.73
140.33
140.22
139.90
136.32
136.24
131.65
131.58
128.32
128.01
127.48
126.85
126.83
126.58
121.75
116.60
116.54
99.67
80.24
80.03
76.85 CDCl3
76.63 CDCl3
76.42 CDCl3
61.39
60.89
50.95
50.77
50.48
49.77
45.43
31.61
27.98
27.93
25.65
8.20
220
210
200
190
180
170
160
150
140
130
120
110
100
90
80
70
60
50
40
30
20
10
0
f1 (ppm)

^{1}H NMR of **66**

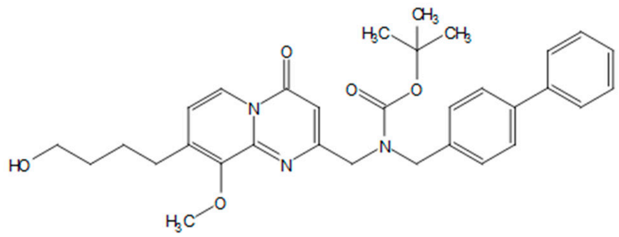

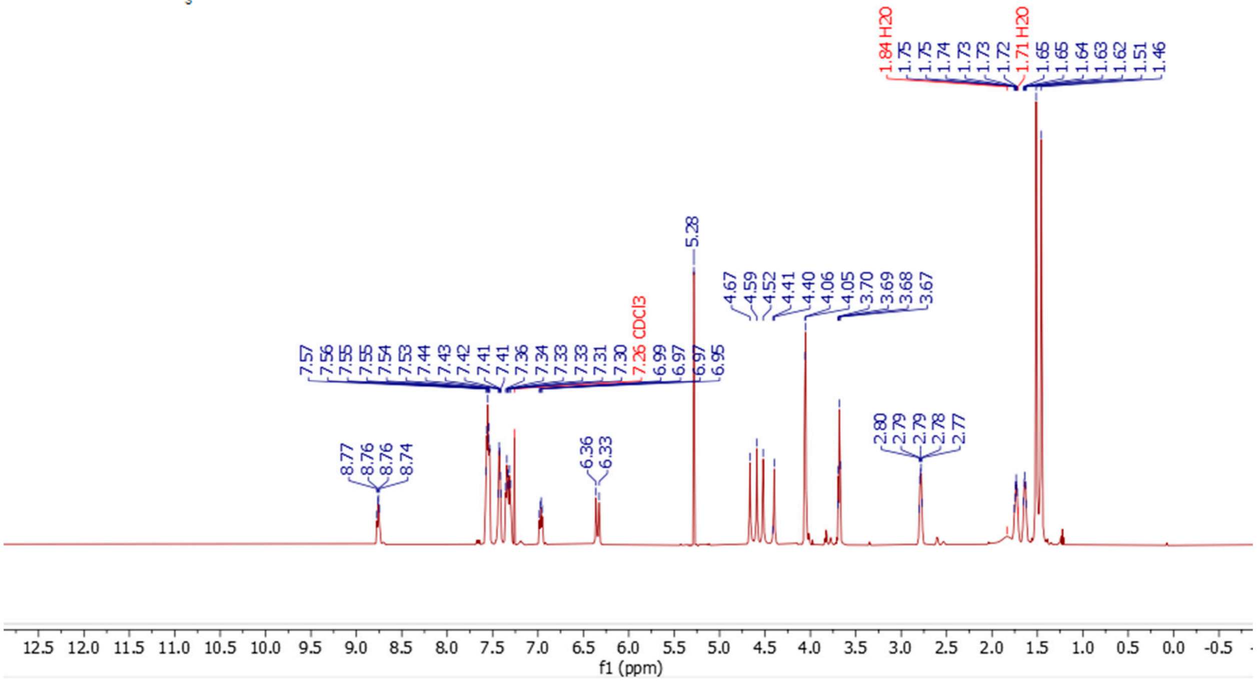

1.84 H2O
1.75
1.75
1.74
1.73
1.73
1.72
1.71 H2O
1.65
1.65
1.64
1.63
1.62
1.51
1.46
5.28
4.67
4.59
4.52
4.41
4.40
4.06
4.05
3.70
3.69
3.68
3.67
7.57
7.56
7.55
7.55
7.54
7.53
7.44
7.43
7.42
7.41
7.41
7.36
7.34
7.33
7.33
7.31
7.30
7.26 CDCl3
6.99
6.97
6.97
6.95
2.80
2.79
2.79
2.78
2.77
8.77
8.76
8.76
8.74
6.36
6.33
12.5 12.0 11.5 11.0 10.5 10.0 9.5 9.0 8.5 8.0 7.5 7.0 6.5 6.0 5.5 5.0 4.5 4.0 3.5 3.0 2.5 2.0 1.5 1.0 0.5 0.0 -0.5
f1 (ppm)

^{13}C NMR of **66**

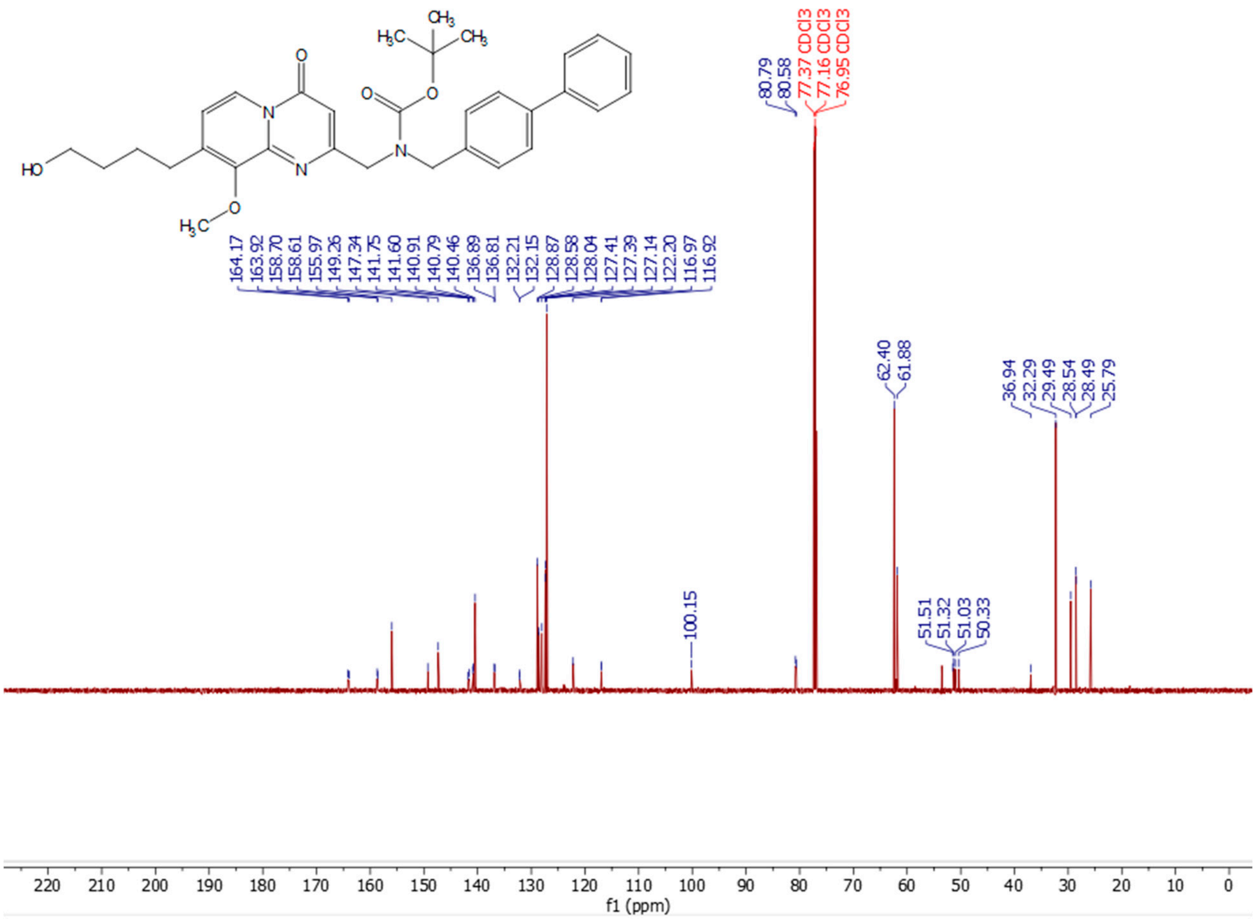

164.17
163.92
158.70
158.61
155.97
149.26
147.34
141.75
141.60
140.91
140.79
140.46
136.89
136.81
132.21
132.15
128.87
128.58
128.04
127.41
127.39
127.14
122.20
116.97
116.92
100.15
80.79
80.58
77.37 CDCl3
77.16 CDCl3
76.95 CDCl3
62.40
61.88
51.51
51.32
51.03
50.33
36.94
32.29
29.49
28.54
28.49
25.79
f1 (ppm)

^{1}H NMR of **67**

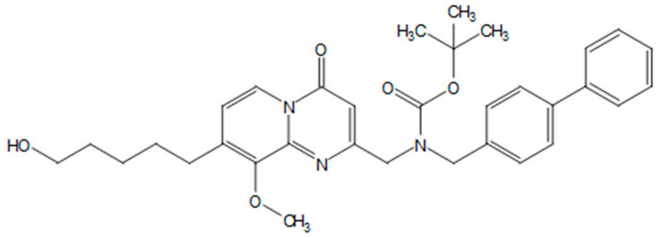

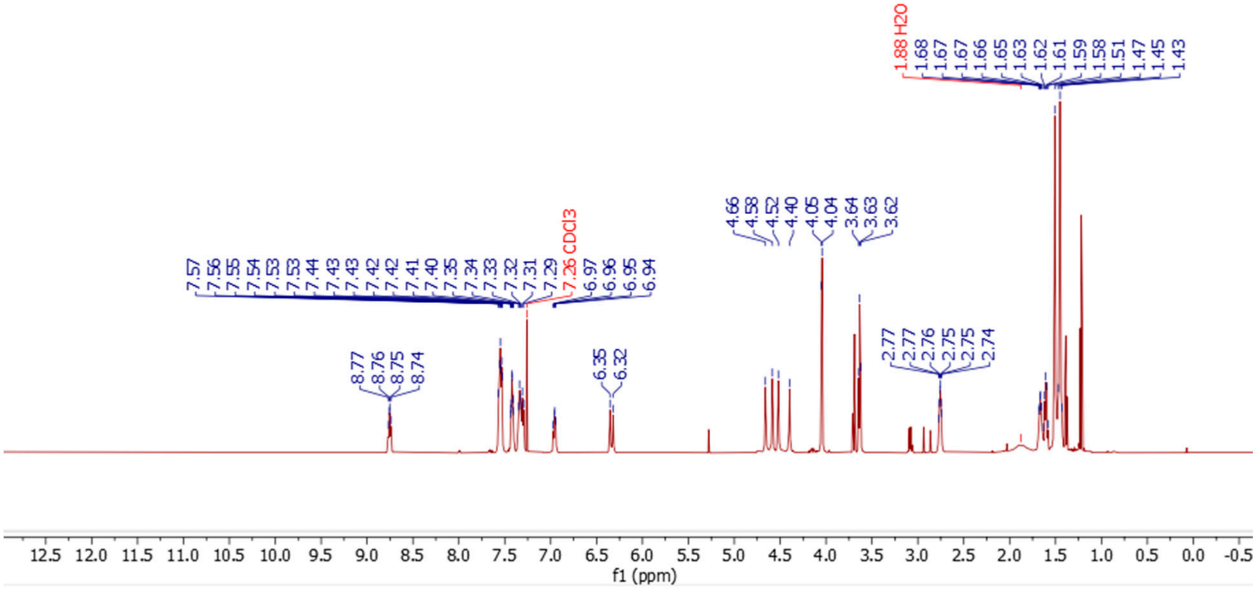
1.88 H2O
1.68
1.67
1.67
1.66
1.65
1.63
1.62
1.61
1.59
1.58
1.51
1.47
1.45
1.43
4.66
4.58
4.52
4.40
4.05
4.04
3.64
3.63
3.62
7.57
7.56
7.55
7.54
7.53
7.53
7.44
7.43
7.43
7.42
7.42
7.41
7.40
7.35
7.34
7.33
7.32
7.31
7.29
7.26 CDCl3
6.97
6.96
6.95
6.94
8.77
8.76
8.75
8.74
6.35
6.32
2.77
2.77
2.76
2.75
2.75
2.74
12.5 12.0 11.5 11.0 10.5 10.0 9.5 9.0 8.5 8.0 7.5 7.0 6.5 6.0 5.5 5.0 4.5 4.0 3.5 3.0 2.5 2.0 1.5 1.0 0.5 0.0 -0.5
f1 (ppm)

^{13}C NMR of **67**

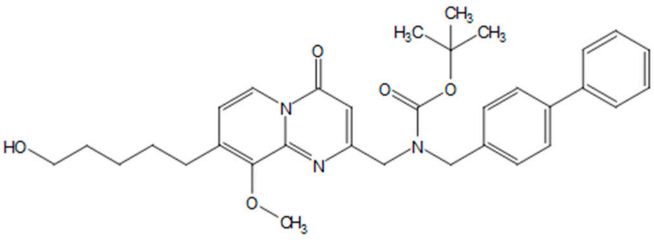

HO
O
O
N
N
O
O
N
O
CH_3
H_3C
CH_3
CH_3

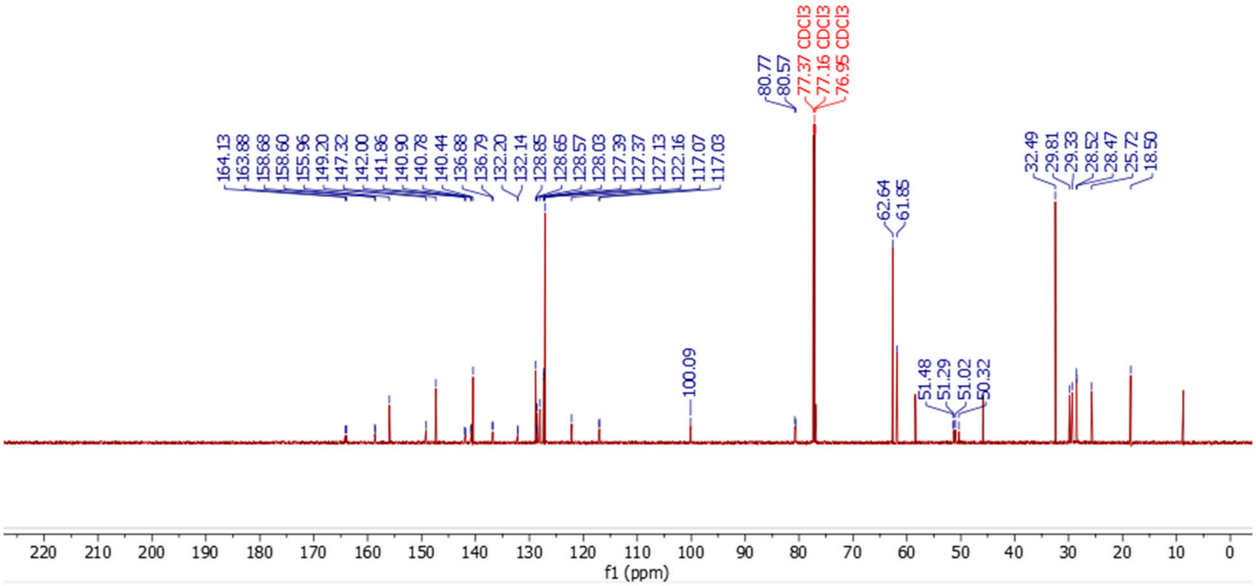

164.13
163.88
158.68
158.60
155.96
149.20
147.32
142.00
141.86
140.90
140.78
140.44
136.88
136.79
132.20
132.14
128.85
128.65
128.57
128.03
127.39
127.37
127.13
122.16
117.07
117.03
100.09
80.77
80.57
77.37 CDCl3
77.16 CDCl3
76.95 CDCl3
62.64
61.85
51.48
51.29
51.02
50.32
32.49
29.81
29.33
28.52
28.47
25.72
18.50
220
210
200
190
180
170
160
150
140
130
120
110
100
90
80
70
60
50
40
30
20
10
0
f1 (ppm)

^{1}H NMR of **68**

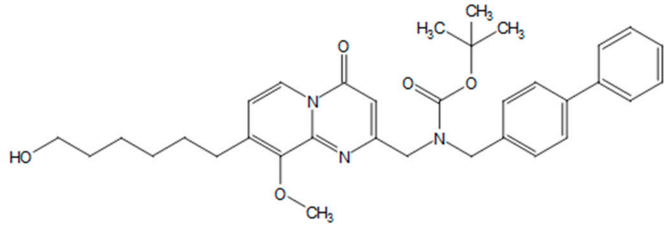

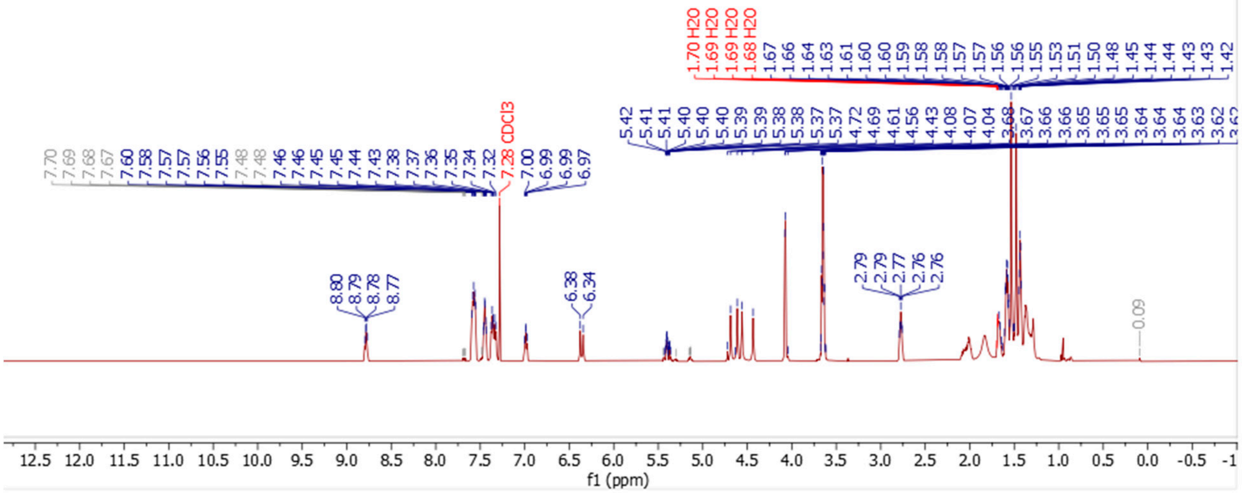

^{13}C NMR of **68**

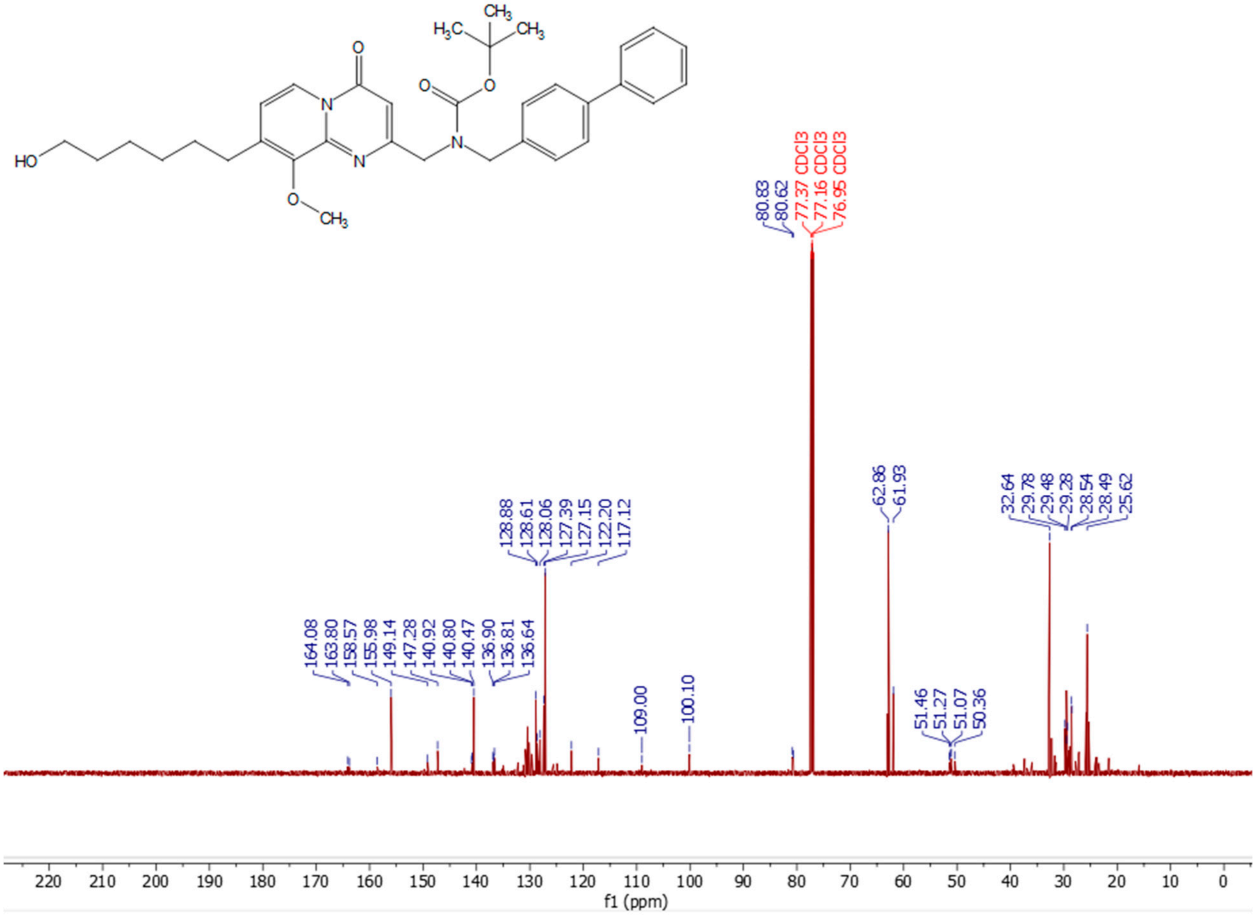

HO
O
CH3
N
N
O
O
N
O
H3C
CH3
CH3
80.83
80.62
77.37 CDCl3
77.16 CDCl3
76.95 CDCl3
164.08
163.80
158.57
155.98
149.14
147.28
140.92
140.80
140.47
136.90
136.81
136.64
128.88
128.61
128.06
127.39
127.15
122.20
117.12
109.00
100.10
62.86
61.93
51.46
51.27
51.07
50.36
32.64
29.78
29.48
29.28
28.54
28.49
25.62
f1 (ppm)

^{1}H NMR of **69**

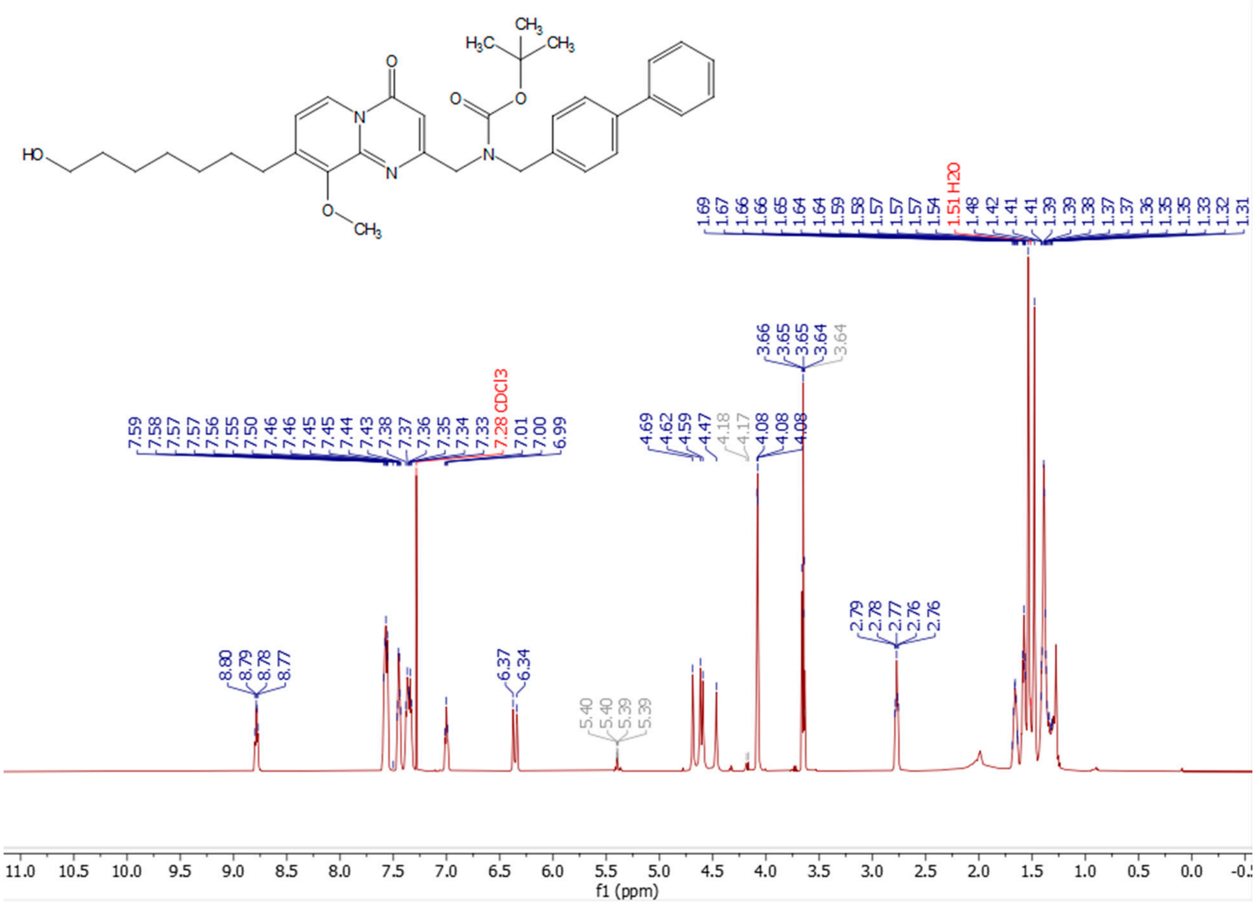

^{13}C NMR of **69**

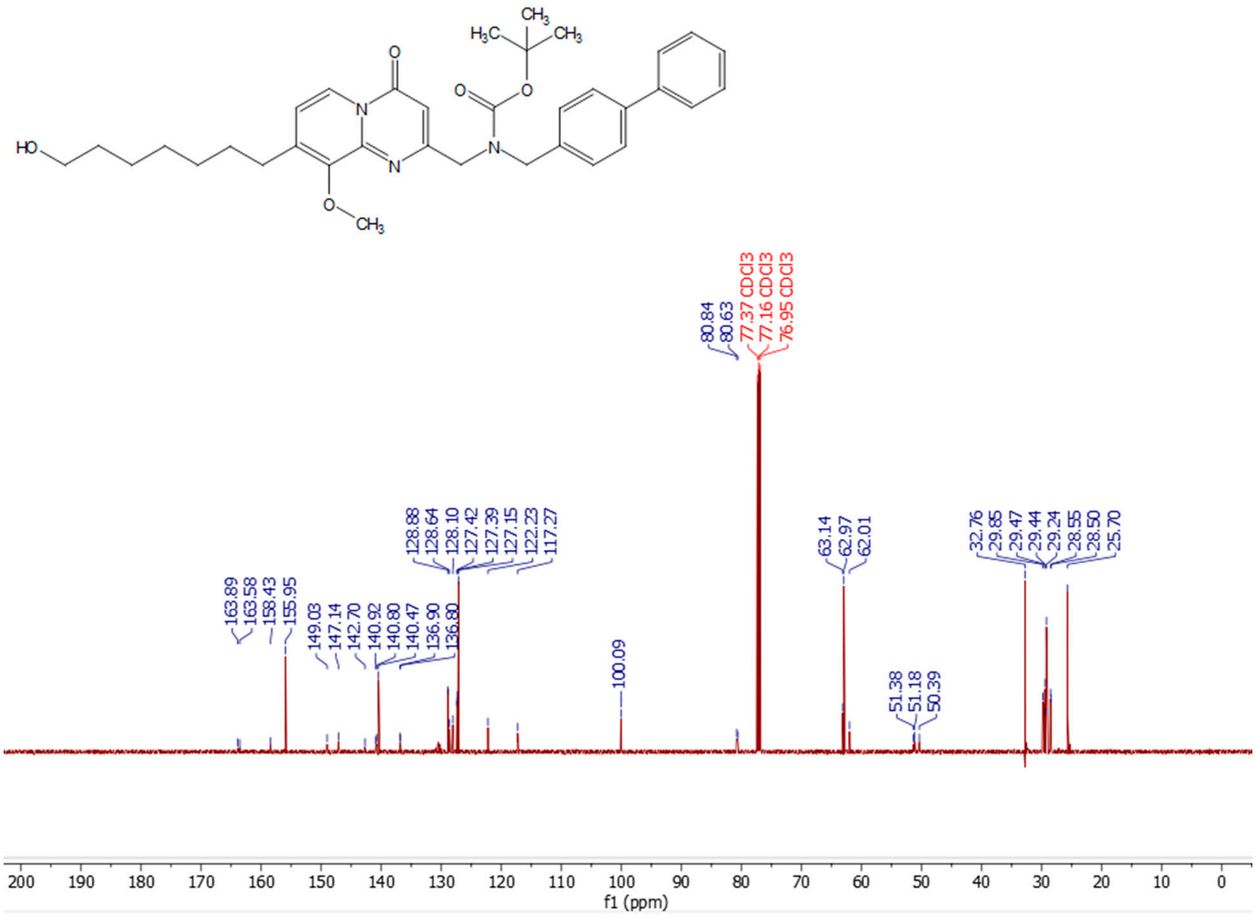
HO
O
N
N
O
O
O
N
O
H_3C
CH_3
CH_3
CH_3
163.89
163.58
158.43
155.95
149.03
147.14
142.70
140.92
140.80
140.47
136.90
136.80
128.88
128.64
128.10
127.42
127.39
127.15
122.23
117.27
100.09
80.84
80.63
77.37 CDCl3
77.16 CDCl3
76.95 CDCl3
63.14
62.97
62.01
51.38
51.18
50.39
32.76
29.85
29.47
29.44
29.24
28.55
28.50
25.70
200 190 180 170 160 150 140 130 120 110 100 90 80 70 60 50 40 30 20 10 0
f1 (ppm)

^{1}H NMR of **70**

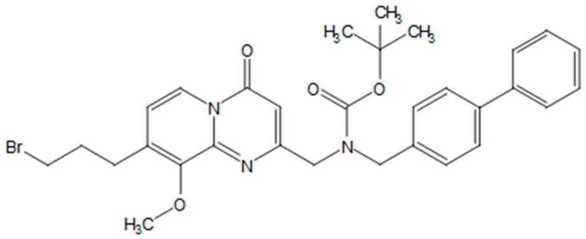

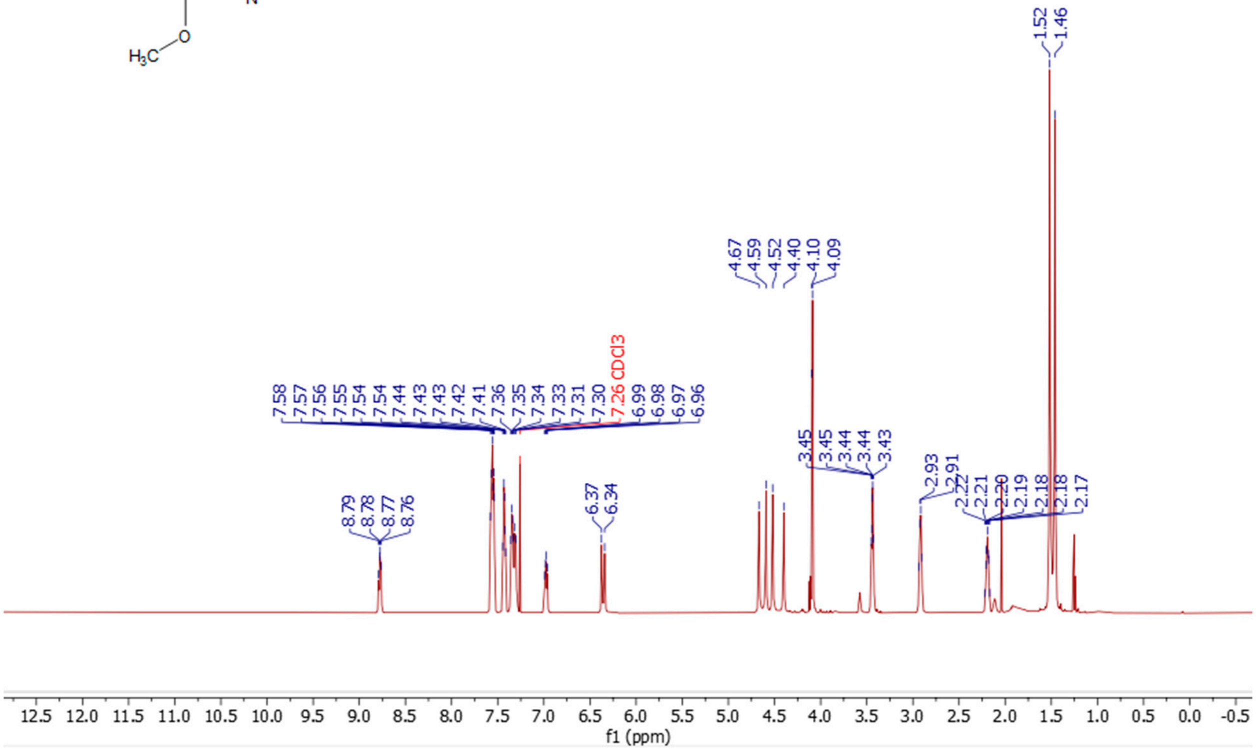
7.58
7.57
7.56
7.55
7.54
7.54
7.44
7.43
7.43
7.42
7.41
7.36
7.35
7.34
7.33
7.31
7.30
7.26 CDCl3
6.99
6.98
6.97
6.96
8.79
8.78
8.77
8.76
6.37
6.34
4.67
4.59
4.52
4.40
4.10
4.09
3.45
3.45
3.44
3.44
3.43
2.93
2.91
2.22
2.21
2.20
2.19
2.18
2.18
2.17
1.52
1.46
f1 (ppm)

^{13}C NMR of **70**

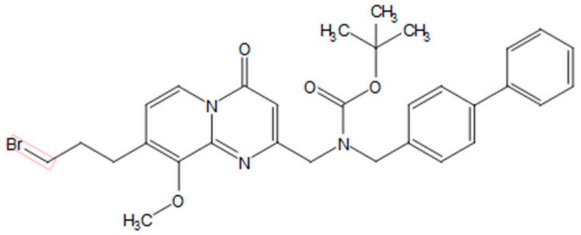

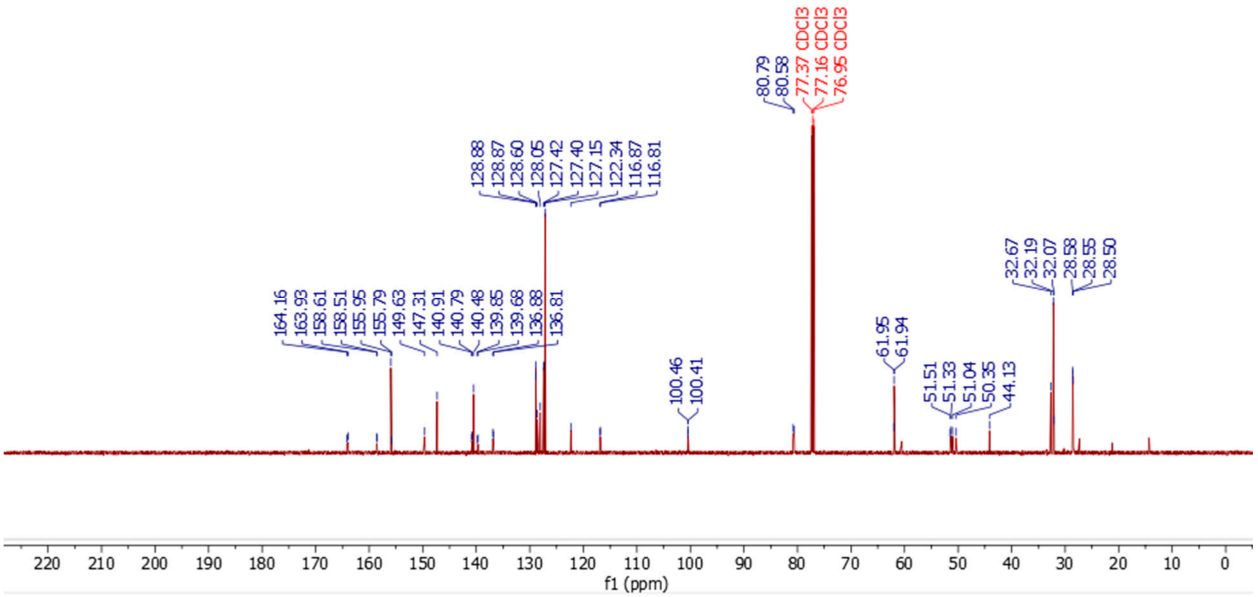

164.16
163.93
158.61
158.51
155.95
155.79
149.63
147.31
140.91
140.79
140.48
139.85
139.68
136.88
136.81
128.88
128.87
128.60
128.05
127.42
127.40
127.15
122.34
116.87
116.81
100.46
100.41
80.79
80.58
77.37 CDCl3
77.16 CDCl3
76.95 CDCl3
61.95
61.94
51.51
51.33
51.04
50.35
44.13
32.67
32.19
32.07
28.58
28.55
28.50
f1 (ppm)

^{1}H NMR of **71**

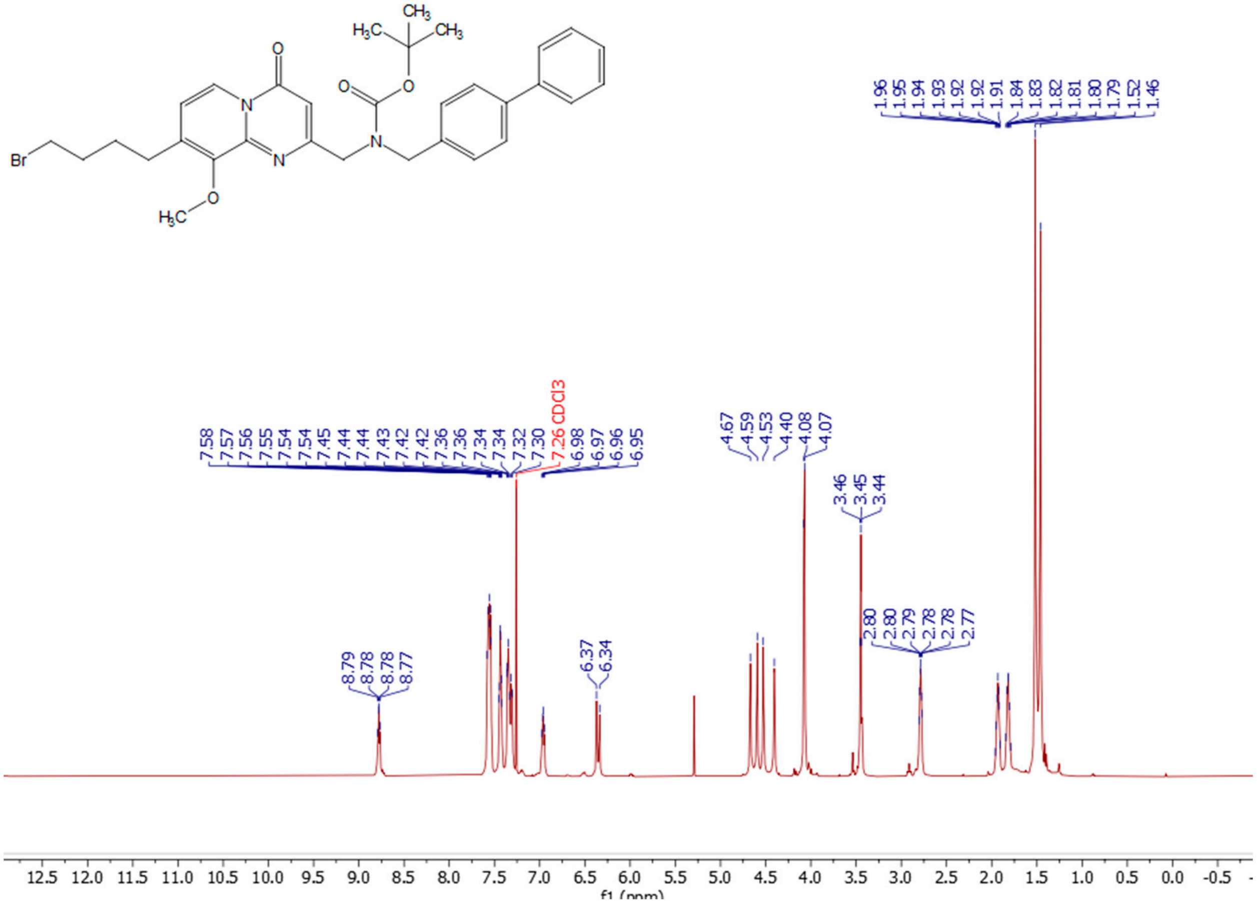

^{13}C NMR of **71**

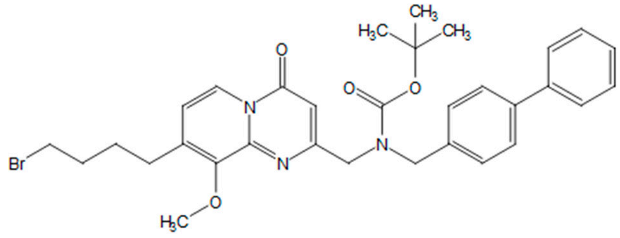

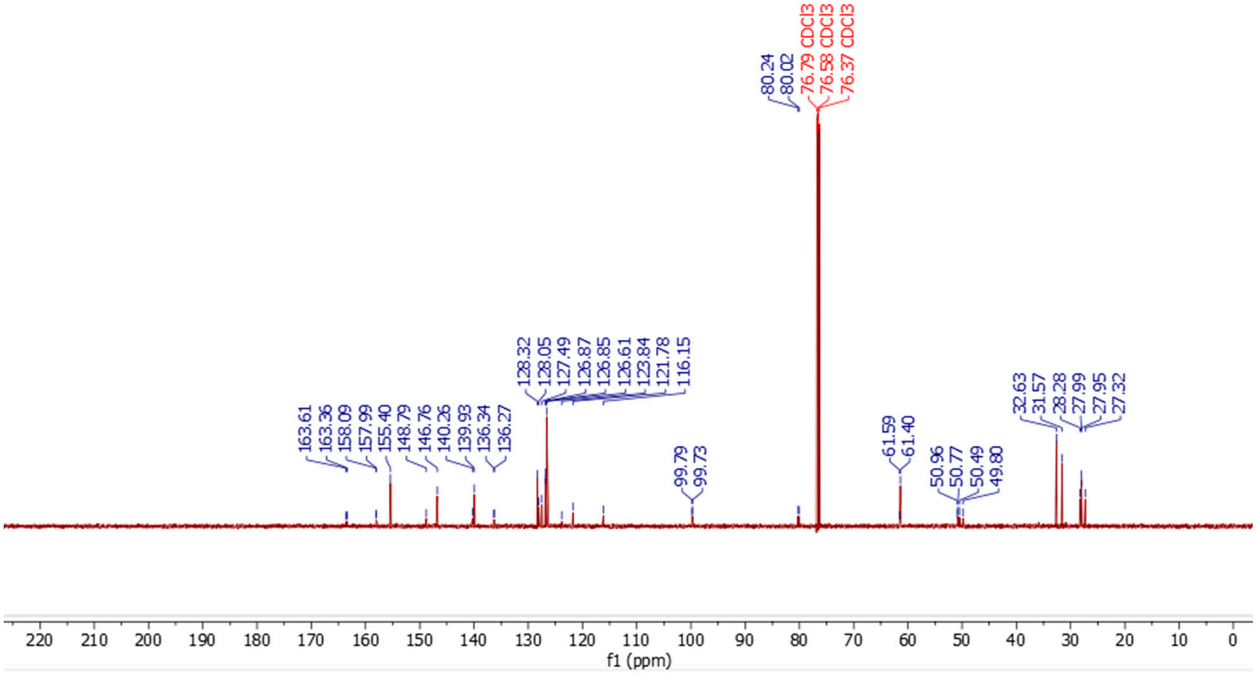

^{1}H NMR of **72**

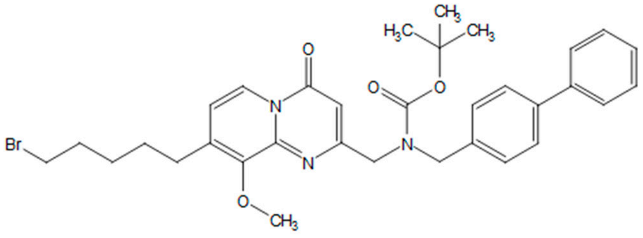

Br
O
N
N
O
CH3
O
N
O
O
H3C
CH3
CH3

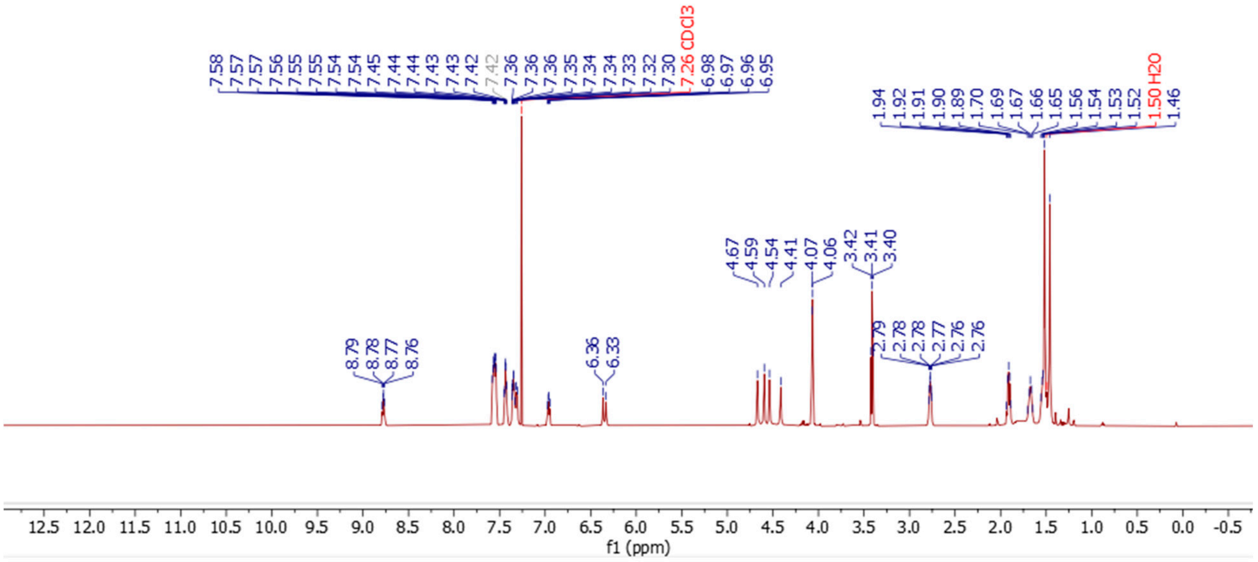

7.58
7.57
7.57
7.56
7.55
7.55
7.54
7.54
7.45
7.44
7.44
7.43
7.43
7.42
7.42
7.36
7.36
7.36
7.35
7.34
7.34
7.33
7.32
7.30
7.26 CDCl3
6.98
6.97
6.96
6.95
1.94
1.92
1.91
1.90
1.89
1.70
1.69
1.67
1.66
1.65
1.56
1.54
1.53
1.52
1.50 H2O
1.46
4.67
4.59
4.54
4.41
4.07
4.06
3.42
3.41
3.40
8.79
8.78
8.77
8.76
6.36
6.33
2.79
2.78
2.78
2.77
2.76
2.76
12.5
12.0
11.5
11.0
10.5
10.0
9.5
9.0
8.5
8.0
7.5
7.0
6.5
6.0
5.5
5.0
4.5
4.0
3.5
3.0
2.5
2.0
1.5
1.0
0.5
0.0
-0.5
f1 (ppm)

^{13}C NMR of **72**

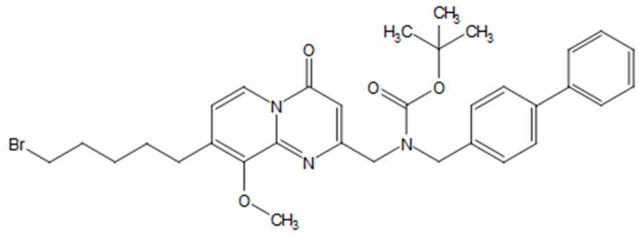
O
H_3C
CH_3
CH_3
O
O
N
N
N
Br
O
CH_3

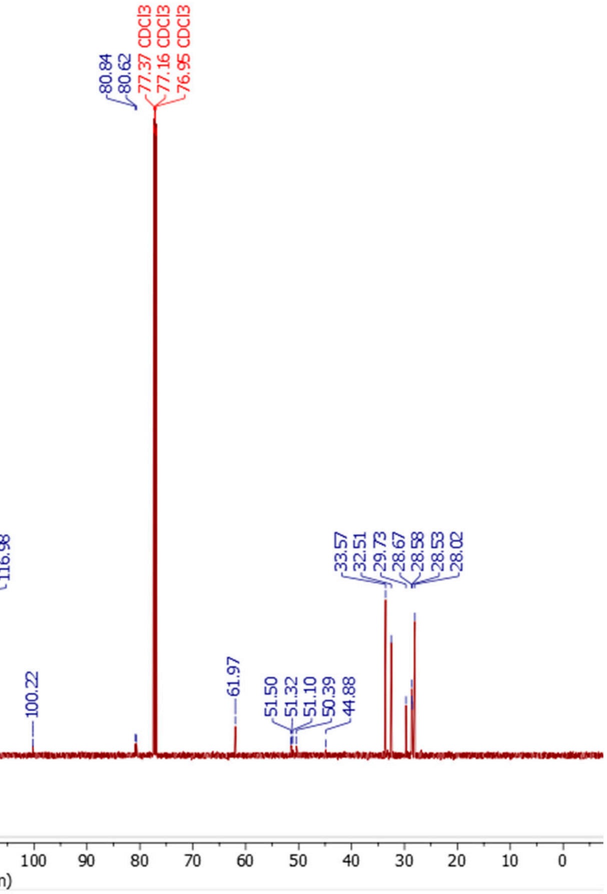
80.84
80.62
77.37 CDCl3
77.16 CDCl3
76.95 CDCl3
164.13
158.62
155.98
149.27
147.31
140.96
140.84
140.51
136.92
136.85
128.90
128.64
128.09
127.45
127.43
127.19
122.30
116.98
100.22
61.97
51.50
51.32
51.10
50.39
44.88
33.57
32.51
29.73
28.67
28.58
28.53
28.02
220 210 200 190 180 170 160 150 140 130 120 110 100 90 80 70 60 50 40 30 20 10 0
f1 (ppm)

^{1}H NMR of **73**

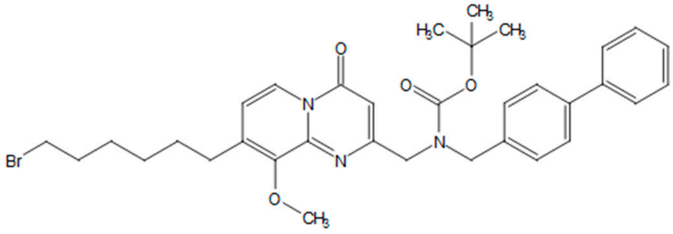

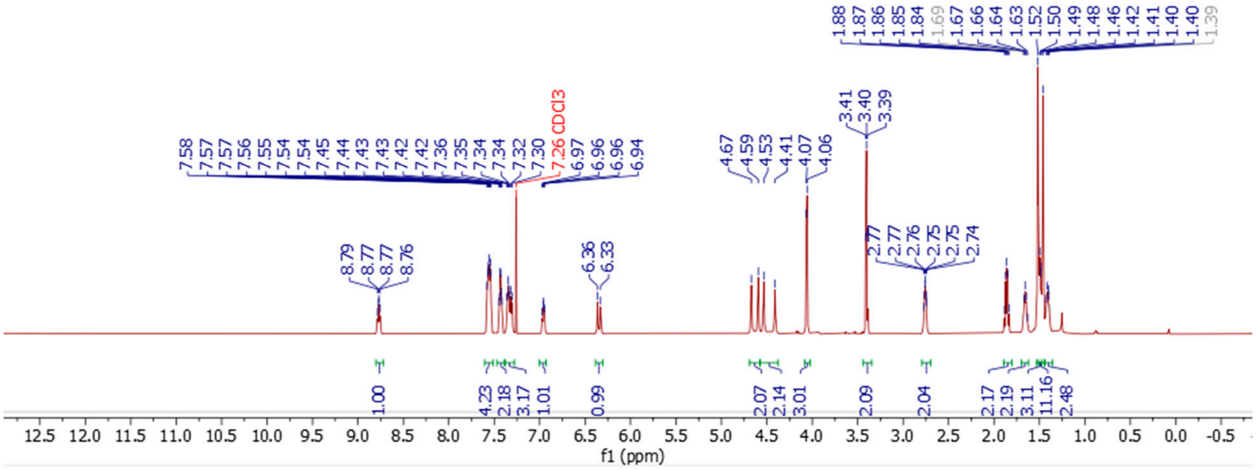

1.88
1.87
1.86
1.85
1.84
1.69
1.67
1.66
1.64
1.63
1.52
1.50
1.49
1.48
1.46
1.42
1.41
1.40
1.40
1.39
7.58
7.57
7.57
7.56
7.55
7.54
7.54
7.45
7.44
7.43
7.43
7.42
7.42
7.36
7.35
7.34
7.34
7.32
7.30
7.26 CDCl3
6.97
6.96
6.96
6.94
4.67
4.59
4.53
4.41
4.07
4.06
3.41
3.40
3.39
8.79
8.77
8.77
8.76
6.36
6.33
2.77
2.77
2.76
2.75
2.75
2.74
1.00
4.23
2.18
3.17
1.01
0.99
2.07
2.14
3.01
2.09
2.04
2.17
2.19
3.11
11.16
2.48
12.5 12.0 11.5 11.0 10.5 10.0 9.5 9.0 8.5 8.0 7.5 7.0 6.5 6.0 5.5 5.0 4.5 4.0 3.5 3.0 2.5 2.0 1.5 1.0 0.5 0.0 -0.5
f1 (ppm)

^{13}C NMR of **73**

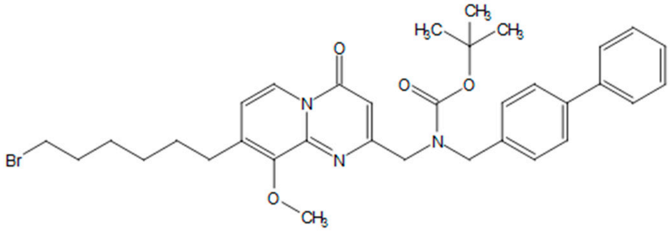

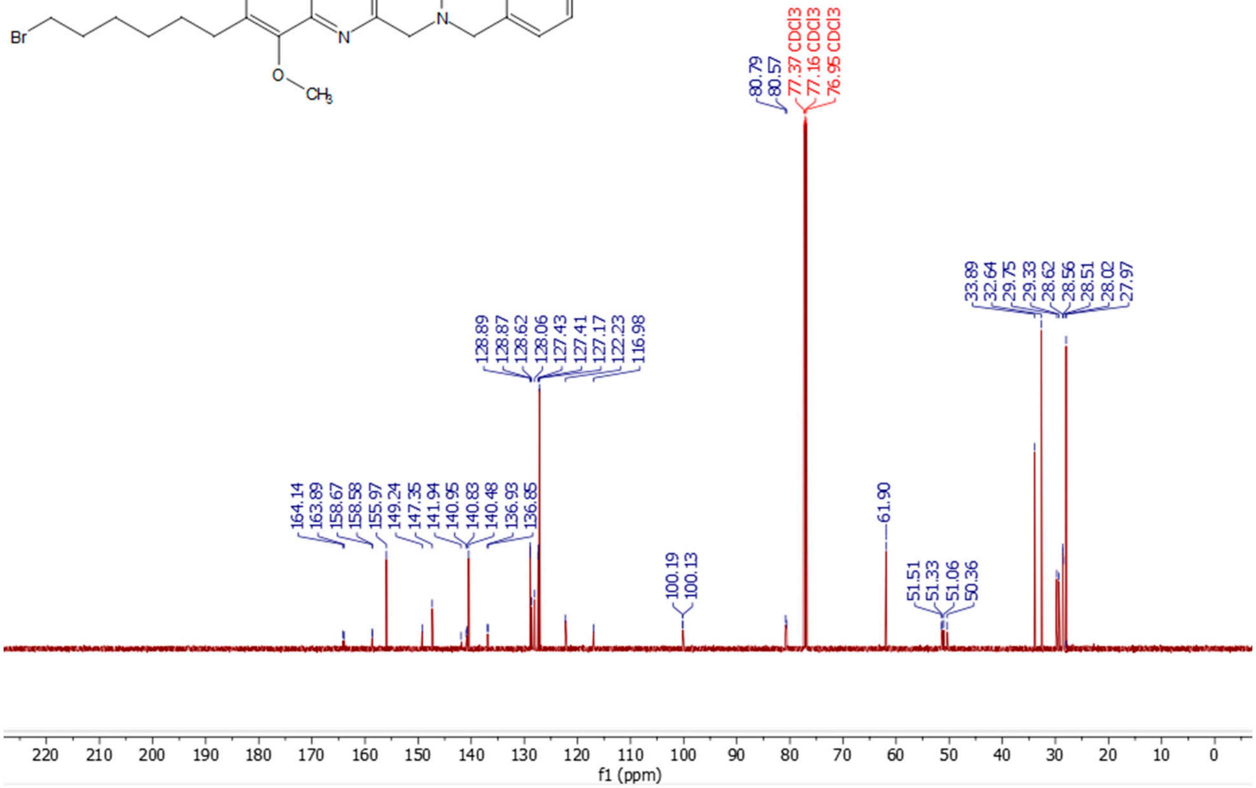

^{1}H NMR of **74**

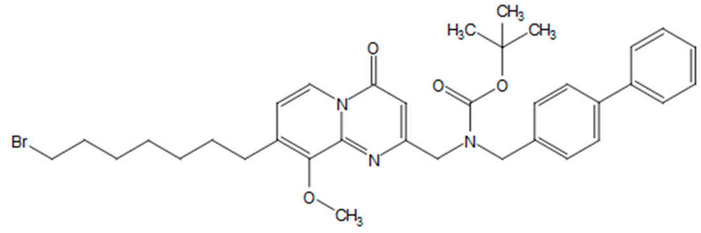

Br
O
N
N
O
CH3
O
O
N
O
H3C
CH3
CH3

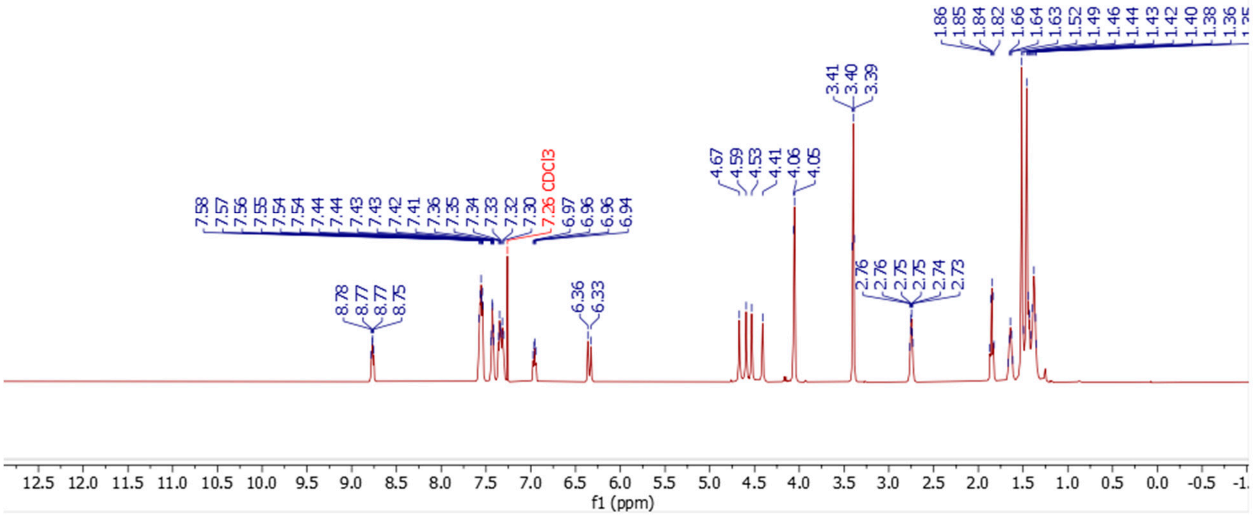

7.58 7.57 7.56 7.55 7.54 7.54 7.44 7.44 7.43 7.43 7.42 7.41 7.36 7.35 7.34 7.33 7.32 7.30
7.26 CDCl3
6.97 6.96 6.96 6.94
8.78 8.77 8.77 8.75
6.36 6.33
4.67 4.59 4.53 4.41
4.06 4.05
3.41 3.40 3.39
2.76 2.76 2.75 2.75 2.74 2.73
1.86 1.85 1.84 1.82 1.66 1.64 1.63 1.52 1.49 1.46 1.44 1.43 1.42 1.40 1.38 1.36
12.5 12.0 11.5 11.0 10.5 10.0 9.5 9.0 8.5 8.0 7.5 7.0 6.5 6.0 5.5 5.0 4.5 4.0 3.5 3.0 2.5 2.0 1.5 1.0 0.5 0.0 -0.5
f1 (ppm)

^{13}C NMR of **74**

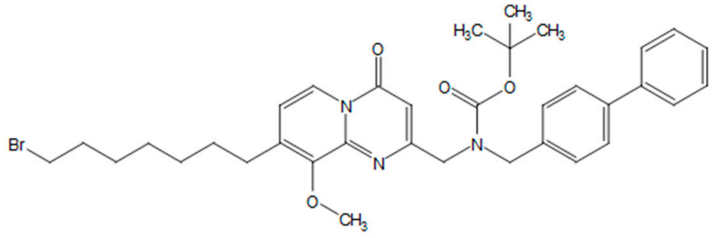

Br
O
N
N
O
CH3
O
N
O
H3C
CH3
CH3

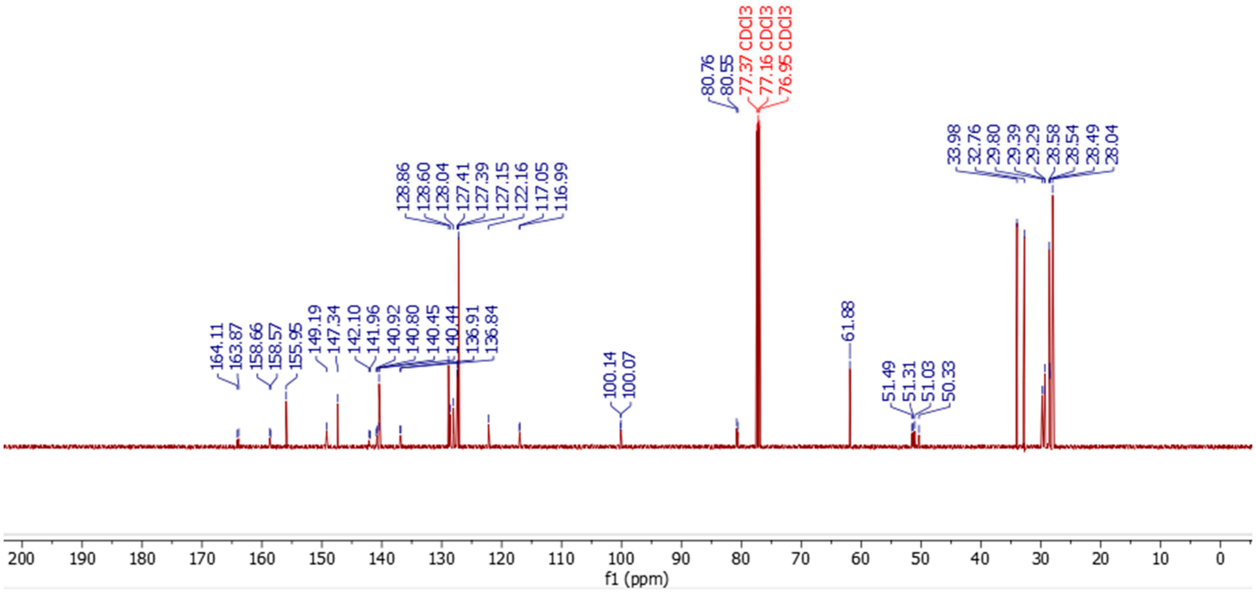

164.11
163.87
158.66
158.57
155.95
149.19
147.34
142.10
141.96
140.92
140.80
140.45
140.44
136.91
136.84
128.86
128.60
128.04
127.41
127.39
127.15
122.16
117.05
116.99
100.14
100.07
80.76
80.55
77.37 CDCl3
77.16 CDCl3
76.95 CDCl3
61.88
51.49
51.31
51.03
50.33
33.98
32.76
29.80
29.39
29.29
28.58
28.54
28.49
28.04
200
190
180
170
160
150
140
130
120
110
100
90
80
70
60
50
40
30
20
10
0
f1 (ppm)

^{1}H NMR of **80**

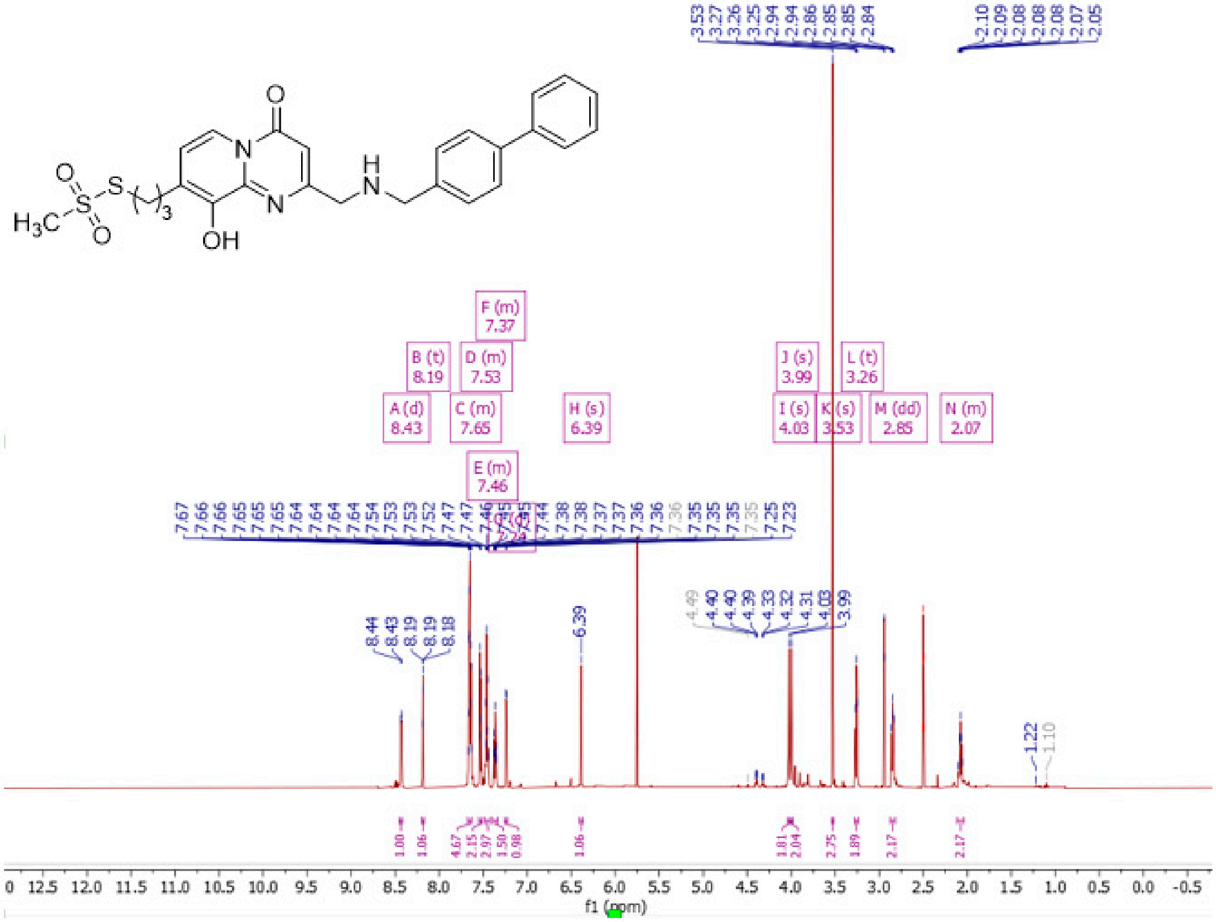

A (d) 8.43
B (t) 8.19
C (m) 7.65
D (m) 7.53
E (m) 7.46
F (m) 7.37
H (s) 6.39
I (s) 4.03
J (s) 3.99
K (s) 3.53
L (t) 3.26
M (dd) 2.85
N (m) 2.07
f1 (ppm)

^{13}C NMR of **80**

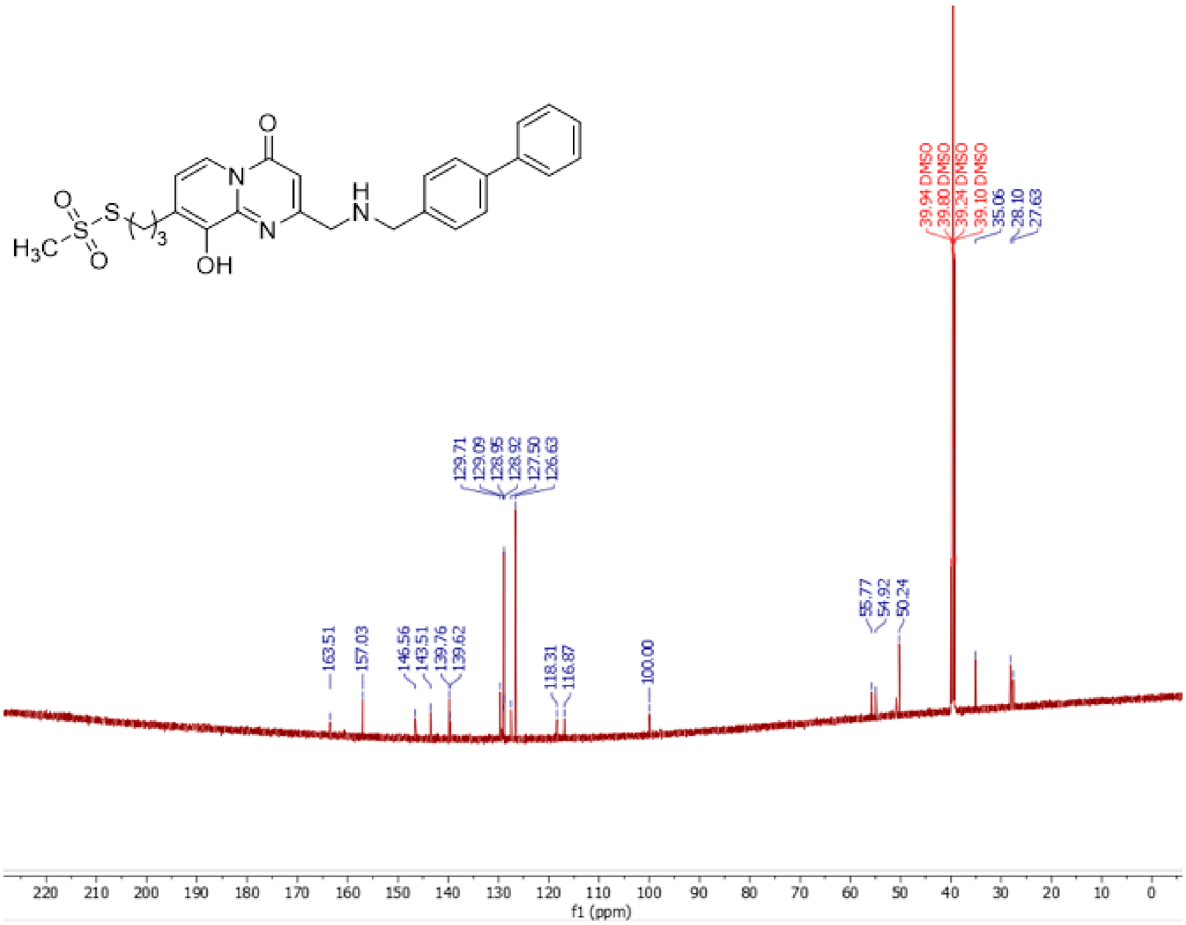

^{1}H NMR of **81**

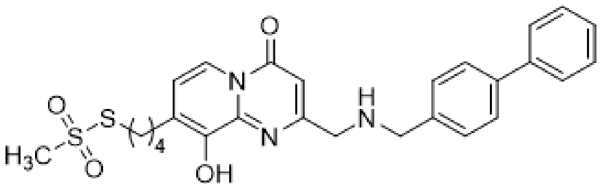

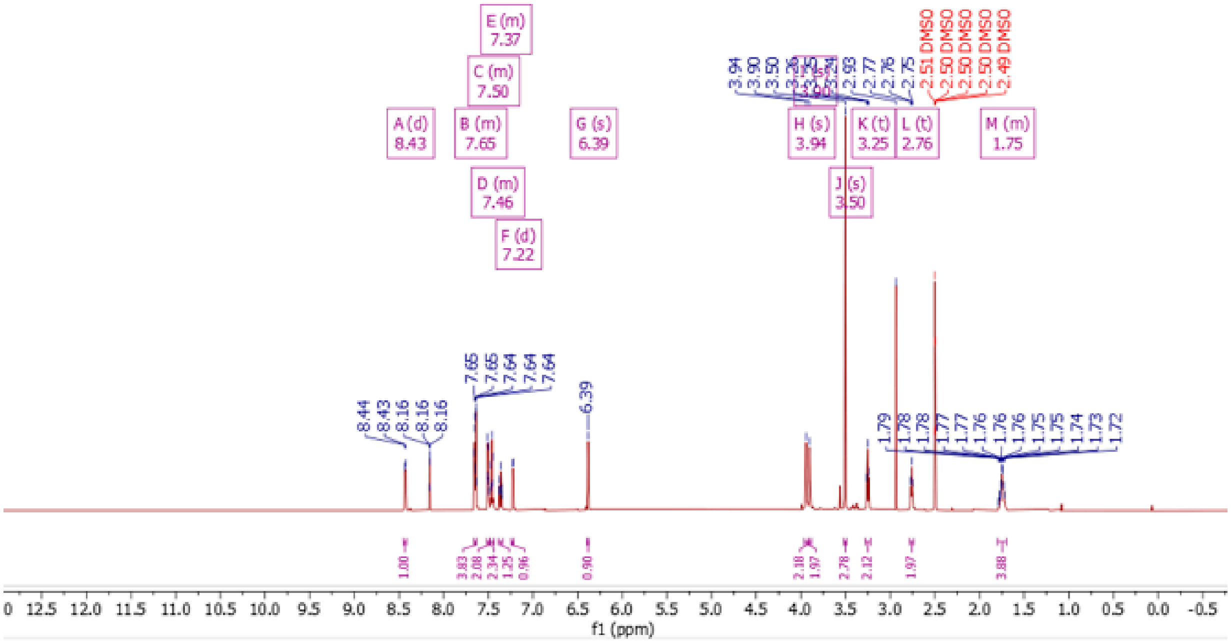

A (d)
8.43
B (m)
7.65
C (m)
7.50
D (m)
7.46
E (m)
7.37
F (d)
7.22
G (s)
6.39
H (s)
3.94
J (s)
3.50
K (t)
3.25
L (t)
2.76
M (m)
1.75
f1 (ppm)

^{13}C NMR of **81**

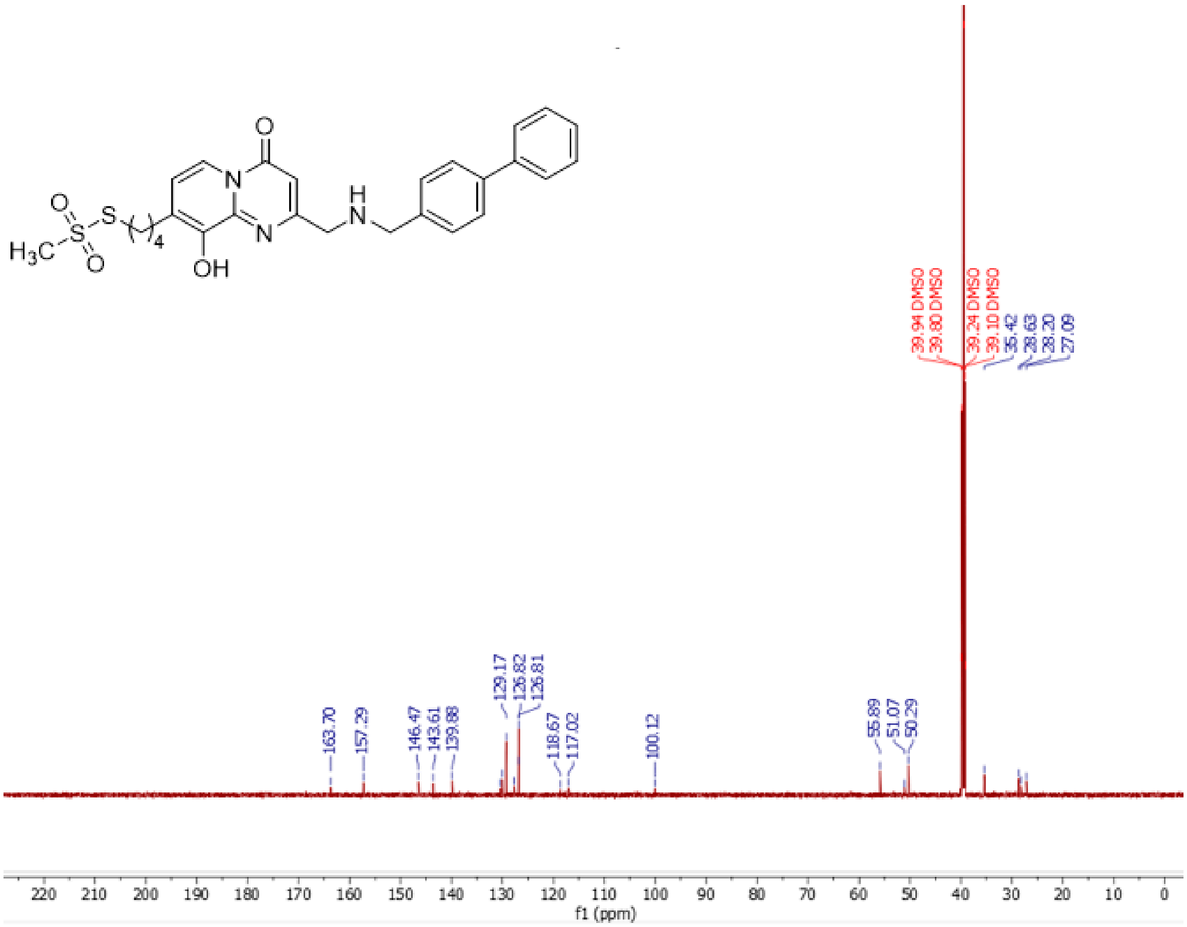

H3C
S
O
O
S
4
OH
N
N
O
H
N
39.94 DMSO
39.80 DMSO
39.24 DMSO
39.10 DMSO
35.42
28.63
28.20
27.09
163.70
157.29
146.47
143.61
139.88
129.17
126.82
126.81
118.67
117.02
100.12
55.89
51.07
50.29
220 210 200 190 180 170 160 150 140 130 120 110 100 90 80 70 60 50 40 30 20 10 0
f1 (ppm)

^{1}H NMR of **82**

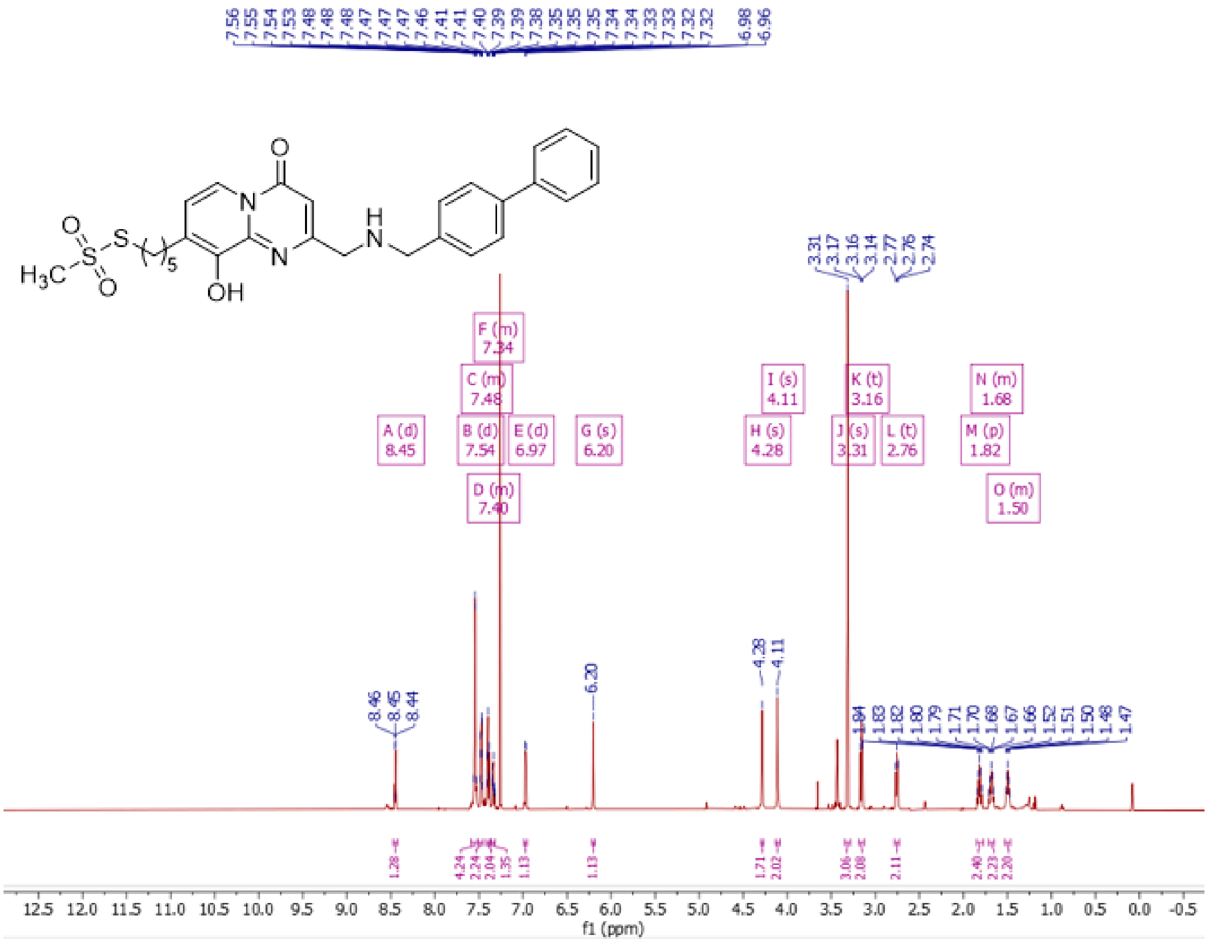
A (d) 8.45
B (d) 7.54
C (m) 7.48
D (m) 7.40
E (d) 6.97
F (m) 7.34
G (s) 6.20
H (s) 4.28
I (s) 4.11
J (s) 3.31
K (t) 3.16
L (t) 2.76
M (p) 1.82
N (m) 1.68
O (m) 1.50
f1 (ppm)

^{13}C NMR of **82**

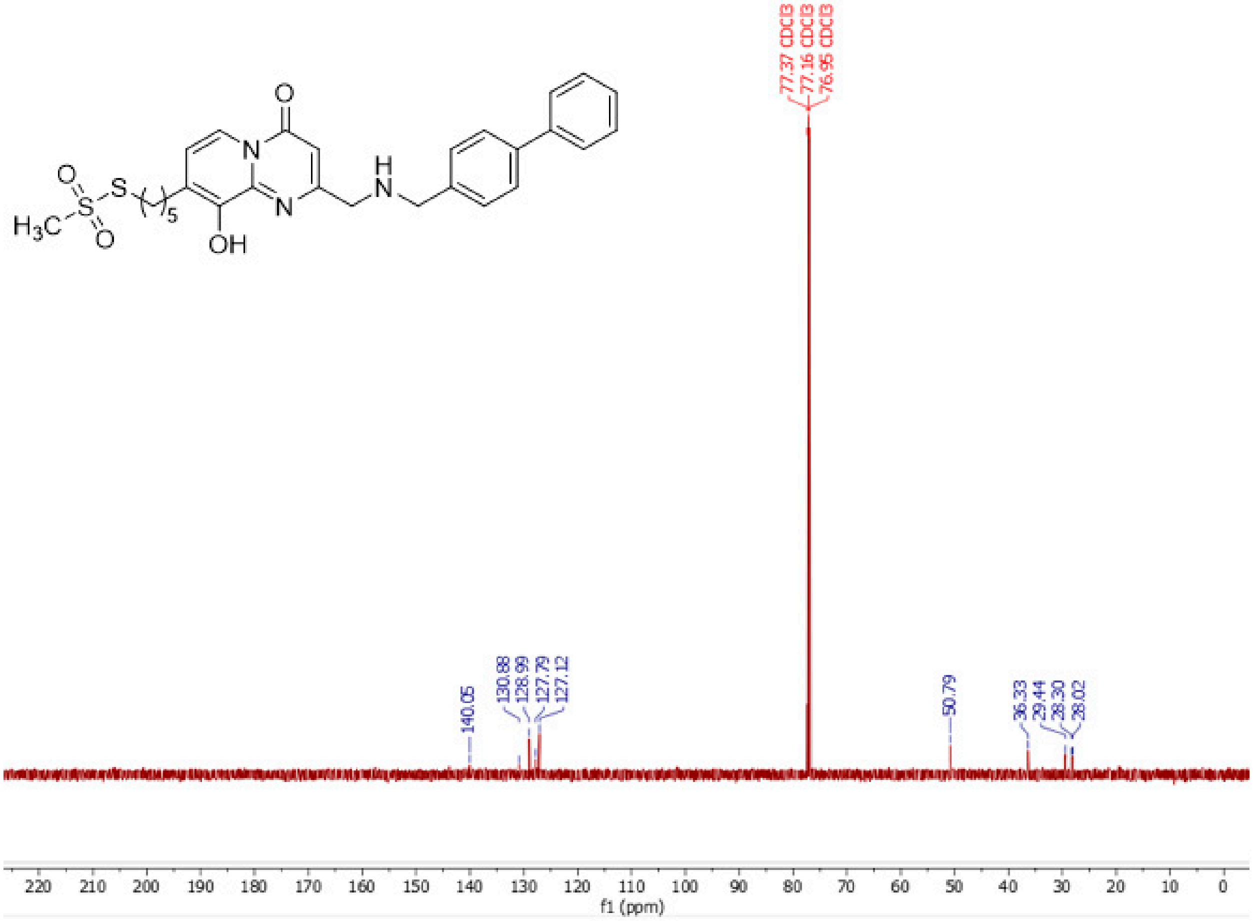

77.37 CDCl3
77.16 CDCl3
76.95 CDCl3
140.05
130.88
128.99
127.79
127.12
50.79
36.33
29.44
28.30
28.02
220 210 200 190 180 170 160 150 140 130 120 110 100 90 80 70 60 50 40 30 20 10 0
f1 (ppm)

^{1}H NMR of **83**

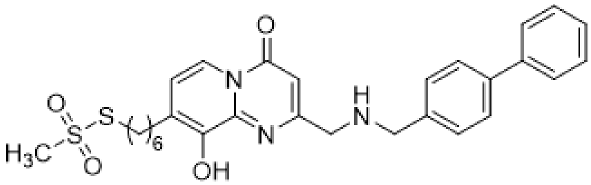

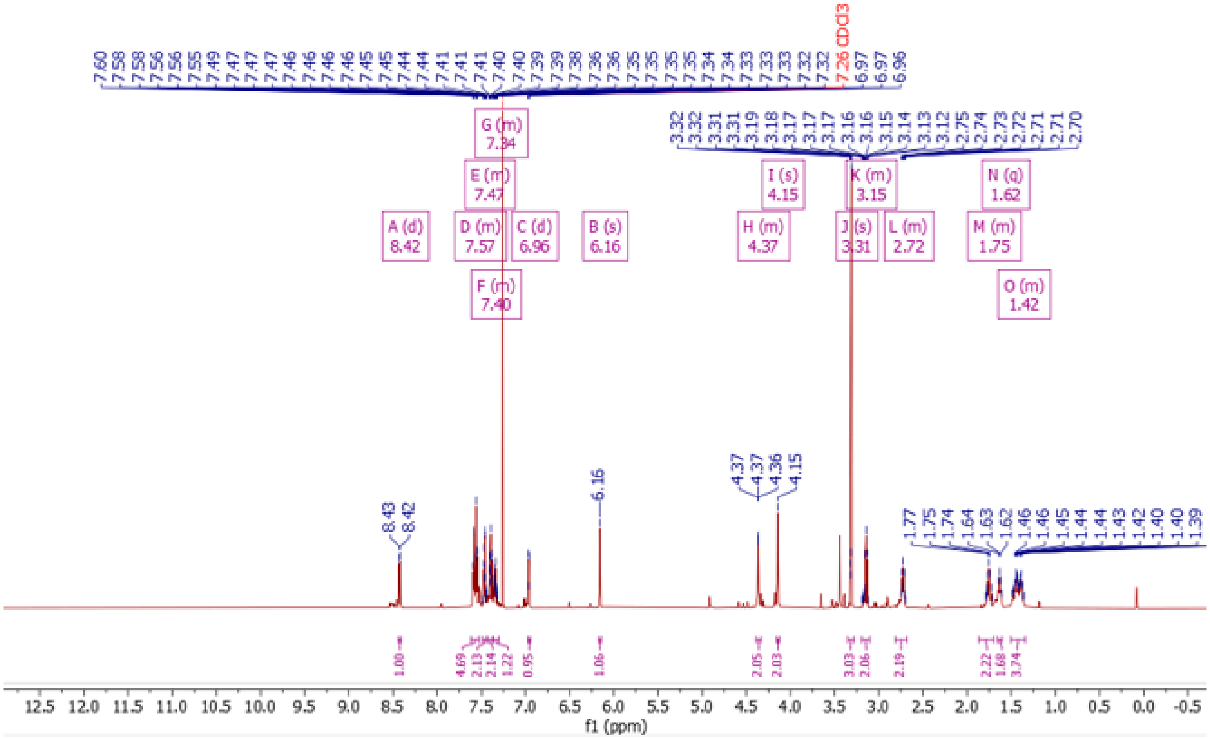

^{13}C NMR of **83**

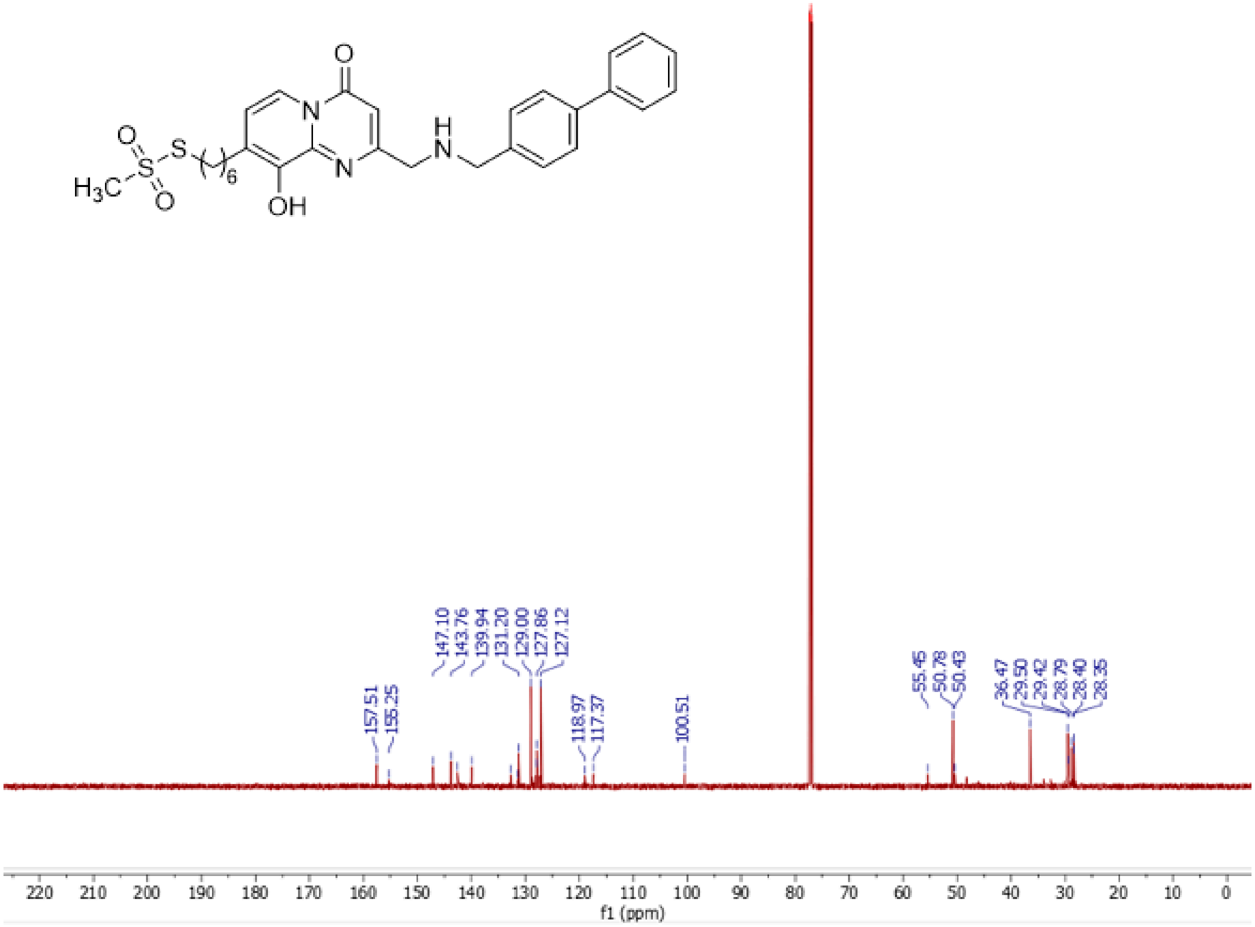

O
N
H
N
O
S
S
6
H3C
O
N
OH
157.51
155.25
147.10
143.76
139.94
131.20
129.00
127.86
127.12
118.97
117.37
100.51
55.45
50.78
50.43
36.47
29.50
29.42
28.79
28.40
28.35
220 210 200 190 180 170 160 150 140 130 120 110 100 90 80 70 60 50 40 30 20 10 0
f1 (ppm)

^{1}H NMR of **84**

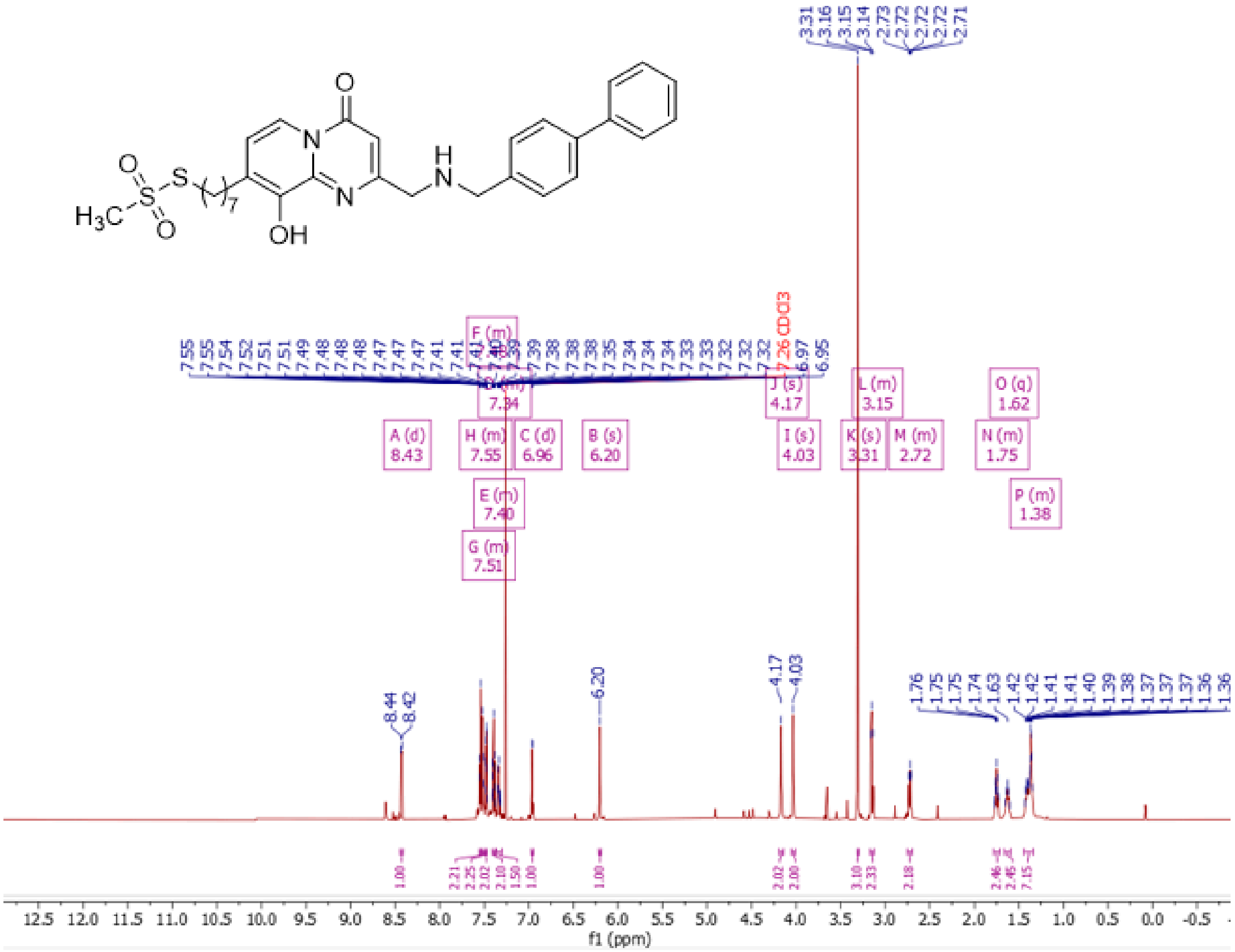

^{13}C NMR of **84**

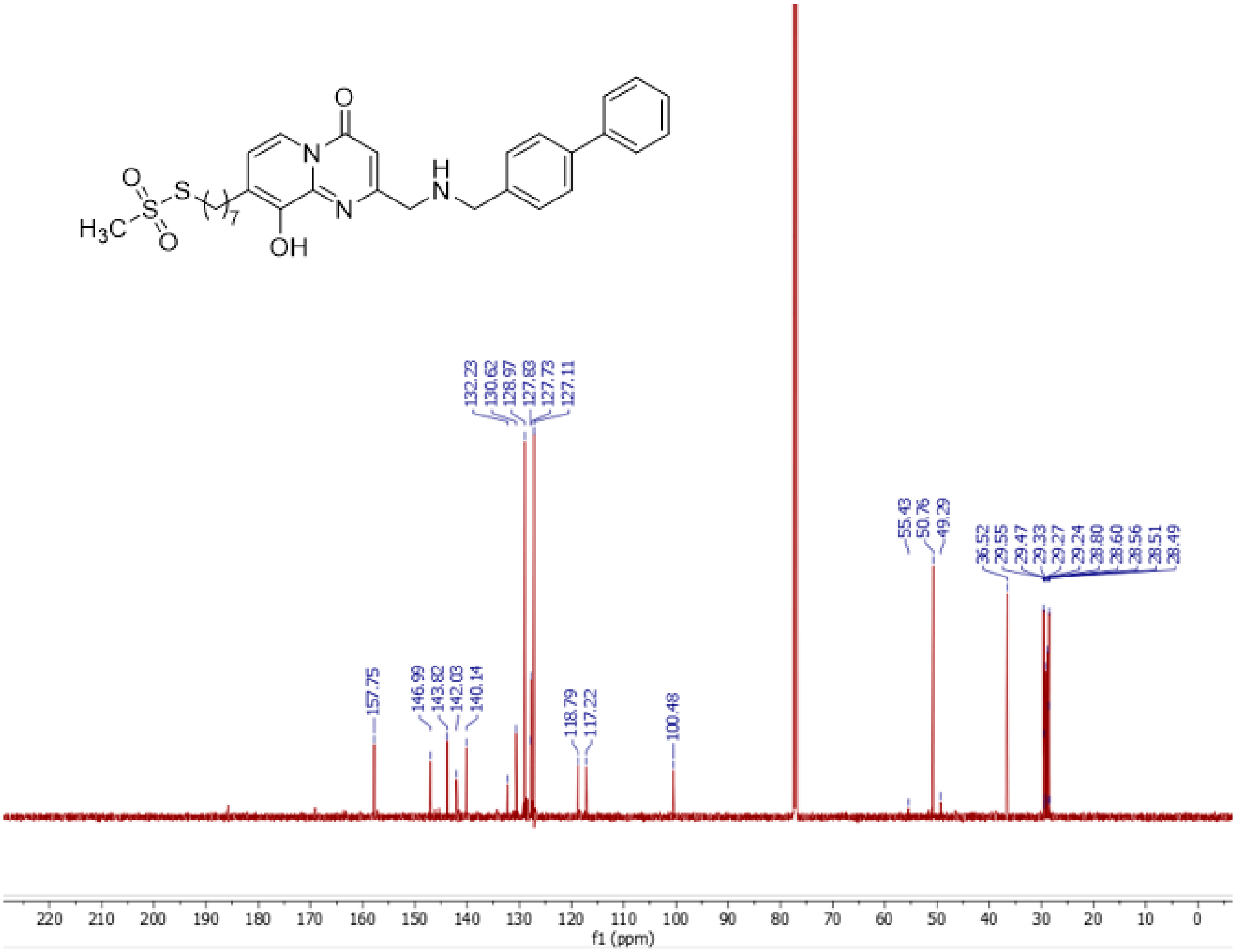

H
N
O
S
H3C
OH
7
157.75
146.99
143.82
142.03
140.14
132.23
130.62
128.97
127.83
127.73
127.11
118.79
117.22
100.48
55.43
50.76
49.29
36.52
29.55
29.47
29.33
29.27
29.24
28.80
28.60
28.56
28.51
28.49
220 210 200 190 180 170 160 150 140 130 120 110 100 90 80 70 60 50 40 30 20 10 0
f1 (ppm)
